# Synthetic Nucleosomes Reveal that GlcNAcylation Modulates Direct Interaction with the FACT Complex

**DOI:** 10.1002/anie.201603106

**Published:** 2016-06-08

**Authors:** Ritu Raj, Lukas Lercher, Shabaz Mohammed, Benjamin G. Davis

**Affiliations:** ^1^ Department of ChemistryUniversity of Oxford, Chemistry Research LaboratoryMansfield RoadOxfordOX1 3TAUK

**Keywords:** epigenetics, GlcNAcylation, nucleosomes, protein modifications, synthetic biology

## Abstract

Transcriptional regulation can be established by various post‐translational modifications (PTMs) on histone proteins in the nucleosome and by nucleobase modifications on chromosomal DNA. Functional consequences of histone O‐GlcNAcylation (O‐GlcNAc=O‐linked β‐N‐acetylglucosamine) are largely unexplored. Herein, we generate homogeneously GlcNAcylated histones and nucleosomes by chemical post‐translational modification. Mass‐spectrometry‐based quantitative interaction proteomics reveals a direct interaction between GlcNAcylated nucleosomes and the “facilitates chromatin transcription” (FACT) complex. Preferential binding of FACT to GlcNAcylated nucleosomes may point towards O‐GlcNAcylation as one of the triggers for FACT‐driven transcriptional control.

The genomic DNA of eukaryotes is packaged into a dynamic DNA–protein amalgam termed chromatin. The basic repeating unit of chromatin is the nucleosome, comprised of histone octamer complexed by approximately 150 base pairs (bp) of DNA.^1^ DNA‐templated processes, including transcriptional regulation, are, in part, controlled by post‐translational modifications (PTMs) to histone proteins.^2^ Addition of *O*‐linked β‐*N*‐acetylglucosamine (*O*‐GlcNAc) to serine and threonine residues in proteins is a nutrient‐sensitive PTM implicated in various aspects of cellular homeostasis. Deregulation of *O*‐GlcNAcylation levels has been linked to neurodegenerative diseases, cancer, and diabetes.^3^ *O*‐GlcNAc is widely observed on cytosolic and nuclear proteins^4^ and has been reported to be part of the “histone code”.^5^ *O*‐GlcNAcylation is catalyzed by a single enzyme, namely *O*‐GlcNAc transferase (OGT). The modification is highly dynamic, in line with other histone modifications, and interestingly the reverse reaction is also catalyzed by a single enzyme, specifically *O*‐GlcNAcase (OGA).

Although *O*‐GlcNAc sites have been identified on all canonical histones, the role of histone GlcNAcylation in transcriptional regulation remains elusive. Of the reported *O*‐GlcNAc sites, H2B‐Ser112‐*O*‐GlcNAc has previously been associated with indirect transcriptional regulation through promotion of H2B‐Lys120 ubiquitination that in turn facilitated transcriptional activation.5c We have previously characterized the effect of H2A‐Thr101 GlcNAcylation on nucleosome structure;^6^ H2A‐Thr101 is located at the dimer–tetramer interface of the nucleosome and GlcNAcylation at this specific site leads to destabilization of the H2A/B dimer in the nucleosome. However, H2B‐Ser112 is remote from any such critical interface and so this mechanism cannot be readily invoked. In contrast to this structural modulation by PTMs, another mechanism by which other PTMs can establish a functional output is through direct recruitment of chromatin “reader”/interactor proteins,2b yet such a direct recruitment has not yet been observed for GlcNAcylation. Mass spectrometry (MS)‐based proteomics provides an unbiased, powerful approach for both identification and quantification of such interactor proteins,^7^ in contrast to traditional Western blotting methods.^8^ Herein, we report the synthesis of modified nucleosomes containing a GlcNAcylation mimic at site H2B‐S112. These synthetic nucleosomes were used for identification of “reader” proteins by MS‐based interaction proteomics (Figure 1). These first direct interaction assessments in a nucleosomal context suggest that, in fact, GlcNAcylation at H2B‐S112 modulates the interaction between the nucleosome and the “facilitates chromatin transcript” (FACT) complex, a pivotal histone chaperone complex.

**Figure 1:**
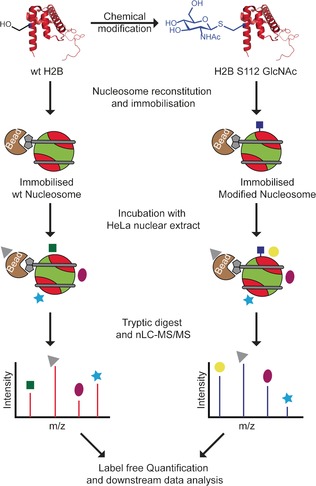
Workflow for identification of H2B‐S112 GlcNAc interactor proteins. H2B‐S112‐GlcNAc was synthesized using a “tag‐and‐modify” approach (Figure 2 b). The modified histone protein (along with other canonical histone proteins and biotinylated DNA (shown in gray)) were used for nucleosome reconstitution. Wildtype (wt) and the GlcNAcylated nucleosome were immobilized on magnetic beads (brown) via streptavidin–biotin affinity to enrich nucleosome‐binding proteins. Pooled proteins from each sample were separately digested and identified by nLC–MS/MS (nLC=nanoflow liquid chromatography). Label‐free quantification (LFQ) was applied for quantification. H2A/B dimers are shown in red, the H3/H4 tetramer is shown in green, GlcNAc shown as a blue square. The green square, dark‐red oval, yellow circle, light‐blue star, and gray triangle represent nucleosome‐binding proteins.

Of the few described histone *O*‐GlcNAcylation sites, H2B‐Ser112 is particularly interesting being both a core domain modification and due to its proximity to the nucleosome “acidic patch” (Figure 2 a), raising numerous potential mechanistic roles. The “acidic patch” is a negatively‐charged binding interface on the nucleosome surface.^9^ A number of proteins, such as LANA,^10^ IL‐33,^11^ RCC1,^12^ Sir3,^13^ HMGN2,^14^ RNF168,^15^ and RING1B/BMI1,^15^ as well as the H4 tail,^1^ are known to interact competitively with the “acidic patch” leading to remodeling of chromatin structure; we speculated that because of its proximity H2B‐Ser112 *O*‐GlcNAcylation could directly modulate binding. We tested this through the creation and characterization of a GlcNAcylated nucleosome. Although pulldown assays with certain modified nucleosomes have been used in conjugation with quantitative MS to study interacting nucleosome–protein partners,^7^, ^16^ few^6^ have investigated *O*‐GlcNAcylation because of a lack of access to pure GlcNAcylated nucleosome.

**Figure 2:**
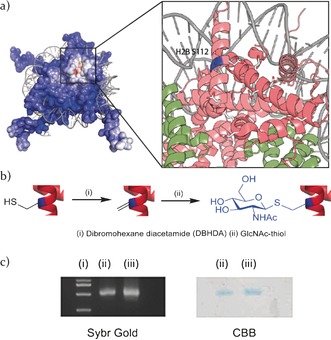
a) Electrostatic potential view of the nucleosome. Blue indicates the positively charged surface, whereas red denotes the negatively charged surface; inset: the acidic patch with H2B‐S112. b) Synthesis of GlcNAcylated H2B‐S112. H2B‐S112C was reacted (i) with DBHDA to generate H2B‐S112Dha, which upon reaction with GlcNAc‐thiol (ii) generated H2B‐S112‐GlcNAc. This was then used to reconstitute synthetically GlcNAcylated nucleosome as per Figure 1 (see the Supporting Information for full details). c) Native PAGE analysis of wt (lane ii) and GlcNAc‐modified (lane iii) nucleosome reconstitution: Sybr Gold staining (left) and Coomassie Brilliant Blue (CBB) staining (right). Lane i shows the 100 bp DNA ladder. Image of the full gel is shown in Figure S9.

For such precise functional characterization of H2B‐S112 *O*‐GlcNAcylation, access to site‐specifically GlcNAcylated protein is required. Access to homogeneous GlcNAcylated histones, and in general glycoproteins, remains a bottleneck.^17^ In vitro enzymatic *O*‐GlcNAcylation of histone proteins by OGT typically leads to incomplete and heterogeneous product mixtures.5c We have previously reported a site‐selective chemical protein modification strategy employing a “tag‐and‐modify” approach^18^ for the generation of differently modified proteins, including GlcNAcylated histone H3.^19^ Herein, by using this approach, we generated H2B‐S112‐GlcNAc protein for the reconstitution of site‐specifically GlcNAcylated nucleosomes.

To access homogeneous GlcNAcylated H2B, we expressed and purified a recombinant Ser112Cys (S112C) mutant of the *Xenopus laevis* H2B.^20^ A dehydroalanine (Dha) “tag” was site‐selectively installed at Cys112 by treating H2B‐S112C with 2,5‐dibromohexanediamide (DBHDA)19a under denaturing conditions. The resulting H2B‐S112Dha intermediate protein was reacted with GlcNAc‐thiol to yield H2B with a GlcNAc mimic installed at S112 (Figure 2 b) that bears a thioether linkage instead of the natural ether linkage; the conformation of the glycosidic bond remains similar for such linkages.^21^ We anticipated that the use of a thioglycosidic linkage, which is more resistant to corresponding glycoside hydrolases (e.g. hOGA), would allow us to ensure homogeneity even inside enzymatically active cell lysates, thereby enabling better precision as an affinity probe. Protein characterization by LC–MS and LC–MS/MS confirmed the full conversion and site‐specific GlcNAc installation (see Figures S1–S3 in the Supporting Information). Circular dichroism (CD) spectra of refolded H2B‐S112‐GlcNAc protein resembled that for wt H2B protein, indicating no gross change in secondary structure of the protein upon GlcNAcylation (Figure S4).

GlcNAcylated H2B‐S112 was used to constitute GlcNAc‐modified H2A/H2B heterodimers by refolding with histone protein H2A; similarly GlcNAc‐modified octamers were constructed by assembling GlcNAcylated H2B‐S112 with histone proteins H2A, H3, and H4 (Figure 1; Figure 2). The resulting multimer species were purified and analyzed by size‐exclusion chromatography (SEC). Similar SEC traces for both modified dimer and octamer when compared to the wt species suggested that the histone fold of the H2A/B dimer and the dimer–tetramer interface were not disrupted upon introduction of GlcNAcylation at H2B‐S112 (Figure S5). Comparison of CD spectra and melting temperature measured by variable‐temperature CD analysis of GlcNAcylated and wt H2A/B dimers revealed no significant changes in both the spectral profiles and melting temperatures (*T*_m_ S112‐GlcNAc dimer=50.73+/−0.27 °C; *T*_m_ wt=52.9+/−0.1 °C) upon GlcNAcylation at H2B‐S112, suggesting little or no influence of the modification on the structure and stability of the dimers (Figures S6, S7). Nucleosome reconstitution was accomplished by the salt‐gradient dialysis method using 145 bp DNA and biotinylated DNA containing the strong “601” nucleosome positioning sequence.^16^, ^22^ The reconstitution of nucleosome was analyzed on native PAGE gels (Figure 2 c). Similar reconstitution yield and mobility on the PAGE gel for the modified nucleosome as compared to the wt nucleosome also suggested no major structural changes upon introduction of the modification, as expected for a surface‐exposed site. Reconstituted nucleosomes were further analyzed by CD spectroscopy. The CD spectra for both the reconstituted nucleosomes were essentially identical and the melting temperatures measured by variabletemperature CD analysis were similar (at 220 nm: *T*_m_ wt= 73.25+/−0.61 °C, *T*_m_ GlcNAcylated=74.63+/−1.16 °C; at 260 nm: *T*_m_ wt=71.86+/−0.25 °C, *T*_m_ GlcNAcylated=70.63+/−0.36 °C) suggesting that GlcNAcylation at the site does not affect the stability of the nucleosome (Figure S10–S14). These combined data suggest that the differential stability mechanism observed for H2A‐T101 GlcNAcylation^6^ does not operate in H2B‐S112 GlcNAcylation.

As represented in Figure 1, H2B‐S112‐GlcNAc‐modified nucleosome (bait) and wt nucleosome (control) were immobilized on magnetic streptavidin beads. These were then incubated with HeLa cell nuclear extract for affinity enrichment of nucleosome‐binding protein partners. Non‐specifically bound proteins were removed by washing. The enriched nucleosome‐binding proteins were digested in‐solution; the resulting peptide mixtures were separated and analyzed by ultra‐high performance LC (UHPLC) coupled to a hybrid quadrupole‐orbitrap (Q‐Exactive) mass spectrometer. To obtain a robust data set, all pulldown experiments were performed as three independent biological replicates. MaxLFQ^23^ (a MS‐based label free quantitation (LFQ) algorithm) analyses allowed us to identify and quantify 584 protein groups. Experimental correlation among LFQ intensities within group and across replicates were monitored using the Pearson correlation coefficient (Figure S15). False discovery rate (FDR) based t‐test statistical analyses revealed FACT subunits, suppressor of Ty (SPT16), and structure specific recognition protein 1 (SSRP1) as being both the statistically most significant and the most enriched interacting protein partners for H2B‐S112‐GlcNAc‐modified nucleosome (Figure 3; see also Table S1 and Figure S16).

**Figure 3:**
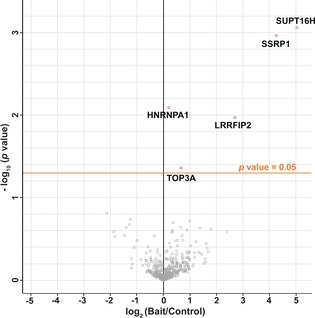
H2B‐S112 GlcNAcylation modulates binding of FACT subunits to nucleosome. Intensity difference amongst protein groups in the H2B‐S112‐GlcNAc nucleosome sample (bait) over wt nucleosome sample (control) against logarithmized *p* value of the FDR‐based t‐test is shown in the volcano plot. Threshold for *p* value (<0.05) is denoted by an orange line. Statistically significant proteins are denoted in red by their gene names.

The FACT complex is a replication^24^ and transcription^25^ factor functioning in various chromatin processes. FACT is a heterodimer protein complex consisting of two subunits (SSRP1 and SPT16) and displays histone chaperone activity.^26^ FACT plays an important role in chromatin remodeling by modulating nucleosome stability and thereby DNA accessibility. FACT can interact with multiple sites on the nucleosome^27^ and can decrease the nucleosomal barrier allowing productive transcriptional elongation, primarily by chaperoning the H2A/B dimer.^28^

Interestingly, we have previously shown that H2A‐T101 GlcNAcylation can destabilize H2A/B dimers in nucleosomes.^6^ We proposed this destabilization as a separate structural mechanism to facilitate transcriptional elongation in a manner that is complementary to FACT recruitment. In addition, FACT is known to stimulate and function cooperatively with H2B‐K120 monoubiquitination,^29^ which has previously been suggested to be S112GlcNAc‐dependent in the regulation of transcriptional elongation. We did not observe the ligase responsible for H2B‐K120 ubiquitination BRE1A/B as suggested by Fujiki et al.5c in our pulldowns both by LC–MS and Western blot (Figure S17), suggesting that this interaction is not strong enough to be detected under these conditions (see the Supporting Information). Since FACT has been reported to associate with both nucleosomes and separate histone proteins, we wanted to investigate how the presence of different components influences the FACT‐with‐GlcNAc interaction. For this, we performed pulldowns with recombinant FLAG‐tagged *Xenopus laevis* H2B containing the chemically installed S112‐GlcNAcylation, both using isolated GlcNAcylated‐H2B protein and also a reconstituted GlcNAcylated‐H2A/B dimer in a similar way to that tested with the nucleosome (Figure S20).

This experiment required the construction of a new H2B protein substrate bearing a suitable affinity motif for retrieval. Thus, *N*‐terminally FLAG‐tagged wt‐H2B (FLAG‐H2B) and the corresponding S112C mutant (FLAG‐H2B‐S112C) were designed, expressed, and purified in a similar manner to before. Essentially identical two‐step GlcNAcylation chemistry (Figure 2 b) to that used for H2B‐S112C (Figure 1) also proved equally robust and successful for GlcNAcylation of FLAG‐H2B‐S112C (See the Supporting Information and Figures S21, S22). FLAG‐tagged wt H2B and H2B‐S112‐GlcNAc monomers were refolded and, again, CD analyses revealed no significant structural changes upon modification (Figure S23).

The corresponding interactomes were analyzed using quantitative MS, as earlier. The H2B monomers were immobilized on anti‐FLAG magnetic beads and used to affinity enrich the interacting partners from nuclear extract (prepared under non‐reducing conditions, see the Supporting Information); experiments were performed in duplicate. MS‐based proteomics allowed us to identify and quantify 948 proteins, amongst which FANCI, INF2, and COAX6A1 were the most significant interacting protein partners for H2B‐S112‐GlcNAc protein as compared to wt‐H2B. (see Table S2, Figure S24). Notably, we did not see a significant enrichment of FACT upon H2B‐S112 GlcNAcylation. We were also not able to detect BRE1A in these experiments, although we do detect BRE1A by MS in the nuclear extract. Next, FLAG‐tagged H2B‐S112‐GlcNAc and FLAG‐tagged wt‐H2B were each combined with wt‐H2A protein to reconstitute FLAG‐tagged, wt, and GlcNAcylated H2A/B heterodimers, essentially as before. Again using quantitative MS, we identified 886 interacting proteins in both samples (see Table S3 and Figure S25). As with the interactome data with the monomer, we did not see any significant enrichment of FACT upon GlcNAcylation and no BRE1A was observed. Together these data suggest a context‐dependent interaction; thus, the difference in our observations here to those published previously5c appears to lie in our use of an intact nucleosomal structure as opposed to the prior use of isolated, partially GlcNAcylated protein (this might also be due to preferential binding of isolated H2B by different histone chaperones, occluding the GlcNAcylation site).

Based on our findings, we propose a possible updated mechanism facilitating ubiquitination of H2B‐Lys120 upon GlcNAcylation at H2B‐Ser112 (Figure 4). This speculative mechanism is the simplest that is consistent with the data gathered here, although, of course, others cannot be discounted. GlcNAcylation of histone H2B at Ser112 by OGT leads to FACT association. FACT recruitment results in nucleosome remodeling making the H2A/B dimer accessible for BRE1A ubiquitination. FACT can in turn directly or indirectly recruit BRE1A complex (RNF20/40) facilitating ubiquitination of H2B‐Lys120.^29^, ^30^ In agreement with this, FACT is required in vivo^30^ for BRE1A/B localization to chromatin in DNA damage responses. In addition, many residues in the acidic patch have been shown to be essential for H2B ubiquitination,^31^ suggesting that this surface is important for the anchoring of BRE1A/B. Strong enrichment of FACT complex may also point towards GlcNAcylation as a trigger for FACT‐driven transcription processes as well as a “relaxed” chromatin state facilitating transcription elongation. While we expected to find BRE1A/B in our pulldown experiments, a lack thereof might be explained by the previous observation that both active transcription (dependent on the presence of dNTPs) and FACT is necessary for H2B‐K120 ubiquitination. It might be that even a FACT‐bound nucleosome is not sufficient for BRE1A/B association and that further structural changes are required.

**Figure 4:**
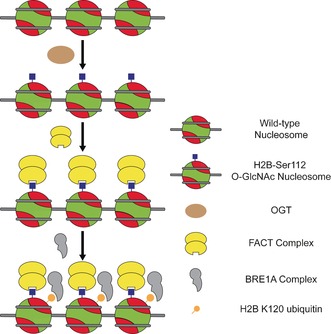
A proposed molecular mechanism for FACT‐mediated H2B‐Lys120 ubiquitination upon GlcNAcylation at H2B‐Ser112.

In summary, by using a “tag‐and‐modify” approach we have synthesized GlcNAcylated H2B histones and corresponding nucleosomes. Coupled with interaction proteomic analyses, we were able to explore the mechanistic details of a transcriptionally relevant segment involving *O*‐GlcNAcylation. In contrast to H2A‐T101 GlcNAcylation, H2B‐S112 GlcNAcylation does not affect nucleosome assembly, but directly influences the nucleosome interactome, highlighting different possible signaling mechanisms for histone GlcNAcylation. It is also important to note that we use here a designed nonhydrolyzable mimetic that despite the anticipated similarity^21^ might give rise to unexpected artefacts. The chemical synthetic approach used here can be employed in principle for the generation of differently (and multiply) modified nucleosomes to complement other biochemical approaches and/or other powerful, multiplexed methods, such as those achievable by, for example, expressed protein ligation^32^ or native chemical ligation.^33^ These synthetic nucleosomes in combination with MS‐based proteomics can elucidate the role of various PTMs as well as revealing “cross‐talk” between PTMs. In turn, we anticipate that they will allow elucidation of the key players to create a precise mechanistic picture of this biology at the molecular level. The field of chromatin biology has relied heavily on short peptides and isolated proteins that are mere fragments of true nucleosomal contexts; the work presented herein, as well as other reports,^34^ suggests that the use of suitably complex probe molecules that provide the correct context may prove vital for relevant interrogation.

## Supporting information

As a service to our authors and readers, this journal provides supporting information supplied by the authors. Such materials are peer reviewed and may be re‐organized for online delivery, but are not copy‐edited or typeset. Technical support issues arising from supporting information (other than missing files) should be addressed to the authors.

Supplementary
