# Supplementary material for: Synthetic Nucleosomes Reveal that GlcNAcylation Modulates Direct Interaction with the FACT Complex
Source: Angew Chem Int Ed Engl. 2016 Jun 8;55(31):8918–22. doi: 10.1002/anie.201603106 (PMC5111754; doi:10.1002/anie.201603106)
Supplement: Supplementary file 1 — Supplementary [file ANIE-55-8918-s001.pdf]

Angewandte Chemie
Eine Zeitschrift der Gesellschaft Deutscher Chemiker

Supporting Information

Synthetic Nucleosomes Reveal that GlcNAcylation Modulates Direct Interaction with the FACT Complex

*Ritu Raj, Lukas Lercher, Shabaz Mohammed, and Benjamin G. Davis**

anie_201603106_sm_miscellaneous_information.pdf

Supporting Information

Expression and purification of Histone proteins

pET3 bacterial plasmids encoding all canonical *Xenopus laevis* histones were kindly donated by Dr Rob Klose, University of Oxford, Oxford. Wt histone proteins (H2A, H2B, H3 and H4) were expressed in E. coli BL21 (DE3)pLysS competent cell and purified by size-exclusion (HiLoad 26/600 Superdex 200, GE Healthcare Life Sciences) followed by ion-exchange (HiTrap SP HP 5 mL, GE Healthcare Life Sciences) chromatography as described previously.[1,2] Purified histone proteins were lyophilized and stored at – 80 °C.

The H2B S112C mutation was carried-out using the QuikChange Site-directed Mutagenesis kit (Agilent) following manufacturer's protocol. The mutation was verified by sequencing. H2B S112C protein was expressed and purified using same protocol as for wt proteins.

Site-selective chemical protein modification

Synthesis of DBHDA and GlcNAc-thiol has been previously described.[2]

(i) Synthesis of H2B Ser112Dha

5.10 mg of H2B S112C protein (0.37 µmol) was taken in 500 µL of freshly prepared reaction buffer (5 M Gd.HCl, 300 mM HEPES pH 7.5). 6 mg of DTT was added to the protein solution. The protein solution was reduced at 37 °C for 30 minutes. The sample was desalted using a pre-equilibrated PD minitrap G-25 column (GE Healthcare Life Sciences). Desalted protein was eluted in 1 mL of reaction buffer.

100 equivalent of DBHDA solution (35 µmol, 0.50 M in DMF, 70 µL) was added to 950 µL (0.35 µmol) of reduced protein sample. The reaction mixture was incubated at room temperature for 30 minutes and then at 37 °C. The progress of reaction was monitored by LC-MS.

LC-MS analysis was performed using a Prominence HPLC system (Shimadzu) connected to an ESI-TOF Micromass LCT Classic (Waters) mass spectrometer. The protein sample was loaded onto a Chromolith® FastGradient RP-18 column (Merck) with solvent A (0.1 % Formic Acid in water). The protein sample was analyzed using a gradient from 20 % to 60 % solvent B (0.1 %

Acetonitrile) in 4.5 minutes at a flow rate of 0.4 mL /minute. The capillary and cone voltage for the electrospray source was kept at 3000 V and 25 V respectively. The raw MS data was processed using MassLynx software (Waters, version 4.1). Myoglobin protein was used for creating calibration file. Deconvolution of spectra was done using in-built MaxEnt algorithm.

The reaction reached completion after 3 hours yielding H2B Ser112Dha (Supplementary Figure S1). The reaction mixture was desalted using a pre-equilibrated PD miditrap G-25 column (GE Healthcare Life Sciences). Protein sample was eluted in 1.5 mL of reaction buffer.

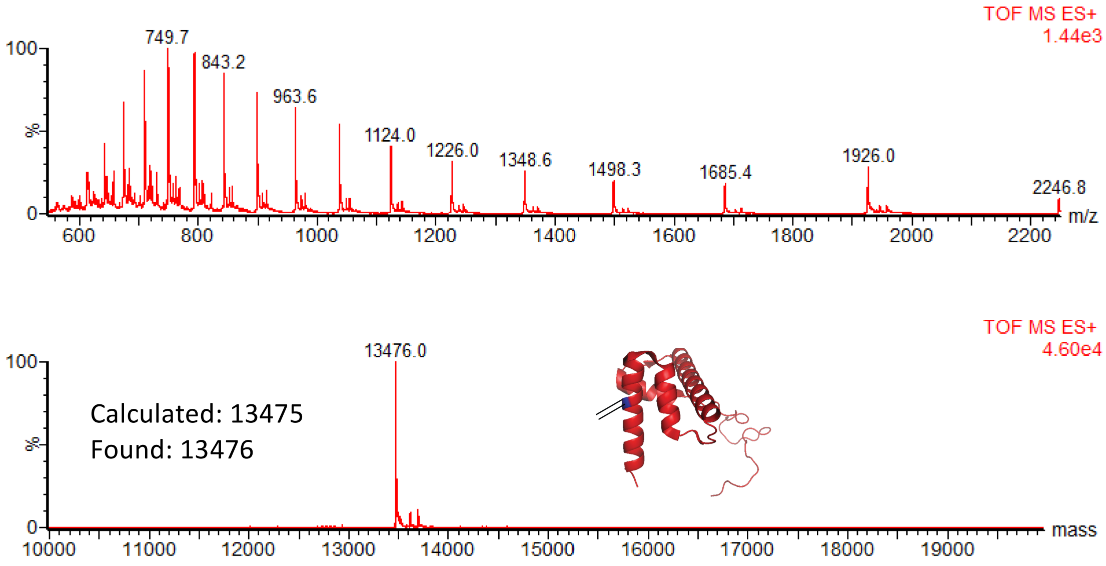

Figure S1. LC-MS Spectrum of H2B S112Dha

(ii) Synthesis of H2B Ser112 GlcNAc

1 mL of desalted H2B S112Dha (2.50 mg, 0.185 µmol) in reaction buffer was taken in an eppendorf tube. 400 equivalent of GlcNAc-thiol (17.55 mg, 74 µmol) was added to the protein sample as solid. The reaction mixture was incubated at room temperature for 30 minutes and then at 37 °C. The progress of reaction was monitored by LC-MS as described earlier. The reaction reached completion in 3 hours resulting in first homogeneous synthesis of H2B S112 GlcNAc (Supplementary Figure S2). The reaction mixture was desalted using a pre-equilibrated PD miditrap G-25 column (GE Healthcare Life Sciences). Protein sample was eluted in 1.5 mL of unfolding buffer (7 M Gd.HCl, 10 mM Tris pH 7.5, 1 mM DTT, 1 mm EDTA) and stored at – 20 °C till further manipulation.

Site-selective installation of GlcNAc at H2B S112 was verified by LC-MS/MS (Supplementary Figure S3). 25 µg of GlcNAcylated H2B protein was buffer exchanged against 100 µL of denaturing buffer (2 M Urea, 50 mM Ammonium Bicarbonate). The sample was reduced

with DTT (2 µL of 200 mM solution in denaturing buffer) at 56 °C for 20 minutes, followed by alkylation with iodoacetamide (4 µL of 200 mM solution in denaturing buffer) at room temperature for 30 minutes. Alkylation was quenched by further addition of 2 µL of DTT solution. Trypsin was added to the sample in 1:50 (Enzyme: Protein (w/w)) ratio. The sample was incubated at 37 °C for 16 hours. The digested sample was diluted to a final concentration of 100 fmol/ µL using 10 % Formic Acid solution.

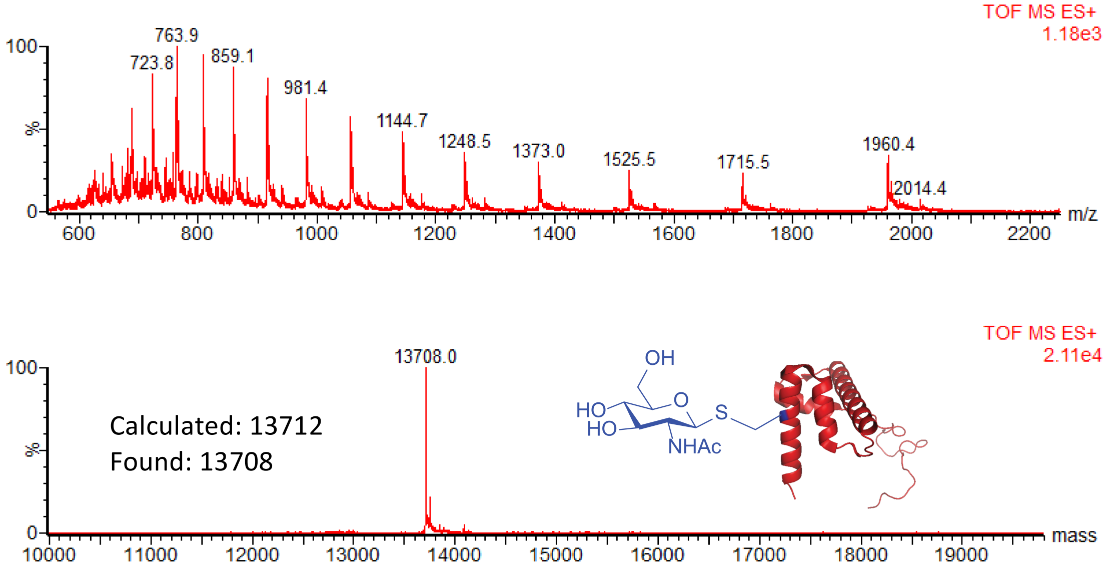

Figure S2. LC-MS Spectrum of H2B S112 GlcNAc

Resulting tryptic peptides were analyzed on an EASY-nLC 1000 UHPLC system (Proxeon) connected to an Orbitrap Elite mass spectrometer (Thermo Fischer Scientific) possessing an EASY-Spray nano-electrospray ion source (Thermo Fischer Scientific). The peptides were trapped on an in-house packed guard column (75 µm i.d. x 20 mm, reprosil C18, 3µm, 120 Å) using solvent A (0.1% Formic Acid, 5 % DMSO in water) at a pressure of 500 bar. The peptides were separated on an EASY-spray Acclaim PepMap® analytical column (75 µm i.d. × 500 mm, RSLC C18, 2 µm, 100 Å) using a linear gradient (length: 58 minutes, 8 % to 30 % solvent B (0.1% formic acid, 5 % DMSO in acetonitrile), flow rate: 200 nL/min). The separated peptides were electrosprayed directly into the mass spectrometer operating in a data-dependent mode using a HCD/ETD based method.[3,4] Full scan MS spectra (scan range 350-1500 m/z, resolution 30000, AGC target 1e6, maximum injection time 250 ms) and subsequent HCD MS/MS spectra (resolution 15000, AGC target 3e4, maximum injection time 500 ms) of 5 most intense peaks were acquired in the Orbitrap. HCD fragmentation was performed at 30 % of normalized collision energy and the signal intensity threshold was kept at 500 counts. If peaks at any of *m/z* 138.0546, 168.0563 or 204.0867 were detected among

top 20 most intense peak of a HCD spectrum, an ETD MS/MS spectrum of the precursor was triggered. ETD spectra (AGC cation target 5e4, AGC anion target 2e5, cation maximum injection time 100 ms) were acquired in the Iontrap.

The raw data was processed using Byonic™ software (Protein Metrics, version 2.0-3). The raw MS file was searched against H2B S112C sequence as well as list of common contaminants. Trypsin with a maximum number of 2 missed cleavages was selected as the protease. Carbamidomethylation (Cysteine) and GlcNAc (Cysteine) were set as variable modifications. Precursor mass tolerance was set as 10 ppm. Fragment mass tolerances for HCD and ETD were set to 50 ppm and 0.8 Da respectively. All spectra were manually validated.

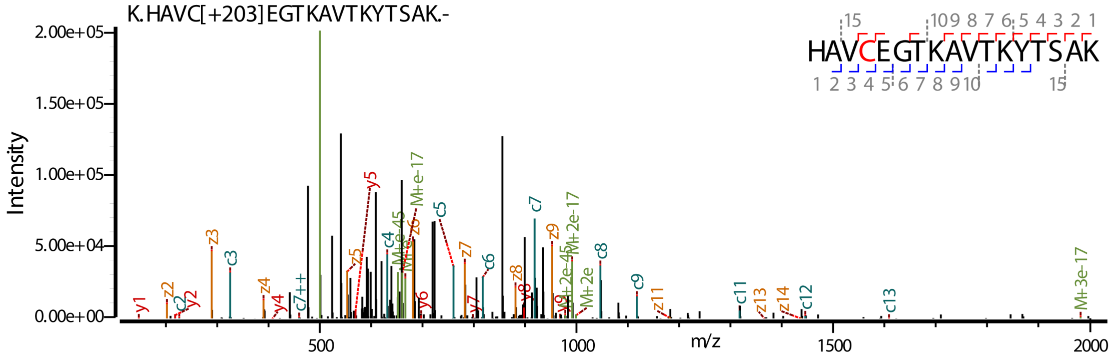

Figure S3. ETD MS/MS Spectrum of H2B S112 GlcNAc peptide

CD analysis of the H2B monomers

Wild-type H2B protein and H2B-S112 GlcNAc protein were buffer exchanged into 20 mM $NaHPO_4$ pH 7.2, 200 mM KCl, 0.1 mM EDTA by dialysis. CD spectra were recorded using a Chirascan CD-spectrophometer (Applied Photophysics). For the measurement, 200 µL of 10 µM protein samples were taken in a micro-cuvette (1 mm path length). CD spectra were recorded from 200–340 nm with a step size of 0.5 nm and 1 s per point. The initial spectra were recorded in triplicates. The three traces were averaged and smoothed using in-built Savitzky-Golay function (window size 4). The raw data were imported from the Pro-data as CSV and further processed using OriginPro. Prior to plotting the spectra, background for the buffer was subtracted.

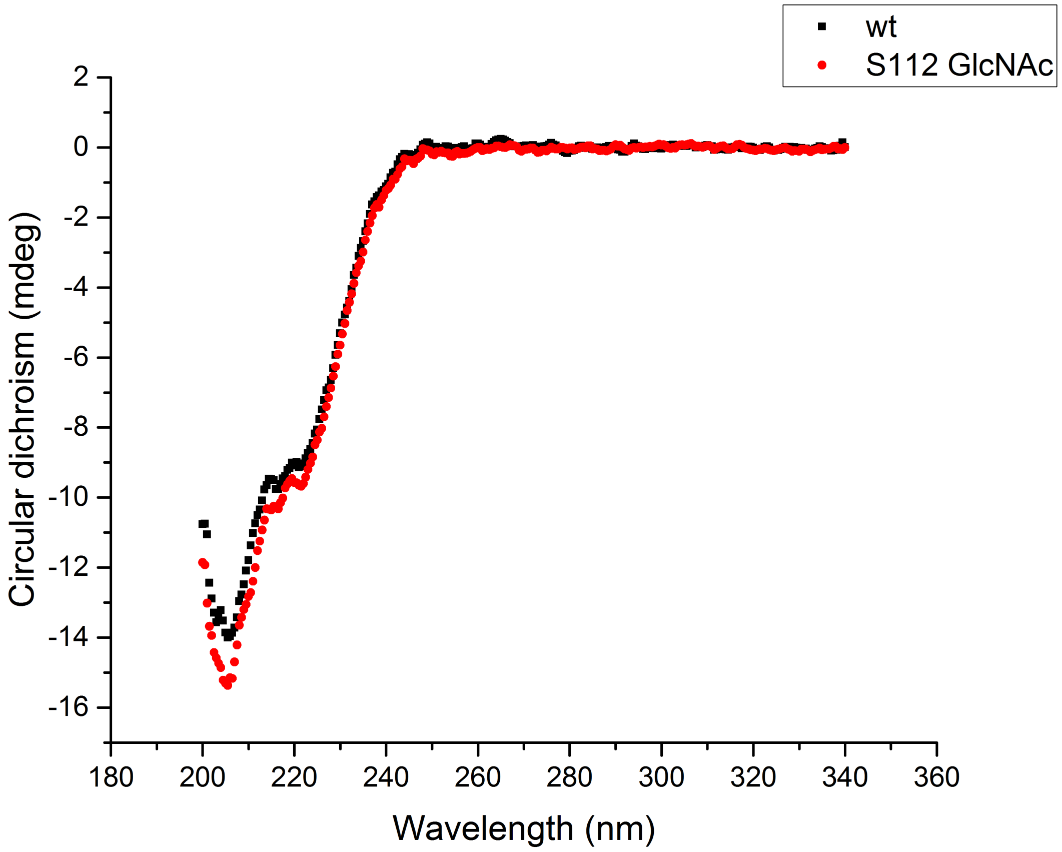

Figure S4. CD analysis of refolded wt H2B monomer (black) and H2B S112 GlcNAc monomer (red).

Histone complex reconstitution

Histone complexes were refolded as described earlier.[1,5,6] For reconstitution purpose, all canonical histone were dissolved in unfolding buffer (7 M Gd.HCl, 10 mM Tris pH 7.5, 1 mM DTT, 1 mm EDTA); H2B S112 GlcNAc modified protein was desalted in unfolding buffer during synthesis itself. Concentration of each protein sample was measured using Nanodrop. For dimer reconstitution, equal amount of H2A and H2B/H2B S112 GlcNAc proteins were mixed to a final protein concentration of 1-1.5 mg/mL. The protein mixture was loaded on a dialysis cassette (MWCO 3500). The sample was dialysed thrice against 2 L of refolding buffer (2 M NaCl, 10 mM Tris pH 7.5, 1 mM DTT, 5 mM β-ME) for at least 3 hours each. After this, the sample was centrifuged to remove precipitate, if any, and refolded histone dimers were purified by gel filtration chromatography (HiLoad 16/600 Superdex 200 column, (GE Healthcare Life Sciences)) in refolding buffer (Supplementary Figure S5). The purified dimers were concentrated and stored in 50 % glycerol at – 20 °C for further use.

Wt or H2B S112 GlcNAc modified octamers were reconstituted by mixing H2B or H2B S112 GlcNAc proteins with H2A, H3 and H4 proteins in a ratio of 1, 0.9 and 0.9 respectively to a final protein concentration of 1-1.5 mg/mL. The protein mixture was loaded on a dialysis cassette (MWCO 3500). The sample was dialysed thrice against refolding buffer for at least 3 hours each. Octamer was purified by gel filtration chromatography as described earlier (Supplementary Figure S5). The purified octamers were concentrated and stored in 50 % glycerol at – 20 °C for further use.

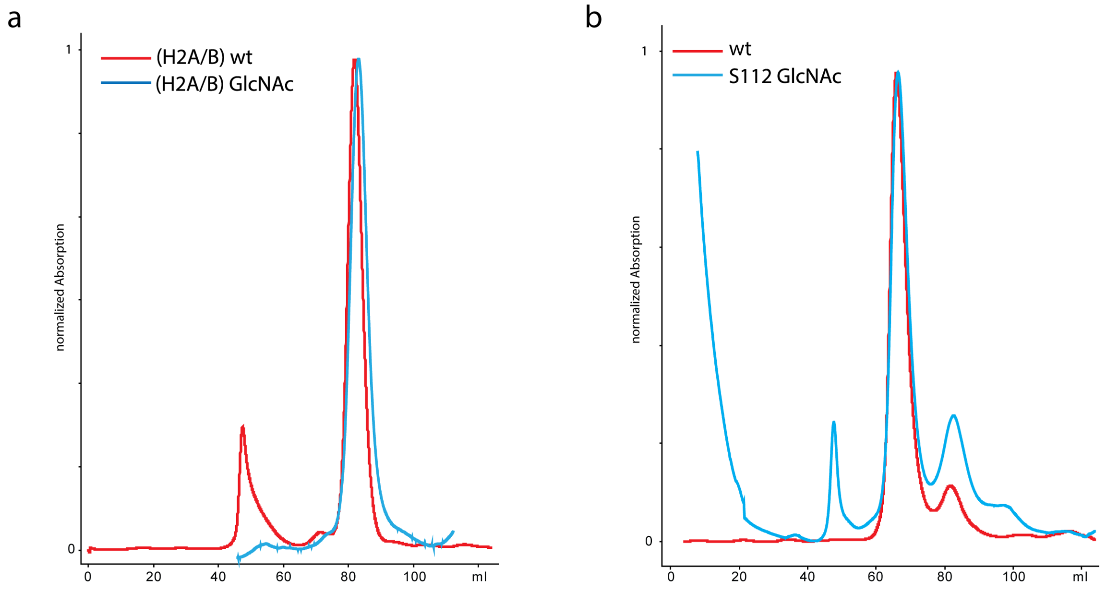

Figure S5. Reconstitution of Histone complexes. (a) Overlapped size exclusion traces of wild type (red) and H2B S112 GlcNAc-modified (blue) dimer. (b) Overlapped size exclusion traces of wild type (red) and H2B-S112-GlcNAc-modified octamer (blue).

CD analysis of the H2A/B dimers

CD analysis was essentially performed as described previously.[7] Wild-type H2A/B dimer and H2B-S112 GlcNAc modified dimer were buffer exchanged into 20 mM $NaHPO_4$, pH 6.7, 1 mM EDTA, 150 mM NaCl by dialysis. CD spectra were recorded using a Chirascan CD-spectrophometer (Applied Photophysics). For the measurement, 200 µL of 6.2 µM protein samples were taken in a micro-cuvette (1 mm path length). CD spectra were recorded from 200–340 nm with a step size of 0.5 nm and 0.5 s per point. The initial spectra were recorded in triplicates. The three traces were averaged and smoothed using in-built savitzky-golay function (window size 4). The raw data were imported from the Pro-data as CSV and further processed using OriginPro. Prior to plotting the spectra, background for the buffer was subtracted.

For melting curves the temperature was increased stepwise from 25–90 ˚C in 1 ˚C steps with a hold time of 60 s. The temperature of the sample as measured by a sample probe was recorded for each step. The raw data were exported from the Pro-data as CSV and further processed using OriginPro. The melting temperature was extracted by plotting the CD at 220 nm against temperature and fitting using a Boltzmann sigmoidal function. Reported are the fitted parameters including the standard error.

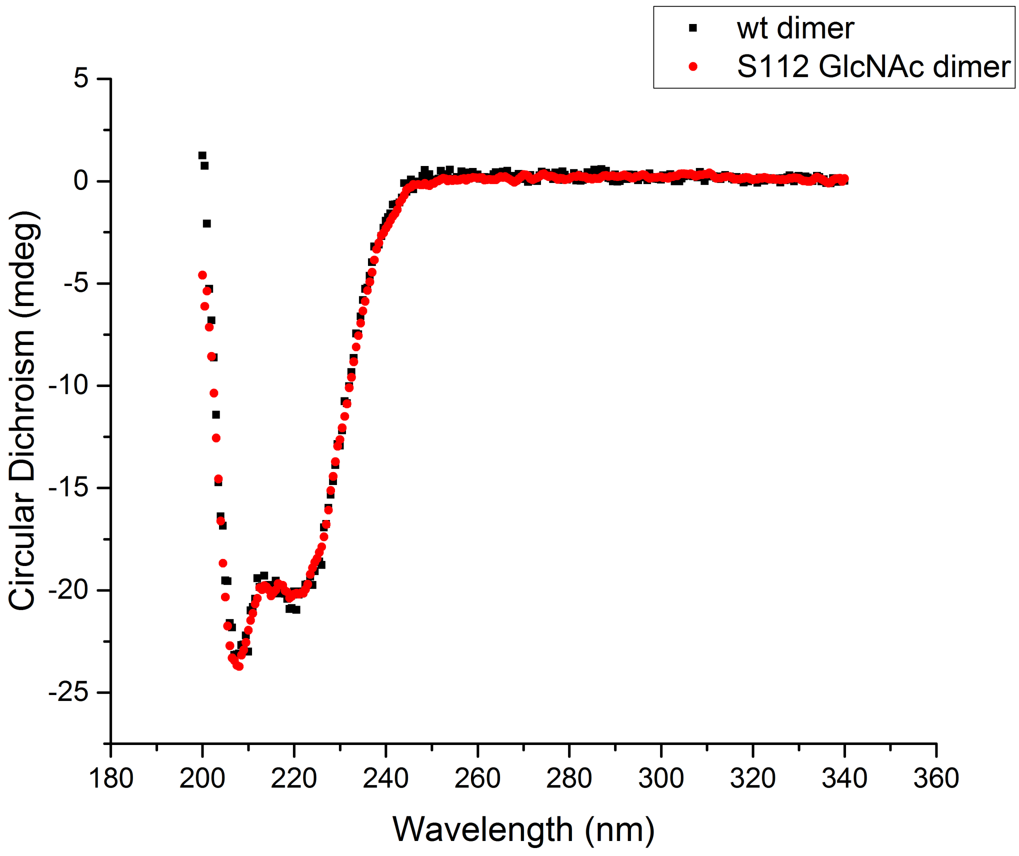

Figure S6. CD analysis of refolded wt H2A/B dimer (black) and S112 GlcNAc modified dimer (red).

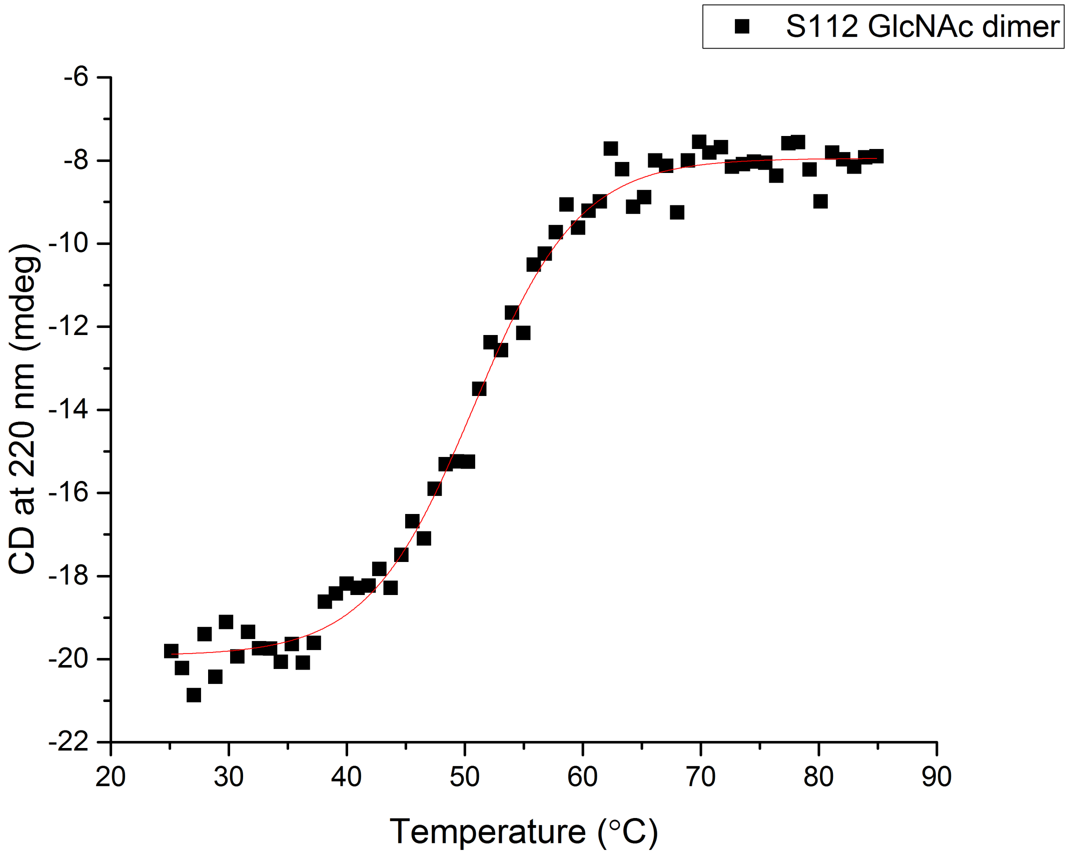

Figure S7. Melting profile of S112 GlcNAc modified dimer. The melting temperature was extracted by monitoring the CD at 220 nm.

Nucleosome reconstitution

For immobilisation, nucleosomes were reconstituted using 186 bp long biotinylated DNA. The bacterial plasmid encoding 186 bp '601' DNA was a kind gift from Dr. Till Bartke, Imperial College, London. The DNA was excised and biotinylated as described.[1,8] Wt / modified nucleosomes were reconstituted by combining wt/modified octamer (in refolding buffer) and biotinylated DNA in 1 : 0.85 molar ratio. The samples were then loaded onto 100 µL dialysis tubing. The samples were dialysed against a series of step-gradients from high-salt buffer (2 M KCl, 10 mM Tris pH 7.5, 1 mM EDTA, 1 mM DTT) to low-salt buffer (250 mM KCl, 10 mM Tris pH 7.5, 1 mM EDTA, 1 mM DTT).

After dialysis, the samples were transferred to eppendorf tubes and centrifuged to remove any precipitation, if any. The reconstituted nucleosomes were analysed by Native PAGE

using Novex TBE Gels (6%, Invitrogen). The TBE gel was pre-run in 0.5 % TBE buffer at 150 V for 90 minutes at 4 °C. 0.5-1 μg (1 μL) of nucleosome samples along with 5 μL of 6 % sucrose solution were loaded per well. 100 bp DNA ladder was also added. The gel was run at 150 V for 90 minutes. The gel was stained with SYBR® Gold nucleic acid gel stain (Invitrogen) followed by Coomassie Brilliant Blue stain.

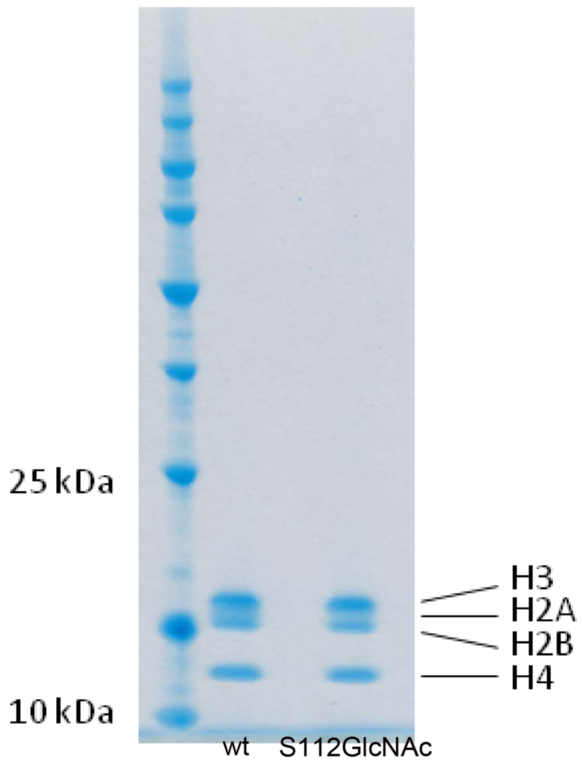

Figure S8. SDS-PAGE analysis of the stoichiometry of reconstituted nucleosomes showing constituent histone proteins.

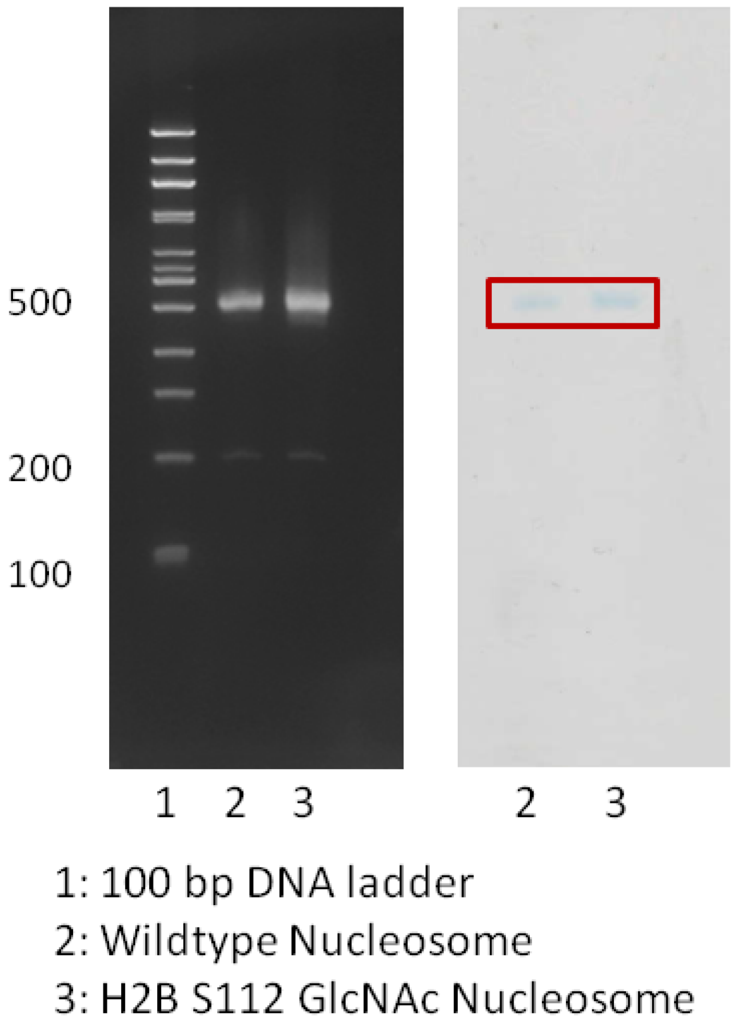

Figure S9. Full image of full gel used in Fig. 2c: native PAGE analyses of wt and H2B-S112-GlcNAcylated nucleosome reconstitution. Reconstituted nucleosomes were subjected to Sybr Gold staining (left) and Coomassie Brilliant Blue (CBB) staining (right).

CD analysis of the nucleosomes

Nucleosome CD analysis was essentially performed as described previously.[7] Nucleosomes (1 µM) were buffer exchanged into 10 mM $NaHPO_4$ pH 7.5, 1 mM EDTA, 150 mM NaCl. For the measurement , 200 µl of nucleosome solution in a micro cuvette (1 mm path length) was used. CD spectra were recorded from 200–340 nm with a step size of 0.5 nm and 1 s measurement per point. Temperature was ramped stepwise from 25–90 ˚C in 1 ˚C steps with 120 s at each temperature. The melting temperature was extracted by plotting the CD at 220 and 260 nm against temperature and fitting using a Boltzmann sigmoidal function. Reported are the fitted parameters including the standard error.

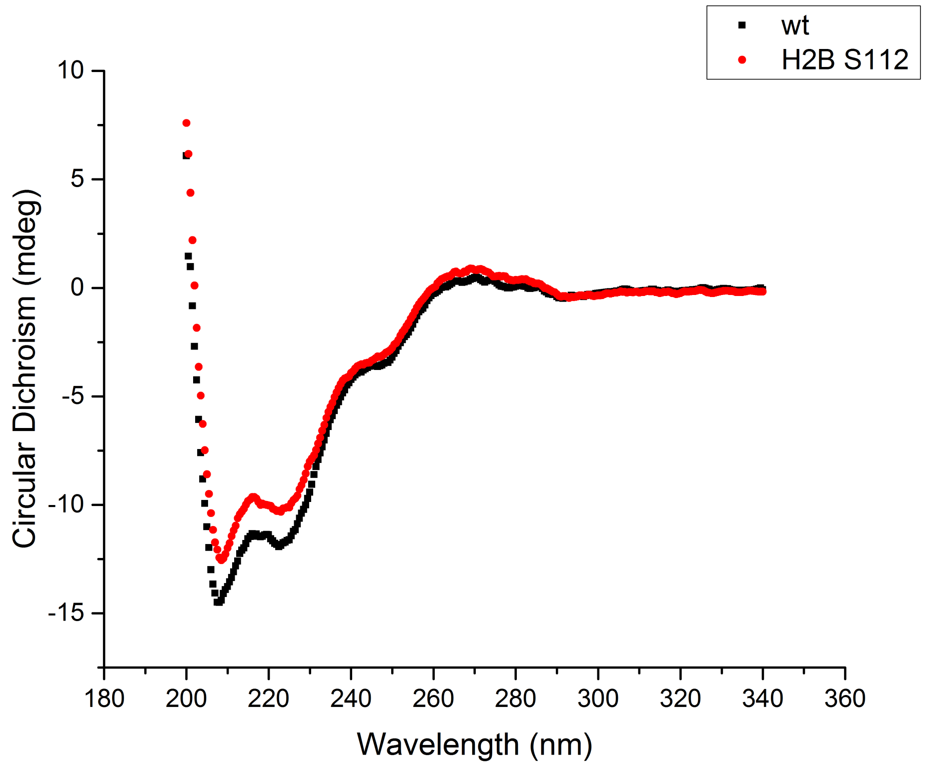

Figure S10. CD analysis of wt (black) and H2B S112 GlcNAc modified nucleosome (red).

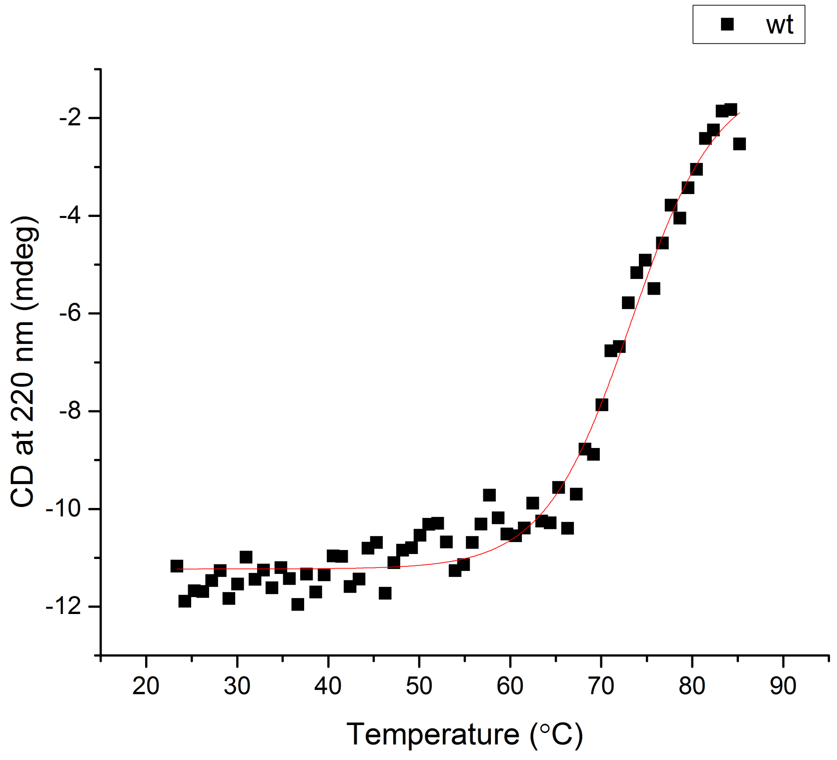

Figure S11. Melting profile of wt nucleosome. The melting temperature was extracted by monitoring the CD at 220 nm.

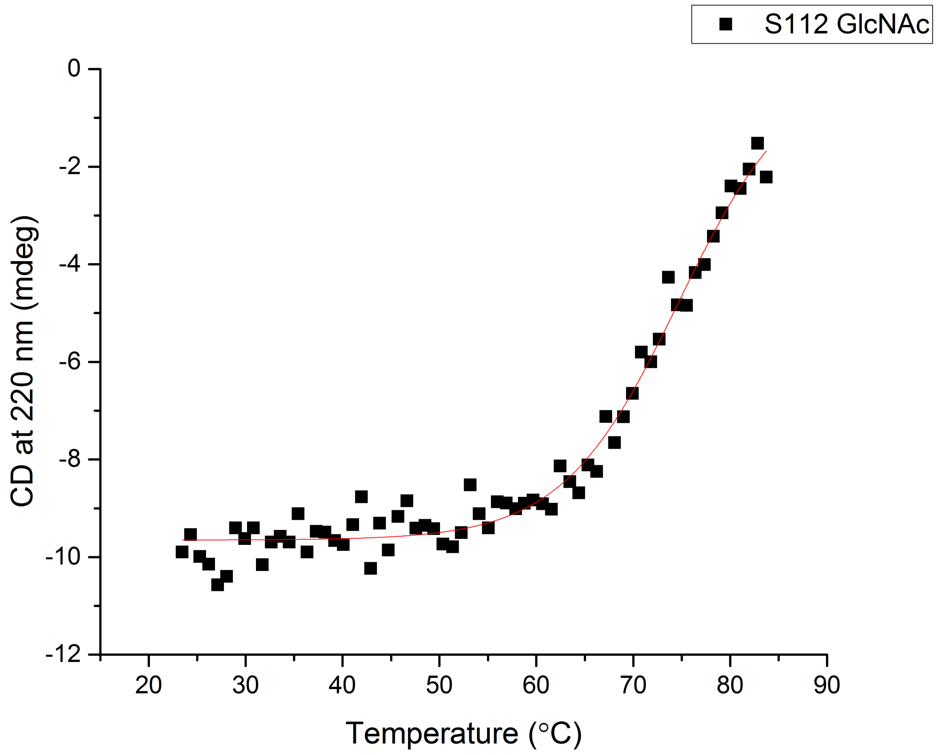

Figure S12. Melting profile of H2B S112 GlcNAc nucleosome. The melting temperature was extracted by monitoring the CD at 220 nm.

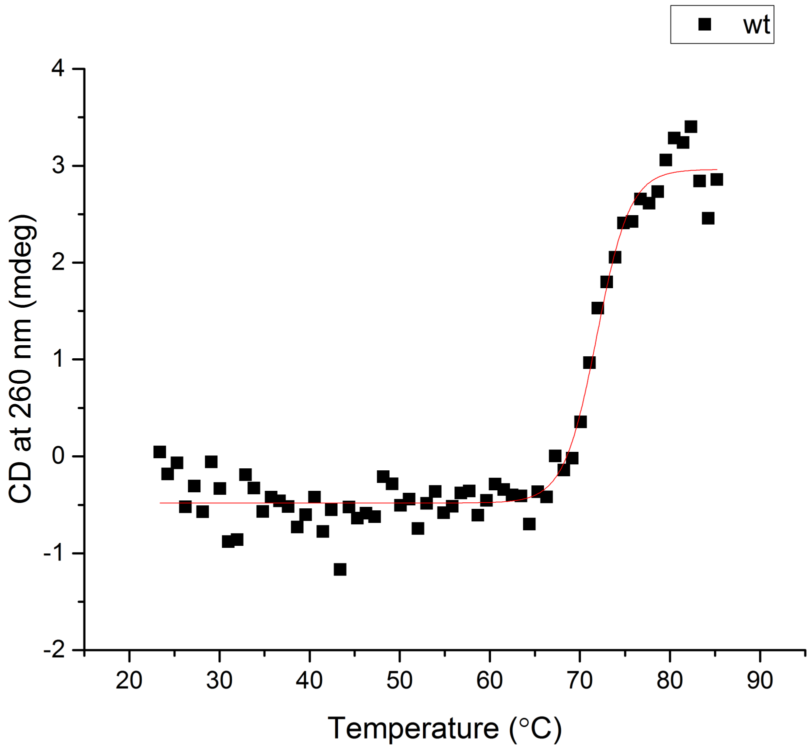

Figure S13. Melting profile of wt nucleosome. The melting temperature was extracted by monitoring the CD at 260 nm.

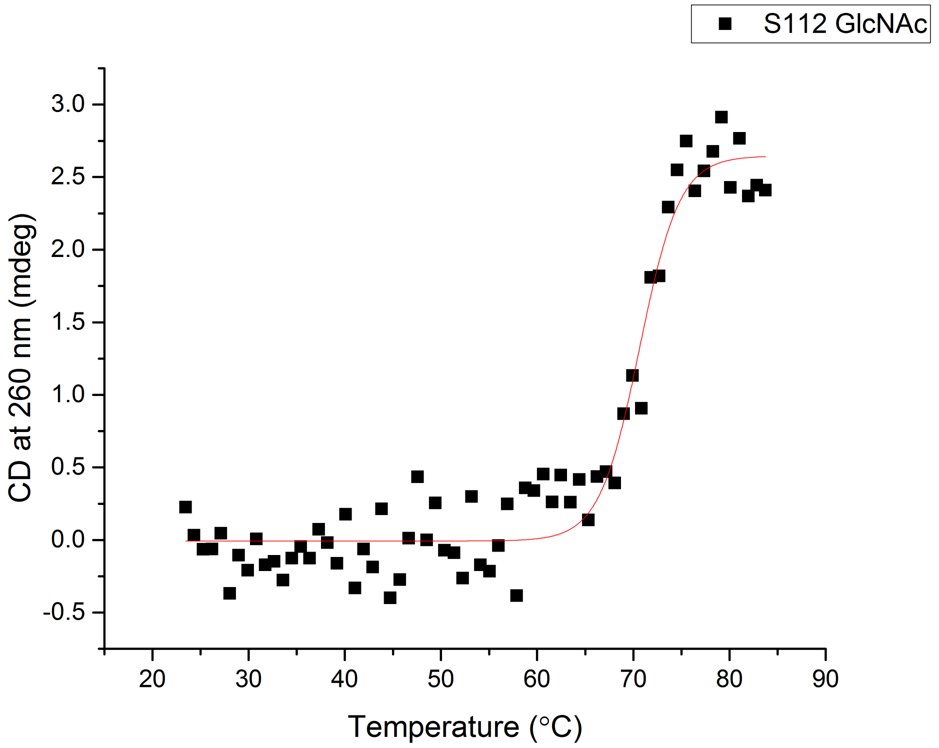

Figure S14. Melting profile of H2B S112 GlcNAc nucleosome. The melting temperature was extracted by monitoring the CD at 260 nm.

Nuclear extract preparation

HeLa cells were grown to near-confluency in a HYPERFlask™ cell culture vessel (Corning Life Sciences) containing 500 mL DMEM medium supplemented with 10 % FBS and 1 % Pen-Strep. Cells were trypsinized and were washed with 10 mL of ice-cold PBS buffer by centrifugation at 300g for 5 minutes at 4 °C.

The HeLa nuclear extract was prepared using an adapted protocol as previously described [http://www.cyclex.co.jp/resource/protocol/classical_biochemical/preparation_of_nuclear_extracts.pdf]. For nuclear extract preparation, following buffers were freshly prepared: (a) Lysis Buffer (10 mM Tris pH 7.5, 10 mM NaCl, 15 mM $MgCl_2$, 250 mM Sucrose, 0.1 mM EGTA, 0.5 % NP-40), (b) Sucrose Cushion (30 % Sucrose, 10 mM Tris pH 7.5, 10 mM NaCl, 3 mM $MgCl_2$), (c) Wash buffer (10 mM Tris pH 7.5, 10 mM NaCl) and (d) Extraction buffer (50 mM HEPES, 420 mM NaCl, 0.5 mM EDTA, 0.1 mM EGTA, 10 % Glycerol, 0.1 % NP-40). All buffers were supplemented with complete protease and phophatase inhibitors.

Briefly, Cells were re-suspended in 10 mL of lysis buffer and vortexed for 10 seconds. Cells were incubated on ice for 15 minutes followed by being spun through 40 mL of sucrose cushion at 1300g for 10 minutes at 4 °C. The supernatant was discarded. The isolated nuclei were washed with 10 mL of ice-cold wash buffer. Nuclei were re-suspended in 1 mL of extraction buffer and sonicated for 30 seconds. The ruptured nuclei were incubated on ice for 30 minutes followed by centrifugation at 13000g for 10 minutes at 4 °C. The supernatant, nuclear extract, was collected. The concentration of nuclear extract was checked using a BCA assay. The nuclear extract was diluted using a buffer such that final concentration was 0.5 mg/mL and buffer conditions were: 20 mM HEPES pH 7.9, 150 mM NaCl, 0.2 mM EDTA, 20 % glycerol, 0.1% NP40 with complete protease and phosphatase inhibitors (Binding Buffer). The nuclear extract aliquots were flash frozen in liquid nitrogen and stored at – 80 °C.

Interaction proteomics experiment

Interaction proteomics experiment was performed as previously described.[7,8] Briefly, 20 µg of each nucleosome were immobilised separately on 100 µL Dynabeads® Streptavidin MyOne™ T1 (Life Technologies) in the reconstitution buffer (10 mM Tris pH 7.5, 250 mM KCl, 1 mM EDTA, 1 mM DTT with 0.1 % NP40). The immobilisation efficiency was monitored by measuring the absorption value at 260 nm of the supernatant. Immobilised nucleosome were washed with the binding buffer (20 mM HEPES pH 7.9, 150 mM NaCl, 0.2 mM EDTA, 20% Glycerol, 0.1% NP40, 1 mM DTT supplemented with complete protease and phosphatase inhibitors) and then incubated with 1 mL of nuclear extract (0.5 mg/mL in binding buffer) for 4 hours at 4 °C. After 4 hours, non-specifically bound proteins were removed by washing with binding buffer (1 mL X 5 times). The interacting partners were eluted in 100 µL of 8 M Urea, 2 M NaCl.

Pooled interacting partners from each nucleosome sample were digested by Trypsin using the Filter-Aided Sample Preparation (FASP) protocol.[9] Digestion was quenched by addition of 1 % Formic Acid solution. Resulting tryptic peptides were analyzed on an EASY-nLC 1000 UHPLC system (Proxeon) connected to a Q Exactive mass spectrometer (Thermo Fischer Scientific) through an EASY-Spray nano-electrospray ion source (Thermo Fischer Scientific). The peptides were trapped on an in-house packed trap column (75 µm i.d. x 20 mm, reprosil C18, 3µm, 120 Å) using solvent A (0.1% Formic Acid, 5 % DMSO in water) at a pressure of 500 bar. The peptides were separated on an EASY-spray Acclaim PepMap® analytical column (75 µm i.d. × 500 mm, RSLC C18, 2 µm, 100 Å) using a linear gradient (length: 120 minutes, 8

% to 28 % solvent B (0.1% formic acid, 5 % DMSO in acetonitrile), flow rate: 200 nL/min). The separated peptides were electrosprayed directly into the mass spectrometer operating in a data-dependent mode. Full scan MS spectra were acquired in the Orbitrap (scan range 350-1500 m/z, resolution 70000, AGC target 3e6, maximum injection time 100 ms). After the MS scans, the 20 most intense peaks were selected for HCD fragmentation at 30 % of normalised collision energy. HCD spectra were also acquired in the Orbitrap (resolution 17500, AGC target 5e4, maximum injection time 120 ms).

The raw data files generated were processed using MaxQuant software (Version 1.4.3.17), integrated with Andromeda search engine as previously described.[10,11] Raw MS data were searched against human database (Swiss-Prot, version 04/13) as well as list of common contaminants by Andromeda. Trypsin with a maximum number of 2 missed cleavages was selected as the protease. Acetylation (Protein N-term) and Oxidation (Methionine) were used as variable modifications while Carbamidomethylation (Cysteine) was set as a fixed modification. Protein and PSM false discovery rate (FDR) were set at 0.01. Label free quantification (LFQ) was performed using the in-built algorithm in the MaxQuant.[12] Further downstream data analysis was performed using Perseus software (Version 1.4.2.27). LFQ intensity of all identified protein groups was uploaded; reverse and protein groups only indentified by site were removed. Protein groups identified in all three replicate experiments of either bait or control samples were used. LFQ intensities were logaritmized and missing LFQ intensities were imputed. False discovery rate (FDR) based two-sample t-test was used to generate fold-change difference as well as a *p* value. Protein groups with corresponding *p* value lower than 0.05 were considered as significant. The data was visualised in a volcano plot of ratio change versus log (*p* value). See Figures S15,16 and Supplementary Data Tables 1,2,3.

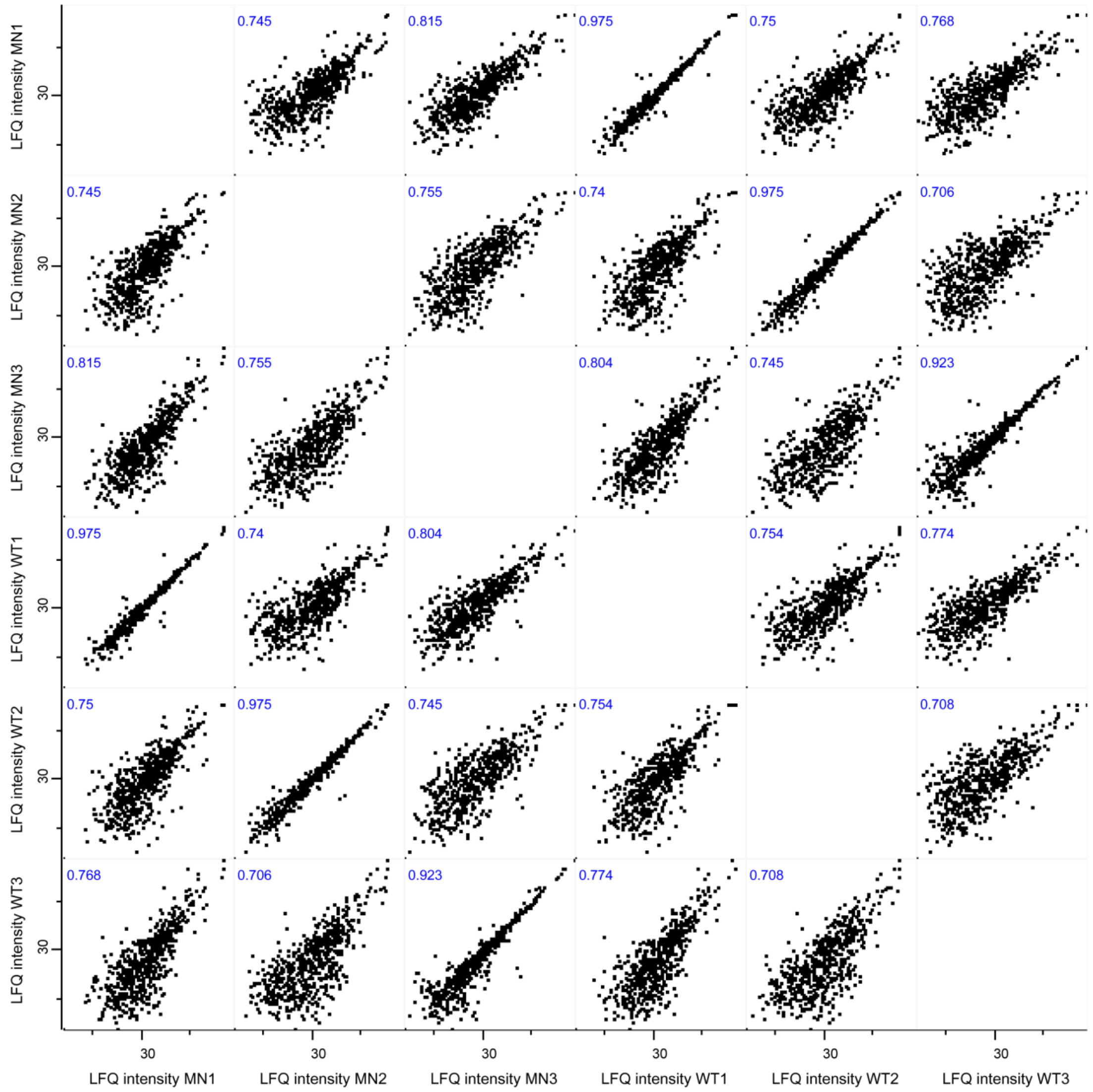

Figure S15. Reproducibility of LFQ intensities within group and across replicates with corresponding Pearson correlation coefficients.

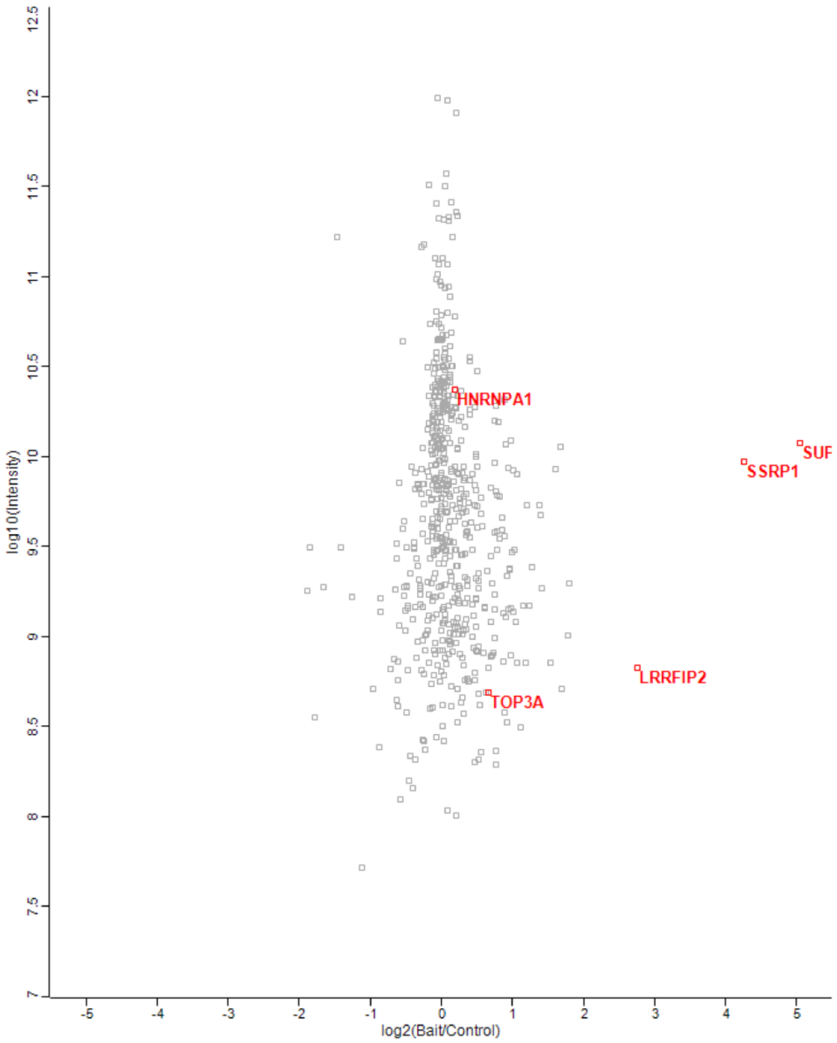

Figure S16. Scatter plot for the interacting protein partners for H2B-S112-GlcNAcylated nucleosome (bait) over wild-type nucleosome (control) among 584 identified proteins. x axis, logarithmized ratio of average LFQ intensities of protein groups in the bait over control pulldown experiment; y axis, log10-transformed summed up intensity of protein groups. The log fold change for most significant interactors, SUPT16H and SSRP1 are much higher than the variance for the non-interacting protein partners.

Western blot against anti-BRE1A antibody

8 µL aliquots from nuclear extract (as positive control), interacting partners from control experiment and interacting partners from bait experiment were subjected to SDS-PAGE analysis. Pre-stained protein standards were also added. The SDS-PAGE analysis was performed using 4-12 % Bis-Tris gel (Novex). The gel was run in 1x MOPS buffer at 200 V for 50 minutes. The proteins were transferred to PVDF membrane using iBlot dry blotting

system (Life Technologies). The membrane was blocked using 50 mL 5 % BSA solution in 1x TBST buffer by gentle rocking at room temperature for 1 hour. After blocking, the membrane was washed with TBST buffer (2 X 30 mL, 5 minutes each). The membrane was probed by primary antibody against BRE1A (Anti-RNF20 antibody, ab181032 (Abcam [EPR13563(B)])) at 1: 10000 dilution in 50 mL of 5 % BSA solution in TBST buffer by gentle rocking at room temperature for 1 hour. The membrane was extensively washed by TBST buffer (6 X 40 mL, 10 minutes each). The membrane was probed by secondary antibody (Goat anti-rabbit antibody conjugated with HRP, Bio-Rad) at 1: 10000 dilution in 50 mL of 5 % BSA solution in TBST buffer by gentle rocking at room temperature for 1 hour. After that, the membrane was extensively washed by TBST buffer (6 X 40 mL, 10 minutes each). The membrane was then developed using 1 mL of Clarity™ Western ECL substrate (Bio-Rad). Excess of ECL reagent was removed and the blot was developed on X-ray film as shown in Supplementary Figure S17.

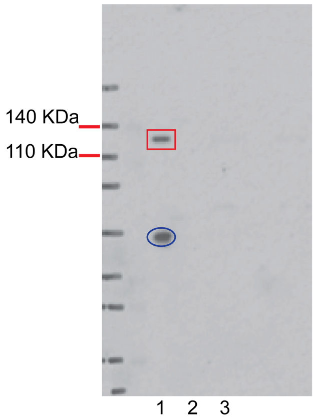

Figure S17. Western blot analysis against anti-BRE1A antibody. Lanes are as follows: 1: Nuclear extract as positive control, 2: Interacting protein partners from control experiment, 3: Interacting protein partners from bait experiment. The red box denotes BRE1A antibody detected in positive control; blue oval indicates antibody cross-reactivity.

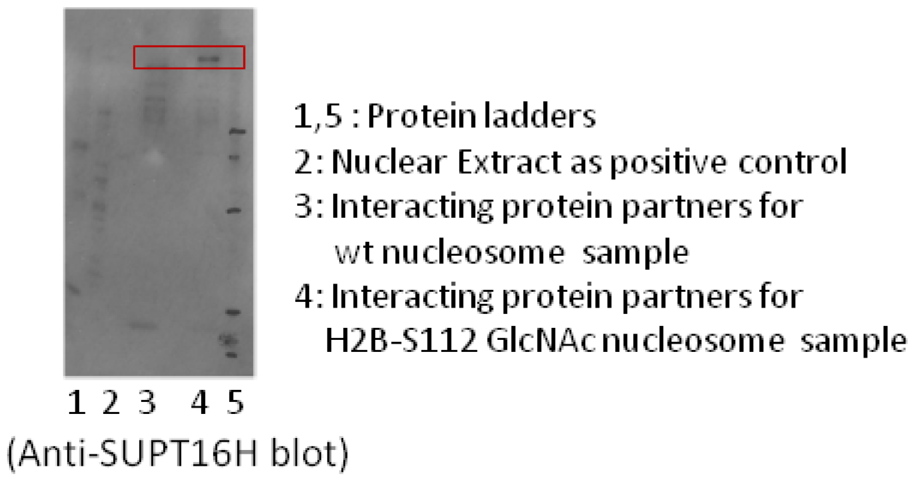

Figure S18. Western blot analysis against anti-SUPT16H antibody (ab108960, [EPR3685], Abcam). Lanes are as follows: 1,5: Protein ladders, 2: Nuclear extract as positive control, 3: Interacting protein partners from control experiment (wt nucleosome), 4: Interacting protein partners from bait experiment (H2B S112 GlcNAc nucleosome).

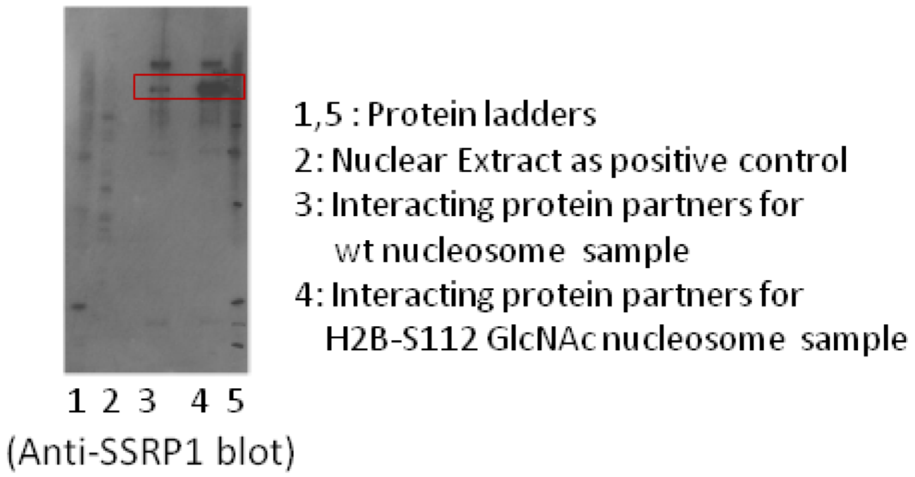

Figure S19. Western blot analysis against anti-SSRP1 antibody (ab129109, [EPR7894], Abcam). Lanes are as follows: 1,5: Protein ladders, 2: Nuclear extract as positive control, 3: Interacting protein partners from control experiment (wt nucleosome), 4: Interacting protein partners from bait experiment (H2B S112 GlcNAc nucleosome).

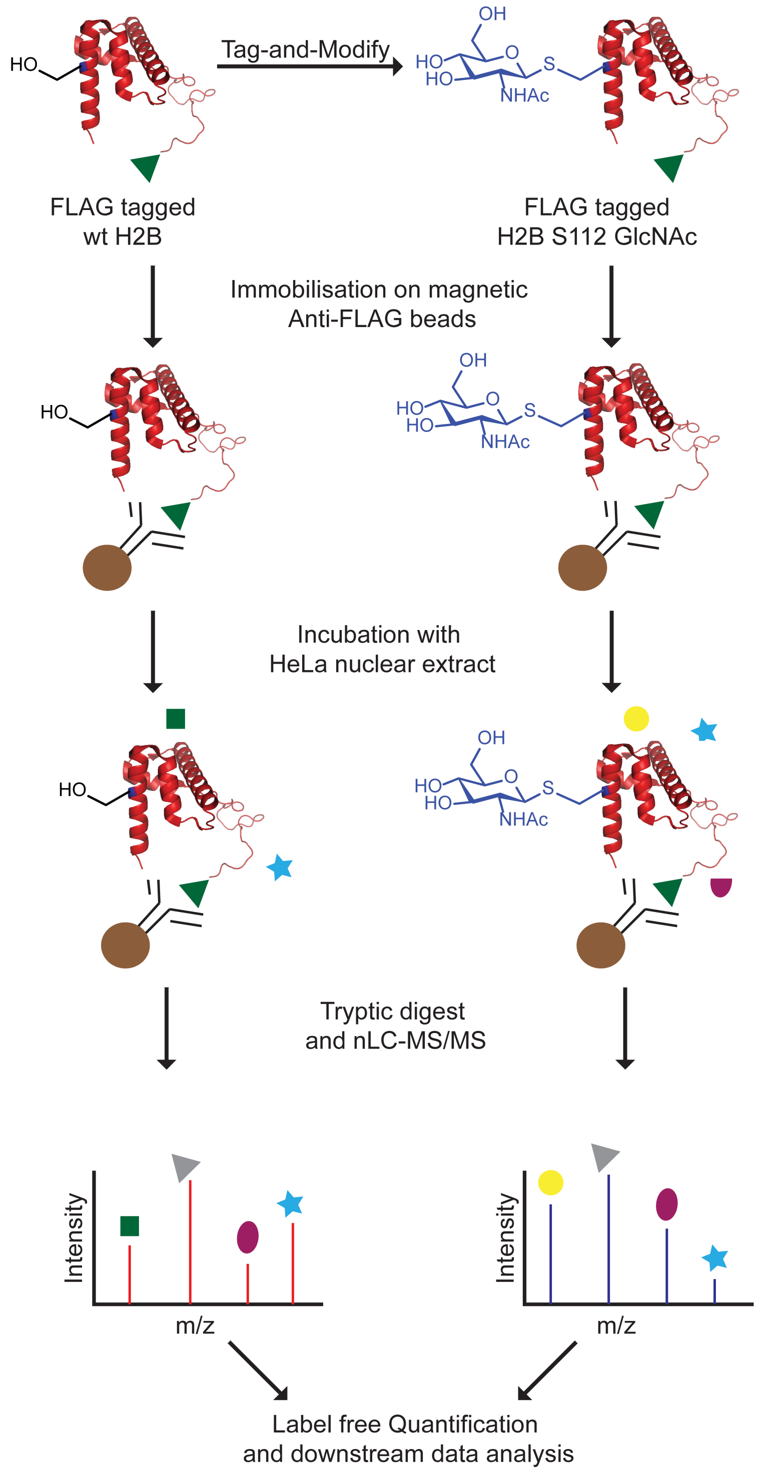

Figure S20. The schematic for identification of interacting protein partners for GlcNAcylated H2B-Ser112 protein. Wt and modified histone H2B protein bearing N-terminal FLAG tag were immobilized on magnetic Anti-FLAG beads to affinity enrich interacting partners. Pooled proteins from each sample were analyzed down-stream similarly as for the nucleosome samples.

Expression and purification of FLAG tagged Histone H2B proteins

FLAG tag was introduced in wt and H2B S112C plasmid using following primers by standard molecular biology techniques:

Forward Primer: acgtac CATATG GACTACAAAGACGACGACGACAAA GGC GCC AAG TCC GCT CCA GCC

Reverse primer: acgtac GGATCC TTA CTT GGC GCT GGT GTA CTT GGT G

The introduction of FLAG tag was verified by sequencing. The proteins were expressed and purified as described earlier for histone proteins.

Site-selective chemical protein modification

Site-selective GlcNAc mimic was installed on FLAG tagged H2B-S112C (FLAG-H2B-S112C) protein in a similar two-step reaction as for H2B-S112C protein.

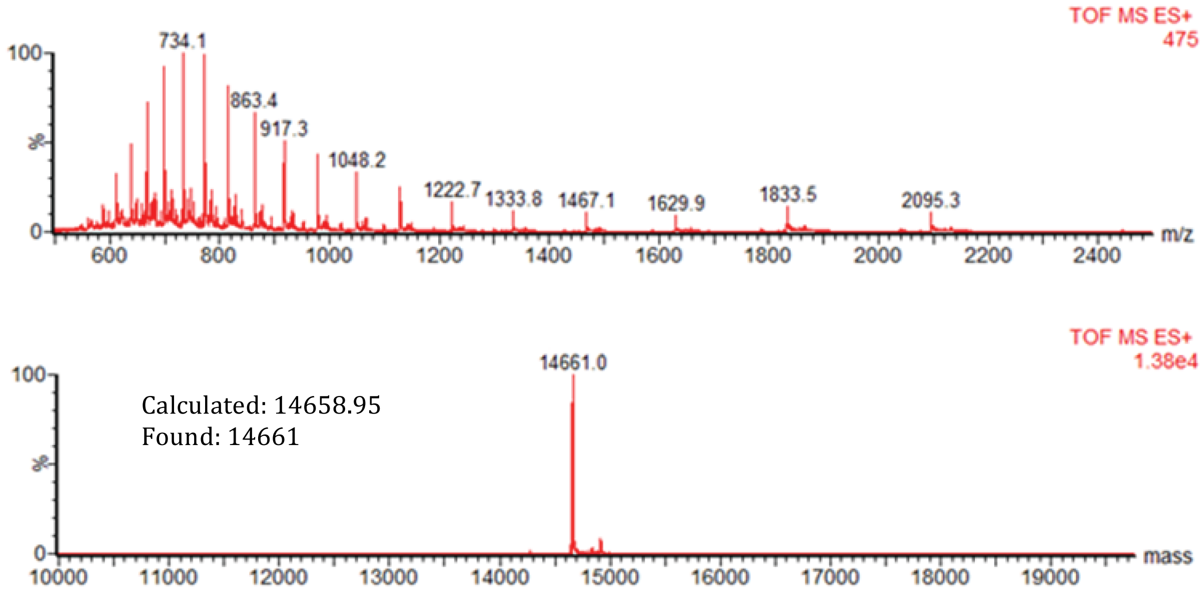

Figure S21. LC-MS Spectrum of FLAG tagged H2B S112Dha

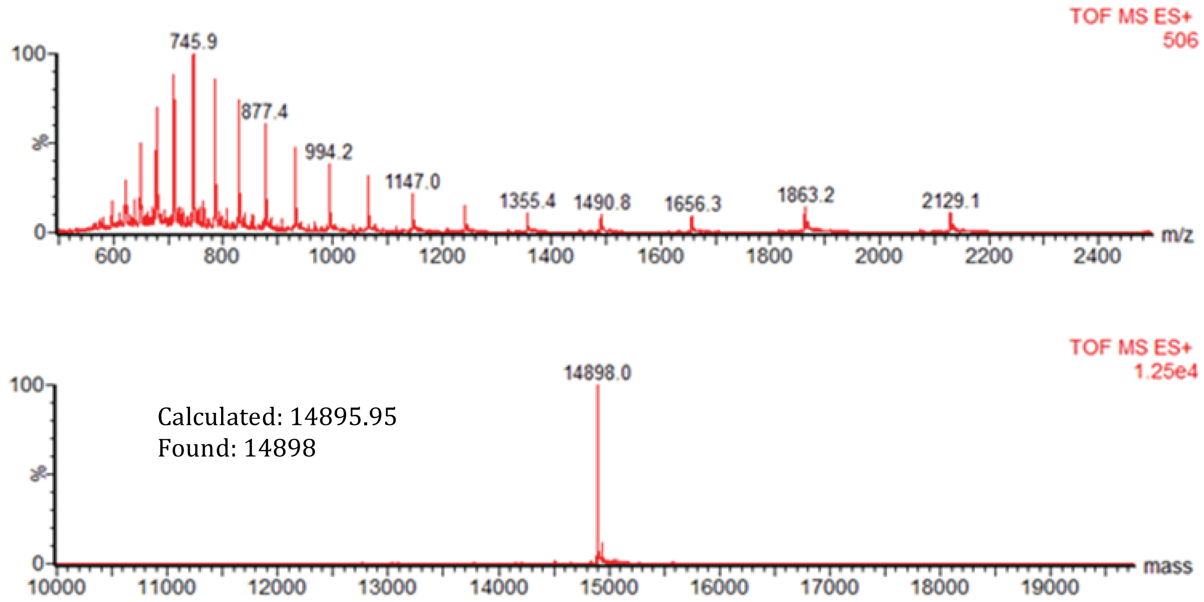

Figure S22. LC-MS Spectrum of FLAG tagged H2B S112GlcNAc

CD analysis of the FLAG tagged H2B monomers

FLAG tagged Wild-type H2B protein and FLAG tagged H2B-S112 GlcNAc protein were buffer exchanged into 10 mM Tris pH 7.5, 250 mM KCl, 1 mM EDTA by dialysis. CD analysis was performed as earlier for the H2B monomers.

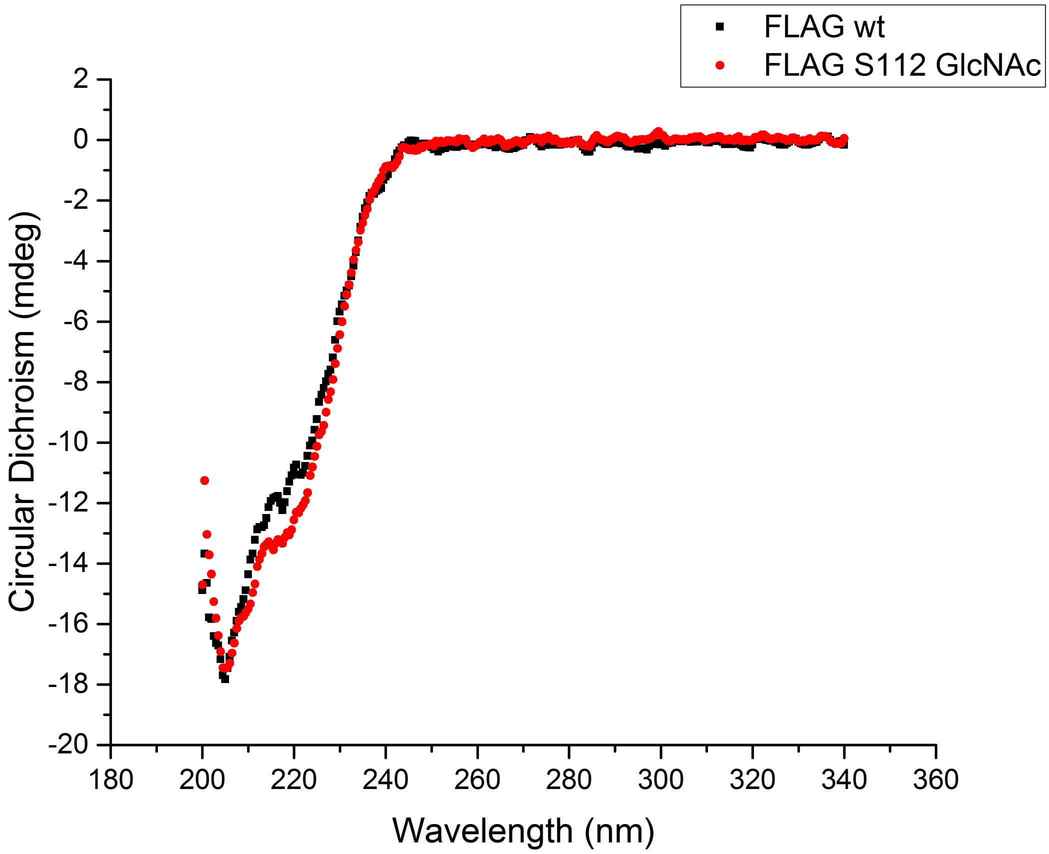

Figure S23. CD analysis of refolded FLAG tagged wt H2B monomer (black) and H2B S112 GlcNAc monomer (red).

Quantitative MS experiment with FLAG tagged H2B S112 GlcNAc monomer and dimer

Nuclear Extract Preparation

Nuclear extract was prepared under non-reducing conditions without the use of DTT following earlier mentioned protocol.

Immobilization on the magnetic Anti-FLAG beads and interaction studies with nuclear extract

20 µg of each monomer/dimer were immobilised separately on 70 µL of Anti-FLAG® M2 magnetic beads (M8823, Sigma-Aldrich) in the reconstitution buffer (10 mM Tris pH 7.5, 20 mM KCl, 1 mM EDTA with 0.1 % NP40). The immobilisation efficiency was monitored by measuring the absorption value at 280 nm of the supernatant. Immobilised protein was

washed with the binding buffer (20 mM HEPES pH 7.9, 150 mM NaCl, 0.2 mM EDTA, 20% Glycerol, 0.1% NP40 with complete protease and phosphatase inhibitors) and then incubated with 1 mL of nuclear extract (0.5 mg/mL in binding buffer) for 4 hours at 4 °C. After stringent washes with binding buffer, the beads were incubated with 250 µL of IgG elution buffer (21004, ThermoFisher Scientific) for 5 minutes at room temperature with gentle rotation. The supernatant was collected in an eppendorf tube containing 50 µL of 1 M Tris pH 9.0 buffer solution. The elution step was repeated once more.

Proteolytic Digestion and nLC-MS/MS Analysis

Tryptic digestion and nLC-MS/MS analysis for the interacting protein partners were performed in a similar way as for interacting protein partners from nucleosome samples.

Data Analysis

The data analysis was performed in a similar way as that for nucleosome samples. The results for interaction study with monomer were visualized by uploading the LFQ intensities into Perseus software (Version 1.4.2.27). Contaminant, reverse and protein groups only identified by site were filtered off. Protein groups identified in at least one of bait and control samples in either replicate were used. Their LFQ intensities were logarithmized and missing LFQ intensities were imputed. Mean intensity value was calculated. The difference between the average LFQ intensities of protein groups between bait and control samples alongside total intensity were evaluated by Significance B using Benjamini-Hochberg FDR (threshold value = 0.05) in order to generate a p-value.

The LFQ intensities of the interacting protein partner for dimer pulldown were uploaded into Perseus. Contaminant, reverse and protein groups only indentified by site were filtered off. Only protein groups identified in each sample were used. The LFQ intensities were logarithmized and a difference between bait and control sample was calculated. The difference between the intensities was plotted against total intensity. Significance B analysis was done in order to generate a p-value.

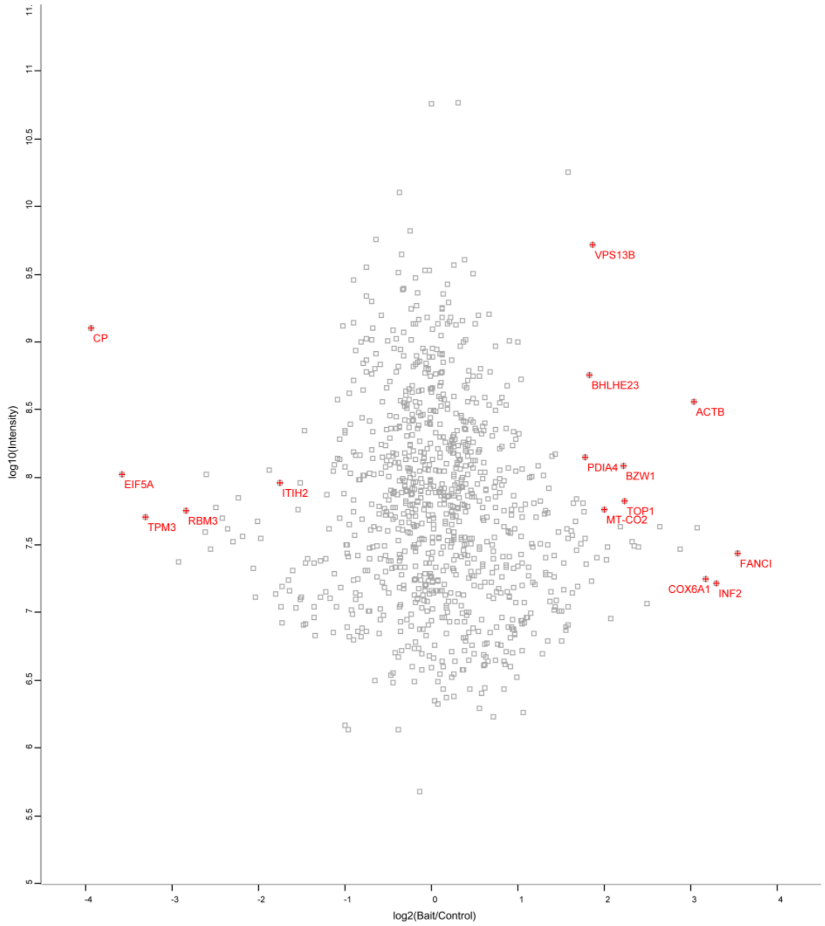

Figure S24. Preferential Interacting protein partners for FLAG tagged H2B S112 GlcNAc monomer (bait) over wild-type FLAG tagged H2B (control) among 948 identified proteins identified in two biological replicates. Top 15 significant interacting protein partners (denoted by gene name) identified by Label free quantification (LFQ) on Significance B analysis are shown in red in a scatter plot. Scatter plot: x axis, logarithmized ratio of average LFQ intensities of protein groups in the bait over control pulldown experiment from two replicates; y axis, log10-transformed summed up intensity of protein groups.

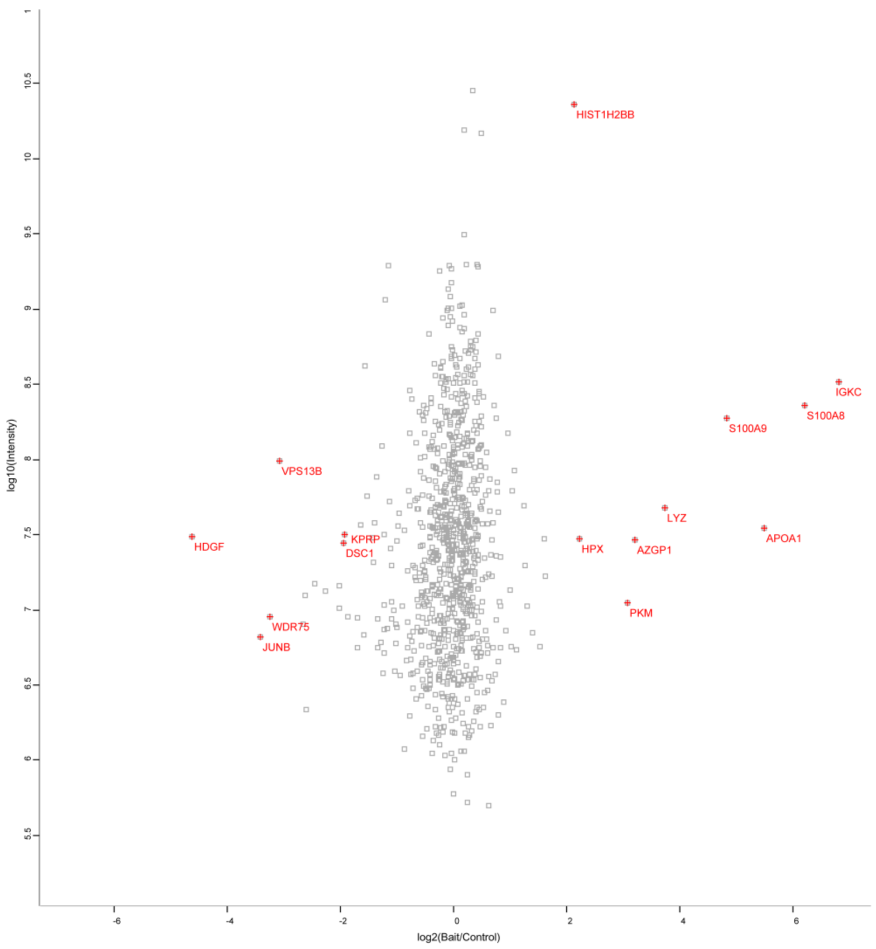

Figure S25. Preferential Interacting protein partners for FLAG tagged H2A/B S112 GlcNAc dimer (bait) over wild-type FLAG tagged H2A/B dimer (control) among 948 identified proteins identified in a single experiment. Top 15 significant interacting protein partners (denoted by gene name) identified by Label free quantification (LFQ) on Significance B analysis are shown in red in a scatter plot. Scatter plot: x axis, logarithmized ratio of average LFQ intensities of protein groups in the bait over control pulldown experiment; y axis, log10-transformed summed up intensity of protein groups.

References:

1. Dyer, P. N.; Edayathumangalam, R. S.; White, C. L.; Bao, Y.; Chakravarthy, S.; Muthurajan, U. M.; Luger, K. in *Methods in Enzymology*, eds. C. D. Allis and W. Carl, Academic Press, **2003**, pp. 23-44.

2. Chalker, J. M.; Lercher, L.; Rose, N. R.; Schofield, C. J.; Davis, B. G. *Angew Chem Int Ed*, **2012**, *51*, 1835.

3. Singh, C.; Zampronio, C. G.; Creese, A. J.; Cooper, H. J. *J Proteome Res*, **2012**, *11*, 4517.

4. Saba, J.; Dutta, S.; Hemenway, E.; Viner, R. *Int J Proteomics* **2012**, *2012*, 560391.

5. Luger, K.; Rechsteiner, T. J.; Richmond, T. J. In *Chromatin Protocols*; Becker, P. B., Ed.; Humana Press: 1999; Vol. 119, p 1.

6. Luger, K.; Rechsteiner, T. J.; Richmond, T. J. In *Methods in Enzymology*; Paul M. Wassarman, A. P. W., Ed.; Academic Press: 1999; Vol. Volume 304, p 3.

7. L. Lercher, R. Raj, N. A. Patel, J. Price, S. Mohammed, C. V. Robinson, C. J. Schofield, B. G. Davis, *Nat Commun* **2015**, 6, 7978, doi:8910.1038/ncomms8978

8. Bartke, T.; Vermeulen, M.; Xhemalce, B.; Robson, S. C.; Mann, M.; Kouzarides, T. *Cell*, **2010**, *143*, 470.

9. Wisniewski, J. R.; Zougman, A.; Nagaraj, N.; Mann, M. *Nat. Methods*, **2009**, *6*, 359.

10. Cox, J.; Mann, M. *Nat Biotechnol*, **2008**, *26*, 1367.

11. Cox, J.; Neuhauser, N.; Michalski, A.; Scheltema, R. A.; Olsen J. V.; Mann, M. *J Proteome Res*, **2011**, *10*, 1794.

12. Cox, J.; Hein, M. Y.; Luber, C. A.; Paron, I.; Nagaraj, N.; Mann, M. *Mol Cell Proteomics*, **2014**, *13*, 2513.

Supplementary table 1							
**Identification and quantification of interacting protein groups for bait (H2B S112 GlcNAc) versus control (wt) samples. Statistically significant interacting partners (*p* value < 0.05) are denoted with a '+' in column F**							
**Majority Protein Ids**	**Protein names**	**Gene names**	**log2(Bait/Control)**	**log10(*p* value)**	**Statistically Significant Interactor**	**PEP**	**MS/MS count**
Q9Y5B9	FACT complex subunit SPT1	SUPT16H	5.038078944	3.061507507	+	0	153
Q08945	FACT complex subunit SSRP	SSRP1	4.251744588	2.968569012	+	0	103
P09651;P09651-3;F8VXY0;F8'	Heterogeneous nuclear ribc	HNRNPA1;HNRNPA:	0.198673248	2.09455328	+	0	180
Q9Y608-2;Q9Y608-4;Q9Y608;	Leucine-rich repeat flightles	LRRFIP2	2.689150492	1.974461635	+	3.96E-136	15
Q13472;B4DK80;Q13472-2;B	DNA topoisomerase 3-alpha	TOP3A	0.659311295	1.360955022	+	8.27E-46	18
Q96HA7;Q96HA7-2	Tonsoku-like protein	TONSL	-2.148585002	0.813891982		0.0025564	6
F5GWP8		JUP	-1.249556859	0.740439051		3.71E-183	26
Q96E14;I3L2E0	RecQ-mediated genome ins	RMI2	0.985944748	0.720380047		8.14E-39	3
Q13151	Heterogeneous nuclear ribc	HNRNPA0	0.362734477	0.701840918		6.96E-102	50
O14974-3;O14974-4;F8VZN8	Protein phosphatase 1 regu	PPP1R12A	-1.294486364	0.674819984		0	4
P48668;CON__P48668	Keratin, type II cytoskeletal	KRT6C	-1.409152985	0.642325754		1.12E-304	53
Q15637-6;Q15637-2;Q15637	Splicing factor 1	SF1	0.64846611	0.640353254		5.76E-72	31
P53999	Activated RNA polymerase I	SUB1	1.380776087	0.631082405		2.34E-14	7
Q9ULW0;Q96RR5	Targeting protein for Xklp2	TPX2;HCA90	1.795644124	0.620288916		2.16E-141	46
P35251;P35251-2	Replication factor C subunit	RFC1	0.881896973	0.611342878		0	180
Q15427	Splicing factor 3B subunit 4	SF3B4	0.337552389	0.608670209		3.69E-80	18
P27694	Replication protein A 70 kD	RPA1	2.389371872	0.593162448		2.65E-233	28
P31943;G8JLB6;E9PCY7;D6RI	Heterogeneous nuclear ribc	HNRNPH1	0.330139796	0.592388256		0	81
P35659;P35659-2;B4DFG0;D(	Protein DEK	DEK	-1.881011963	0.589908866		2.67E-82	28
Q6I9Y2	THO complex subunit 7 hon	THOC7	0.29744784	0.584337699		1.81E-131	15
P38919	Eukaryotic initiation factor	EIF4A3	0.699170431	0.545048404		1.33E-118	49
Q8IUE6	Histone H2A type 2-B	HIST2H2AB	-1.474319458	0.543799059		5.00E-138	587
M0QZM1		HNRNPM	-0.352521261	0.539989717		2.10E-161	7
P08779;CON__P08779	Keratin, type I cytoskeletal :	KRT16	-1.842549006	0.530661051		0	62
Q9Y2S0	DNA-directed RNA polymer	POLR1D	0.535025915	0.488518186		2.71E-64	8
P41208	Centrin-2	CETN2	0.721959432	0.487406039		2.10E-125	11
P02533;CON__P02533;CON_	Keratin, type I cytoskeletal :	KRT14	-1.000313441	0.481790332		1.02E-212	17
Q9BQ04;E9PLB0;E9PM61	RNA-binding protein 4B	RBM4B	0.459698995	0.477326723		7.36E-89	11
P62841;K7EM56;K7ELC2;K7E	40S ribosomal protein S15	RPS15	1.222133001	0.46908449		3.62E-23	52
P62805	Histone H4	HIST1H4A	0.21070226	0.452649414		0	1742
P35249;C9JZI1;C9J8M3;C9JT	Replication factor C subunit	RFC4	1.614082972	0.447245625		0	85
Q9NUW8;Q86TV8;G3V2F4;E	Tyrosyl-DNA phosphodieste	TDP1	0.520119985	0.419889768		1.23E-17	9
P35250;P35250-2;H7C5P4	Replication factor C subunit	RFC2	1.011479696	0.418357593		0	104
Q9UII2	ATPase inhibitor, mitochon	ATPIF1	-0.590906143	0.407960103		1.10E-06	15
Q96KR1	Zinc finger RNA-binding pro	ZFR	0.254386902	0.404218832		0	83
Q9NUK0;Q9NUK0-2;B1AKI6	Muscleblind-like protein 3	MBNL3	-1.111483256	0.397996501		1.81E-18	12
Q9UNL2;C9JA28;B4E2P2	Translocon-associated prot	SSR3	1.639628092	0.394513594		6.14E-15	10
P62306;F8W0W6	Small nuclear ribonucleopr	SNRPF	-0.231740316	0.372402552		5.30E-22	11
Q5M775;Q5M775-4;Q5M775	Cytospin-B	SPECC1	0.129275004	0.364275713		2.23E-269	53

P35580;P35580-2;F8W6L6;P3	Myosin-10	MYH10	-0.507511139	0.357291105	0	56
P50402;Q5HY57	Emerin	EMD	0.484237671	0.351980254	7.41E-160	33
Q12904;Q12904-2	Aminoacyl tRNA synthase co	AIMP1	0.882725398	0.348019465	2.96E-196	43
P40938;P40938-2	Replication factor C subunit	RFC3	1.682502111	0.347867729	0	118
Q14011;K7EMY9;K7EPM4;K7	Cold-inducible RNA-binding	CIRBP	1.312144597	0.343152371	6.08E-60	21
P05787;CON__P05787;P0578	Keratin, type II cytoskeletal	KRT8	0.096977234	0.342957126	0	1035
Q9ULV0	Unconventional myosin-Vb	MYO5B	0.972047806	0.342476917	7.19E-35	15
P40937;P40937-2;F8W9B4	Replication factor C subunit	RFC5	0.949289958	0.340766737	0	90
Q86XZ4	Spermatogenesis-associated	SPATS2	1.166576385	0.335598594	3.52E-149	19
P15328	Folate receptor alpha	FOLR1	-0.231505712	0.334229184	9.69E-101	22
P13647;CON__P13647;F8W0	Keratin, type II cytoskeletal	KRT5	-0.664825439	0.33262993	9.40E-243	21
Q9UKF6;G5E9W3	Cleavage and polyadenylati	CPSF3	1.385275523	0.330879965	1.14E-161	42
P63173;J3KT73;J3QL01;J3KSP	60S ribosomal protein L38	RPL38	0.334237417	0.322421622	2.84E-16	51
P56182	Ribosomal RNA processing p	RRP1	0.910595576	0.320776341	4.06E-21	16
P62873;B3KVK2	Guanine nucleotide-binding	GNB1	0.700613022	0.318089115	1.06E-73	12
Q9H307;Q9H307-2	Pinin	PNN	0.495153427	0.314424009	6.64E-124	37
Q9P2J5;F5H698;B4DJ10;B4DE	Leucine--tRNA ligase, cytopl	LARS	1.438474655	0.312061811	8.53E-70	31
G5E9M5;Q12906-4;Q12906-6	Interleukin enhancer-bindin	ILF3	-0.114262899	0.305783365	0	142
P47755;F8W9N7	F-actin-capping protein sub	CAPZA2	0.711062749	0.293689324	6.83E-90	19
P08729;CON__Q3KNV1;F8VZ	Keratin, type II cytoskeletal	KRT7	0.272989909	0.282669348	0	14
Q9HC52	Chromobox protein homolo	CBX8	1.167825063	0.280525126	6.32E-60	23
Q9P035;B4DRF4;H3BS72;H3B	Very-long-chain (3R)-3-hydr	PTPLAD1	0.910380046	0.277353553	6.65E-85	17
P62273;P62273-2	40S ribosomal protein S29	RPS29	-0.497578939	0.276189419	6.06E-20	15
Q9Y3B4	Pre-mRNA branch site prote	SF3B14	0.947014491	0.274286213	1.19E-22	19
Q86XK2;Q86XK2-5;Q86XK2-6	F-box only protein 11	FBXO11	0.86783282	0.273025699	1.88E-83	27
Q9H7E2;Q9H7E2-3;Q9H7E2-2	Tudor domain-containing pr	TDRD3	0.430744171	0.272482783	6.19E-59	27
P17480;P17480-2;E9PKP7	Nucleolar transcription fact	UBTF	0.333123525	0.267464829	0	100
Q12926;Q12926-2;B1AM49;E	ELAV-like protein 2;ELAV-lik	ELAVL2;ELAVL4	0.702601751	0.267226027	4.18E-91	17
Q96CW1;Q96CW1-2;E9PFW3	AP-2 complex subunit mu	AP2M1	-1.218945821	0.266732592	2.87E-114	37
P16403;P16402	Histone H1.2;Histone H1.3	HIST1H1C;HIST1H1D	-0.284726461	0.266580802	1.69E-38	136
Q15287;H3BV80;Q15287-3;Q	RNA-binding protein with se	RNPS1	0.120931625	0.26435906	1.50E-30	19
Q96DI7;B4DQJ1	U5 small nuclear ribonucleo	SNRNP40	1.575176875	0.26340872	5.51E-252	47
P51114;B4DXZ6;P51114-2;P5	Fragile X mental retardation	FXR1	0.085731506	0.26159061	0	237
Q13045;J3QLG3;Q13045-2	Protein flightless-1 homolog	FLII	-0.480203629	0.261370147	1.62E-299	55
P27105;B4E2V5	Erythrocyte band 7 integral	STOM	0.745147069	0.260115799	1.38E-50	19
Q6P5R6;C9JYQ9;H0Y8C2	60S ribosomal protein L22-l	RPL22L1	0.311777751	0.256257337	8.24E-107	8
Q9NYL9;H0YNJ8	Tropomodulin-3	TMOD3	0.13660876	0.255274788	0	74
Q6NZI2;Q6NZI2-2	Polymerase I and transcript	PTRF	-0.849328359	0.255220508	3.42E-74	24
P62847;P62847-2;E7ETK0;P6	40S ribosomal protein S24	RPS24	0.976221085	0.254890641	3.63E-17	63
P42167;G5E972;P42167-2;H0	Lamina-associated polypept	TMPO	0.213640849	0.250682074	1.36E-180	64
P49207	60S ribosomal protein L34	RPL34	0.473462423	0.248722194	3.02E-13	27
Q12800;F8VX55;Q12800-3;Q	Alpha-globin transcription f	TFCP2	0.97723643	0.24842123	2.80E-231	26
Q14134;Q14134-2;B7Z8U9;E9	Tripartite motif-containing p	TRIM29	-0.486513138	0.245107986	2.88E-28	13
Q9Y4P3;E9PF19	Transducin beta-like protein	TBL2	-0.329182307	0.2406	3.43E-238	80
P60468	Protein transport protein Se	SEC61B	-0.666510264	0.240323845	2.15E-05	6
Q9BWJ5	Splicing factor 3B subunit 5	SF3B5	-0.191724141	0.238121442	2.49E-14	6

Q9NRC1;B7Z4U3;Q9NRC1-4;E	Suppressor of tumorigenicit	ST7;ST7L	-0.646879196	0.237425658	1.23E-06	11
P18077;C9K025;F8WBS5;F8W	60S ribosomal protein L35a	RPL35A	0.35761706	0.236900686	1.18E-15	55
O75531	Barrier-to-autointegration f	BANF1	0.28351593	0.232152398	3.55E-18	12
P61513;C9J4Z3;Q6P4E4;E9PE	60S ribosomal protein L37a	RPL37A	0.315478007	0.232067988	2.14E-36	44
Q7Z2E3;Q7Z2E3-7;Q7Z2E3-8;	Aprataxin	APTX	-0.134639104	0.231204974	9.91E-145	77
P61619;B4DR61;P61619-3	Protein transport protein Se	SEC61A1	-0.464300791	0.229743817	1.39E-28	23
Q9Y3A4	Ribosomal RNA-processing	RRP7A	0.750528971	0.22677029	8.88E-76	35
Q96HS1;Q96HS1-2;F5GXG4	Serine/threonine-protein pl	PGAM5	0.788026174	0.226390393	5.55E-137	46
Q71U36;G3V1U9;Q9BQE3;F5	Tubulin alpha-1A chain;Tub	TUBA1A;TUBA1C;TU	1.583175659	0.223523119	3.86E-299	76
Q01844;Q01844-6;B0QYK0;Q	RNA-binding protein EWS	EWSR1	-0.082613627	0.223279646	1.35E-177	94
Q86U38;A8MY76	Nucleolar protein 9	NOP9	-0.285728455	0.220283318	4.49E-115	10
Q8N3X1;Q8N3X1-2	Formin-binding protein 4	FNBP4	0.361172994	0.220226401	0	42
Q9UHB7;Q9UHB7-2	AF4/FMR2 family member 4	AFF4	0.87548701	0.220058374	6.37E-76	18
O43823	A-kinase anchor protein 8	AKAP8	-0.357156118	0.218005793	2.40E-150	41
Q8N684;Q8N684-2;Q8N684-3	Cleavage and polyadenylati	CPSF7	0.720559438	0.216646566	2.68E-116	32
O95425;O95425-2;F5H2Q5	Supervillin	SVIL	0.318286896	0.21502921	2.14E-171	40
Q12905	Interleukin enhancer-bindin	ILF2	0.49299113	0.214753924	0	107
Q5BKZ1;Q5BKZ1-3	DBIRD complex subunit ZNF	ZNF326	0.743563334	0.211638274	7.49E-69	25
Q9Y3T9	Nucleolar complex protein 2	NOC2L	0.751158396	0.211458966	1.05E-45	29
Q29RF7	Sister chromatid cohesion p	PDS5A	0.492919922	0.209806009	3.95E-195	38
P14866;M0QXS5;P14866-2	Heterogeneous nuclear ribo	HNRNPL	0.503950119	0.208714954	0	118
P55265;P55265-5;P55265-4;E	Double-stranded RNA-speci	ADAR	1.533538183	0.208155294	0	70
Q9H583;Q5T3Q7	HEAT repeat-containing pro	HEATR1	0.880447388	0.20745311	6.04E-150	45
Q13148;Q13148-2;B1AKP7;G	TAR DNA-binding protein 43	TARDBP	0.356906255	0.206529765	7.28E-70	20
P16401	Histone H1.5	HIST1H1B	-0.532803853	0.20620755	1.39E-40	69
P62854;Q5JNZ5;F8VZW7	40S ribosomal protein S26;F	RPS26;RPS26P11	0.796175639	0.204174215	1.93E-50	54
P38159;P38159-2;H0Y6E7;H3	RNA-binding motif protein,	RBMX;RBMXL1	-0.076836904	0.201628903	1.24E-180	44
Q9P0K7;Q9P0K7-4;Q9P0K7-3	Ankycorbin	RAI14	-0.188624064	0.201515667	0	79
Q14671;Q14671-2;H0YEH2;E	Pumilio homolog 1	PUM1	0.602442424	0.200834837	2.43E-105	26
Q01081;Q01081-2;Q01081-4	Splicing factor U2AF 35 kDa	U2AF1	0.516981761	0.198832036	5.35E-64	13
Q9NX63;C9JRZ6;G3V1K1;F8W	Coiled-coil-helix-coiled-coil-	CHCHD3	-1.189995448	0.196037814	1.51E-66	21
Q9Y2R4;A8MTP9	Probable ATP-dependent RI	DDX52	1.130537669	0.194322716	1.07E-194	49
Q96I24;Q96I24-2	Far upstream element-bind	FUBP3	-0.64308993	0.194071205	7.78E-151	35
O95831;O95831-3;Q1L6K4;O	Apoptosis-inducing factor 1	AIFM1	0.900039673	0.192599762	1.18E-48	33
E7EWI9;P51991-2	Heterogeneous nuclear ribo	HNRNPA3	0.223924637	0.190719298	0	77
Q9NY12;Q9NY12-2	H/ACA ribonucleoprotein co	GAR1	0.4815286	0.190536548	6.30E-128	68
Q86W92;Q86W92-2;Q86W92	Liprin-beta-1	PPFIBP1	0.230793635	0.18996197	9.90E-97	23
P62891;Q59GN2	60S ribosomal protein L39;F	RPL39;RPL39P5	-0.122477849	0.189746899	7.20E-10	17
P35637;P35637-2;H3BPE7	RNA-binding protein FUS	FUS	-0.053173065	0.189571008	0	137
Q86UE4	Protein LYRIC	MTDH	-0.116986593	0.188252848	0	149
P26368;P26368-2;K7ENG2	Splicing factor U2AF 65 kDa	U2AF2	0.856957118	0.185521197	1.05E-106	35
P05783;F8VZY9	Keratin, type I cytoskeletal 1	KRT18	-0.076917013	0.18533237	0	1113
Q13595;Q13595-2;B4DQI6;B4	Transformer-2 protein hom	TRA2A	-0.284554164	0.184329218	5.43E-23	13
O15145;C9JZD1;F8VR50	Actin-related protein 2/3 co	ARPC3	0.331354777	0.184328035	4.89E-19	10
Q9NVN8	Guanine nucleotide-binding	GNL3L	-0.784830729	0.182116139	5.43E-78	32
Q6ZNB6;Q6ZNB6-2	NF-X1-type zinc finger prote	NFXL1	0.660271962	0.17992998	3.47E-123	20

Q7RTV0	PHD finger-like domain-con	PHF5A	-0.308056513	0.178402527	1.36E-76	23
Q15629;G3XAN4;B4E0K2	Translocating chain-associat	TRAM1	-0.202694575	0.177912759	1.27E-173	16
O43290	U4/U6.U5 tri-snRNP-associa	SART1	0.918571472	0.177715326	2.34E-259	63
Q9UPQ9;Q9UPQ9-1;H0Y720	Trinucleotide repeat-contai	TNRC6B	0.643892288	0.177356345	5.15E-193	37
P62314;J3QLI9	Small nuclear ribonucleopro	SNRPD1	0.651203156	0.177299261	2.86E-86	24
Q8NCA5;Q8NCA5-2;E9PH82;I	Protein FAM98A	FAM98A	0.386144002	0.17567217	0	58
P11021	78 kDa glucose-regulated pr	HSPA5	0.409109751	0.175293604	5.86E-169	67
Q9NR56;C9JP00;Q9NR56-3;Q	Muscleblind-like protein 1	MBNL1	-0.363865534	0.173674514	2.08E-17	7
Q96EY1;Q96EY1-2;E7ES32	DnaJ homolog subfamily A m	DNAJA3	0.507000605	0.173034138	4.42E-176	50
P24928	DNA-directed RNA polymera	POLR2A	-0.560366313	0.172113246	8.94E-128	28
P14373;P14373-2;B0V297	Zinc finger protein RFP	TRIM27	0.205828985	0.170162913	8.90E-155	44
Q9NW13;Q9NW13-2	RNA-binding protein 28	RBM28	0.626188914	0.169785871	4.77E-38	17
Q9UNQ0;Q9UNQ0-2	ATP-binding cassette sub-fa	ABCG2	-0.166984558	0.169273721	1.80E-12	8
Q99700;H0YH87;Q99700-2;Q	Ataxin-2	ATXN2	1.103461583	0.169090907	1.17E-196	33
P61313;E7EQV9;E7EX53;E7EI	60S ribosomal protein L15;P	RPL15	0.774974187	0.166924361	4.46E-87	94
Q15269	Periodic tryptophan protein	PWP2	0.951863607	0.165617953	0	87
Q12872;Q12872-2;F5H5X1	Splicing factor, suppressor c	SFSWAP	0.472863515	0.164897006	2.33E-42	12
Q9BUT9	Protein FAM195A	FAM195A	-0.290112178	0.16351826	3.64E-148	7
O95782;O95782-2	AP-2 complex subunit alpha	AP2A1	0.817335765	0.162315816	5.52E-176	63
O95816;B4DXE2	BAG family molecular chape	BAG2	-0.307242076	0.161369171	0	77
P60842;J3QLN6;J3KS25;J3QR	Eukaryotic initiation factor 4	EIF4A1;EIF4A2	0.372303009	0.159739214	5.20E-55	16
Q9UBB4;Q9UBB4-2	Ataxin-10	ATXN10	0.477761587	0.157376366	3.76E-45	21
Q92522	Histone H1x	H1FX	-0.197905858	0.157251741	7.97E-49	21
Q15366-4;Q15366-6;Q15366	Poly(rC)-binding protein 2	PCBP2	-0.269107183	0.156921698	1.16E-109	43
Q15393	Splicing factor 3B subunit 3	SF3B3	0.257021586	0.156651002	0	95
Q99933;Q99933-4;Q99933-3	BAG family molecular chape	BAG1	0.169298172	0.155946435	1.22E-14	7
Q8WYP5;Q8WYP5-2;Q8WYP5	Protein ELYS	AHCTF1	-0.379303614	0.154767144	3.65E-238	85
P46783;F6U211	40S ribosomal protein S10	RPS10	0.95215416	0.154484272	3.40E-68	39
Q02543;M0R1A7;M0R3D6;M	60S ribosomal protein L18a	RPL18A	0.185241699	0.153504094	2.77E-208	141
P62979;P0CG47;J3QS39;J3QT	Ubiquitin-40S ribosomal pro	RPS27A;UBB;UBC;U	-0.0658741	0.153116411	2.78E-207	65
Q9BRJ6;H7C0T1;H7C2R9;C9J	Uncharacterized protein C7	C7orf50	0.541238785	0.152504371	6.41E-16	10
P62318;B4DJP7	Small nuclear ribonucleopro	SNRPD3	0.310180664	0.15141752	2.64E-166	44
Q07157;Q07157-2;G5E9E7;G	Tight junction protein ZO-1	TJP1	-1.217394511	0.148998556	0	56
Q14103;Q14103-3;H0YA96;H	Heterogeneous nuclear ribo	HNRNPD	0.114749908	0.145104978	5.57E-56	47
Q7Z7K6;Q7Z7K6-3;Q7Z7K6-2	Centromere protein V	CENPV	0.654734929	0.142059391	4.69E-44	19
P62995;P62995-3	Transformer-2 protein hom	TRA2B	0.240797679	0.141842293	5.00E-118	50
Q13428-6;J3KQ96;Q13428-7;	Treacle protein	TCOF1	-0.269649506	0.140757857	1.33E-210	67
Q7Z2W4;C9J6P4;Q7Z2W4-2;C	Zinc finger CCCH-type antivi	ZC3HAV1	0.34281985	0.140638185	0	77
Q14498;Q14498-2;Q14498-3	RNA-binding protein 39	RBM39	0.355051041	0.140426194	2.87E-272	57
E7ERS3;Q86VM9;Q86VM9-2	Zinc finger CCCH domain-co	ZC3H18	0.298557281	0.139594271	2.80E-82	21
Q9H9Y6;F5GZX4;Q9H9Y6-2;F	DNA-directed RNA polymera	POLR1B	0.955305735	0.138780055	5.14E-103	47
Q9P2I0	Cleavage and polyadenylati	CPSF2	0.698778788	0.137786343	1.37E-109	28
CON__Streptavidin			0.092631658	0.137631475	1.23E-246	40
Q96AG4	Leucine-rich repeat-contain	LRRC59	0.218009949	0.137022576	0	111
P51116	Fragile X mental retardation	FXR2	0.07637914	0.136813322	0	172
P04908;P0C0S8;Q99878;Q93	Histone H2A type 1-B/E;Hist	HIST1H2AB;HIST1H2	-0.238797506	0.135991587	1.95E-86	138

Q96EP5;Q96EP5-2;K7EQ02;K7	DAZ-associated protein 1	DAZAP1	0.904953003	0.135131912	1.09E-91	18
Q8IWR0;I3L2K5	Zinc finger CCCH domain-co	ZC3H7A	0.979173024	0.135126917	0	50
P48634	Protein PRRC2A	PRRC2A	0.062806447	0.132684516	1.92E-255	95
O60563	Cyclin-T1	CCNT1	0.599611282	0.13158414	2.86E-151	22
Q14684;Q14684-2	Ribosomal RNA processing p	RRP1B	0.749744415	0.129634665	0	161
Q96PK6	RNA-binding protein 14	RBM14	0.137941996	0.128570308	3.18E-188	117
Q9BZK7	F-box-like/WD repeat-conta	TBL1XR1	0.091720581	0.128496153	2.30E-40	9
P46776;E9PJD9;E9PLL6	60S ribosomal protein L27a	RPL27A	-0.10433197	0.127110629	5.23E-97	65
Q5UIP0;Q5UIP0-2	Telomere-associated protei	RIF1	0.197634379	0.126350045	3.41E-284	109
Q8TDD1;Q8TDD1-2	ATP-dependent RNA helicas	DDX54	0.828330358	0.125860164	1.86E-108	29
Q9NPE3	H/ACA ribonucleoprotein co	NOP10	-0.365484238	0.125310231	2.57E-138	39
O15143	Actin-related protein 2/3 co	ARPC1B	-0.269436518	0.124686599	3.01E-71	18
P31689;B7Z5C0	DnaJ homolog subfamily A r	DNAJA1	0.439849854	0.123342321	1.87E-104	30
Q9NUL3;Q9NUL3-2;E9PH62;Q	Double-stranded RNA-bindi	STAU2	-0.572971344	0.122894449	2.26E-148	35
Q9UHB9;F5H5Y3;G3V1U4;Q9	Signal recognition particle s	SRP68	0.611813863	0.121805884	3.84E-107	31
Q14676;Q14676-2;E9PGY5;Q	Mediator of DNA damage cl	MDC1	0.828233083	0.121606135	0	89
Q9UKV8;Q9UKV8-2	Protein argonaute-2	AGO2	-0.527801514	0.120807156	2.33E-204	49
Q9BYJ9;F8W840	YTH domain family protein 1	YTHDF1	-0.496463776	0.120804016	4.01E-86	33
Q6UN15;Q6UN15-5;Q6UN15	Pre-mRNA 3-end-processing	FIP1L1	0.412851334	0.11978872	9.18E-121	41
P49458;E9PE20;P49458-2	Signal recognition particle 9	SRP9	-0.183481216	0.118756164	4.74E-40	26
P63010;P63010-2;Q7Z451;K7	AP-2 complex subunit beta	AP2B1	0.802996953	0.118365899	4.00E-216	79
P39656;E7EWT1	Dolichyl-diphosphooligosac	DDOST	0.361787796	0.117910755	6.06E-35	20
P20042	Eukaryotic translation initia	EIF2S2	0.578777949	0.11737932	1.58E-203	60
P46779;P46779-2;P46779-3;H	60S ribosomal protein L28	RPL28	-0.073652903	0.116304636	1.67E-39	35
O15446;O15446-2	DNA-directed RNA polymera	CD3EAP	-0.537755966	0.115728047	1.58E-180	54
Q7Z6E9;Q7Z6E9-2;Q7Z6E9-4	E3 ubiquitin-protein ligase F	RBBP6	0.717622121	0.115464492	3.57E-232	46
Q9NQG5	Regulation of nuclear pre-m	RPRD1B	0.230208715	0.115274147	5.05E-48	16
P05386	60S acidic ribosomal protein	RPLP1	0.399029414	0.11496863	8.61E-158	25
Q9P275;Q9P275-2;E9PEW0	Ubiquitin carboxyl-terminal	USP36	0.550163905	0.114546753	0	109
Q16637;Q16637-4;Q16637-2	Survival motor neuron prot	SMN1	-0.143174489	0.114459314	4.21E-198	39
P07910;P07910-4;P07910-2;C	Heterogeneous nuclear ribo	HNRNPC	0.137295405	0.114066067	3.48E-85	62
P18887;F5H8D7	DNA repair protein XRCC1	XRCC1	-0.057131449	0.113512728	0	121
Q9Y3Y2;Q9Y3Y2-4;Q9Y3Y2-3;	Chromatin target of PRMT1	CHTOP	-0.246803284	0.113385253	1.02E-33	12
Q9H2Y7;E9PE29	Zinc finger protein 106	ZNF106	-0.62471962	0.112709791	0	63
P61978;P61978-2;Q5T6W1	Heterogeneous nuclear ribo	HNRNPK	0.440475464	0.112523146	0	90
P51571;H7C1C6;A6NLM8	Translocon-associated prote	SSR4	0.535345713	0.11195644	6.23E-84	17
P61158;F5H3P5;B4DXW1	Actin-related protein 3	ACTR3	0.130383174	0.111787975	6.03E-54	25
O60506;O60506-2	Heterogeneous nuclear ribo	SYNCRIP	0.126490911	0.110470857	0	139
Q53GS9;B9A018;B8ZZD1;G5E	U4/U6.U5 tri-snRNP-associa	USP39	0.830551147	0.107925422	2.24E-270	60
Q13769	THO complex subunit 5 hom	THOC5	0.477025986	0.107911745	8.89E-136	26
CON__Q8VED5			0.231921514	0.106406353	7.52E-76	17
O00571;O00571-2;O15523;B4	ATP-dependent RNA helicas	DDX3X;DDX3Y	0.089059194	0.10596625	0	178
Q9NR30;Q9NR30-2	Nucleolar RNA helicase 2	DDX21	0.404545466	0.10506887	0	235
P04843;B7Z4L4	Dolichyl-diphosphooligosac	RPN1	0.372299194	0.105054069	1.00E-118	47
P62857	40S ribosomal protein S28	RPS28	0.56444931	0.103081757	8.25E-43	14
Q9H089	Large subunit GTPase 1 hom	LSG1	-0.504476547	0.102496895	3.80E-35	19

P04844;P04844-2	Dolichyl-diphosphooligosac	RPN2	0.201605479	0.102393853	3.42E-55	23
P26038	Moesin	MSN	-0.462253571	0.102159432	6.84E-42	15
P62244;I3L3P7;I3L246;H3BN	40S ribosomal protein S15a	RPS15A	0.306032181	0.101794211	7.61E-118	75
P07437;Q5JP53;F8VYX6;F8V	Tubulin beta chain;Tubulin	TUBB;TUBB2B;TUBE	-0.633658091	0.100886966	6.86E-148	46
C9JKI3;Q03135-2;P56539;E9	Caveolin;Caveolin-1;Caveoli	CAV1;CAV3	0.469692866	0.100877903	8.92E-07	8
O43707;D6PXK4;F5GXS2	Alpha-actinin-4	ACTN4	-0.620875676	0.100788434	1.58E-293	67
Q14807;B7Z265;H3BRB3	Kinesin-like protein KIF22	KIF22	-0.467016856	0.100660384	2.47E-89	23
Q9NSU2;Q9NSU2-2;Q9NSU2-	Three prime repair exonucl	TREX1	0.757205327	0.099657717	1.47E-142	66
Q9BPX5	Actin-related protein 2/3 co	ARPC5L	-0.280153275	0.099490505	0.00071224	4
Q9Y446	Plakophilin-3	PKP3	0.049195608	0.09884952	1.99E-84	57
P55769;B1AHD1	NHP2-like protein 1;NHP2-li	NHP2L1	-0.24163119	0.098186081	3.09E-70	39
Q8IVT2	Mitotic interactor and subs	MISP	-0.274806976	0.097920765	3.73E-290	82
Q9BUQ8	Probable ATP-dependent R	DDX23	0.902580897	0.09734573	0	132
Q8IWX8;J3QK89	Calcium homeostasis endo	CHERP	0.167039235	0.097287232	1.05E-117	34
P62249;M0R210;M0R3H0	40S ribosomal protein S16	RPS16	-0.194525401	0.097152543	4.56E-51	82
P06753-2;Q5VU66;P06753-3;	Tropomyosin alpha-3 chain	TPM3	-0.29712232	0.096502596	5.51E-107	28
P47756-2;B1AK87;B1AK88;P4	F-actin-capping protein sub	CAPZB	0.039557139	0.095964671	3.91E-205	46
P56537;B7ZBG9	Eukaryotic translation initia	EIF6	-0.338989894	0.095955493	4.03E-263	27
P08621;P08621-2;P08621-3	U1 small nuclear ribonucle	SNRNP70	-0.441486359	0.095720615	1.18E-71	23
Q9BX40;Q5TBP9;Q9BX40-2	Protein LSM14 homolog B	LSM14B	0.126488368	0.095672071	1.11E-05	4
Q9BVJ6;F8WD00;E9PEL7;Q9	U3 small nucleolar RNA-ass	UTP14A	-0.487159729	0.095468878	4.36E-203	57
Q3B726	DNA-directed RNA polymer	TWISTNB	-0.334627787	0.095225752	1.64E-187	23
P16989;P16989-3;P16989-2	Y-box-binding protein 3	YBX3	-0.129456202	0.093825788	3.37E-220	22
Q9BVP2;Q9BVP2-2	Guanine nucleotide-binding	GNL3	-0.480167389	0.093465851	1.13E-121	39
P62861;E9PR30	40S ribosomal protein S30	FAU	-0.128075918	0.093317641	6.51E-14	25
P67809;H0Y449	Nuclease-sensitive element	YBX1	0.150520325	0.093275167	0	120
Q9NZ01;B3KSQ1;M0R3C3	Very-long-chain enoyl-CoA	TECR	0.124434153	0.092498749	7.42E-99	38
Q9UNX4	WD repeat-containing prot	WDR3	0.495845159	0.09217351	0	185
O94905;E5RHW4	Erlin-2	ERLIN2	0.48789533	0.091890298	4.75E-93	28
E7EUE1;P28288;F5GYC1;P28	ATP-binding cassette sub-fa	ABCD3	-0.269155502	0.091565116	5.16E-245	73
O76021;J3QSV6;I3L3U9	Ribosomal L1 domain-conta	RSL1D1	0.504037221	0.091113671	3.89E-292	88
Q9Y4A5;H0Y4W2;Q9Y4A5-2;I	Transformation/transcriptic	TRRAP	-0.466222127	0.09038134	9.90E-157	58
Q99459	Cell division cycle 5-like pro	CDC5L	0.402372996	0.089980535	0	105
P18124;A8MUD9	60S ribosomal protein L7	RPL7	-0.200439453	0.089825947	1.88E-242	134
P57678;I3L2C7	Gem-associated protein 4	GEMIN4	0.236846924	0.089757059	6.90E-206	83
P13010	X-ray repair cross-complem	XRCC5	0.224533081	0.089399889	0	991
Q7LGA3;Q7LGA3-2;A6ND99;	Heparan sulfate 2-O-sulfotr	HS2ST1	0.227979024	0.088076856	2.71E-51	29
P43243;A8MXP9;D6REM6;D6	Matrin-3	MATR3	0.076528549	0.088063706	0	167
P56192;A6NC17;B3KVK7	Methionine--tRNA ligase, cy	MARS	0.296885173	0.087757908	0	52
P21796;C9JI87	Voltage-dependent anion-s	VDAC1	-0.13344574	0.085867723	4.13E-19	19
P62241;Q5JR95	40S ribosomal protein S8	RPS8	0.075837453	0.084973255	0	215
Q8NI36;D6R922	WD repeat-containing prot	WDR36	0.510762533	0.084776027	0	131
Q92576;Q92576-2;E7EVH3	PHD finger protein 3	PHF3	-0.369639079	0.084662186	0	102
P62879;E7EP32;C9JXA5;C9JIS	Guanine nucleotide-binding	GNB2	0.193232218	0.083365127	3.21E-113	40
Q5JTH9;Q5JTH9-2;Q5JTH9-3;	RRP12-like protein	RRP12	0.279253006	0.082954242	0	123
P52597	Heterogeneous nuclear ribo	HNRNPF	0.120400747	0.082752812	7.10E-251	41

Q9BYG3;C9J808;C9J6C5	MKI67 FHA domain-interact	MKI67IP	0.527893066	0.081968606	9.16E-143	27
P54132;H0YNU5	Bloom syndrome protein	BLM	0.240589778	0.080340173	7.10E-220	54
P78406;E9PQ57;E9PPG9	mRNA export factor	RAE1	0.33769544	0.07997101	8.16E-245	35
Q9UMS4;F5GY56	Pre-mRNA-processing facto	PRPF19	0.396805445	0.079836447	0	121
P68431;P84243;K7EK07;K7EN	Histone H3.1;Histone H3.3;I	HIST1H3A;H3F3A;H3	0.076531728	0.079631996	1.80E-114	290
Q5SW79;H0Y2V6;Q5SW79-2;	Centrosomal protein of 170	CEP170	0.241763433	0.078720707	1.11E-42	17
Q9BV38;K7EIR0;K7EML4	WD repeat-containing prote	WDR18	0.230246226	0.077749925	1.36E-49	23
Q14966;Q14966-3;Q14966-4	Zinc finger protein 638	ZNF638	-0.144885381	0.077051751	0	145
Q12788;J3KNP2	Transducin beta-like protein	TBL3	0.218910853	0.076574437	0	117
Q9HAV0;C9JD14	Guanine nucleotide-binding	GNB4	-0.165062586	0.075404791	7.12E-96	8
P49454	Centromere protein F	CENPF	0.116288503	0.075365798	0	604
P49916;P49916-2;K7ERZ5	DNA ligase 3	LIG3	-0.043404897	0.074516811	0	317
P38646	Stress-70 protein, mitochon	HSPA9	0.173075994	0.073971001	3.41E-64	25
O43293	Death-associated protein ki	DAPK3	0.277876536	0.073849599	2.72E-53	22
Q9H0D6;Q9H0D6-2;B4DZC3	5-3 exoribonuclease 2	XRN2	0.107152303	0.07372706	3.13E-148	40
P25398	40S ribosomal protein S12	RPS12	-0.145244598	0.07360775	3.76E-164	62
P48651;B4DE85;Q9BUQ5	Phosphatidylserine synthase	PTDSS1	0.232301076	0.073297923	3.26E-102	22
P08590;P05976;P05976-2	Myosin light chain 3;Myosin	MYL3;MYL1	0.131204605	0.073105744	0.0011525	6
P02545;P02545-2;Q6UYC3;P(	Prelamin-A/C;Lamin-A/C	LMNA	-0.07038943	0.072050727	4.03E-77	32
P62266;D6RD47	40S ribosomal protein S23	RPS23	0.190172831	0.072001629	1.96E-43	39
Q15070;E7EVY0;Q15070-2;J3	Mitochondrial inner membr	OXA1L	-0.188414892	0.071812898	8.66E-16	8
P60866;P60866-2;E5RJX2	40S ribosomal protein S20	RPS20	-0.267499924	0.071734764	4.75E-35	51
Q86UP2;Q86UP2-2;Q86UP2-3	Kinectin	KTN1	-0.178264618	0.071673839	0	195
P41252;J3KR24	Isoleucine--tRNA ligase, cyto	IARS	-0.170585632	0.070917803	0	147
O60287	Nucleolar pre-ribosomal-ass	URB1	0.300289154	0.070874016	0	143
P62913;P62913-2;Q5VVC8	60S ribosomal protein L11	RPL11	-0.118606567	0.070514981	1.37E-64	65
P12956;B1AHC8;B1AHC9;F5H	X-ray repair cross-complem	XRCC6	0.204321543	0.070135025	0	927
Q9BY89	Uncharacterized protein KIA	KIAA1671	-0.381790161	0.070104095	2.25E-156	36
Q9H0H5	Rac GTPase-activating prote	RACGAP1	-0.29189237	0.070041408	1.13E-151	41
Q9P1U0	DNA-directed RNA polymera	ZNRD1	-0.270434062	0.069865681	3.46E-39	9
CON__P08729			0.092525482	0.069725168	0	185
O15160;O15160-2;E7EQB9;D	DNA-directed RNA polymera	POLR1C	0.244497935	0.06900627	2.02E-110	26
Q14157-1;Q14157;Q14157-4	Ubiquitin-associated protein	UBAP2L	0.12935257	0.068914402	0	160
Q14254;J3QLD9;E7EMK3	Flotillin-2	FLOT2	0.189719518	0.068327525	8.67E-148	29
Q9H2U1;Q9H2U1-3;Q9H2U1-	Probable ATP-dependent RI	DHX36	0.256106695	0.067880546	1.91E-271	49
Q16698;B7Z6B8;E5RFV2;E5R.	2,4-dienoyl-CoA reductase,	DECR1	0.356396993	0.066587015	1.41E-257	66
Q13523;F5H2U2;H0YDJ3	Serine/threonine-protein ki	PRPF4B	-0.276676814	0.06615252	4.35E-60	34
O43795;E9PDF6;O43795-2	Unconventional myosin-Ib	MYO1B	-0.420419693	0.065692175	0	120
P29558;P29558-2;B4DN88;E7	RNA-binding motif, single-st	RBMS1	0.210026423	0.065556253	2.36E-88	16
P54136;P54136-2;F5H3T8	Arginine--tRNA ligase, cytop	RARS	-0.134105682	0.06546227	1.15E-194	112
Q86TG7	Retrotransposon-derived pr	PEG10	0.184673309	0.065264811	2.29E-121	40
Q86V48;Q86V48-2;Q86V48-3	Leucine zipper protein 1	LUZP1	-0.074485779	0.065071805	0	193
H9KV59;O43663;O43663-2;F	Protein regulator of cytokin	PRC1	0.154167175	0.065033251	8.23E-110	46
P12236;I7HJJ0	ADP/ATP translocase 3	SLC25A6	-0.127056758	0.064649728	4.44E-173	144
Q14527;Q14527-2	Helicase-like transcription fa	HLTF	0.115907669	0.064167816	0	327
P80723;P80723-2	Brain acid soluble protein 1	BASP1	0.483149211	0.063617273	3.94E-126	28

Q6PKG0;Q6PKG0-3	La-related protein 1	LARP1	0.241060893	0.063572577	0	143
P26373;J3QSB4;P26373-2	60S ribosomal protein L13	RPL13	0.131276449	0.06349933	1.26E-154	103
Q96IZ0	PRKC apoptosis WT1 regula	PAWR	0.190090179	0.063269575	1.65E-98	16
Q03701	CCAAT/enhancer-binding pı	CEBPZ	0.292724609	0.06326656	0	115
P14868;Q68CR9	Aspartate--tRNA ligase, cytc	DARS;DKFZp781B11	0.351195653	0.062440767	1.02E-145	64
Q5T6F2;F5H2U4;E7EWG4;A6	Ubiquitin-associated protei	UBAP2	-0.173709234	0.062190309	2.05E-235	36
Q9BY77;B4DMM2	Polymerase delta-interactin	POLDIP3	0.232696533	0.062116874	2.39E-245	81
P14678;P63162;P14678-2;B3	Small nuclear ribonucleoprc	SNRPB;SNRPN	-0.292150497	0.061461108	8.14E-36	33
P36578;H3BM89	60S ribosomal protein L4	RPL4	0.117121379	0.0613138	0	269
P08107;P08107-2;F8VZJ4;E7E	Heat shock 70 kDa protein 1	HSPA1A	-0.019503911	0.061302754	0	68
P16615;P16615-5;P16615-2;F	Sarcoplasmic/endoplasmic ı	ATP2A2	0.219546	0.061231733	1.33E-75	33
Q12797;F5H667	Aspartyl/asparaginyl beta-h	ASPH	0.090648651	0.061175829	0	116
P62701	40S ribosomal protein S4, X	RPS4X	-0.155138016	0.060893353	1.05E-207	188
O14974;O14974-5;O14974-2	Protein phosphatase 1 regu	PPP1R12A	-0.060338338	0.060828995	0	171
Q9UM00;J3QQY2;Q9UM00-2	Transmembrane and coiled-	TMCO1	-0.177295685	0.06072748	5.35E-25	9
P07814	Bifunctional glutamate/prol	EPRS	-0.172912598	0.060678567	0	260
P42677;Q5T4L4	40S ribosomal protein S27	RPS27	-0.090937297	0.060561366	5.25E-48	55
O75477;B0QZ43	Erlin-1	ERLIN1	0.321115494	0.060282197	7.38E-40	18
Q9NVP1	ATP-dependent RNA helicas	DDX18	0.562267303	0.059971732	1.59E-142	48
P46782;M0R0F0;M0QZN2;M(	40S ribosomal protein S5;4(	RPS5	0.104817708	0.059963393	1.47E-96	37
P47897;B4DWJ2;H7C0R3	Glutamine--tRNA ligase	QARS	-0.367730459	0.059907632	1.13E-202	59
O75152;E9PQ61;E9PBY7	Zinc finger CCCH domain-co	ZC3H11A	-0.293258667	0.059899764	9.14E-257	36
Q969Q0;P83881;H7BZ11;H0Y	60S ribosomal protein L36a-	RPL36AL;RPL36A	-0.124975204	0.059621374	3.57E-22	24
P46778;M0R181;G3V1B3	60S ribosomal protein L21	RPL21	0.142843246	0.059089025	4.27E-78	75
Q07666;Q07666-3;Q07666-2	KH domain-containing, RNA	KHDRBS1	-0.098495483	0.059004907	3.21E-255	83
Q06787-6;Q06787-4;Q06787-	Fragile X mental retardatior	FMR1	0.026473999	0.058918829	0	149
Q92499	ATP-dependent RNA helicas	DDX1	0.153116226	0.058659003	0	166
P09234	U1 small nuclear ribonuclec	SNRPC	0.225678126	0.05861328	1.29E-67	11
Q15046;Q15046-2	Lysine--tRNA ligase	KARS	0.347000122	0.058340312	3.39E-240	49
Q9Y4W2;Q9Y4W2-2;Q9Y4W2	Ribosomal biogenesis prote	LAS1L	0.137249629	0.057631722	1.14E-66	16
P11142;E9PKE3;P11142-2;E9I	Heat shock cognate 71 kDa	HSPA8	-0.061753591	0.057630338	0	209
Q7L2E3-2;Q7L2E3-3;H7BXY3	Putative ATP-dependent RN	DHX30	0.208616257	0.057407115	0	123
P15880;H0YEN5;E9PQD7;I3L4	40S ribosomal protein S2	RPS2	-0.112714767	0.057059432	9.72E-104	97
Q14690	Protein RRP5 homolog	PDCD11	0.409331004	0.056909987	0	214
Q16666;Q16666-2;Q16666-3;	Gamma-interferon-inducibl	IFI16	-0.351898829	0.056611018	2.32E-199	69
P62753;A2A3R5	40S ribosomal protein S6	RPS6	0.098481496	0.056411252	2.57E-98	95
P62277;J3KMX5	40S ribosomal protein S13	RPS13	0.226308187	0.055497162	3.88E-138	118
Q8IY81	pre-rRNA processing protei	FTSJ3	0.389081319	0.055350241	1.31E-285	42
P52272-2;P52272	Heterogeneous nuclear ribc	HNRNPM	0.056627909	0.054978692	0	437
Q6SPF0;E9PIW9	Atherin	SAMD1	0.144804001	0.054724327	5.33E-131	33
Q9Y3F4;B4DNJ6;B0AZV0	Serine-threonine kinase rec	STRAP	0.238067627	0.054343552	2.00E-73	34
P49750;P49750-4;P49750-3;F	YLP motif-containing protei	YLPM1	0.092388153	0.054211526	2.52E-239	75
Q96SB3	Neurabin-2	PPP1R9B	0.035043081	0.053538294	1.80E-09	5
CON__P02769			0.464349747	0.053191397	0	254
P98175-2;P98175;P98175-3;F	RNA-binding protein 10	RBM10	-0.146750768	0.05255712	0	82
P62899;C9JU56;B7Z4E3;P628	60S ribosomal protein L31	RPL31	0.067014058	0.052467288	4.39E-53	51

P52907	F-actin-capping protein sub	CAPZA1	0.031592051	0.052283959	9.68E-150	60
P06748;P06748-2;P06748-3	Nucleophosmin	NPM1	0.139532725	0.052228292	0	852
P63261;I3L3I4;I3L3I0;I3L1U9;	Actin, cytoplasmic 2;Actin, c	ACTG1	-0.052032471	0.052074056	0	1837
Q00059;H7BYN3;A8MRB2	Transcription factor A, mito	TFAM	0.166582108	0.051469217	2.89E-120	37
P61254;J3QRI7;J3QQQ9;J3Q(	60S ribosomal protein L26;6	RPL26;KRBA2;RPL26	-0.027118047	0.051442207	8.99E-60	55
P46777	60S ribosomal protein L5	RPL5	-0.088989894	0.051275101	1.07E-168	105
P05141	ADP/ATP translocase 2;ADP	SLC25A5	-0.044323603	0.050959593	6.99E-115	46
Q16629;Q16629-3;Q16629-2	Serine/arginine-rich splicing	SRSF7	-0.28404808	0.050932038	7.84E-153	39
Q9UHI6	Probable ATP-dependent RI	DDX20	-0.19775327	0.050313978	0	100
P13645;CON__P13645	Keratin, type I cytoskeletal 1	KRT10	-0.105246226	0.050153275	0	233
O15226;G3V1N1	NF-kappa-B-repressing facto	NKRF	0.071233114	0.049957332	0	98
P08754	Guanine nucleotide-binding	GNAI3	-0.096733729	0.049325273	2.32E-165	44
P62750;H7BY10;K7EJV9;K7ER	60S ribosomal protein L23a	RPL23A	-0.038513184	0.049272637	6.04E-70	87
O95602;B9ZVN9	DNA-directed RNA polymera	POLR1A	-0.237196604	0.048719437	0	128
Q8IX01;M0R2Z9;M0R3F6;Q8I	SURP and G-patch domain-c	SUGP2	0.327231725	0.048588623	2.01E-52	30
Q7Z417	Nuclear fragile X mental ret	NUFIP2	-0.124330521	0.048579117	0	108
P40429;M0QYS1	60S ribosomal protein L13a	RPL13A	-0.097590764	0.048370349	4.17E-84	115
O75643	U5 small nuclear ribonuclec	SNRNP200	0.283317566	0.048095688	0	327
P42766;H0Y3A0;F2Z388	60S ribosomal protein L35	RPL35	-0.070740382	0.046446129	1.14E-158	46
O43143	Putative pre-mRNA-splicing	DHX15	-0.054734548	0.046340307	2.17E-194	109
Q15029;Q15029-2;K7EJ81	116 kDa U5 small nuclear ril	EFTUD2	0.278768539	0.046262586	0	195
P78527;E7EUY0;P78527-2	DNA-dependent protein kin	PRKDC	-0.062975566	0.045865634	0	1090
Q9NYF8;Q9NYF8-2;Q9NYF8-4	Bcl-2-associated transcriptio	BCLAF1	0.145033518	0.045339884	0	135
P04003	C4b-binding protein alpha c	C4BPA	-0.326857249	0.045149884	0	61
P41091;Q2VIR3;Q2VIR3-2;F8	Eukaryotic translation initia	EIF2S3;EIF2S3L	-0.169295629	0.045083383	1.02E-140	68
Q92804;Q92804-2;K7EPT6	TATA-binding protein-assoc	TAF15	-0.091424306	0.045053095	0	67
P27635;F8W7C6;Q96L21;B8A	60S ribosomal protein L10;6	RPL10;RPL10L	0.116713206	0.045039108	8.73E-262	137
Q9Y3U8;J3QSB5	60S ribosomal protein L36	RPL36	-0.076628367	0.044846976	2.09E-108	63
P22087;M0QXL5;M0R2Q4;M	rRNA 2-O-methyltransferas	FBL	-0.126659393	0.04463292	9.26E-237	129
Q13283;F5H4D6	Ras GTPase-activating prote	G3BP1	0.190055847	0.044492027	0	223
Q09161	Nuclear cap-binding protein	NCBP1	-0.252756119	0.044286917	1.79E-128	24
P37108;H0YLA2	Signal recognition particle 1	SRP14	0.124384562	0.043658721	1.67E-50	21
Q9UHB6;Q9UHB6-4;Q9UHB6	LIM domain and actin-bindi	LIMA1	-0.039580027	0.043347445	0	256
P39880;P39880-2;P39880-3;F	Homeobox protein cut-like	CUX1	0.12167867	0.043251936	3.28E-142	27
P09874	Poly [ADP-ribose] polymera	PARP1	0.094080607	0.043210667	0	2716
P11387	DNA topoisomerase 1	TOP1	0.097653707	0.042752421	0	190
Q14126	Desmoglein-2	DSG2	-0.084225337	0.042592805	4.26E-113	29
Q86Y07;Q86Y07-3;Q86Y07-4	Serine/threonine-protein ki	VRK2	0.184058507	0.042459441	2.35E-30	17
Q04837;E7EUY5;C9K0U8	Single-stranded DNA-bindin	SSBP1	-0.086399078	0.042044644	1.69E-224	85
Q00839;Q00839-2	Heterogeneous nuclear ribo	HNRNPU	0.112108866	0.042025213	1.37E-265	78
P62304;A6NHK2	Small nuclear ribonucleopro	SNRPE	-0.172011058	0.041716357	2.62E-40	9
Q5T3I0;Q5T3I0-3;E9PAV9;F8	G patch domain-containing	GPATCH4	-0.103925069	0.04167613	0	95
Q13813;Q13813-3;Q13813-2	Spectrin alpha chain, non-er	SPTAN1	0.143533707	0.041307538	0	268
O75533	Splicing factor 3B subunit 1	SF3B1	0.285294215	0.040907601	0	131
P46013-2	Antigen KI-67	MKI67	-0.120347341	0.040557404	0	7
CON__P15636			-0.177829742	0.039917673	0	228

P05388;Q3B7A4;F8VWS0;F8\	60S acidic ribosomal proteir	RPLP0;RPLP0P6	0.107060115	0.039801675	4.12E-199	210
P62424;Q5T8U2;Q5T8U3	60S ribosomal protein L7a	RPL7A	-0.063568115	0.039763549	0	187
Q07020;J3QQ67;F8VWC5;G3	60S ribosomal protein L18	RPL18	0.090079625	0.039753322	5.35E-135	66
Q14980;Q14980-2;Q14980-4	Nuclear mitotic apparatus p	NUMA1	0.170436223	0.039750458	0	150
Q08211	ATP-dependent RNA helicas	DHX9	0.1681633	0.039690463	0	214
Q6WCQ1;Q6WCQ1-2;Q6WC(	Myosin phosphatase Rho-in	MPRIP	0.029628118	0.039524542	0	136
Q96I25;Q5W011;Q5W010	Splicing factor 45	RBM17	-0.124991099	0.03919017	1.40E-146	23
P83111;P83111-2	Serine beta-lactamase-like	LACTB	0.103129705	0.039059723	0	136
P18621;J3QQT2;J3KRX5;J3QL	60S ribosomal protein L17	RPL17	-0.052298228	0.038079295	5.97E-82	97
Q9Y230;B3KQ59;M0R0Y3	RuvB-like 2	RUVBL2	-0.149974187	0.038029565	9.65E-114	33
P08865;C9J9K3;A6NE09	40S ribosomal protein SA	RPSA;RPSAP58	-0.162754695	0.037365085	2.98E-71	11
P46781;B5MCT8;C9JM19	40S ribosomal protein S9	RPS9	-0.092318853	0.037054917	5.53E-91	74
Q8NI27	THO complex subunit 2	THOC2	0.205177943	0.037007899	0	78
P61247;D6RG13;D6RAT0;F5H	40S ribosomal protein S3a	RPS3A	0.042772929	0.036307748	0	165
P19338;H7BY16	Nucleolin	NCL	-0.106084824	0.035660141	0	230
Q69YQ0;F5H1H6;F8WAN1;C9	Cytospin-A	SPECC1L	-0.10425059	0.035453985	0	65
P62280;M0QZC5	40S ribosomal protein S11	RPS11	0.044268926	0.03539309	1.12E-131	138
P11940;P11940-2;E7EQV3;E7	Polyadenylate-binding prot	PABPC1	0.090333303	0.03518215	0	400
Q9NVI7-2;Q9NVI7;H0Y2W2;C	ATPase family AAA domain-	ATAD3A	-0.034694672	0.03492067	0	124
Q13310-3;Q13310;Q13310-2	Polyadenylate-binding prot	PABPC4	-0.102282842	0.034406957	0	125
Q8NC51;Q8NC51-3;Q8NC51-	Plasminogen activator inhib	SERBP1	0.310577393	0.034223507	0	49
Q9BZF9;H0YNH8;G3XAG2;F5I	Uveal autoantigen with coil	UACA	0.133086522	0.034093588	4.45E-125	22
Q9NZI8;Q9NZI8-2	Insulin-like growth factor 2	IGF2BP1	-0.091298421	0.033985463	4.79E-226	82
Q14677-2;Q14677-3;Q14677	Clathrin interactor 1	CLINT1	-0.132141113	0.032943454	1.65E-105	38
P84090;G3V279	Enhancer of rudimentary ho	ERH	0.07371521	0.032912371	2.09E-128	49
Q3MHD2;Q3MHD2-2;K7ELG9	Protein LSM12 homolog	LSM12	0.109951019	0.031386841	7.44E-14	14
Q9UDY2;Q9UDY2-3;Q9UDY2-	Tight junction protein ZO-2	TJP2	-0.275241852	0.031149573	7.89E-50	24
O14979-3;O14979-2;O14979	Heterogeneous nuclear ribo	HNRPDL	-0.082021713	0.030887359	4.27E-219	79
Q00325-2;F8VVM2;Q00325	Phosphate carrier protein, r	SLC25A3	0.047609329	0.030803835	3.75E-161	71
P39019;M0R2L9;M0QXK4;M(	40S ribosomal protein S19	RPS19	-0.055765152	0.030719497	4.81E-103	66
P49790;F6QR24	Nuclear pore complex prote	NUP153	0.051777522	0.029820068	0	77
Q9Y2W1	Thyroid hormone receptor-	THRAP3	0.10243543	0.029746322	0	124
O94906;O94906-2	Pre-mRNA-processing facto	PRPF6	0.252869288	0.029230009	0	132
Q9UN86	Ras GTPase-activating prote	G3BP2	-0.06293869	0.028927703	0	134
P83731;C9JXB8;C9JNW5	60S ribosomal protein L24	RPL24	0.035640717	0.028719316	4.71E-41	70
P35527;CON__P35527	Keratin, type I cytoskeletal	KRT9	-0.046009064	0.02839055	0	229
Q9GZS1-2;Q9GZS1;E7EX70	DNA-directed RNA polymer	POLR1E	-0.081334432	0.02813142	3.77E-62	34
P68133;P68032;P63267;P627	Actin, alpha skeletal muscle	ACTA1;ACTC1;ACTG	-0.038832347	0.028047643	0	169
P62917;E9PKZ0;E9PKU4;G3V	60S ribosomal protein L8	RPL8	0.060602824	0.027858603	3.04E-288	105
P62906	60S ribosomal protein L10a	RPL10A	0.046988169	0.02781219	2.46E-130	121
Q15233;Q15233-2;H7C367;C	Non-POU domain-containin	NONO	0.156016032	0.027261674	0	470
Q01082	Spectrin beta chain, non-ery	SPTBN1	-0.115846634	0.027185306	0	220
O75955;B4DVY7;B0V109;A2A	Flotillin-1	FLOT1	0.075298945	0.02710247	5.73E-133	18
Q00610;Q00610-2	Clathrin heavy chain 1	CLTC	0.209073385	0.026700985	0	207
Q12965	Unconventional myosin-Ie	MYO1E	-0.130340576	0.02623282	0	147
Q9NZB2;Q9NZB2-4;Q9NZB2-(	Constitutive coactivator of F	FAM120A	0.088909785	0.026118948	0	97

Q08J23;Q08J23-2;G3V1R4	tRNA (cytosine(34)-C(5))-me	NSUN2	-0.117675781	0.026001056	1.02E-152	27
P46940;H0YLE8	Ras GTPase-activating-like p	IQGAP1	0.093566895	0.025967663	2.49E-286	102
Q92841;Q92841-1;Q92841-2	Probable ATP-dependent RI	DDX17	0.012505849	0.025602894	0	494
Q10570	Cleavage and polyadenylati	CPSF1	-0.11953481	0.024838079	0	89
O43390;Q2L7G6	Heterogeneous nuclear ribo	HNRNPR	0.013704936	0.024611271	1.81E-256	99
Q02880-2;Q02880;E9PCY5	DNA topoisomerase 2-beta;	TOP2B	0.112443924	0.024292897	0	91
P26599;P26599-2;P26599-3;A	Polypyrimidine tract-binding	PTBP1	0.049533844	0.024091264	0	210
Q8TEQ6	Gem-associated protein 5	GEMIN5	0.147439957	0.024079321	0	47
Q86V81	THO complex subunit 4	ALYREF	0.032365163	0.023726366	0	85
Q9BZI7;Q9BZI7-2	Regulator of nonsense trans	UPF3B	0.098591487	0.023197851	1.02E-46	20
O60832;O60832-2;C9IYT0;H7	H/ACA ribonucleoprotein cc	DKC1	-0.068130493	0.023118875	0	143
Q09028;Q09028-3;Q09028-4	Histone-binding protein RBI	RBBP4	-0.085817973	0.022979261	6.56E-198	19
O14950;P19105;J3QRS3;P248	Myosin regulatory light chai	MYL12B;MYL12A;M	0.018238068	0.022752378	2.31E-156	44
P46013	Antigen KI-67	MKI67	0.053525289	0.022636114	0	1341
P45880;P45880-2;P45880-1;E	Voltage-dependent anion-se	VDAC2	0.055490494	0.022492731	2.61E-43	26
Q9Y2X3	Nucleolar protein 58	NOP58	0.086426417	0.02249155	0	112
Q16643;A8MV58;D6R9W4	Drebrin	DBN1	0.049020767	0.022351962	0	115
P62910;D3YTB1;F8W727	60S ribosomal protein L32	RPL32	0.030359268	0.022339743	7.74E-82	65
O00425	Insulin-like growth factor 2	IGF2BP3	-0.040712357	0.022050892	3.38E-228	74
P08708;P0CW22;H0YN88;H0Y	40S ribosomal protein S17;4	RPS17;RPS17L	0.03704071	0.022026852	1.75E-166	104
P36873;F8VYE8;P36873-2;F8V	Serine/threonine-protein pl	PPP1CC	-0.10174942	0.021889434	0	25
P42285;F5H7E2	Superkiller viralicidic activity	SKIV2L2	-0.138899485	0.021787051	7.42E-137	36
Q86SQ0;Q86SQ0-2;Q86SQ0-3	Pleckstrin homology-like do	PHLDB2	-0.058052699	0.021669738	8.60E-117	24
P32969;D6RAN4;H0Y9V9;E7E	60S ribosomal protein L9	RPL9	-0.050457001	0.021517006	2.25E-121	78
Q9P0L0;Q9P0L0-2	Vesicle-associated membrai	VAPA	-0.112434387	0.021158437	4.70E-84	34
P84098;J3QR09;J3KTE4	60S ribosomal protein L19;F	RPL19	-0.027706782	0.021113515	1.64E-87	42
Q13247;Q13247-3	Serine/arginine-rich splicing	SRSF6	-0.07492129	0.020918845	1.77E-46	45
Q96C57;E7ENF1;G5EA44;F5H	Uncharacterized protein C1	C12orf43	-0.093044281	0.020617655	1.83E-119	30
P23246;P23246-2	Splicing factor, proline- and	SFPQ	0.112603505	0.02045868	0	513
O00567;Q5JXT2	Nucleolar protein 56	NOP56	-0.078982671	0.020373389	0	156
P08174;P08174-2;H3BLV0;Q1	Complement decay-accelera	CD55	0.031395594	0.020185969	1.82E-184	50
P06746;E7EW18;B7Z1W5;H0	DNA polymerase beta	POLB	0.047376633	0.020001195	4.08E-191	72
O00159-2	Unconventional myosin-Ic	MYO1C	0.026741028	0.019770608	0	310
O75475;O75475-2;O75475-3	PC4 and SFRS1-interacting p	PSIP1	-0.048936844	0.019753458	0	44
P84103;B4E241	Serine/arginine-rich splicing	SRSF3;SFRS3	-0.079807281	0.019563664	5.66E-116	41
Q9Y224;G3V4C6	UPF0568 protein C14orf166	C14orf166	0.051845551	0.01946659	3.18E-257	85
P60660;P60660-2;F8W1R7;G	Myosin light polypeptide 6	MYL6	-0.027866364	0.019336904	8.17E-176	82
Q9BWF3;E9PB51;Q9BWF3-4;	RNA-binding protein 4	RBM4	0.046536128	0.019287549	3.33E-128	34
Q9UG63;Q75MJ1	ATP-binding cassette sub-fa	ABCF2	0.154354731	0.019261694	1.91E-246	70
Q9Y3I0	tRNA-splicing ligase RtcB ho	C22orf28	-0.045758565	0.01864441	2.30E-252	99
Q9BQG0;Q9BQG0-2;I3L1L3	Myb-binding protein 1A	MYBBP1A	0.064024607	0.018545225	0	241
Q96DH6;Q96DH6-2;B4DHE8;	RNA-binding protein Musas	MSI2	-0.035651525	0.01792137	9.60E-78	27
Q07065	Cytoskeleton-associated pro	CKAP4	0.047580083	0.017839696	1.60E-166	49
P08670;B0YJC4;B0YJC5	Vimentin	VIM	-0.084545771	0.017821409	0	418
P30050;P30050-2	60S ribosomal protein L12	RPL12	0.02988561	0.017550266	3.54E-171	103
P62140;E7ETD8;C9J9S3;C9JP4	Serine/threonine-protein pl	PPP1CB	-0.025916417	0.017010013	0	182

P17844;J3KTA4;B4DLW8	Probable ATP-dependent RI	DDX5	-0.01370875	0.016998363	0	429
Q5T5U3;E7ESW5;Q5T5U3-3;F	Rho GTPase-activating prot	ARHGAP21	0.015026093	0.016585183	4.68E-165	50
Q1ED39	Lysine-rich nucleolar protei	KNOP1	-0.072722117	0.016397207	0	78
P63208;E5RJR5;E7ERH2;F8W	S-phase kinase-associated p	SKP1	0.052379608	0.015557886	4.85E-51	22
Q8WYQ5;Q8WYQ5-3	Microprocessor complex su	DGCR8	-0.079186757	0.015409681	2.93E-90	24
O76094;O76094-2	Signal recognition particle s	SRP72	0.072907766	0.015132131	1.90E-232	61
Q9H6R0;I3L1L6;Q9H6R0-2;Q	Putative ATP-dependent RN	DHX33	-0.074112574	0.015036975	9.81E-188	55
Q9HCE1;Q5JR04;Q9HCE1-2	Putative helicase MOV-10	MOV10	0.057825724	0.015006591	0	126
Q9Y5S9;Q9Y5S9-2	RNA-binding protein 8A	RBM8A	0.008385976	0.014787975	9.49E-168	34
P60709	Actin, cytoplasmic 1;Actin, c	ACTB	-0.042658488	0.014671459	0	64
P62158;H0Y7A7;E7ETZ0;E7EN	Calmodulin	CALM1;CALM2	0.008892059	0.014290161	7.50E-87	56
Q13155;A8MU58;F8W950	Aminoacyl tRNA synthase co	AIMP2	0.096893311	0.014243557	5.68E-53	29
Q14444;Q14444-2;G3V153;E	Caprin-1	CAPRIN1	-0.050722758	0.013855391	0	218
Q8N8A6	ATP-dependent RNA helicas	DDX51	0.079984665	0.013648501	1.39E-52	25
P23396;J3KN86;E9PL09;F2Z2	40S ribosomal protein S3	RPS3	0.029817581	0.013317315	3.28E-301	181
Q99729-3;D6R9P3;D6RD18;D	Heterogeneous nuclear ribo	HNRNPAB	0.042352676	0.013258325	3.42E-19	17
Q99590;Q99590-2;F8VXG7;F	Protein SCAF11	SCAF11	-0.045466105	0.013066882	0	84
P62851	40S ribosomal protein S25	RPS25	0.021113714	0.012943191	6.88E-24	33
Q9Y265;Q9Y265-2;E7ETR0	RuvB-like 1	RUVBL1	0.061347961	0.012914809	5.32E-302	76
Q99877;Q93079;P58876;O60	Histone H2B type 1-N;Histo	HIST1H2BN;HIST1H2	0.032132467	0.012658906	0	248
Q02241;Q02241-2;H7BYN4;B	Kinesin-like protein KIF23	KIF23	-0.045483907	0.012348276	1.02E-219	56
Q96C19;H0Y4Y4	EF-hand domain-containing	EFHD2	-0.073593775	0.012339224	6.57E-106	38
P62316;P62316-2	Small nuclear ribonucleopro	SNRPD2	-0.048912048	0.01216884	1.52E-69	37
P61353;K7ELC7;K7EQQ9	60S ribosomal protein L27	RPL27	-0.009798686	0.012150409	5.73E-143	96
P13987;E9PNW4;E9PR17;H0Y	CD59 glycoprotein	CD59	-0.011289597	0.01186574	4.67E-37	36
Q9H0A0;E7ESU4	N-acetyltransferase 10	NAT10	-0.06768163	0.011420948	0	381
Q13435;E9PPJ0	Splicing factor 3B subunit 2	SF3B2	-0.031016032	0.011380386	0	83
P05187;P10696	Alkaline phosphatase, place	ALPP;ALPPL2	-0.006908417	0.011168014	1.99E-262	69
P35268;K7ERI7;K7ELC4;K7EM	60S ribosomal protein L22	RPL22	0.016244253	0.01094712	2.06E-177	73
P05387;H0YDD8	60S acidic ribosomal protei	RPLP2	-0.012053172	0.010858969	3.11E-269	117
P61160;P61160-2;F5H6T1	Actin-related protein 2	ACTR2	0.038699468	0.01049076	1.76E-56	17
O96019;O96019-2;H7C5S0	Actin-like protein 6A	ACTL6A	0.028393428	0.010456256	1.26E-229	38
Q9NX58	Cell growth-regulating nucl	LYAR	-0.025905609	0.010378283	0	117
P62888;E5RI99	60S ribosomal protein L30	RPL30	0.024414063	0.009821911	2.02E-272	89
P07355;H0YN42;A6NMY6;P0	Annexin A2;Annexin;Putativ	ANXA2;ANXA2P2	0.045404434	0.009324299	2.81E-48	15
Q09666	Neuroblast differentiation-a	AHNAK	0.009531021	0.009295634	1.23E-65	61
P50914;E7EPB3	60S ribosomal protein L14	RPL14	-0.013216019	0.00927096	7.91E-88	79
Q8NE71;Q8NE71-2;H0YGW7	ATP-binding cassette sub-fa	ABCF1	-0.038746516	0.009203026	1.46E-211	53
P46087;P46087-2;P46087-4;F	Putative ribosomal RNA me	NOP2	0.042083104	0.009175416	4.44E-241	108
Q9H6R4;Q9H6R4-4;Q9H6R4-	Nucleolar protein 6	NOL6	-0.07366689	0.008872482	0	149
O60841	Eukaryotic translation initia	EIF5B	0.030753454	0.007608074	4.81E-30	14
P22626;P22626-2	Heterogeneous nuclear ribo	HNRNPA2B1	0.016864777	0.007449645	0	253
E9PMS6		LMO7	-0.0133063	0.007420123	0	195
Q9Y520;Q9Y520-4;E7EPN9;Q	Protein PRRC2C	PRRC2C	0.014044444	0.007331856	0	188
CON__P35908;P35908	Keratin, type II cytoskeletal	KRT2	0.006359736	0.007261109	2.90E-270	88
Q6ZRS2;Q6ZRS2-3;Q6ZRS2-2	Helicase SRCAP	SRCAP	0.044411977	0.007126348	2.56E-160	42

Q9UBI6	Guanine nucleotide-binding	GNG12	-0.014805476	0.006971416	2.14E-23	13
Q15149;Q15149-6;Q15149-7	Plectin	PLEC	-0.027557373	0.006818576	0	1361
P62081;B5MCP9	40S ribosomal protein S7	RPS7	-0.028418223	0.006747345	1.37E-189	127
P62136;P62136-2;A6NNR3;E	Serine/threonine-protein pl	PPP1CA	0.009897868	0.006680097	3.13E-304	19
Q9P2E9;Q9P2E9-2;Q9P2E9-3	Ribosome-binding protein 1	RRBP1	-0.006429672	0.006647698	0	120
P26196	Probable ATP-dependent RI	DDX6	-0.022424698	0.006592557	1.53E-298	90
P62269	40S ribosomal protein S18	RPS18	0.008849462	0.006473844	8.08E-298	86
Q04637;Q04637-3;Q04637-5	Eukaryotic translation initia	EIF4G1	0.062699	0.006314078	0	73
P49756;P49756-2;P49756-3;E	RNA-binding protein 25	RBM25	0.037248611	0.006142721	4.69E-198	27
Q9HBH0;Q9HBH0-2	Rho-related GTP-binding pr	RHOF	0.009188334	0.00584693	2.73E-14	8
Q6P2Q9	Pre-mRNA-processing-splici	PRPF8	0.044700623	0.005506048	0	377
O75691	Small subunit processome c	UTP20	0.026435852	0.005458572	0	219
Q02878;F8W181	60S ribosomal protein L6	RPL6	0.00935936	0.005433983	4.52E-179	129
Q9Y5A9;Q9Y5A9-2	YTH domain family protein	YTHDF2	-0.013853709	0.005360107	1.56E-133	45
Q76FK4;Q76FK4-4;F5H101;Q	Nucleolar protein 8	NOL8	0.022940318	0.004938881	1.32E-279	69
O15042;O15042-2;E7ET15;O	U2 snRNP-associated SURP	U2SURP	0.009204865	0.004908572	4.70E-239	63
Q14247;Q14247-3;Q14247-2	Src substrate cortactin	CTTN	0.020079295	0.004886976	1.33E-161	70
P47914	60S ribosomal protein L29	RPL29	-0.006610235	0.004759672	1.07E-20	21
Q8TDN6	Ribosome biogenesis protei	BRIX1	-0.024536133	0.003825724	0	110
P11388;P11388-2;P11388-3;I	DNA topoisomerase 2-alpha	TOP2A	-0.009558996	0.003772896	0	299
P14923	Junction plakoglobin	JUP	-0.002857844	0.003673412	5.70E-223	70
P62263;E5RH77	40S ribosomal protein S14	RPS14	-0.005116145	0.00354246	4.74E-248	69
P62829;J3KT29;C9JD32;B9ZV	60S ribosomal protein L23	RPL23	-0.006421407	0.003461222	2.63E-195	105
Q71RC2;Q71RC2-3;Q71RC2-4	La-related protein 4	LARP4	-0.016270955	0.003272712	0	93
P19388	DNA-directed RNA polymer	POLR2E	-0.010197957	0.003129321	2.91E-70	22
P21333;P21333-2;Q5HY54	Filamin-A	FLNA	-0.016258876	0.003042645	0	175
Q9NX24;D6RC52;D6RCB9;J3C	H/ACA ribonucleoprotein co	NHP2	0.006680171	0.002886594	1.27E-278	54
Q9BUJ2;Q9BUJ2-4;B7Z4B8;Q	Heterogeneous nuclear ribo	HNRNPUL1	-0.004414241	0.002633105	4.54E-294	104
Q99848;H7C2Q8	Probable rRNA-processing p	EBNA1BP2	0.007567724	0.002557492	7.16E-78	45
Q15058	Kinesin-like protein KIF14	KIF14	-0.006120046	0.002419134	0	91
P04264;CON__P04264	Keratin, type II cytoskeletal	KRT1	-0.00206248	0.002267379	0	312
Q15717;B4DVB8;M0QZR9	ELAV-like protein 1	ELAVL1	0.003926595	0.001454258	5.54E-224	88
P35579;P35579-2	Myosin-9	MYH9	-0.001059214	0.000776695	0	777
Q14978-2;Q14978	Nucleolar and coiled-body p	NOLC1	-0.002361298	0.000565002	1.26E-266	130
P38432	Coilin	COIL	0.003501892	0.000560628	1.76E-109	31
Q8WWM7;Q8WWM7-6;Q8W	Ataxin-2-like protein	ATXN2L	-0.001240412	0.000461766	0	126
P39023;G5E9G0;H7C422;B5N	60S ribosomal protein L3	RPL3	-0.000403086	0.000334238	1.60E-264	241
Q92900;Q92900-2	Regulator of nonsense trans	UPF1	0.000610352	0.000262629	0	252
Q9H0S4	Probable ATP-dependent RI	DDX47	-0.00098292	0.000139688	0	45

Supplementary table 2						
**Identification and quantification of interacting protein groups for bait (H2B S112 GlcNAc monomer) versus control (wt H2B monomer) samples. Statistically significant interacting partners are denoted with a '+' in column F**						
**Majority Protein Ids**	**Protein names**	**Gene names**	**log2(Bait/Control)**	**Significance B value**	**Statistically Significant Interactor**	**PEP**
P00450;E9PFZ2;H7C5R1	Ceruloplasmin	CP	-3.933731	1.35E-15	+	8.79E-159
P63241;I3L397;I3L504;P6324	Eukaryotic translation initia	EIF5A;EIF5AL1	-3.576971	3.74E-13	+	3.73E-92
P60709	Actin, cytoplasmic 1;Actin, c	ACTB	3.030903	2.17E-08	+	6.00E-273
P32969;D6RAN4;E7ESE0;H0Y	60S ribosomal protein L9	RPL9	-2.60548	1.22E-07	+	7.62E-50
Q7Z401;H3BTW5;Q7Z401-2	C-myc promoter-binding pr	DENND4A	-2.494539	4.08E-07	+	0.007715
P43487;C9JJ34;C9JXG8;C9JG\	Ran-specific GTPase-activat	RANBP1	-2.230852	5.91E-06	+	9.97E-67
P11387	DNA topoisomerase 1	TOP1	2.230305	3.79E-05	+	6.75E-66
Q7L1Q6;Q7L1Q6-4;Q7L1Q6-3	Basic leucine zipper and W2	BZW1	2.216646	4.22E-05	+	2.01E-101
P06753-2;P06753-3;Q5VU59;	Tropomyosin alpha-3 chain	TPM3	-3.30904	5.64E-05	+	1.27E-43
Q9NVI1;F8W7R3;Q9NVI1-2;C	Fanconi anemia group I pro	FANCI	3.539764	0.000123629	+	2.41E-72
Q14624;Q14624-2;H7C0L5;Q	Inter-alpha-trypsin inhibitor	ITIH4	-1.878428	0.000137014	+	6.58E-86
P00403	Cytochrome c oxidase subu	MT-CO2	1.993118	0.000231137	+	4.50E-38
P62495;Q96CG1;B7Z7P8	Eukaryotic peptide chain rel	ETF1	-2.920744	0.000347697	+	7.41E-32
P19823;Q5T985	Inter-alpha-trypsin inhibitor	ITIH2	-1.754955	0.000367166	+	1.57E-51
Q27J81;Q27J81-2	Inverted formin-2	INF2	3.28673	0.000383421	+	3.16E-32
P98179	Putative RNA-binding prote	RBM3	-2.840151	0.000495113	+	3.63E-19
Q7Z7G8;Q7Z7G8-4;Q7Z7G8-3	Vacuolar protein sorting-ass	VPS13B	1.8582	0.000596431	+	4.18E-05
P12074;Q5T8I0;H0YIV9	Cytochrome c oxidase subu	COX6A1;COX6A1P2	3.171822	0.000624728	+	0.0022159
Q8NDY6	Class E basic helix-loop-heli	BHLHE23	1.823597	0.000753451	+	0.0041677
Q15125;C9J719;C9JJ78	3-beta-hydroxysteroid-Delta	EBP	3.06864	0.000955277	+	4.80E-08
P13667	Protein disulfide-isomerase	PDIA4	1.772488	0.00105662	+	9.23E-133
Q9H4A6	Golgi phosphoprotein 3	GOLPH3	1.748428	0.001235374		4.25E-19
Q96EP5;K7EQ02;Q96EP5-2	DAZ-associated protein 1	DAZAP1	-2.608852	0.001304372		1.00E-78
P09496-2;P09496;F5H6N3;P0	Clathrin light chain A	CLTA	-2.551218	0.001643107		4.36E-82
Q6UXN9	WD repeat-containing prote	WDR82	1.670712	0.00202111		1.01E-60
P82979;Q567R9;H0YHG0;F8V	SAP domain-containing ribo	SARNP;CIP29	-1.516016	0.002089947		9.72E-103
Q08211	ATP-dependent RNA helicas	DHX9	2.869242	0.002092643		8.80E-78
P78527;E7EUY0;P78527-2	DNA-dependent protein kin	PRKDC	1.641226	0.002423928		1.93E-199
P33240;P33240-2;E7EWR4;E9	Cleavage stimulation factor	CSTF2;CSTF2T	-2.422033	0.002715313		1.56E-28
P16403;P10412;P16402	Histone H1.2;Histone H1.4;H	HIST1H1C;HIST1H1E	-1.46331	0.002977465		3.27E-13
P55060;B4DUC5;P55060-3;F8	Exportin-2	CSE1L	1.582863	0.003445264		1.58E-45
P18846;B4DRF9	Cyclic AMP-dependent tran	ATF1	-2.349073	0.003572872		1.81E-16
P51858;A8K8G0;P51858-2;Q5	Hepatoma-derived growth f	HDGF	-2.292467	0.004400584		9.90E-96
P10606	Cytochrome c oxidase subu	COX5B	2.636181	0.004925247		4.03E-16
Q05519;Q5T760;Q05519-2	Serine/arginine-rich splicing	SRSF11	-2.178993	0.006602409		1.32E-27
O14787-2;O14787	Transportin-2	TNPO2	2.490864	0.008127471		3.11E-35
P18124;A8MUD9	60S ribosomal protein L7	RPL7	1.43066	0.008194288		4.92E-35

Q2TAY7;B4E3L0	WD40 repeat-containing pr	SMU1	-2.06321	0.009825279	3.37E-99
P14625	Endoplasmin	HSP90B1	1.387947	0.010314439	1.41E-107
Q9UJU6;Q9UJU6-2;Q9UJU6-3	Drebrin-like protein	DBNL	-2.033878	0.010837785	1.33E-28
Q9Y3B3;Q6JUT2;Q9Y3B3-2	Transmembrane emp24 dor	TMED7;TICAM2	2.396786	0.011091364	1.15E-09
P19827;F5H7E1;P19827-2;P1	Inter-alpha-trypsin inhibitor	ITIH1	-2.007128	0.01184099	8.05E-157
P36578;H3BM89	60S ribosomal protein L4	RPL4	1.351997	0.012463591	3.22E-55
Q9HCG8;B7WP27;B7WP74	Pre-mRNA-splicing factor C\	CWC22	2.346035	0.013060028	8.03E-85
P04792;F8WE04	Heat shock protein beta-1	HSPB1	-1.96756	0.013475911	1.42E-48
P84103;B4E241	Serine/arginine-rich splicing	SRSF3;SFRS3	-1.205643	0.014424212	1.57E-29
P21796;C9JI87	Voltage-dependent anion-se	VDAC1	2.314293	0.01444298	5.03E-60
P17844;B4DLW8;J3KTA4	Probable ATP-dependent RI	DDX5	1.298483	0.016397436	1.14E-169
P61313;E7EQV9;E7ENU7	60S ribosomal protein L15;F	RPL15	1.255165	0.020342019	9.58E-17
P61964	WD repeat-containing prote	WDR5	2.185143	0.021489505	7.69E-24
P38159;Q96E39;P38159-2;H0	RNA-binding motif protein,	RBMX;RBMXL1	-1.13059	0.02178882	3.42E-78
Q9NYL9;H0YNJ8	Tropomodulin-3	TMOD3	-1.123825	0.022590895	1.21E-209
P14927;P14927-2;B7Z2R2	Cytochrome b-c1 complex s	UQCRB	-1.799099	0.022877348	1.34E-17
P12236;I7HJJ0	ADP/ATP translocase 3	SLC25A6	1.229916	0.02300412	1.42E-67
P62158;H0Y7A7;E7ETZ0;E7EN	Calmodulin	CALM1;CALM2	-1.089409	0.027080476	9.19E-58
Q96BP2;A6NJX6	Coiled-coil-helix-coiled-coil-	CHCHD1	-1.736664	0.027592311	1.33E-10
Q14011;K7ELV6;K7ENX8;K7E.	Cold-inducible RNA-binding	CIRBP	-1.081627	0.028196304	7.46E-115
P14854	Cytochrome c oxidase subu	COX6B1	1.186441	0.028299512	3.06E-43
Q9Y570;F5H2D4;J3QT22	Protein phosphatase methy	PPME1	-1.726671	0.028419938	5.06E-05
Q96B54;M0QXZ5	Zinc finger protein 428	ZNF428	-1.724455	0.028606375	1.09E-07
O14980;C9IZS4;C9JQ02;C9JV	Exportin-1	XPO1	2.074486	0.029742018	1.29E-27
Q14103;Q14103-3;H0YA96;H	Heterogeneous nuclear ribo	HNRNPD	-1.066395	0.030495408	5.92E-55
P39023;H7C422;G5E9G0;H7C	60S ribosomal protein L3	RPL3	1.159866	0.032028935	3.48E-53
Q9UHB6;Q9UHB6-4;F8VQE1;	LIM domain and actin-bindi	LIMA1	-1.055786	0.032190217	9.11E-244
P21127;P21127-12;P21127-4	Cyclin-dependent kinase 11	CDK11B;CDK11A	2.040298	0.032789217	9.36E-29
O00571;O00571-2;O15523;B	ATP-dependent RNA helicas	DDX3X;DDX3Y	2.02795	0.033953713	8.58E-63
P36954;K7EKS1	DNA-directed RNA polymer	POLR2I	-1.65325	0.035182552	4.98E-170
Q86VP6;Q86VP6-2	Cullin-associated NEDD8-dis	CAND1	1.13509	0.035877778	2.08E-130
O43324;D6RCQ0;H0YAL7;O43	Eukaryotic translation elong	EEF1E1;hCG_20432	-1.645809	0.035938912	5.08E-08
P35637;P35637-2;H3BPE7	RNA-binding protein FUS	FUS	-1.028553	0.036914549	3.96E-49
P52298;E9PAR5;C9JQX9;B3KS	Nuclear cap-binding protein	NCBP2	-1.016911	0.039107637	6.49E-19
P41236;E7EMN6;E7EUI7;Q6N	Protein phosphatase inhibit	PPP1R2;PPP1R2P3	-1.602255	0.040649954	5.89E-13
O43809;H3BND3;H3BV41	Cleavage and polyadenylati	NUDT21	-0.9989548	0.042706438	5.84E-37
Q9NY12;Q9NY12-2	H/ACA ribonucleoprotein co	GAR1	-0.9979153	0.042923088	1.84E-35
Q6FI13;P0C0S8;Q9BTM1;Q16	Histone H2A type 2-A;Histo	HIST2H2AA3;HIST1H	-0.9937096	0.043809136	3.15E-68
Q04637;Q04637-3;Q04637-5	Eukaryotic translation initia	EIF4G1	1.087231	0.044435652	2.90E-49
Q9UBS4	DnaJ homolog subfamily B r	DNAJB11	-1.562435	0.045405228	1.65E-105
Q6ZW49;Q6ZW49-4;Q6ZW49	PAX-interacting protein 1	PAXIP1	1.909166	0.047075365	3.50E-52
P17096;P17096-3;H7BYM6	High mobility group protein	HMGA1	-1.544928	0.047639829	1.51E-13
Q9NXL6;Q9NXL6-2;J3KPQ7	SID1 transmembrane family	SIDT1	-1.517628	0.051308352	0.0023186
Q7KZ85	Transcription elongation fac	SUPT6H	1.049192	0.052411784	3.05E-208

P16220;P16220-3;P16220-2;(	Cyclic AMP-responsive elem	CREB1;CREM	-1.506956	0.052805303	2.32E-09
Q01844;Q01844-6;B0QYK0;Q	RNA-binding protein EWS	EWSR1	-0.9484062	0.054371595	5.60E-139
Q8N1F7;H3BVG0;Q8N1F7-2	Nuclear pore complex prote	NUP93	1.846777	0.055528827	4.59E-17
Q9Y5B9	FACT complex subunit SPT1	SUPT16H	1.035215	0.055628694	4.08E-189
Q9UHA3;H3BMQ2	Probable ribosome biogene	RSL24D1	-1.4828	0.056329025	0.00043987
Q86T24	Transcriptional regulator Ka	ZBTB33	1.031662	0.056472261	9.29E-105
P62857	40S ribosomal protein S28	RPS28	-0.9378338	0.057121094	2.05E-42
Q9UHX1-4;Q9UHX1-6;Q9UH)	Poly(U)-binding-splicing fact	PUF60	-0.9366798	0.057428073	6.70E-111
P60903	Protein S100-A10	S100A10	-1.450411	0.061358738	0.0025205
P08195;P08195-3;P08195-2;I	4F2 cell-surface antigen hea	SLC3A2	1.011839	0.061375827	2.47E-211
P52907	F-actin-capping protein sub	CAPZA1	-1.449451	0.06151322	1.24E-25
P25440;H0Y6K2;H0Y5T9;P25	Bromodomain-containing p	BRD2;DKFZp313H13	1.004726	0.063219025	6.45E-243
A6NHQ2	rRNA/tRNA 2-O-methyltran	FBLL1	-1.429802	0.064748672	5.01E-07
Q8NE71;H0YGW7;Q8NE71-2	ATP-binding cassette sub-fa	ABCF1	1.785368	0.065048719	5.08E-56
Q07065	Cytoskeleton-associated pro	CKAP4	1.780746	0.06581659	1.33E-84
Q86V81	THO complex subunit 4	ALYREF	-0.9056301	0.066217118	5.23E-188
Q9P013	Spliceosome-associated pro	CWC15	-0.9047966	0.06646754	1.38E-70
Q92979	Ribosomal RNA small subun	EMG1	-0.9008656	0.067659127	3.98E-64
P01617;P01614;P06309;P063	Ig kappa chain V-II region TEW;Ig kappa chain V-		-0.8989601	0.068243001	1.37E-27
P26599;P26599-2;P26599-3;I	Polypyrimidine tract-binding	PTBP1	-0.8989182	0.068255905	5.50E-211
P13073;Q86WV2;H3BN72;H3	Cytochrome c oxidase subu	COX4I1	1.764494	0.068576525	2.00E-09
O00203;E5RJ68	AP-3 complex subunit beta-	AP3B1	0.9780369	0.070547867	7.25E-69
Q96ST2;Q96ST2-2;Q96ST2-3	Protein IWS1 homolog	IWS1	0.968874	0.073219287	5.50E-106
Q86VM9;E7ERS3;Q86VM9-2	Zinc finger CCCH domain-co	ZC3H18	1.730736	0.074613487	2.71E-17
P16401	Histone H1.5	HIST1H1B	-0.8787842	0.074683194	2.18E-18
Q15165;Q15165-3;J3QT77;Q	Serum paraoxonase/arylest	PON2	0.9634438	0.074841131	4.30E-81
P61978-2;P61978;P61978-3;(	Heterogeneous nuclear ribo	HNRNPK	-0.8761673	0.075553748	3.68E-245
Q9UFW8;C9JUJ0	CGG triplet repeat-binding	CGGBP1	-1.360332	0.077331191	2.18E-15
P62979;P0CG47;J3QS39;J3QT	Ubiquitin-40S ribosomal pro	RPS27A;UBB;UBC;U	0.9548283	0.077474437	9.38E-202
Q8N1G0;Q8N1G0-2;A2A3Q2;	Zinc finger protein 687	ZNF687	-1.355858	0.078205623	1.08E-58
Q9Y3Y2;Q9Y3Y2-4;Q9Y3Y2-3	Chromatin target of PRMT1	CHTOP	-1.353992	0.078572525	1.34E-31
Q9NR30;Q9NR30-2	Nucleolar RNA helicase 2	DDX21	0.9456711	0.080355543	1.01E-113
P20290;P20290-2;H0Y9Y1;D6	Transcription factor BTF3	BTF3	-1.342087	0.080947371	2.40E-47
P62241;Q5JR95	40S ribosomal protein S8	RPS8	0.9427147	0.081304081	1.48E-99
P42568;B7Z755;B1APT5	Protein AF-9	MLLT3	1.686241	0.083226514	6.08E-11
Q9BXW9;Q9BXW9-2;Q9BXW	Fanconi anemia group D2 p	FANCD2	1.673532	0.085829352	7.24E-95
Q7RTV0	PHD finger-like domain-con	PHF5A	1.661978	0.088252538	5.12E-28
Q99729-3;D6R9P3;D6RD18;D	Heterogeneous nuclear ribo	HNRNPAB	-0.83811	0.089175635	8.73E-46
Q96C19;H0Y4Y4	EF-hand domain-containing	EFHD2	-1.286554	0.092805225	5.42E-28
P12273	Prolactin-inducible protein	PIP	-1.282055	0.093824092	1.99E-28
Q8WYQ3;B5MBW9	Coiled-coil-helix-coiled-coil-	CHCHD10	1.632891	0.094597166	1.31E-14
Q16698;B7Z6B8;E5RJG7;E5RI	2,4-dienoyl-CoA reductase,	DECR1	-0.8234806	0.094911806	2.02E-101
P35613;P35613-2;P35613-4;I	Basigin	BSG	0.9027824	0.095028102	1.33E-145
P63104;E7EX29;E7ESK7;B0AZ	14-3-3 protein zeta/delta	YWHAZ	-0.8206635	0.096049644	7.84E-114

Q15269	Periodic tryptophan protein	PWP2	1.62429	0.096541563	4.71E-39
P53985;Q5T8R3;Q49A45;Q5T	Monocarboxylate transport	SLC16A1	1.614723	0.098742019	2.92E-21
P09651;P09651-3;F8VXY0;F8'	Heterogeneous nuclear ribo	HNRNPA1;HNRNPA1	-0.8130856	0.099164563	1.45E-166
P23284	Peptidyl-prolyl cis-trans isor	PPIB	-0.807251	0.101617394	1.56E-72
P43246;P43246-2;E9PHA6	DNA mismatch repair prote	MSH2	0.8848152	0.101780653	1.13E-121
P39656;E7EWT1	Dolichyl-diphosphooligosac	DDOST	0.8815002	0.103067275	1.30E-66
P49792;E9PGT0	E3 SUMO-protein ligase Rar	RANBP2	1.579988	0.107069328	1.38E-34
Q07955;Q07955-3;J3KTL2;Q0	Serine/arginine-rich splicing	SRSF1	-0.794241	0.107260695	5.61E-64
Q15427;Q5SZ64	Splicing factor 3B subunit 4	SF3B4	-0.7939243	0.107401067	1.56E-130
Q9C0C2;Q9C0C2-2	182 kDa tankyrase-1-bindin	TNKS1BP1	1.577923	0.107581567	2.43E-77
P23634;P23634-2;P23634-6;F	Plasma membrane calcium-	ATP2B4	-1.223873	0.107823218	1.96E-176
Q4G0J3;H0YA82;Q4G0J3-2	La-related protein 7	LARP7	1.576897	0.107836752	8.35E-52
Q9BRT6	Protein LLP homolog	LLPH	-1.221218	0.108499517	2.82E-12
Q9NQC3;Q9NQC3-2;Q9NQC3	Reticulon-4	RTN4	0.8659935	0.109258432	2.09E-68
P08240;P08240-2	Signal recognition particle r	SRPR	1.56946	0.109700478	5.08E-75
Q9UQ35	Serine/arginine repetitive m	SRRM2	0.862793	0.110572153	3.25E-162
P22626;P22626-2	Heterogeneous nuclear ribo	HNRNPA2B1	-0.7856712	0.111111628	1.97E-180
P05556;P05556-5;P05556-2;F	Integrin beta-1	ITGB1	1.551567	0.114288972	9.44E-24
Q12872;Q12872-2;F5H1A5	Splicing factor, suppressor c	SFSWAP	1.549686	0.114779893	4.16E-07
P27348	14-3-3 protein theta	YWHAQ	-0.7720566	0.11745308	6.07E-179
Q9BVK6	Transmembrane emp24 dor	TMED9	0.8432579	0.118863119	6.11E-45
O43681	ATPase ASNA1	ASNA1	1.532795	0.119263928	2.34E-16
P05114;A6NL93;A6NEL0	Non-histone chromosomal p	HMGN1	-1.180307	0.119350362	2.29E-15
Q13838;Q13838-2;F8VQ10;Q	Spliceosome RNA helicase D	DDX39B	-1.177315	0.120176338	6.26E-77
Q9UNP9;Q9UNP9-2;Q5TGA3;	Peptidyl-prolyl cis-trans isor	PPIE	1.524946	0.121393641	3.74E-15
O76071	Probable cytosolic iron-sulfu	CIAO1	1.507164	0.126328731	1.64E-12
P05783;F8VZY9	Keratin, type I cytoskeletal 1	KRT18	-0.7534924	0.12655354	0
P02545-2;Q5TCI8;Q6UYC3	Prelamin-A/C;Lamin-A/C	LMNA	1.50248	0.127654462	1.45E-185
Q8NC51;Q8NC51-2	Plasminogen activator inhib	SERBP1	-0.7511635	0.127732928	3.81E-225
P30050;P30050-2	60S ribosomal protein L12	RPL12	-0.7505875	0.128025951	2.87E-186
P35659;P35659-2;B4DFG0	Protein DEK	DEK	-0.7484283	0.129128953	3.06E-147
P01024	Complement C3;Compleme	C3	-0.7468452	0.129942371	0
Q9H3P2;B3KSP0;H0Y3X6;C9J	Negative elongation factor A	NELFA;WHSC2	-1.138828	0.131201986	1.87E-09
O60220	Mitochondrial import inner	TIMM8A	0.8098373	0.134168027	6.96E-49
Q8TED0;B4DXK8;H0Y8P4	U3 small nucleolar RNA-ass	UTP15	1.478029	0.134750428	7.82E-10
O43719	HIV Tat-specific factor 1	HTATSF1	0.8083916	0.134862998	2.03E-292
Q00325-2;F8VVM2;Q00325;F	Phosphate carrier protein, r	SLC25A3	0.8080387	0.135033036	1.01E-24
Q92804;K7EPT6;Q92804-2	TATA-binding protein-assoc	TAF15	-1.123322	0.135859984	7.68E-44
Q9Y3T9	Nucleolar complex protein 2	NOC2L	1.469315	0.137352031	9.37E-36
Q7Z5K2;Q7Z5K2-2;Q7Z5K2-3	Wings apart-like protein ho	WAPAL	0.7896538	0.144123705	0
Q9Y421;Q9Y421-3;K7EIY1	Protein FAM32A	FAM32A	-1.09433	0.144909925	5.25E-13
Q9Y224;G3V4C6	UPF0568 protein C14orf166	C14orf166	-0.7178173	0.145572459	4.17E-117
P52272;P52272-2;M0R019	Heterogeneous nuclear ribo	HNRNPM	0.7863464	0.145807739	4.89E-80
Q5C9Z4	Nucleolar MIF4G domain-co	NOM1	1.441277	0.145985441	3.21E-08

P16615;P16615-5;P16615-2;F	Sarcoplasmic/endoplasmic r	ATP2A2	0.7846775	0.146663223	3.19E-257
Q13151	Heterogeneous nuclear ribo	HNRNPA0	0.7846584	0.146673022	1.21E-130
P05141	ADP/ATP translocase 2;ADP	SLC25A5	0.7832069	0.147420205	1.27E-30
P60510;H3BTA2	Serine/threonine-protein ph	PPP4C	1.435974	0.147663878	1.65E-23
Q9NX58	Cell growth-regulating nucle	LYAR	-0.7133617	0.148093956	3.76E-223
Q15363;E7EQ72;F5GX39	Transmembrane emp24 dom	TMED2	1.420635	0.152602324	1.51E-29
O43172;O43172-2	U4/U6 small nuclear ribonu	PRPF4	1.414017	0.154771603	9.51E-35
Q5JPE7;P69849;Q4G177;F5H	Nodal modulator 2;Nodal m	NOMO2;NOMO3;NO	1.41325	0.15502442	2.87E-54
Q96PK6	RNA-binding protein 14	RBM14	-1.058926	0.156579547	1.25E-50
P11940;E7EQV3;P11940-2;E7	Polyadenylate-binding prote	PABPC1	-0.6948586	0.158923369	1.29E-264
Q9Y3F4;B4DNJ6;B0AZV0	Serine-threonine kinase rec	STRAP	-0.6946583	0.159043777	1.13E-197
E7EWI9;P51991-2;P51991	Heterogeneous nuclear ribo	HNRNPA3	-0.6928463	0.160136315	4.71E-56
P13639	Elongation factor 2	EEF2	-1.04324	0.161972142	9.80E-49
P08670;B0YJC4;B0YJC5	Vimentin	VIM	-0.6893673	0.16224988	0
P67809;H0Y449	Nuclease-sensitive element	YBX1	-0.6866112	0.163939191	6.31E-168
P02790	Hemopexin	HPX	-0.6840897	0.165496262	1.67E-138
Q5T8D3-3;Q5T8D3-4;Q5T8D3	Acyl-CoA-binding domain-co	ACBD5	1.376125	0.167644033	1.26E-19
Q9Y5L4	Mitochondrial import inner	TIMM13	0.7458038	0.167692937	3.41E-66
O15042;O15042-2;E7ET15;O1	U2 snRNP-associated SURP	U2SURP	0.7448521	0.168234838	6.56E-82
Q86TC9;F5GWA6;Q86TC9-3;Q	Myopalladin	MYPN	1.370214	0.169722547	1.26E-13
O95373	Importin-7	IPO7	0.741684	0.170048111	3.57E-151
Q9NX24;D6RC52;D6RCB9;J3C	H/ACA ribonucleoprotein co	NHP2	-0.6767082	0.170118365	1.64E-93
Q9UQ80;F8VTY8	Proliferation-associated pro	PA2G4	-0.6761093	0.17049759	2.88E-59
Q06546	GA-binding protein alpha ch	GABPA	0.7395515	0.171276827	8.15E-112
P52294	Importin subunit alpha-5	KPNA1	1.363966	0.171940721	1.72E-114
P01011;G3V5I3;G3V3A0	Alpha-1-antichymotrypsin;A	SERPINA3	-0.6718903	0.173187039	9.75E-159
Q92973;Q92973-2;Q92973-3	Transportin-1	TNPO1	0.7355995	0.173571575	2.00E-201
O14773;O14773-2	Tripeptidyl-peptidase 1	TPP1	0.735302	0.173745272	1.65E-36
Q7Z5J4;A8MXE8;Q7Z5J4-4;Q7	Retinoic acid-induced prote	RAI1	-1.003994	0.17607685	2.86E-05
Q8N2W9	E3 SUMO-protein ligase PIA	PIAS4	0.7290764	0.177409374	6.00E-105
P56192;A6NC17;B3KVK7	Methionine--tRNA ligase, cy	MARS	1.347237	0.177986258	2.33E-92
Q9H8H0;J3QKS9;J3QLQ6;J3Q	Nucleolar protein 11	NOL11	1.345476	0.178631537	3.73E-47
O96008;K7EJ57;O96008-2;K7	Mitochondrial import recep	TOMM40	1.34371	0.179280662	7.69E-05
O00425	Insulin-like growth factor 2	IGF2BP3	-0.9852905	0.183112562	8.00E-70
O15160;O15160-2;E7EQB9;D	DNA-directed RNA polymera	POLR1C	-0.9794407	0.185355319	4.32E-48
Q96B01;Q96B01-2;B4DUS5;C	RAD51-associated protein 1	RAD51AP1	-0.97509	0.187036437	6.08E-15
Q09666	Neuroblast differentiation-a	AHNAK	-0.9733982	0.187693197	3.70E-30
P05023;P05023-3;F5H3A1;P0	Sodium/potassium-transpor	ATP1A1	1.32111	0.187742172	5.43E-113
O00148;Q8N5M0;B1Q2N1;K7	ATP-dependent RNA helicas	DDX39A;DDX39	-0.6487179	0.188525912	2.77E-123
Q14331;E9PRR7;Q9BZ01;H0Y	Protein FRG1;Protein FRG1B	FRG1;FRG1B	1.31744	0.189143492	4.52E-23
Q8NFC6	Biorientation of chromosom	BOD1L1	1.315765	0.189785528	1.80E-86
Q96IZ7;H7C5Q0;Q96IZ7-2;C9	Serine/Arginine-related pro	RSRC1	-0.966444	0.190410679	6.44E-39
P11717	Cation-independent manno	IGF2R	1.313587	0.190623015	6.20E-29
O14617;O14617-5;O14617-4;	AP-3 complex subunit delta	AP3D1	1.311699	0.191351231	2.17E-84

Q9Y5J9;G3XAN8	Mitochondrial import inner	TIMM8B	0.7051792	0.192009183	1.24E-21
O60828;O60828-5;O60828-4;	Polyglutamine-binding prot	PQBP1	-0.643364	0.192208295	3.78E-62
Q9UJZ1;B4E1K7	Stomatin-like protein 2, mit	STOML2	-0.9564457	0.194368322	1.90E-05
P05387;H0YDD8	60S acidic ribosomal protein	RPLP2	-0.6367264	0.196846635	1.34E-120
P50991;B7Z9L0;P50991-2;B7	T-complex protein 1 subuni	CCT4	-0.9479904	0.197762017	2.30E-16
P62805	Histone H4	HIST1H4A	-0.9470921	0.198125125	1.84E-19
Q9BVP2;Q9BVP2-2	Guanine nucleotide-binding	GNL3	1.284174	0.202199607	5.97E-27
P04844;P04844-2	Dolichyl-diphosphooligosac	RPN2	0.6804771	0.208009987	3.78E-287
Q13868;B4DKK6;A3KFL5;A3K	Exosome complex compone	EXOSC2	-0.9228096	0.208125295	1.51E-20
Q6P2E9	Enhancer of mRNA-decappi	EDC4	-0.9091492	0.213909391	6.86E-12
P17480;P17480-2;E9PKP7	Nucleolar transcription fact	UBTF	0.6696663	0.215308991	2.15E-291
P20674;H3BNX8;H3BRI0	Cytochrome c oxidase subu	COX5A	1.252178	0.215365416	2.45E-09
Q8N9E0;G3XAI9;Q5BKY9	Protein FAM133A;Protein F	FAM133A;FAM133E	-0.9023952	0.216811597	0.00021616
Q15054;Q32N00;Q32MZ9	DNA polymerase delta subu	POLD3	-0.9023285	0.216840423	4.04E-34
E9PLD3;E9PRG8		C11orf48	-0.6082382	0.217685798	4.10E-12
Q96FJ2;P63167	Dynein light chain 2, cytopl	DYNLL2;DYNLL1	-0.8974438	0.218957206	2.40E-15
P24534	Elongation factor 1-beta	EEF1B2	-0.892601	0.221070413	2.81E-61
Q68E01;Q68E01-2;Q68E01-3	Integrator complex subunit	INTS3	0.6611786	0.22116749	9.02E-257
Q6NZI2;Q6NZI2-2	Polymerase I and transcript	PTRF	0.6542358	0.226043885	3.03E-88
Q9NUP9;G3V1D4	Protein lin-7 homolog C	LIN7C	-0.8725281	0.229985358	3.57E-16
P25311;C9JEV0	Zinc-alpha-2-glycoprotein	AZGP1	-0.8717022	0.230357554	6.61E-16
P23246;P23246-2	Splicing factor, proline- and	SFPQ	-0.5917969	0.230411577	9.23E-84
P20645;F5GXE0;H0YGE9;F5G	Cation-dependent mannose	M6PR	1.209211	0.234001657	2.16E-25
O95816;B4DXE2	BAG family molecular chape	BAG2	-0.8563042	0.237375453	1.71E-62
Q6W2J9;H7C2V9;A6NE70;Q6	BCL-6 corepressor	BCOR	1.196232	0.239849734	1.46E-36
Q9ULC4;Q9ULC4-3;Q9ULC4-2	Malignant T-cell-amplified s	MCTS1	-0.8495369	0.240507046	2.74E-48
P62861;E9PR30	40S ribosomal protein S30	FAU	-0.5790882	0.240604327	7.12E-09
P14923	Junction plakoglobin	JUP	0.6328068	0.241577561	4.78E-90
Q07666;Q07666-3;Q07666-2	KH domain-containing, RNA	KHDRBS1	-0.5775585	0.241852279	9.50E-41
Q07157;Q07157-2;G5E9E7;G	Tight junction protein ZO-1	TJP1	-0.8445377	0.242839051	1.49E-09
P84090;G3V279	Enhancer of rudimentary ho	ERH	-0.5755482	0.243499272	1.35E-126
Q13601;Q13601-2	KRR1 small subunit process	KRR1	1.18199	0.246384426	7.41E-14
F8W9Q2;Q8N7H5-2;M0QX35	RNA polymerase II-associat	PAF1	0.6245766	0.247738782	4.47E-71
P52701;F5H2F9;B4DF41;P52	DNA mismatch repair prote	MSH6	0.6217022	0.249916263	9.07E-294
Q09161	Nuclear cap-binding protein	NCBP1	1.173248	0.250456462	1.84E-37
Q99547;H3BSB3;H3BNT4	M-phase phosphoprotein 6	MPHOSPH6	-0.8280296	0.250652492	1.90E-26
Q9H089	Large subunit GTPase 1 hon	LSG1	0.6178684	0.252841291	2.77E-84
P20020;P20020-6;P20020-3;I	Plasma membrane calcium-	ATP2B1	1.165148	0.254271697	2.63E-196
Q9BTT0;Q5TB20;Q5TB19;E9F	Acidic leucine-rich nuclear p	ANP32E	0.6158257	0.254409554	1.98E-273
O14950;P19105;J3QRS3;P248	Myosin regulatory light chai	MYL12B;MYL12A;M	-0.5622501	0.254592329	1.27E-106
P62913;P62913-2;Q5VVC9;Q	60S ribosomal protein L11	RPL11	-0.5619736	0.254826709	1.22E-24
P30153;B3KQV6;F5H3X9;P30	Serine/threonine-protein pl	PPP2R1A;PPP2R1B	-0.818697	0.25514658	1.88E-30
Q7Z6E9;Q7Z6E9-2;Q7Z6E9-4;	E3 ubiquitin-protein ligase F	RBBP6	-0.8164215	0.256250761	1.80E-36
Q6PD62	RNA polymerase-associated	CTR9	1.158545	0.257411515	1.18E-68

P10620;F5H7F6;F5H6X2;P106	Microsomal glutathione S-tı	MGST1	-0.8069878	0.260863913	7.36E-08
Q9UG63;Q75MJ1	ATP-binding cassette sub-fa	ABCF2	1.151205	0.260932861	1.23E-17
P16435;H0Y4R2;F5H468;E7E\	NADPH--cytochrome P450 r	POR	0.6039505	0.263660249	1.72E-117
P09429;Q5T7C4;Q5T7C6;Q5T	High mobility group protein	HMGB1	-0.8007622	0.263939536	1.71E-19
O00264;B7Z1L3	Membrane-associated prog	PGRMC1	0.6030293	0.264387476	4.94E-42
Q13310;Q13310-2;B1ANR0;C	Polyadenylate-binding prot	PABPC4	-0.5432625	0.271033202	4.84E-198
O00410;O00410-3;O00410-2	Importin-5	IPO5	0.593029	0.272370583	1.19E-144
P31943;G8JLB6;E9PCY7;D6RI	Heterogeneous nuclear ribo	HNRNPH1	0.5920258	0.273180492	5.05E-233
P40937;P40937-2;F8W9B4;F!	Replication factor C subunit	RFC5	1.124424	0.274063781	8.33E-36
Q93009;F5H8E5;B7Z815;H3B	Ubiquitin carboxyl-terminal	USP7	1.123348	0.274600493	4.74E-32
Q9UKL0;J3KN32	REST corepressor 1	RCOR1	0.5889282	0.275691433	1.79E-34
Q9BXP5;Q9BXP5-4;Q9BXP5-2	Serrate RNA effector molec	SRRT	-0.5357056	0.277774614	6.56E-76
P54727;P54727-2;H0Y579	UV excision repair protein R	RAD23B	-0.7698994	0.279555368	7.58E-33
P56537;B7ZBG9	Eukaryotic translation initia	EIF6	-0.5329723	0.280240769	4.72E-96
Q12797;F5H667	Aspartyl/asparaginyl beta-h	ASPH	0.5816422	0.281659637	2.76E-86
Q8IZL8;F8WDZ1;I3L3A8;C9JF	Proline-, glutamic acid- and	PELP1	1.106958	0.282866564	5.80E-06
P62875	DNA-directed RNA polymer	POLR2L	1.097439	0.287744323	1.05E-07
Q9UKS6;E9PIY1	Protein kinase C and casein	PACSIN3	-0.5242805	0.288181887	0
Q9Y4Z0	U6 snRNA-associated Sm-lik	LSM4	-0.7480011	0.29100905	5.92E-08
P55769;B1AHD1	NHP2-like protein 1;NHP2-li	NHP2L1	-0.5206718	0.291523077	1.56E-30
P55010;H0YLZ1;H0YN40	Eukaryotic translation initia	EIF5	0.5691071	0.292131252	2.47E-160
Q9BQ61	Uncharacterized protein C1	C19orf43	-0.5197258	0.292403283	1.16E-07
Q9BY44;B4DQ14;F8WAE5;B4	Eukaryotic translation initia	EIF2A	-0.7438602	0.293209872	9.98E-44
Q9Y3E5;J3KQ48	Peptidyl-tRNA hydrolase 2,	PTRH2	0.5674992	0.293493147	8.17E-218
P46937;P46937-2;E9PRV2;P4	Yorkie homolog	YAP1	-0.7329617	0.29905563	1.24E-71
P48681	Nestin	NES	1.075415	0.299245729	3.39E-16
Q9Y3U8;J3QSB5	60S ribosomal protein L36	RPL36	-0.5117264	0.299917305	3.25E-37
P78344;D3DQV9;P78344-2;H	Eukaryotic translation initia	EIF4G2	0.5580158	0.301612139	1.10E-231
P46977;E9PIJ8;E9PN73;E9PI3	Dolichyl-diphosphooligosac	STT3A	1.068916	0.302697094	1.13E-07
Q9BRP8;Q9BRP8-2	Partner of Y14 and mago	WIBG	-0.7211151	0.305497495	7.18E-13
P68366;A8MUB1	Tubulin alpha-4A chain	TUBA4A	0.5521507	0.30670758	2.98E-228
Q92791;K7ERA3	Synaptonemal complex pro	LEPREL4	0.5498943	0.308682977	1.21E-234
O75521;O75521-2;F1LLU7;C9	Enoyl-CoA delta isomerase	ECI2	-0.7152481	0.30872166	1.95E-40
Q9HAF1;Q9HAF1-2;B1AK64;C	Chromatin modification-rel	MEAF6	-0.7138901	0.309471151	2.13E-11
Q8WWC4;H7C0V0	Uncharacterized protein C2	C2orf47	1.055689	0.309803882	0.00037694
P35268;K7ELC4;K7EMH1;K7E	60S ribosomal protein L22	RPL22	-0.5013695	0.30983543	1.67E-53
Q00341;C9J5E5;C9JIZ1;C9JM	Vigilin	HDLBP	1.052677	0.311437275	4.26E-15
Q9UBQ5;K7ES31;K7EMQ9;K7	Eukaryotic translation initia	EIF3K	1.050003	0.312892323	1.74E-25
Q07020;F8VUA6;F8VWC5;G3	60S ribosomal protein L18	RPL18	1.049175	0.31334365	7.19E-48
Q9HD45	Transmembrane 9 superfan	TM9SF3	1.048832	0.313530962	0.00041671
A6NKV8;Q9H074;D6REB4;Q9	Polyadenylate-binding prot	PAIP1	1.046799	0.314641776	2.56E-34
Q9Y5M8;H7C4H2	Signal recognition particle r	SRPRB	0.5418205	0.31582009	1.49E-193
Q06830	Peroxiredoxin-1	PRDX1	-0.494791	0.316246421	2.71E-22
Q14919;E9PQX9;Q14919-2;C	Dr1-associated corepressor	DRAP1	-0.6998138	0.317310509	1.04E-26

P27797;K7EJB9	Calreticulin	CALR	0.5401497	0.317310509	8.11E-93
P62258;P62258-2;K7EM20	14-3-3 protein epsilon	YWHAE	-0.4937077	0.317310509	4.73E-144
Q9P0L0;Q9P0L0-2	Vesicle-associated membra	VAPA	1.041933	0.317310509	8.71E-17
Q9H1C3	Glycosyltransferase 8 doma	GLT8D2	1.040302	0.318208279	8.27E-11
P40429;M0QYS1;Q6NVV1	60S ribosomal protein L13a;	RPL13A	1.037668	0.319661874	4.44E-09
Q13243;Q13243-3;B4DJK0;B4	Serine/arginine-rich splicing	SRSF5	-0.4900379	0.320932343	3.96E-32
Q01650	Large neutral amino acids tr	SLC7A5	1.032709	0.322410316	5.29E-15
P07910-4;P07910-2;G3V4W0	Heterogeneous nuclear ribo	HNRNPC;HNRNPCL1	-0.4838104	0.327140116	7.36E-89
Q5F1R6;Q5F1R6-3;Q5F1R6-2	DnaJ homolog subfamily C r	DNAJC21	-0.6802845	0.328400606	1.35E-42
Q03111	Protein ENL	MLLT1	0.5243511	0.331630705	6.16E-31
P46783;F6U211	40S ribosomal protein S10	RPS10	-0.4780521	0.33294907	7.29E-87
Q16629;Q16629-3;Q16629-2	Serine/arginine-rich splicing	SRSF7	-0.4777489	0.333256842	6.85E-42
Q96AG4	Leucine-rich repeat-contain	LRRC59	0.5220642	0.333737704	1.68E-147
P14866;P14866-2;M0QXS5;M	Heterogeneous nuclear ribo	HNRNPL	-0.4758148	0.335223931	1.94E-137
Q68CQ4	Digestive organ expansion f	DIEXF	0.5179729	0.337528598	5.15E-240
P62633;P62633-2;P62633-4;E	Cellular nucleic acid-binding	CNBP	-0.6608543	0.33968081	7.71E-83
O15446;O15446-2	DNA-directed RNA polymera	CD3EAP	-0.4714394	0.339701666	9.28E-131
P07355;H0YN42;P07355-2;H0	Annexin A2;Annexin;Putativ	ANXA2;ANXA2P2	-0.4691162	0.342094635	6.97E-133
P08651-4;P08651-3;P08651-2	Nuclear factor 1 C-type	NFIC	0.9978409	0.342168822	2.75E-18
Q9H1E3	Nuclear ubiquitous casein a	NUCKS1	0.5103111	0.344702027	9.92E-146
Q9NTK5;C9JTK6;Q9NTK5-3;J3	Obg-like ATPase 1	OLA1	-0.6510372	0.345473517	9.15E-12
Q8TDN6	Ribosome biogenesis protei	BRIX1	0.9896526	0.346918786	1.78E-13
P63173;J3KT73;J3QL01	60S ribosomal protein L38	RPL38	-0.4629269	0.348522475	1.39E-19
Q9UGP8	Translocation protein SEC63	SEC63	0.5051899	0.34955064	0
Q9UNF0;Q9UNF0-2	Protein kinase C and casein	PACSIN2	0.5045853	0.350125927	4.78E-157
Q13247;Q13247-3;Q13247-2	Serine/arginine-rich splicing	SRSF6	-0.6330833	0.356229229	1.33E-19
O60884	DnaJ homolog subfamily A r	DNAJA2	0.496727	0.357657411	8.91E-78
P45880;B4DKM5;P45880-2;P	Voltage-dependent anion-se	VDAC2	0.97118	0.357787989	4.76E-15
P38919	Eukaryotic initiation factor 4	EIF4A3	-0.6271172	0.359849655	7.14E-29
P62750;H7BY10;K7EJV9;K7ER	60S ribosomal protein L23a	RPL23A	-0.4493904	0.362845997	1.05E-71
Q4LE39;Q4LE39-2;Q4LE39-3	AT-rich interactive domain-c	ARID4B	0.9613914	0.363633419	3.28E-47
P14618;P14618-2;H3BT25;H3	Pyruvate kinase PKM;Pyruva	PKM	0.9611187	0.363797149	1.80E-10
Q9NZN4;B4DNU6	EH domain-containing prote	EHD2	0.9607162	0.364038821	2.39E-15
Q9Y333	U6 snRNA-associated Sm-lik	LSM2	0.9554672	0.367200052	2.72E-11
P09874	Poly [ADP-ribose] polymera	PARP1	0.4866123	0.367500338	3.74E-148
P60468	Protein transport protein Se	SEC61B	0.4865551	0.367556497	0.00010624
P27824;B4DGP8;B4E2T8	Calnexin	CANX	0.4850349	0.369050408	0
Q92945	Far upstream element-bind	KHSRP	-0.6098442	0.370460997	5.59E-55
O60763;O60763-2;F5H4X1;F5	General vesicular transport	USO1	0.4835014	0.370561261	3.13E-164
Q9H4L7;Q9H4L7-2;Q9H4L7-3	SWI/SNF-related matrix-ass	SMARCAD1	-0.6094246	0.370721176	1.35E-25
Q9H936;E9PJH7	Mitochondrial glutamate ca	SLC25A22	0.9485207	0.37140984	1.26E-18
Q05682;Q05682-3;Q05682-2	Caldesmon	CALD1	-0.6071911	0.372107945	1.54E-124
Q9UDW1	Cytochrome b-c1 complex s	UQCR10	0.9468384	0.37243383	9.25E-09
Q9NPE3	H/ACA ribonucleoprotein co	NOP10	-0.4402905	0.372679216	7.05E-29

Q92522	Histone H1x	H1FX	-0.4402027	0.372774821	3.18E-48
J3KN66;Q5JTV8;H0Y4R4;Q5JT	Torsin-1A-interacting protei	TOR1AIP1	0.4800835	0.373942529	4.87E-225
Q9BQG0;Q9BQG0-2;I3L1L3	Myb-binding protein 1A	MYBBP1A	0.9430122	0.374769275	1.63E-28
Q13445	Transmembrane emp24 dor	TMED1	-0.4383488	0.374798533	6.47E-26
Q16531;F5GY55	DNA damage-binding protei	DDB1	0.4786434	0.375372812	4.69E-136
P62277;J3KMX5	40S ribosomal protein S13	RPS13	-0.4377956	0.375403628	2.32E-20
Q9UHB9;F5H5Y3;G3V1U4;Q9	Signal recognition particle s	SRP68	0.478426	0.375589069	5.75E-268
Q9BWJ5	Splicing factor 3B subunit 5	SF3B5	-0.4361324	0.377226719	8.90E-44
Q96A26;F8W7Q4;E9PH05	Protein FAM162A	FAM162A	0.9371452	0.378367985	1.73E-05
Q13509;A8K854	Tubulin beta-3 chain	TUBB3	-0.5957375	0.379269847	2.14E-228
Q8IX15;F8WCA3	Homeobox and leucine zipp	HOMEZ	0.4724464	0.381566244	1.47E-38
Q8IVT2	Mitotic interactor and subst	MISP	-0.5918903	0.381694334	1.12E-22
Q15291;Q15291-2	Retinoblastoma-binding prc	RBBP5	0.9316864	0.38173537	1.78E-06
P63220;Q8WVC2;Q9BYK1	40S ribosomal protein S21	RPS21	-0.432003	0.381776633	4.58E-36
Q9Y2T2	AP-3 complex subunit mu-1	AP3M1	0.470665	0.383358228	1.31E-150
Q9UMS4;F5GY56	Pre-mRNA-processing facto	PRPF19	0.9290352	0.383377435	1.12E-94
Q7Z7H5;Q7Z7H5-3;Q7Z7H5-2	Transmembrane emp24 dor	TMED4	0.9278946	0.384085206	4.25E-41
P01834	Ig kappa chain C region	IGKC	-0.5874672	0.384493554	3.14E-267
P39019	40S ribosomal protein S19	RPS19	-0.4280357	0.386179477	2.48E-23
O00566;C9JX83	U3 small nucleolar ribonucl	MPHOSPH10	0.9241447	0.386417722	1.14E-09
P52739;P52739-2;D6RJH2;D6	Zinc finger protein 131	ZNF131	0.9146271	0.392376701	1.59E-37
P15880;H0YEN5;E9PQD7	40S ribosomal protein S2	RPS2	0.9111252	0.394583172	2.26E-13
Q15910;Q15910-2;G3XAL2;Q	Histone-lysine N-methyltrar	EZH2	0.9099083	0.395351662	2.24E-08
P16989;P16989-2;P16989-3	Y-box-binding protein 3	YBX3	-0.4192944	0.395989363	2.22E-127
Q9Y6E2;E7ETZ4;B5MCH7;B5M	Basic leucine zipper and W2	BZW2	0.4572716	0.396995122	4.50E-66
P62701	40S ribosomal protein S4, X	RPS4X	0.457098	0.397173746	6.32E-125
Q96DH6;Q96DH6-2;B4DHE8;	RNA-binding protein Musas	MSI2	-0.4179974	0.397457615	2.65E-69
Q9NWS0;M0QYF4;M0QXD5;I	PIH1 domain-containing prc	PIH1D1	-0.5664558	0.397961446	4.05E-42
P49755;G3V2K7	Transmembrane emp24 dor	TMED10	0.4547882	0.399555425	3.85E-53
Q9Y230;B3KQ59;M0R0Y3	RuvB-like 2	RUVBL2	0.4530697	0.401332987	1.39E-97
P61619;B4DR61;P61619-3	Protein transport protein Se	SEC61A1	0.8999443	0.401678092	2.00E-10
Q9UBV2	Protein sel-1 homolog 1	SEL1L	0.8952789	0.40466104	5.58E-22
Q9H501	ESF1 homolog	ESF1	0.8941994	0.405353172	4.13E-24
P0DI83	Ras-related protein Rab-34,	RAB34	-0.5548496	0.405521189	1.43E-19
P62304;A6NHK2	Small nuclear ribonucleoprc	SNRPE	-0.4107704	0.405698732	3.21E-75
Q9Y6M0;Q9Y6M0-2;I3L3C7;C	Testisin	PRSS21	0.88974	0.408219678	2.99E-69
P35251;P35251-2	Replication factor C subunit	RFC1	0.8888206	0.408812132	1.85E-45
O43660;O43660-2;A8MW61	Pleiotropic regulator 1	PLRG1	0.8876152	0.409589735	1.83E-47
P26368;K7ENG2;P26368-2	Splicing factor U2AF 65 kDa	U2AF2	-0.4044495	0.412989731	3.61E-96
O75459	P antigen family member 1	PAGE1	-0.5426102	0.413585636	2.67E-20
Q9NYF8-3;E9PK91;E9PK09;E9	Bcl-2-associated transcriptio	BCLAF1	0.8786106	0.415426152	1.35E-241
P07919	Cytochrome b-c1 complex s	UQCRH	-0.4022636	0.415528952	4.79E-120
P98175-2;P98175	RNA-binding protein 10	RBM10	-0.4015675	0.416339623	0
Q9Y2S0	DNA-directed RNA polymera	POLR1D	-0.5375919	0.416919351	6.23E-55

P13984	General transcription factor	GTF2F2	-0.4009914	0.417011076	2.53E-162
P45973;G3V1X9	Chromobox protein homolo	CBX5	-0.5337429	0.419487045	9.14E-38
Q9H2H8;B8ZZ77;Q9H2H8-2;H	Peptidyl-prolyl cis-trans isor	PPIL3	-0.3977547	0.420795964	2.36E-31
Q9Y3I0	tRNA-splicing ligase RtcB ho	C22orf28	-0.5301704	0.421878541	7.09E-94
Q14165	Malectin	MLEC	0.8665981	0.42328817	6.22E-142
P52655;J3KNC0	Transcription initiation facto	GTF2A1	-0.5241508	0.425926237	5.20E-25
Q16576;Q16576-2;E9PC52	Histone-binding protein RBI	RBBP7	0.4290676	0.42665242	9.71E-49
Q969V3;K7EQ66;K7ELZ9;K7EI	Nicalin	NCLN	0.8612061	0.426845348	4.06E-06
Q92688;Q92688-2;Q5T6W8	Acidic leucine-rich nuclear p	ANP32B	0.4269829	0.42889468	1.15E-162
Q02878;F8W181	60S ribosomal protein L6	RPL6	0.8576031	0.429231933	1.69E-11
Q9BRJ6;C9JQV0;H7C0T1;H7C	Uncharacterized protein C7	C7orf50	-0.5190125	0.429399259	2.57E-32
O94992	Protein HEXIM1	HEXIM1	0.8549156	0.43101712	2.89E-18
Q9NR50;H0Y580;Q9NR50-3;C	Translation initiation factor	EIF2B3	0.4244461	0.431632359	5.07E-48
Q14318;Q14318-2;J3KQ73	Peptidyl-prolyl cis-trans isor	FKBP8	0.4223328	0.433920786	5.58E-118
O15355	Protein phosphatase 1G	PPM1G	0.4207573	0.435631332	2.94E-192
Q9BQ15;C9JT95;C9JMP5	SOSS complex subunit B1	NABP2	-0.5084991	0.436556224	2.79E-39
P14678;P63162;P14678-2;B3	Small nuclear ribonucleopro	SNRPB;SNRPN	-0.3835716	0.437616893	1.61E-82
Q16181;H0Y3Y4;H0YFF6;Q16	Septin-7	SEPT7	-0.3825188	0.438880807	3.10E-53
O60885;O60885-2;Q4G0X8;N	Bromodomain-containing p	BRD4	0.4170704	0.439649513	3.46E-167
P62910;D3YTB1;F8W727	60S ribosomal protein L32	RPL32	-0.3802052	0.441665569	8.29E-104
Q99590;F8W028;Q99590-2;F	Protein SCAF11	SCAF11	0.4150658	0.441843151	1.12E-49
D6R9B1;Q15723-4;E9PCX3;Q	ETS-related transcription fa	ELF2	0.8386917	0.441885205	1.15E-07
P08238	Heat shock protein HSP 90-I	HSP90AB1	0.8369141	0.443085479	2.41E-128
F5GWP8		JUP	0.4138069	0.443223893	2.84E-100
P04843;B7Z4L4	Dolichyl-diphosphooligosac	RPN1	0.4133644	0.44370983	3.07E-248
Q8WXG6;Q8WXG6-3;Q8WXG	MAP kinase-activating deatl	MADD	0.8355446	0.444011423	1.29E-11
O94874;O94874-3;O94874-2	E3 UFM1-protein ligase 1	UFL1	0.8340244	0.445040539	2.99E-47
Q92733;A6NG79	Proline-rich protein PRCC	PRCC	0.4116173	0.445631414	3.65E-138
Q9BQE9;Q9BQE9-4;Q9BQE9-	B-cell CLL/lymphoma 7 prot	BCL7B	-0.4938641	0.446632624	1.49E-09
P63261;I3L3I4;I3L4N8;I3L3I0;	Actin, cytoplasmic 2;Actin, c	ACTG1	-0.3759995	0.446753554	0
P62899;H7C2W9;C9JU56;B7Z	60S ribosomal protein L31	RPL31	-0.3748627	0.448134514	3.54E-22
Q9UPP1;Q9UPP1-2;Q9UPP1-	Histone lysine demethylase	PHF8	0.8272991	0.449609727	4.62E-37
P60660;P60660-2;F8VZV5;F8	Myosin light polypeptide 6	MYL6	-0.373642	0.44962012	5.67E-26
Q15370;B8ZZU8;Q15370-2;I3	Transcription elongation fac	TCEB2	-0.4883766	0.450444631	1.36E-08
Q9UHD8;Q9UHD8-2;Q9UHD8	Septin-9	SEPT9	-0.3710365	0.452800282	7.21E-261
P46776;E9PJD9;E9PLL6	60S ribosomal protein L27a	RPL27A	-0.3694305	0.45476684	1.30E-19
Q15517;Q2L6G8;G8JLG2	Corneodesmosin	CDSN	0.8160229	0.457330193	0.0016978
Q13724;F5H6D0;C9J8D4	Mannosyl-oligosaccharide g	MOGS	0.3996849	0.458881342	2.23E-190
Q96JB5;F5H3I5;J3QQY1;Q96J	CDK5 regulatory subunit-as	CDK5RAP3	0.3983498	0.460377523	1.02E-63
O75643	U5 small nuclear ribonuclec	SNRNP200	0.398016	0.460751994	5.95E-137
Q14676;Q14676-2;E9PGY5;Q	Mediator of DNA damage cl	MDC1	0.3967876	0.462131513	6.00E-105
Q14974;B7ZAV6;F5H4R7;J3K	Importin subunit beta-1	KPNB1	0.3964272	0.462536809	0
Q13416	Origin recognition complex	ORC2	-0.4710064	0.462632074	4.26E-157
Q9NX40;D6RG39;D6RIT9;Q9N	OCIA domain-containing pro	OCIAD1	0.8075581	0.463174349	6.92E-49

Q08945	FACT complex subunit SSRP	SSRP1	0.3946247	0.46456626	2.63E-186
Q9BUA3	Uncharacterized protein C1	C11orf84	-0.4675407	0.465085497	2.57E-45
Q14151	Scaffold attachment factor I	SAFB2	-0.4664345	0.465870169	5.03E-49
P25490;H0YJV7	Transcriptional repressor pr	YY1	0.393362	0.465990896	5.71E-62
P33993;P33993-3	DNA replication licensing fa	MCM7	0.3927002	0.466738614	2.46E-138
P49411	Elongation factor Tu, mitocl	TUFM	0.3921509	0.467359704	4.41E-124
O75179;O75179-5;O75179-3;	Ankyrin repeat domain-con	ANKRD17	0.7980881	0.469761528	3.31E-34
Q96N66;Q96N66-3;Q96N66-2	Lysophospholipid acyltransf	MBOAT7	0.7973404	0.470283799	4.65E-14
O75400;O75400-2;O75400-3	Pre-mRNA-processing facto	PRPF40A	-0.4599838	0.47046023	8.34E-22
Q96RE7	Nucleus accumbens-associa	NACC1	0.3889961	0.470935514	2.38E-105
Q13185	Chromobox protein homolo	CBX3	-0.3555202	0.472000151	1.27E-108
Q86XP3;Q86XP3-2	ATP-dependent RNA helicas	DDX42	0.3857613	0.474617748	3.56E-226
P26373;J3QSB4;P26373-2	60S ribosomal protein L13	RPL13	-0.3520393	0.476368235	8.82E-39
P62318;B4DJP7;H3BT13	Small nuclear ribonucleopro	SNRPD3	-0.3506012	0.478179357	7.47E-79
P68363	Tubulin alpha-1B chain	TUBA1B	0.3816471	0.479323626	2.17E-292
P16070;H0YE40;H0YD13;H0Y	CD44 antigen	CD44	-0.4464474	0.480172978	6.32E-31
P27816;P27816-6;E7EVA0;P2	Microtubule-associated pro	MAP4	-0.3485279	0.480796961	1.24E-60
P42704	Leucine-rich PPR motif-cont	LRPPRC	0.3777676	0.483784384	2.28E-186
P55081	Microfibrillar-associated pro	MFAP1	0.3760948	0.485714656	2.29E-87
P55199	RNA polymerase II elongatio	ELL	0.7749596	0.486064876	2.98E-40
O43290	U4/U6.U5 tri-snRNP-associa	SART1	-0.4379101	0.486354471	5.29E-94
Q04917;A2IDB2	14-3-3 protein eta	YWHAH	-0.4377747	0.486452869	1.19E-31
P40939	Trifunctional enzyme subun	HADHA	0.3736839	0.488504029	0
P55209;F8VUX1;F8VY35;B3KI	Nucleosome assembly prote	NAP1L1	0.3735828	0.488621177	6.97E-227
P08574	Cytochrome c1, heme prote	CYC1	0.3722477	0.490169832	2.73E-44
Q6P1N0;Q6P1N0-2;K7EJY5;K7	Coiled-coil and C2 domain-c	CC2D1A	0.7605152	0.496399933	1.95E-10
Q15428;K7EP23;K7EMT0;K7E	Splicing factor 3A subunit 2	SF3A2	-0.4230423	0.497221518	1.97E-54
Q16649	Nuclear factor interleukin-3	NFIL3	-0.4200172	0.499448298	3.34E-23
Q03701	CCAAT/enhancer-binding pr	CEBPZ	0.7559433	0.499695426	5.46E-58
O43395;E7EVD1	U4/U6 small nuclear ribonu	PRPF3	0.7552013	0.500231332	1.07E-49
P68133;P68032;P63267;P627	Actin, alpha skeletal muscle	ACTA1;ACTC1;ACTG	-0.3328114	0.500891196	3.95E-86
Q8IWA0	WD repeat-containing prote	WDR75	0.7537346	0.501291642	4.72E-113
Q5VT52;Q5VT52-3;Q5VT52-2	Regulation of nuclear pre-m	RPRD2	0.3625031	0.501552315	5.14E-160
P98175-3;P98175-4	RNA-binding protein 10	RBM10	-0.4162312	0.502242727	2.86E-151
Q96EY4;D6RA57;H0Y9X1	Translation machinery-asso	TMA16	0.7494965	0.50436205	7.15E-72
P84098;J3QL15;J3QR09;J3KTI	60S ribosomal protein L19;F	RPL19	0.3595467	0.505033069	3.38E-29
Q15366;Q15366-4;Q15366-5	Poly(rC)-binding protein 2;P	PCBP2;PCBP3	-0.3295708	0.505089072	7.48E-40
Q9H4L4;J3KNH7	Sentrin-specific protease 3	SENP3	0.7462769	0.506701178	2.26E-09
Q9UHB7;Q9UHB7-2	AF4/FMR2 family member 4	AFF4	0.3565254	0.508603282	2.93E-114
Q8IYB3;A9Z1X7;Q8IYB3-2;E9I	Serine/arginine repetitive m	SRRM1	-0.3244514	0.511758259	2.23E-66
P62888;E5RI99	60S ribosomal protein L30	RPL30	-0.3233414	0.513210448	1.35E-97
P09234	U1 small nuclear ribonucleo	SNRPC	-0.3232555	0.51332282	2.65E-66
O75607	Nucleoplasmin-3	NPM3	0.3525333	0.513340954	4.58E-225
P51648;P51648-2;J3QRD1;J3	Fatty aldehyde dehydrogen	ALDH3A2	0.7362061	0.514054535	1.55E-19

Q92841;Q92841-3;Q92841-1	Probable ATP-dependent RI	DDX17	0.7335491	0.516003752	1.26E-81
P22695;H3BRG4;H3BSJ9;H3B	Cytochrome b-c1 complex s	UQCRC2	0.3493214	0.517169438	1.96E-119
O00483	NADH dehydrogenase [ubiq	NDUFA4	0.3478298	0.518952287	3.15E-08
Q15061	WD repeat-containing prote	WDR43	0.3454933	0.52175148	7.67E-97
Q99714;Q99714-2;Q5H928	3-hydroxyacyl-CoA dehydro	HSD17B10	-0.3165398	0.522154863	1.06E-255
Q9Y265;Q9Y265-2;E7ETR0;H7	RuvB-like 1	RUVBL1	0.3441086	0.523414088	2.13E-135
Q9P287;Q9P287-3;Q9P287-2	BRCA2 and CDKN1A-interac	BCCIP	-0.3863468	0.524586765	2.67E-84
P30419;O60551;B4DXS1;P30	Glycylpeptide N-tetradecan	NMT1;NMT2	-0.3860359	0.524821866	2.26E-05
P55039;A8MZF9;J3QR71;J3Q	Developmentally-regulated	DRG2	0.7204056	0.525702381	4.09E-16
P62273;P62273-2	40S ribosomal protein S29	RPS29	-0.384407	0.526054502	3.53E-07
Q14247;Q14247-3;Q14247-2	Src substrate cortactin	CTTN	-0.3134727	0.526214182	3.71E-62
Q99613;H3BRV0;B5ME19	Eukaryotic translation initia	EIF3C;EIF3CL	0.7196941	0.526230001	5.34E-144
Q96JJ7	Protein disulfide-isomerase	TMX3	0.718668	0.526991495	2.33E-15
Q99848;H7C2Q8	Probable rRNA-processing p	EBNA1BP2	-0.3105545	0.53009151	5.30E-58
P82673;P82673-2;H0YG82	28S ribosomal protein S35,	MRPS35	-0.3765926	0.531988488	3.63E-22
Q15424;B7ZLP6;A0AV56;Q15	Scaffold attachment factor I	SAFB	-0.3079376	0.533580734	0
Q66PJ3;Q66PJ3-2;Q66PJ3-3;F	ADP-ribosylation factor-like	ARL6IP4	-0.3078003	0.533764162	4.60E-114
Q86X53;E5RHA3;H0YB25;H0Y	Glutamate-rich protein 1	ERICH1	0.7090263	0.534173785	1.57E-28
Q9Y6A4	UPF0468 protein C16orf80	C16orf80	0.3348827	0.534560239	2.85E-147
J3KPD9;P22392-2;Q32Q12;P2	Nucleoside diphosphate kin	NME2;NME1-NME2	-0.3067551	0.535161297	8.59E-55
P08708;P0CW22;H0YN88;H0Y	40S ribosomal protein S17;4	RPS17;RPS17L	-0.3061371	0.535988206	1.49E-29
O43670;J3KS31;O43670-3;O4	Zinc finger protein 207	ZNF207	-0.3709793	0.53627188	2.11E-07
Q8IXQ4;Q8IXQ4-3;Q8IXQ4-4;	Uncharacterized protein KIA	KIAA1704	-0.3670559	0.539276013	1.26E-05
O60832;H7C2Q9;H7C0M1;C9	H/ACA ribonucleoprotein co	DKC1	0.7021179	0.539350247	2.74E-22
P20042	Eukaryotic translation initia	EIF2S2	-0.3030014	0.540193894	1.21E-137
Q8IWX8;J3QK89	Calcium homeostasis endop	CHERP	0.7009811	0.540204435	2.53E-45
P50454;E9PKH2;E9PR70;E9PI	Serpin H1	SERPINH1	-0.365778	0.540256328	1.79E-14
Q12888;C9JXV0;A6NNK5;F8V	Tumor suppressor p53-bind	TP53BP1	0.3294125	0.541225223	4.98E-165
P06493;P06493-2	Cyclin-dependent kinase 1	CDK1	-0.3642673	0.541416296	2.43E-21
O76094;O76094-2	Signal recognition particle s	SRP72	0.3285542	0.542274738	0
Q7L0Y3;C9JVB6	Mitochondrial ribonuclease	TRMT10C	-0.301384	0.542369687	4.07E-170
O43852;O43852-3;O43852-6	Calumenin	CALU	-0.3013496	0.542415918	3.00E-153
P17028;K7EQP8;K7EPZ8;K7EI	Zinc finger protein 24	ZNF24	-0.3621178	0.543069048	4.13E-12
P37108;H0YLA2	Signal recognition particle 1	SRP14	-0.2991829	0.545337614	4.99E-123
Q99933;Q99933-4;Q99933-3	BAG family molecular chape	BAG1	-0.3583069	0.54600529	1.21E-12
Q8WXI9	Transcriptional repressor p6	GATAD2B	-0.3568726	0.547112457	1.65E-19
P13861;Q9BUB1;H7C1L0	cAMP-dependent protein ki	PRKAR2A	0.6916752	0.54722237	1.36E-52
Q13112	Chromatin assembly factor	CHAF1B	-0.3565826	0.547336381	2.72E-56
P41208	Centrin-2	CETN2	-0.3559799	0.547802052	7.85E-13
P06730;D6RBW1;P06730-3;P	Eukaryotic translation initia	EIF4E	0.3235378	0.548428799	7.92E-24
Q9NXS2	Glutaminyl-peptide cyclotra	QPCTL	0.3232746	0.548752661	1.21E-78
Q13547;F5GXM1	Histone deacetylase 1	HDAC1	-0.2959213	0.549750265	1.08E-119
Q15365	Poly(rC)-binding protein 1	PCBP1	-0.2950459	0.550937702	9.94E-46
Q8WX92	Negative elongation factor I	NELFB	-0.3504009	0.552121733	3.41E-29

P50402;Q5HY57	Emerin	EMD	-0.3500404	0.552401425	6.21E-73
O14979;O14979-3;O14979-2	Heterogeneous nuclear ribo	HNRPDL	-0.2936497	0.552833999	9.40E-53
O15427	Monocarboxylate transport	SLC16A3	0.6812973	0.555101522	0.00033088
O00629	Importin subunit alpha-3	KPNA4	0.315279	0.558635321	2.96E-53
Q9C005;B4DIS3	Protein dpy-30 homolog	DPY30;LOC84661	-0.3407822	0.559608356	0.0016526
P22087;M0R299;M0QXL5;M0	rRNA 2-O-methyltransferase	FBL	-0.2877579	0.560871346	1.21E-57
P19388	DNA-directed RNA polymera	POLR2E	-0.2854652	0.564014077	2.81E-154
P61981	14-3-3 protein gamma;14-3	YWHAG	-0.3345909	0.564453138	2.32E-107
P53618;E9PP73	Coatomer subunit beta	COPB1	0.6672325	0.565867561	3.86E-35
P08865;C9J9K3;A6NE09	40S ribosomal protein SA	RPSA;RPSAP58	-0.2835312	0.566671845	5.91E-81
P06748;P06748-2;P06748-3	Nucleophosmin	NPM1	0.3060036	0.570207127	0
P62851	40S ribosomal protein S25	RPS25	-0.2807751	0.570469642	1.80E-11
Q15554;H3BR06;H3BTA7;Q15	Telomeric repeat-binding fa	TERF2	-0.3266506	0.570695974	1.16E-57
P38432	Coilin	COIL	0.3051357	0.571295649	9.16E-65
P55084;B4E2W0;B5MD38;F5	Trifunctional enzyme subun	HADHB	0.3046761	0.571872605	1.55E-103
P50914;E7EPB3	60S ribosomal protein L14	RPL14	0.3044128	0.572203103	2.63E-24
Q8WVV9;Q8WVV9-4;D6W59	Heterogeneous nuclear ribo	HNRNPLL;HNRPLL	-0.3237057	0.573019687	1.45E-28
Q9BQE3;F5H5D3	Tubulin alpha-1C chain	TUBA1C	0.3028107	0.574216786	5.84E-259
P31930	Cytochrome b-c1 complex s	UQCRC1	0.3006477	0.576940567	4.55E-255
Q9NQW6;Q9NQW6-2	Actin-binding protein anillin	ANLN	-0.3178024	0.577691118	3.76E-56
Q9Y6N8	Cadherin-10	CDH10	0.6515903	0.577957521	0.0012038
P55072	Transitional endoplasmic re	VCP	-0.2740707	0.579758167	2.24E-219
O15533;O15533-2;D3YTI9;A8	Tapasin	TAPBP	0.298233	0.579988577	2.00E-14
C9J3L7;C9JZE3;C9JYJ8;Q9BW	Replication initiator 1	REPIN1	-0.3142948	0.580475271	2.67E-22
P55347;P55347-2;E7EPN6;H0	Homeobox protein PKNOX1	PKNOX1	0.2976341	0.580745727	7.97E-21
Q14694;Q14694-3;Q14694-2	Ubiquitin carboxyl-terminal	USP10	0.2975636	0.580834976	7.50E-65
Q13144;E9PC74	Translation initiation factor	EIF2B5	0.6450272	0.583066204	9.50E-150
P46100;P46100-5;P46100-3;P	Transcriptional regulator AT	ATRX	0.6423302	0.585171587	1.82E-37
Q12899;A2AE48	Tripartite motif-containing p	TRIM26	0.293005	0.586613522	1.72E-75
Q9NRP0;D6RH22	Oligosaccharyltransferase c	OSTC	0.6395111	0.587376032	0.00023698
Q9BZZ5;Q9BZZ5-2;Q9BZZ5-3;	Apoptosis inhibitor 5	API5	-0.3026924	0.589729118	1.19E-46
O43143	Putative pre-mRNA-splicing	DHX15	0.6309528	0.594091849	9.46E-11
P30508;P30505;E7ERM2;B0S	HLA class I histocompatibilit	HLA-C	0.2860069	0.595535907	2.59E-205
O75475;O75475-2;O75475-3	PC4 and SFRS1-interacting p	PSIP1	-0.2615509	0.597291143	1.44E-180
Q9NP74;Q9NP74-2;Q9NP74-3	Palmdelphin	PALMD	-0.2601852	0.599218133	7.52E-74
P49321;P49321-2;P49321-3;(	Nuclear autoantigenic sperr	NASP	0.2830181	0.599365359	1.32E-103
Q969S3	Zinc finger protein 622	ZNF622	-0.2596645	0.599953607	1.21E-82
Q9BT78;Q9BT78-2;D6RAX7;D	COP9 signalosome complex	COPS4	0.6225662	0.600706821	2.06E-42
P34932	Heat shock 70 kDa protein 4	HSPA4	0.6216927	0.601397759	2.31E-22
P51571;H7C1C6;A6NLM8	Translocon-associated prote	SSR4	0.2799664	0.603286924	6.55E-127
P49643	DNA primase large subunit	PRIM2	0.6169701	0.605139244	4.55E-11
Q8N4Q1;Q8N4Q1-2	Mitochondrial intermembra	CHCHD4	-0.2542915	0.607566452	2.83E-76
P53999	Activated RNA polymerase I	SUB1	-0.2538223	0.6082333	3.67E-38
Q01130;J3QL05;J3KP15;B4DN	Serine/arginine-rich splicing	SRSF2;SFRS2	-0.2533836	0.608857071	3.45E-26

Q9BUF5;K7ESM5	Tubulin beta-6 chain	TUBB6	-0.2774086	0.610126878	9.56E-83
O43847;O43847-2;B1AKJ5;H(	Nardilysin	NRD1	0.6105423	0.610248342	3.04E-21
Q8WU90;B4DMW2	Zinc finger CCCH domain-co	ZC3H15	-0.2766457	0.610747224	1.36E-51
Q01658	Protein Dr1	DR1	-0.2511501	0.612037283	6.63E-17
Q99832;F8WAM2;Q99832-3	T-complex protein 1 subuni	CCT7	0.6055107	0.614261	2.86E-17
P46781;B5MCT8;C9JM19	40S ribosomal protein S9	RPS9	0.6046066	0.614983231	5.68E-20
Q9Y5U2;E9PL88;C9JDU0;A6N	Protein TSSC4	TSSC4	-0.271431	0.614994757	0.0025834
Q9BUL8;C9J5C3;C9J363;C9J6	Programmed cell death pro	PDCD10	-0.2481594	0.616307137	3.01E-58
P62753;A2A3R7;A2A3R5	40S ribosomal protein S6	RPS6	0.2696705	0.616601605	2.10E-13
Q9NPA8;G3V117;E5RHX8	Enhancer of yellow 2 transc	ENY2	-0.2478867	0.616697193	2.20E-40
P63000;P15153;P60763;P63(	Ras-related C3 botulinum to	RAC1;RAC2;RAC3	0.6013947	0.617552147	0.00035271
P52434;C9JBJ6;C9JLU1;C9JCL	DNA-directed RNA polymer	POLR2H	-0.2678013	0.6179589	1.17E-39
Q8WYP5;Q8WYP5-2;Q8WYP5	Protein ELYS	AHCTF1	0.2663021	0.620985427	1.14E-119
P10599;P10599-2	Thioredoxin	TXN	-0.2633133	0.621632583	8.70E-10
P19338;H7BY16	Nucleolin	NCL	-0.2440872	0.622141914	0
Q8NI22;Q8NI22-2;Q8NI22-3	Multiple coagulation factor	MCFD2	0.2642956	0.623603278	1.97E-90
P61513;C9J4Z3;M0R0A1;Q6P	60S ribosomal protein L37a	RPL37A	-0.2425632	0.624331661	1.96E-77
P31689;B7Z5C0	DnaJ homolog subfamily A r	DNAJA1	0.2633743	0.624806798	3.60E-46
P63010;Q7Z451;P63010-2;K7	AP-2 complex subunit beta;	AP2B1;AP1B1	-0.2421265	0.624959874	6.28E-93
Q99583	Max-binding protein MNT	MNT	-0.2591152	0.625077511	2.78E-58
Q9ULU4;B7ZM62;H7C4X9;Q9	Protein kinase C-binding pro	ZMYND8	0.5884628	0.627942089	2.70E-89
Q9H211;H3BSY1	DNA replication factor Cdt1	CDT1	0.587925	0.628375858	3.84E-23
Q3ZCQ8;Q3ZCQ8-2;M0R0C3;	Mitochondrial import inner	TIMM50	0.5865345	0.62949779	2.06E-14
Q8IVL5;D3DNV8	Prolyl 3-hydroxylase 2	LEPREL1	0.2583141	0.631435219	7.59E-97
P62316;P62316-2	Small nuclear ribonucleopro	SNRPD2	-0.2375717	0.631527018	7.85E-110
P40855;B7Z6I5;Q5QNY5;P40	Peroxisomal biogenesis fact	PEX19;DCAF8	-0.2511101	0.631669208	3.57E-287
Q9H334;G5E965;H0Y882;Q9H	Forkhead box protein P1	FOXP1	0.5824089	0.63283168	7.87E-67
P62263	40S ribosomal protein S14	RPS14	-0.2366066	0.632922314	3.61E-66
Q9Y2X3	Nucleolar protein 58	NOP58	0.2551498	0.635595326	4.58E-37
Q8N556;Q8N556-2	Actin filament-associated p	AFAP1	0.2540302	0.637070054	2.11E-99
Q9NS69	Mitochondrial import recep	TOMM22	0.2538223	0.637344055	1.08E-86
P02545;P02545-3;D6RAQ3;P(	Prelamin-A/C;Lamin-A/C	LMNA	0.2534008	0.63789975	9.65E-238
P68371	Tubulin beta-4B chain	TUBB4B	0.2533207	0.63800538	0
Q5SSJ5;B0QZK4;Q5SSJ5-3;Q5	Heterochromatin protein 1-	HP1BP3	0.5759315	0.638081087	7.45E-08
P32519;P32519-2	ETS-related transcription fa	ELF1	0.2514782	0.640436899	2.09E-89
Q9UKV3;E7EQT4;Q9UKV3-5	Apoptotic chromatin conde	ACIN1	0.2511253	0.640903005	0
P49959;P49959-2;F8W7U8;B	Double-strand break repair	MRE11A	-0.2394028	0.641362232	3.90E-24
Q9UNF1;Q5H909;Q9UNF1-2;	Melanoma-associated antig	MAGED2	0.5713787	0.641781732	1.47E-86
P46782;M0R0F0;M0QZN2;M(	40S ribosomal protein S5;4(	RPS5	-0.228199	0.64513223	5.39E-128
P46778;G3V1B3;M0R181	60S ribosomal protein L21	RPL21	-0.2274857	0.646172667	1.21E-33
P05089;P05089-2;P05089-3	Arginase-1	ARG1	-0.2327805	0.646872488	1.60E-25
E5RGW4		NPM1	0.2464828	0.647048574	1.46E-47
Q13435;E9PPJ0;E9PJ04;H0YC	Splicing factor 3B subunit 2	SF3B2	0.2462597	0.647344597	4.12E-149
Q9GZS3;H0YL19;H0YN81;H3E	WD repeat-containing prote	WDR61	0.2449188	0.649124438	6.05E-103

Q92667;I3L0W0;Q92667-2	A-kinase anchor protein 1, r	AKAP1	0.5609322	0.650306486	4.28E-08
Q8TBX8;Q8TBX8-2;Q8TBX8-3	Phosphatidylinositol 5-phos	PIP4K2C	-0.2270107	0.651689179	0.0004405
Q9Y3A6;M0R072;B1AKT3;B1	Transmembrane emp24 doi	TMED5	0.5584869	0.652308554	9.08E-31
Q9BRK5;Q9BRK5-6;Q9BRK5-2	45 kDa calcium-binding prot	SDF4	-0.2229691	0.652776249	1.52E-37
E9PAV3;F8VZJ2;F8W0W4;H0	Nascent polypeptide-associ	NACA	-0.2249374	0.653423572	2.70E-39
Q8WWI1-3;E9PMS6;Q8WWI	LIM domain only protein 7	LMO7	-0.2218475	0.654420242	1.40E-265
Q15233;C9JYS8;Q15233-2;C9	Non-POU domain-containin	NONO	-0.2210045	0.655657137	1.05E-51
P35606;B4DZI8	Coatomer subunit beta	COPB2	0.5526848	0.657069184	3.33E-29
P60866;P60866-2;G3XAN0	40S ribosomal protein S20	RPS20	-0.219162	0.658363683	4.17E-20
Q16656;Q96AN2;Q16656-2;(	Nuclear respiratory factor 1	NRF1	-0.219101	0.658453417	7.63E-26
Q15185;B4DP11;B4DP21	Prostaglandin E synthase 3	PTGES3	0.2375221	0.658978495	6.43E-52
O43707;D6PXK4;F5GXS2	Alpha-actinin-4	ACTN4	0.2371426	0.659485776	2.96E-214
Q14157;Q14157-1;Q14157-4	Ubiquitin-associated protei	UBAP2L	-0.2177773	0.660400748	0
Q15029;Q15029-2;K7EJ81	116 kDa U5 small nuclear ri	EFTUD2	0.2361412	0.660824832	4.62E-152
Q15019;B5MCX3;Q15019-2;(	Septin-2	SEPT2	-0.2141933	0.662441309	6.66E-48
Q8NAV1	Pre-mRNA-splicing factor 3	PRPF38A	-0.2161255	0.662833929	1.16E-134
Q16643-2;Q16643;A8MV58;[	Drebrin	DBN1	-0.2109184	0.665199852	1.68E-44
Q9H2P0	Activity-dependent neuropr	ADNP	-0.2140694	0.665867738	2.33E-77
Q12906;Q12906-4;Q12906-5	Interleukin enhancer-bindin	ILF3	-0.2121696	0.668675645	3.03E-168
Q5XKP0;K7EIR2;K7EKR0	Protein QIL1	QIL1;C19orf70	0.5382748	0.66895253	1.83E-20
P62847;P62847-2;E7ETK0;P6	40S ribosomal protein S24	RPS24	-0.2118568	0.669138437	3.37E-17
O15391	Transcription factor YY2	YY2	0.5378208	0.669328253	6.29E-29
P25705;K7ERX7;P25705-2;K7	ATP synthase subunit alpha	ATP5A1	0.2291451	0.670210479	1.89E-89
O14579;O14579-2;M0QXB4;I	Coatomer subunit epsilon	COPE	0.2265778	0.673667664	2.89E-75
Q15293;B7Z1M1	Reticulocalbin-1	RCN1	-0.2082138	0.67453749	9.32E-253
P19387	DNA-directed RNA polymer	POLR2C	-0.1997509	0.674640469	3.12E-24
Q8N6N3;Q8N6N3-2	UPF0690 protein C1orf52	C1orf52	-0.1992683	0.675049577	3.64E-83
Q9Y3B4	Pre-mRNA branch site prote	SF3B14	-0.1990223	0.67525821	1.07E-26
O60684;F5H4G7;F5GYL8;O15	Importin subunit alpha-7;Im	KPNA6;KPNA5	0.2253799	0.675283041	1.20E-125
P63208;E5RJR5;E5RGM3;E7E	S-phase kinase-associated p	SKP1	-0.2073841	0.675769475	1.28E-89
Q9NVA2;D6RGI3;D6RER5;Q9	Septin-11	SEPT11	-0.1964149	0.677470609	5.18E-42
P07437;Q5JP53;F8VYX6;F8V	Tubulin beta chain	TUBB	0.2233543	0.67801818	0
P26196	Probable ATP-dependent RI	DDX6	-0.194397	0.679184824	2.45E-78
P62306;F8W0W6	Small nuclear ribonucleopro	SNRPF	-0.2042351	0.68045324	1.66E-50
P32119;A6NIW5	Peroxiredoxin-2	PRDX2	-0.1914654	0.681678092	1.08E-14
Q14966;Q14966-3;Q14966-5	Zinc finger protein 638	ZNF638	0.2198544	0.682754148	2.13E-154
P42677;Q5T4L4	40S ribosomal protein S27	RPS27	-0.1899223	0.682991823	1.23E-15
O60341;O60341-2;F6S0T5	Lysine-specific histone dem	KDM1A	-0.1889305	0.683836757	6.32E-107
Q9NZI7;Q9NZI7-4	Upstream-binding protein 1	UBP1	-0.185009	0.687181362	6.98E-44
Q92541	RNA polymerase-associated	RTF1	-0.1844826	0.687630814	9.76E-27
Q9HC35;B5MCW9;B5MBZ0;F	Echinoderm microtubule-as	EML4	-0.1841965	0.687875128	1.03E-17
Q15459;E9PAW1	Splicing factor 3A subunit 1	SF3A1	-0.183754	0.688253064	1.00E-67
Q9NWV8;M0QY17;Q9NWV8-	BRISC and BRCA1-A comple	BABAM1	-0.1826572	0.689190095	6.66E-62
Q9Y232;Q9Y232-2;Q9Y232-3	Chromodomain Y-like prote	CDYL	-0.1813889	0.690274387	1.17E-27

Q13428;Q13428-3;Q13428-2	Treacle protein	TCOF1	0.2136574	0.691170133	2.11E-284
O15156;O15156-2	Zinc finger and BTB domain-	ZBTB7B	-0.179287	0.692072612	2.41E-12
P29692;E9PK01;P29692-2;P2	Elongation factor 1-delta	EEF1D	-0.1943417	0.695248035	3.58E-112
P62879;C9JIS1;C9JXA5;C9JZN	Guanine nucleotide-binding	GNB2	-0.1683807	0.701430845	1.35E-26
Q07021;I3L3Q7;I3L3B0	Complement component 1	C1QBP	-0.1900749	0.701664989	1.01E-231
Q9UI10;E7ERK9;F8W8L6	Translation initiation factor	EIF2B4	0.4988422	0.701889949	8.36E-58
O95365	Zinc finger and BTB domain-	ZBTB7A	0.4979591	0.702634327	3.46E-74
O95049;F5H2X0;K7ESC0;F5H	Tight junction protein ZO-3	TJP3	0.4961472	0.704162562	0.0012097
Q12800;Q12800-4;F8VWL0;C	Alpha-globin transcription f	TFCP2	-0.1879387	0.704885813	1.86E-60
P41091;Q2VIR3;Q2VIR3-2;F8	Eukaryotic translation initia	EIF2S3;EIF2S3L	0.2022114	0.706814808	1.71E-33
O60841	Eukaryotic translation initia	EIF5B	0.2020473	0.707039932	0
Q9NQZ2	Something about silencing p	UTP3	0.4919624	0.707696539	1.54E-119
O43447;C9JQD4;A6NM32;H0	Peptidyl-prolyl cis-trans isor	PPIH	-0.1581135	0.710282421	6.60E-07
Q01664;I3L254	Transcription factor AP-4	TFAP4	-0.183712	0.711274009	0
Q96RL1;Q96RL1-3;F8VQY2;Q	BRCA1-A complex subunit R	UIMC1	-0.15555	0.712498604	6.82E-50
Q96D15	Reticulocalbin-3	RCN3	-0.182869	0.712550634	1.66E-169
P11021	78 kDa glucose-regulated pr	HSPA5	-0.1811752	0.715117872	0
Q96QA5;J3KRG2	Gasdermin-A	GSDMA	-0.1520653	0.715515122	5.90E-28
O43823	A-kinase anchor protein 8	AKAP8	0.4812832	0.716743533	4.39E-21
P09661;H0YMA0;H0YLR3	U2 small nuclear ribonucleo	SNRPA1	-0.1797256	0.717317621	1.78E-144
O43491;E9PHY5;E9PPD9;O43	Band 4.1-like protein 2	EPB41L2	0.1933327	0.719036732	4.72E-100
P01040;C9J0E4	Cystatin-A;Cystatin-A, N-ter	CSTA	-0.1468849	0.720007658	1.01E-36
O43865;O43865-2;Q96HN2;C	Putative adenosylhomocyst	AHCYL1;AHCYL2	-0.1448536	0.721771927	1.51E-11
Q13283;F5H4D6	Ras GTPase-activating prote	G3BP1	-0.1731148	0.72737914	4.72E-194
Q15059;Q15059-2	Bromodomain-containing p	BRD3	0.1854286	0.729977901	0
Q01105-2;Q01105-3;Q5VXV2	Protein SET	SET	-0.1351833	0.730191004	0
Q92896;Q92896-2;H3BM42;C	Golgi apparatus protein 1	GLG1	0.4648018	0.730783804	4.22E-28
P13010	X-ray repair cross-complem	XRCC5	0.464716	0.730857164	1.51E-54
Q9H0E9-2;Q9H0E9;H7C128;H	Bromodomain-containing p	BRD8	0.4645233	0.731021823	2.46E-56
Q9NYF8;Q9NYF8-2;Q9NYF8-4	Bcl-2-associated transcriptio	BCLAF1	-0.170372	0.73156749	4.46E-247
O00303;H0YDT6;B4DEW9;B3	Eukaryotic translation initia	EIF3F	-0.1333313	0.731807168	9.97E-13
P11142;E9PKE3;P11142-2;E9	Heat shock cognate 71 kDa	HSPA8	-0.1691494	0.73343707	2.81E-235
Q13263;Q13263-2;M0R0K9	Transcription intermediary f	TRIM28	-0.1307793	0.734036128	8.18E-59
Q8N9T8;H0YFD2;D3YTE0;Q8N	Protein KRI1 homolog	KRI1	-0.1306553	0.734144468	3.43E-94
P39687;H0YN26	Acidic leucine-rich nuclear p	ANP32A	0.1798382	0.737749929	1.06E-182
Q9BUJ2;Q9BUJ2-4;B7Z4B8;Q	Heterogeneous nuclear ribo	HNRNPUL1	0.179718	0.737917282	1.85E-215
P25398	40S ribosomal protein S12	RPS12	-0.1661968	0.737958567	3.57E-65
P15924;P15924-2	Desmoplakin	DSP	0.1795502	0.738151065	1.77E-227
O75925;B3KSY9	E3 SUMO-protein ligase PIA	PIAS1	0.4558144	0.738478594	6.88E-29
P52597	Heterogeneous nuclear ribo	HNRNPF	0.1790733	0.738815355	2.38E-218
Q9Y605	MORF4 family-associated pr	MRFAP1	-0.1652622	0.73939169	2.74E-91
P31151;Q86SG5	Protein S100-A7;Protein S10	S100A7;S100A7A	-0.1234493	0.740450558	1.26E-08
Q92572;F5H459	AP-3 complex subunit sigma	AP3S1	-0.1640377	0.741270743	1.68E-79
P23497;P23497-4;P23497-2;P	Nuclear autoantigen Sp-100	SP100	0.4514961	0.742185143	3.55E-98

P17693;Q5RJ85	HLA class I histocompatibilit	HLA-G	0.4498177	0.743627473	1.26E-09
Q6QNY0	Biogenesis of lysosome-rela	BLOC1S3	-0.1163673	0.746665195	4.09E-20
Q9Y2D5-4;C9JVY5;Q9Y2D5;Q	A-kinase anchor protein 2	AKAP2	-0.1162395	0.746777489	1.73E-80
O14929;O14929-2	Histone acetyltransferase ty	HAT1	0.4433899	0.749159221	0.00014558
P12956;B1AHC9;B1AHC8;F5H	X-ray repair cross-complem	XRCC6	0.1704025	0.750928353	1.40E-99
Q9Y2W2;F5H721	WW domain-binding protei	WBP11	-0.1571178	0.751918159	0
P14316;D6RB34;D6R9N5;D6R	Interferon regulatory factor	IRF2;IRF1	-0.1070766	0.754843025	6.84E-25
P06702	Protein S100-A9	S100A9	0.4365559	0.755054784	2.64E-25
Q9Y295	Developmentally-regulated	DRG1	-0.1546211	0.755771541	3.27E-179
Q53F19;Q53F19-2	Uncharacterized protein C1	C17orf85	-0.1058178	0.755953227	1.50E-30
P22415;E9PME6;Q7Z5Y1;B1A	Upstream stimulatory facto	USF1;usf1-bd	0.4351463	0.756272544	1.82E-23
P14635;H0YA62;Q5TZP9;E9P	G2/mitotic-specific cyclin-B	CCNB1	-0.105402	0.756320041	8.17E-06
P15170;H3BR35;P15170-2;J3	Eukaryotic peptide chain rel	GSPT1	-0.1045055	0.757111065	4.52E-11
Q04837;C9K0U8;E7EUY5	Single-stranded DNA-bindin	SSBP1	-0.1027813	0.758633239	4.53E-22
O00472;B4DNK7	RNA polymerase II elongatio	ELL2	0.4317207	0.759234598	2.35E-23
P49458;E9PE20;P49458-2	Signal recognition particle 9	SRP9	-0.1518097	0.760117922	2.41E-46
P62891;Q59GN2	60S ribosomal protein L39;F	RPL39;RPL39P5	-0.1513119	0.760888327	4.99E-05
P62834;P61224;P61224-2;P6	Ras-related protein Rap-1A;	RAP1A;RAP1B	0.4290524	0.761544353	8.18E-18
P09012;M0R268;M0QZG7	U1 small nuclear ribonucleo	SNRPA	-0.1497078	0.763372351	1.57E-148
Q6UX04;Q6UX04-2	Peptidyl-prolyl cis-trans isor	CWC27	0.4198265	0.769546524	1.03E-77
P42167;G5E972;P42167-2;H0	Lamina-associated polypept	TMPO	0.1557102	0.771592665	1.92E-88
P12270	Nucleoprotein TPR	TPR	0.4158363	0.773015132	3.44E-09
P21333;Q5HY54;P21333-2	Filamin-A	FLNA	-0.1433678	0.773213938	0
Q9UFC0;H7C5S6	Leucine-rich repeat and WD	LRWD1	-0.08474731	0.774608806	4.44E-38
O95831;O95831-3;O95831-2	Apoptosis-inducing factor 1	AIFM1	0.1535683	0.774619235	2.51E-109
P38646	Stress-70 protein, mitochon	HSPA9	-0.1422997	0.774875611	7.68E-281
P62854;Q5JNZ5;F8VZW7	40S ribosomal protein S26;F	RPS26;RPS26P11	0.1528549	0.775627956	7.28E-35
Q9UBI6	Guanine nucleotide-binding	GNG12	-0.08359718	0.775630985	5.12E-11
O43818	U3 small nucleolar RNA-inte	RRP9	0.1526604	0.775903128	1.64E-219
P63272;D3DTZ5;J3QSB9	Transcription elongation fac	SUPT4H1	-0.1409073	0.777043267	2.19E-08
P29084;E5RH41	Transcription initiation fact	GTF2E2	-0.1407013	0.77736411	1.53E-163
Q96DI7;B4DQJ1	U5 small nuclear ribonucleo	SNRNP40	0.1508312	0.778491652	9.66E-88
O14974;O14974-4;O14974-3;	Protein phosphatase 1 regu	PPP1R12A	-0.1394272	0.779349421	0
Q2KHR3;Q2KHR3-2;H0YCQ7	Glutamine and serine-rich p	QSER1	0.4085445	0.77936548	7.60E-29
Q13427;C9JN15;Q13427-2;C9	Peptidyl-prolyl cis-trans isor	PPIG	-0.07910538	0.779626787	1.95E-18
P55735;P55735-2;B4DXJ1	Protein SEC13 homolog	SEC13	0.405098	0.782372232	4.64E-11
Q9H6Y2;G3V1J0	WD repeat-containing prote	WDR55	0.1477699	0.782829344	4.89E-86
P62829;J3KT29;C9JD32;B9ZV	60S ribosomal protein L23	RPL23	-0.1369247	0.783252896	1.23E-69
P26641;B4DTG2;E7EMT2	Elongation factor 1-gamma	EEF1G	0.4038734	0.783441272	1.08E-81
O75821;K7EL20;K7EP16;K7EN	Eukaryotic translation initia	EIF3G	-0.07070732	0.787113059	6.78E-61
P18583-6;P18583;P18583-3;P	Protein SON	SON	0.1444874	0.787488048	1.40E-50
P35250;P35250-2;H7C5Q7;F8	Replication factor C subunit	RFC2	-0.07006073	0.787690276	3.86E-12
Q76FK4;F8WE42;Q76FK4-3;F	Nucleolar protein 8	NOL8	0.3980503	0.788530603	5.82E-37
O00505	Importin subunit alpha-4	KPNA3	0.1403503	0.793370307	5.73E-99

Q12948;Q99958;O43638	Forkhead box protein C1;Fo	FOXC1;FOXC2;FOXS	0.3907738	0.794902868	8.26E-12
Q92879;Q92879-2;Q92879-3	CUGBP Elav-like family men	CELF1	-0.06196213	0.794929736	2.98E-36
P62081;B5MCP9	40S ribosomal protein S7	RPS7	-0.1286793	0.796152878	1.89E-36
Q14160;Q14160-3;Q14160-2	Protein scribble homolog	SCRIB	0.3885307	0.796869946	2.74E-84
Q9NW82;D6RIW8	WD repeat-containing prote	WDR70	0.137352	0.7976409	3.61E-72
Q96AQ6;B4E1J0;F5H2F6;Q96	Pre-B-cell leukemia transcri	PBXIP1	-0.0588131	0.797749515	4.03E-21
Q9BW71;Q9BW71-2	HIRA-interacting protein 3	HIRIP3	0.1361847	0.799305145	1.48E-251
P36873;F8VYE8;P36873-2;F8'	Serine/threonine-protein pl	PPP1CC	-0.05666733	0.799672443	0
Q15393;Q15393-3	Splicing factor 3B subunit 3	SF3B3	0.3845291	0.80038244	1.18E-56
P37275;G3V1R3;F5H4I8;Q5V	Zinc finger E-box-binding hc	ZEB1	0.3845177	0.800392491	9.72E-68
Q9UI10-2;Q9UI10-3	Translation initiation factor	EIF2B4	0.383419	0.801357549	7.26E-31
Q9GZL7	Ribosome biogenesis protei	WDR12	-0.05452156	0.801596586	1.40E-23
Q14697;Q14697-2;F5H6X6;E!	Neutral alpha-glucosidase A	GANAB	-0.1250935	0.801780519	1.49E-153
Q8NEM2	SHC SH2 domain-binding pr	SHCBP1	0.3827801	0.801918964	9.00E-58
P32322;B4DMU0;E2QRB3;P3	Pyrroline-5-carboxylate red	PYCR1	-0.053339	0.802657518	6.45E-28
Q9GZT3;H0YJ40;G3V4X6;J3KI	SRA stem-loop-interacting F	SLIRP	-0.1238022	0.803809606	4.24E-31
Q99873;E9PKG1;Q99873-3;Q	Protein arginine N-methyltr	PRMT1	-0.1236191	0.804097442	2.12E-65
Q96A33;Q96A33-2	Coiled-coil domain-containi	CCDC47	0.132534	0.804515842	0
Q96C57;E7ENF1;F5H7W8;F5I	Uncharacterized protein C1	C12orf43	-0.1226463	0.805627016	1.51E-112
Q92769;B3KRS5;J3KPW7	Histone deacetylase 2;Histo	HDAC2	0.3769188	0.807073635	8.66E-41
Q09028;Q09028-3;Q09028-4	Histone-binding protein RBI	RBBP4	0.1303215	0.807678088	1.28E-54
Q9H3Q1;B3KUS7;J3KRZ9	Cdc42 effector protein 4	CDC42EP4	-0.1209011	0.808373113	1.84E-178
Q15904;H7C2Y8;A6QRJ1;A6N	V-type proton ATPase subu	ATP6AP1	0.3735142	0.810071673	5.59E-63
O00267;O00267-2	Transcription elongation fac	SUPT5H	0.12784	0.811228451	0
Q7Z4V5;Q7Z4V5-2;K7EQZ6;I3	Hepatoma-derived growth f	HDGFRP2	0.1271782	0.812176056	1.54E-111
P46777	60S ribosomal protein L5	RPL5	0.1246338	0.815821559	7.05E-142
P46060;F8W7I9	Ran GTPase-activating prote	RANGAP1	0.1243114	0.816283682	0
P09543;P09543-2	2,3-cyclic-nucleotide 3-phos	CNP	0.36549	0.817148595	2.44E-20
Q12874;E7EUT8	Splicing factor 3A subunit 3	SF3A3	0.3649082	0.817662246	6.05E-40
O00193;E9PRZ9;E3W975;E9P	Small acidic protein	SMAP;C11orf58	-0.03643036	0.817866019	3.32E-297
P24752;G3XAB4;H0YEL7;E9PI	Acetyl-CoA acetyltransferas	ACAT1	0.3641987	0.818288839	6.38E-29
Q13148;Q13148-2;B1AKP7;G	TAR DNA-binding protein 4:	TARDBP	0.1227188	0.818567888	2.52E-48
Q14978-3;Q14978	Nucleolar and coiled-body p	NOLC1	0.1224747	0.818918178	1.97E-122
Q9NRY2;Q9NRY2-2	SOSS complex subunit C	INIP	-0.03475189	0.819379566	0.00033232
Q15007;Q15007-2	Pre-mRNA-splicing regulato	WTAP	-0.03290367	0.821046967	8.90E-65
Q96QC0;F5H5K4	Serine/threonine-protein pl	PPP1R10	-0.0323391	0.821556468	1.74E-35
Q9H814	Phosphorylated adapter RN	PHAX	0.3592911	0.822625929	1.78E-08
P43307;C9J3L8;C9J5W0;E9PA	Translocon-associated prote	SSR1	0.1195011	0.823187486	1.94E-71
Q9BSD7;Q5TDF0	Cancer-related nucleoside-t	NTPCR	-0.03030396	0.823393708	3.65E-11
O43852-4;O43852-2;O43852-	Calumenin	CALU	-0.02959633	0.82403275	1.79E-124
P51148;P51148-2;K7ENY4;K7	Ras-related protein Rab-5C	RAB5C	-0.02935982	0.824246365	6.08E-12
Q9NPL8;C9JU35	Translocase of inner mitoch	TIMMDC1	0.3566208	0.824988079	2.42E-07
Q9UN86;Q9UN86-2;D6RAC7;	Ras GTPase-activating prote	G3BP2	-0.1085281	0.82790774	7.64E-87
P10809	60 kDa heat shock protein,	HSPD1	-0.107933	0.828850074	1.66E-108

Q6SPF0;E9PIW9	Atherin	SAMD1	-0.02071953	0.832058982	1.41E-42
Q06587;Q06587-2	E3 ubiquitin-protein ligase F	RING1	0.1131725	0.832291089	2.05E-59
P18621;J3QQT2;J3KRX5;J3QL	60S ribosomal protein L17	RPL17	0.1131077	0.832384494	2.67E-78
P23258;Q9NRH3;K7EKE5;K7E	Tubulin gamma-1 chain;Tub	TUBG1;TUBG2	0.3478889	0.83272327	8.13E-23
P05388;Q3B7A4;F8VWS0;F8\	60S acidic ribosomal protei	RPLP0;RPLP0P6	0.1113586	0.83490456	7.95E-122
P35269;M0R0R9;E7EUG6;M0	General transcription factor	GTF2F1	-0.1036682	0.835610594	1.15E-245
Q7Z6M4;B4DKD5;H7C316;C9	mTERF domain-containing p	MTERFD2	0.3444862	0.835742031	7.62E-08
P61353;K7ELC7;K7EQQ9	60S ribosomal protein L27	RPL27	-0.1033344	0.836140224	2.20E-17
Q9BVI4;F5H303	Nucleolar complex protein 4	NOC4L	0.3437958	0.836354879	1.37E-32
P30101;G5EA52	Protein disulfide-isomerase	PDIA3	0.1095219	0.837552868	3.38E-133
P19784;H3BSA1	Casein kinase II subunit alpl	CSNK2A2	0.1088867	0.838469068	0
P17544;P17544-2;P17544-4;F	Cyclic AMP-dependent tran	ATF7	-0.01325798	0.838819179	1.77E-15
O94776	Metastasis-associated prote	MTA2	0.3406811	0.839120697	8.01E-71
P04406;E7EUT4;E7EUT5	Glyceraldehyde-3-phosphat	GAPDH	0.1074505	0.840541641	6.88E-101
Q00610;Q00610-2	Clathrin heavy chain 1	CLTC	0.1071053	0.841039994	2.53E-271
P62249;M0R3H0;M0R210;Q6	40S ribosomal protein S16	RPS16	-0.09998703	0.841455728	2.51E-15
P40425;F8VSK3;F8VNW9;F8V	Pre-B-cell leukemia transcri	PBX2	-0.00717926	0.844335356	7.44E-56
P49770	Translation initiation factor	EIF2B2	-0.00576973	0.845615546	1.68E-34
P40938;P40938-2	Replication factor C subunit	RFC3	-0.004699707	0.846587655	2.96E-15
Q9P289;Q8NBY1;B4E0Y9;Q9I	Serine/threonine-protein ki	MST4	0.1014442	0.849220837	3.89E-153
P31944	Caspase-14;Caspase-14 sub	CASP14	-0.00179863	0.849224434	1.21E-39
P60842;J3KT12;P60842-2;J3K	Eukaryotic initiation factor 4	EIF4A1	0.09967422	0.851782108	2.49E-102
Q8IXH7;Q8IXH7-4	Negative elongation factor (	NELFCD	0.001096725	0.851857696	4.82E-27
Q9NQG5	Regulation of nuclear pre-m	RPRD1B	0.3263531	0.851868894	8.98E-111
Q9BQ70;H3BS87;H3BMJ8;H3	Transcription factor 25	TCF25	0.001382828	0.85211799	8.49E-07
P33991;J3KPV4	DNA replication licensing fa	MCM4	0.0986557	0.853256654	9.22E-136
Q86UE4;E5RJU9	Protein LYRIC	MTDH	0.09711838	0.855483248	2.09E-168
P06454;P06454-2;B8ZZQ6	Prothymosin alpha;Prothym	PTMA	0.09698486	0.855676679	2.73E-05
Q53EL6;Q53EL6-2	Programmed cell death pro	PDCD4	0.09594154	0.857188504	4.46E-102
Q00839;Q00839-2	Heterogeneous nuclear ribo	HNRNPU	0.09564781	0.857614232	3.11E-264
P14406;D6RIE3;D6RGV5;H0U	Cytochrome c oxidase subu	COX7A2	0.009567261	0.859570844	2.99E-46
Q02413	Desmoglein-1	DSG1	-0.08828735	0.860089546	1.69E-38
Q5QJE6;J3KP30	Deoxynucleotidyltransferas	DNTTIP2	0.09380341	0.86028841	1.08E-152
Q9BPZ3;D6RA77	Polyadenylate-binding prot	PAIP2	0.01086807	0.860756552	6.17E-18
P09132;P09132-2	Signal recognition particle 1	SRP19	0.01092911	0.860812194	8.40E-50
O95218;O95218-2	Zinc finger Ran-binding dom	ZRANB2	0.09309387	0.861317581	5.56E-160
P52292	Importin subunit alpha-1	KPNA2	-0.08735085	0.861584619	1.13E-227
Q9Y6E0-2;B4DR80;Q9Y6E0;H	Serine/threonine-protein ki	STK24	0.0125618	0.862300876	8.46E-101
Q969G3;K7EMQ8;C0IMW7;C	SWI/SNF-related matrix-ass	SMARCE1	0.01316643	0.8628523	1.00E-41
Q9BRX2	Protein pelota homolog	PELO	-0.08617401	0.86346406	1.19E-242
Q92499	ATP-dependent RNA helicas	DDX1	0.01477242	0.864317288	1.45E-43
P55795	Heterogeneous nuclear ribo	HNRNPH2	0.01706314	0.866407704	4.41E-95
Q6P1J9	Parafibromin	CDC73	0.08864784	0.867771796	4.19E-52
O95400	CD2 antigen cytoplasmic tai	CD2BP2	0.02024269	0.869310768	1.00E-58

Q6P5R6;C9JYQ9;H0Y8C2	60S ribosomal protein L22-l	RPL22L1	0.02033997	0.869399612	8.38E-16
O94842;B4DPY8	TOX high mobility group bo:	TOX4	0.0225029	0.871375508	7.02E-63
Q9NYB0	Telomeric repeat-binding fa	TERF2IP	0.08602524	0.871583145	3.22E-43
P17987;E7EQR6;F5H282;E7EI	T-complex protein 1 subuni	TCP1	0.3034401	0.87233481	3.87E-37
P08107;P08107-2;F8VZJ4;E7E	Heat shock 70 kDa protein 1	HSPA1A	-0.07895279	0.875013107	4.14E-113
P05198;H0YJS4;G3V4T5	Eukaryotic translation initia	EIF2S1	0.2996445	0.875733718	7.69E-49
Q9HCY8	Protein S100-A14	S100A14	0.02786446	0.876276867	3.33E-96
P62314;J3QLI9	Small nuclear ribonucleopro	SNRPD1	-0.07797623	0.876577046	3.16E-227
P78347;P78347-2;P78347-4;F	General transcription factor	GTF2I	0.08144569	0.878245556	1.05E-175
Q08554;Q08554-2	Desmocollin-1	DSC1	0.03006172	0.878286918	1.52E-45
P40424-2;P40424;H0YLF5;P4(	Pre-B-cell leukemia transcri	PBX1	-0.07606888	0.879632997	7.99E-105
P18887;F5H8D7	DNA repair protein XRCC1	XRCC1	-0.07545853	0.880611283	2.62E-68
O60237;F8W8M3;H7C4R6;O6	Protein phosphatase 1 regu	PPP1R12B	0.03292274	0.880905346	3.38E-19
P05455;E9PFH8;E7ERC4;E9P(	Lupus La protein	SSB	-0.07467461	0.881868036	7.74E-176
Q6ZRS2;Q6ZRS2-3;Q6ZRS2-2	Helicase SRCAP	SRCAP	0.03495979	0.882770462	0.0012458
P61254;Q9UNX3;J3KTJ8;J3QF	60S ribosomal protein L26;6	RPL26;RPL26L1;KRB	0.07590866	0.886312428	2.57E-11
Q8WYA6;B4DE16;Q8WYA6-3	Beta-catenin-like protein 1	CTNNBL1	0.0737381	0.889477974	0
P17275	Transcription factor jun-B	JUNB	0.2828369	0.890811275	3.21E-51
P43243;A8MXP9;D6REM6;D6	Matrin-3	MATR3	-0.06845856	0.891843658	9.40E-84
P78316;E9PFK5;P78316-2	Nucleolar protein 14	NOP14	0.2797012	0.893628745	2.15E-29
P47712;E7EU42	Cytosolic phospholipase A2;	PLA2G4A	0.2795887	0.893729884	5.53E-51
O95782;O95782-2	AP-2 complex subunit alpha	AP2A1	0.04842377	0.89511391	4.05E-50
Q99614;H0YB37	Tetratricopeptide repeat pr	TTC1	0.275526	0.897382309	4.16E-24
Q5SRQ6;P67870;Q5SRQ3	Casein kinase II subunit beta	CSNK2B;CSNK2B-LY(	0.06723976	0.898965481	1.30E-105
O96019;O96019-2;H7C5S0	Actin-like protein 6A	ACTL6A	-0.06315994	0.900360563	1.17E-52
P14314;P14314-2;K7ELL7;K7E	Glucosidase 2 subunit beta	PRKCSH	-0.06185532	0.902459397	5.31E-138
Q14839;F5GWX5;Q14839-2	Chromodomain-helicase-DN	CHD4	0.2682076	0.903967257	1.05E-73
Q96RT1;B4DIP2;Q96RT1-7;Q9	Protein LAP2	ERBB2IP	0.2647552	0.907075848	0.00093425
P62906	60S ribosomal protein L10a	RPL10A	-0.05830574	0.908173233	1.49E-113
P35221;G3XAM7;P35221-2;F	Catenin alpha-1	CTNNA1	0.2627373	0.908893577	1.78E-78
Q6UN15;Q6UN15-4;Q6UN15	Pre-mRNA 3-end-processing	FIP1L1	0.06359482	0.909052708	4.70E-120
P39880;P39880-6;P39880-4;F	Homeobox protein cut-like	CUX1	0.258194	0.912987776	3.02E-48
Q9H8N7;E5RH90;E5RG59;E5F	Zinc finger protein 395	ZNF395	0.2578506	0.913297256	2.00E-07
Q969X6;Q969X6-2;H3BSH7;C	Cirhin	CIRH1A	0.2563629	0.91463849	1.04E-50
P61247;D6RG13;D6RAT0;D6F	40S ribosomal protein S3a	RPS3A	-0.05405235	0.915026249	5.74E-44
Q9NS91;F8WE49	E3 ubiquitin-protein ligase F	RAD18	0.2557278	0.915211168	2.04E-31
O60506;O60506-4;O60506-3;	Heterogeneous nuclear ribo	SYNCRIP	0.05546951	0.916185819	7.21E-104
P61201;B4DIH5;P61201-2	COP9 signalosome complex	COPS2	0.2543106	0.916489103	2.16E-53
Q8TAQ2;Q8TAQ2-2;F8VZW6;	SWI/SNF complex subunit S	SMARCC2	0.07325935	0.917947149	3.64E-64
Q9HAV0	Guanine nucleotide-binding	GNB4	0.07330322	0.917987547	2.17E-18
Q96EY1;Q96EY1-2;E7ES32	DnaJ homolog subfamily A r	DNAJA3	0.07332039	0.918003355	5.92E-40
Q9H307;Q9H307-2	Pinin	PNN	0.0536499	0.918851701	2.87E-74
P49790;F6QR24	Nuclear pore complex prote	NUP153	0.2513924	0.919121328	3.18E-105
Q9NV56	MRG/MORF4L-binding prot	MRGBP	0.07480431	0.919369978	6.89E-21

Q5T749	Keratinocyte proline-rich pr	KPRP	0.2488518	0.921413635	3.00E-25
P55036;Q5VWC4;P55036-2;A	26S proteasome non-ATPas	PSMD4	0.07765198	0.921993223	4.63E-56
P62826;B5MDF5;F5H018;J3K	GTP-binding nuclear protein	RAN	0.05109787	0.922592175	4.30E-32
P68104;Q5VTE0	Elongation factor 1-alpha 1;	EEF1A1;EEF1A1P5	0.0483284	0.926653278	1.71E-50
P80303;P80303-2;J3KQU0;H0	Nucleobindin-2;Nesfatin-1	NUCB2	0.2430058	0.926690799	5.40E-49
Q12905	Interleukin enhancer-bindin	ILF2	-0.04460144	0.930275356	1.68E-39
Q9Y2W1	Thyroid hormone receptor-a	THRAP3	-0.04390144	0.931405894	3.96E-216
P62136;P62136-2;A6NNR3;E9	Serine/threonine-protein ph	PPP1CA	0.23773	0.931455937	0
Q9UBU8;H0YMJ0;H0YLJ3;Q9U	Mortality factor 4-like prote	MORF4L1	0.08799934	0.931532044	2.43E-19
Q71UM5;C9JLI6;H0YMV8	40S ribosomal protein S27-l	RPS27L	0.044981	0.931564386	5.16E-19
P10155;Q5LJA0;P10155-3;P10	60 kDa SS-A/Ro ribonucleop	TROVE2	0.2369499	0.932160756	1.14E-33
O95251;O95251-4;E7EUP3;G	Histone acetyltransferase K	KAT7	0.08899307	0.932448663	4.49E-43
Q9UBD5;Q9UBD5-2;Q9UBD5	Origin recognition complex	ORC3	0.2353077	0.933644668	2.35E-60
Q9H1E5;E9PIN7	Thioredoxin-related transm	TMX4	0.233326	0.935435665	2.31E-16
Q8IWS0;E9PC97;B4E0G4;Q5J	PHD finger protein 6	PHF6	-0.04107285	0.93597564	1.53E-163
Q8WWY3;E7EVX8;Q8WWY3-	U4/U6 small nuclear ribonu	PRPF31	0.2314587	0.937123536	1.81E-33
Q5JRA6;Q5JRA6-3;Q5JRA6-2;	Melanoma inhibitory activit	MIA3	0.2309189	0.937611502	3.27E-05
Q14257;F8WCY5;A8MXP8	Reticulocalbin-2	RCN2	0.09555626	0.938504759	9.75E-22
O43684;J3QT28;O43684-2;B4	Mitotic checkpoint protein	BUB3	-0.03789139	0.941117962	4.30E-47
Q92542;Q5T205;H0Y6T7;H0Y	Nicastrin	NCSTN	0.09945869	0.94210739	0.00038474
P07237;H7BZ94;I3L2P8;F5H8	Protein disulfide-isomerase	P4HB	-0.03500938	0.945778391	1.85E-86
P08579	U2 small nuclear ribonucleo	SNRPB2	-0.03443909	0.946700832	4.84E-60
Q9UJX3;Q9UJX3-2;F8VZ62	Anaphase-promoting compl	ANAPC7	0.2172699	0.949957625	8.53E-31
Q15853;M0QXT0;Q6YI47;Q15	Upstream stimulatory facto	USF2	0.2171116	0.9501009	1.12E-12
P62424;Q5T8U2	60S ribosomal protein L7a	RPL7A	-0.03158188	0.951323343	5.17E-100
Q08J23;Q08J23-2;G3V1R4	tRNA (cytosine(34)-C(5))-me	NSUN2	0.1108513	0.95263126	2.35E-53
O75534;E9PLT0;O75534-2;O7	Cold shock domain-containi	CSDE1	0.2128601	0.953949192	7.39E-08
Q14444;G3V153;Q14444-2;E9	Caprin-1	CAPRIN1	0.02790451	0.956654631	1.69E-59
O43390;O43390-2;E7ETM7;C	Heterogeneous nuclear ribo	HNRNPR	0.1181087	0.959339741	1.51E-35
Q01105	Protein SET	SET	-0.02621078	0.960017234	0
Q9P258	Protein RCC2	RCC2	0.02509117	0.960793023	1.33E-146
P80723	Brain acid soluble protein 1	BASP1	0.1992073	0.966314048	1.30E-06
Q9Y657	Spindlin-1	SPIN1	0.1258678	0.966515084	2.14E-18
Q9UHR5;J3QQJ0;Q9UHR5-2;J	SAP30-binding protein	SAP30BP	-0.02173996	0.967257583	2.13E-83
Q14119;J9JIC7;J3QSH4	Vascular endothelial zinc fin	VEZF1	0.1971569	0.968171795	3.14E-14
Q9BUQ8	Probable ATP-dependent RI	DDX23	0.1279831	0.968471689	3.27E-05
P29083	General transcription factor	GTF2E1	-0.02049637	0.969272042	2.58E-191
P05386	60S acidic ribosomal protein	RPLP1	-0.019701	0.970560535	5.25E-40
Q6UWP8	Suprabasin	SBSN	0.1303902	0.970698461	1.75E-19
Q9ULX3;H3BUR4	RNA-binding protein NOB1	NOB1	0.131197	0.971444888	1.04E-85
Q9H2K8;G3V1Q8	Serine/threonine-protein ki	TAOK3	0.1923866	0.972494515	1.08E-31
P49736;H0Y8E6	DNA replication licensing fa	MCM2	0.1908379	0.973898154	1.10E-67
P62140;E7ETD8	Serine/threonine-protein ph	PPP1CB	0.01555634	0.974825863	0
Q96CB9;Q96CB9-4	5-methylcytosine rRNA met	NSUN4	0.1359119	0.975807463	3.24E-23

Q02543;M0R0P7;M0R3D6;M	60S ribosomal protein L18a	RPL18A	-0.01609612	0.976401378	9.44E-14
P60059	Protein transport protein Se	SEC61G	0.1872177	0.977179379	1.95E-05
P62269;J3JS69	40S ribosomal protein S18	RPS18	-0.01545906	0.97743371	6.71E-26
P61011;P61011-2;G3V4F7	Signal recognition particle 5	SRP54	0.01377869	0.977443101	5.85E-231
Q96HY6;Q96HY6-2	DDRGK domain-containing p	DDRGK1	0.1384392	0.978146123	1.84E-48
Q14566	DNA replication licensing fa	MCM6	-0.01407623	0.979674678	8.70E-93
P68400;E7EU96;P68400-2	Casein kinase II subunit alph	CSNK2A1	0.011343	0.981029554	0
P11532;P11532-3;P11532-2;P	Dystrophin	DMD	-0.01256561	0.982122925	0.0010488
Q9Y4X5;H3BSK4;H3BNB9	E3 ubiquitin-protein ligase A	ARIH1	0.145916	0.985066052	3.35E-46
P12004	Proliferating cell nuclear an	PCNA	0.1782207	0.985335591	1.18E-22
Q99733;F5HFY4;C9JZI7;H0YC	Nucleosome assembly prote	NAP1L4;NAP1L4b	0.00838089	0.985391662	5.72E-126
Q96B36;Q96B36-2;J3KPM3	Proline-rich AKT1 substrate	AKT1S1	0.1772156	0.986246943	5.78E-12
Q7L014	Probable ATP-dependent RI	DDX46	0.007551193	0.986613586	2.39E-203
Q9Y305;C9J7L8;Q9Y305-3;Q9	Acyl-coenzyme A thioestera	ACOT9	0.176527	0.986871238	1.18E-09
Q15287;H3BV80;H3BMM9;H	RNA-binding protein with se	RNPS1	-0.009506226	0.987081736	1.66E-34
Q9Y6H1;Q5T1J5	Coiled-coil-helix-coiled-coil-	CHCHD2;CHCHD2P9	-0.009208679	0.987564044	4.54E-81
P0C0S5;Q71UI9;Q71UI9-3;Q7	Histone H2A.Z;Histone H2A	H2AFZ;H2AFV	-0.009002686	0.987897952	1.03E-13
P62917;E9PKZ0;G3V1A1;E9PI	60S ribosomal protein L8	RPL8	-0.008401871	0.988871863	3.54E-89
H7BZJ3	Thioredoxin	PDIA3	-0.008041382	0.989456217	4.47E-27
Q9H3P7	Golgi resident protein GCP6	ACBD3	0.173336	0.989764539	2.23E-52
P62308;Q49AN9;F5H013;A8M	Small nuclear ribonucleopro	SNRPG	-0.007591248	0.990185895	1.00E-32
P23396;J3KN86;E9PPU1;E9PL	40S ribosomal protein S3	RPS3	-0.00718689	0.990841376	2.15E-87
P62280;M0QZC5;M0R1H5	40S ribosomal protein S11	RPS11	0.004558563	0.991021179	1.80E-42
O75128;O75128-2;H7C1N2;C	Protein cordon-bleu	COBL	0.1715088	0.991421375	3.53E-17
Q9H0A0;E7ESU4	N-acetyltransferase 10	NAT10	0.1534367	0.992027714	1.29E-87
P33778;Q16778;Q99877;Q93	Histone H2B type 1-B;Histor	HIST1H2BB;HIST2H2	-0.004795074	0.994718717	1.27E-70
P06576;F8VPV9;H0YH81;F8W	ATP synthase subunit beta,	ATP5B	0.1674309	0.995119114	5.32E-143
P62995;P62995-3;H7BXF3	Transformer-2 protein hom	TRA2B	0.1653748	0.996983594	1.95E-18
O75152;E9PQ61	Zinc finger CCCH domain-co	ZC3H11A	0.1645851	0.997699642	2.98E-12
P83731;C9JXB8;C9JNW5	60S ribosomal protein L24	RPL24	-0.001537323	1	1.39E-43
P62244;I3L3P7;I3L246;H3BN9	40S ribosomal protein S15a	RPS15A	0.1620483	1	6.31E-19

Supplementary table 3						
Identification and quantification of interacting protein groups for bait (H2A/B S112 GlcNAc dimer) versus control (wt H2A/B dimer) samples. Statistically significant interacting partners are denoted with a '+' in column F						
**Majority Protein Ids**	**Protein names**	**Gene names**	**log2(Bait/Control)**	**Significance B value**	**Statistically Significant Interactor**	**PEP**
P01834	Ig kappa chain C region	IGKC	6.802614	5.06E-80	+	0
P05109	Protein S100-A8;Protein S1(	S100A8	6.20429	7.85E-67	+	4.87E-28
P02647;F8W696	Apolipoprotein A-I;Truncate	APOA1	5.482416	1.58E-52	+	3.34E-92
P06702	Protein S100-A9	S100A9	4.830589	3.99E-41	+	5.18E-63
P51858;A8K8G0;P51858-2;Q!	Hepatoma-derived growth f	HDGF	-4.617218	2.85E-40	+	4.42E-91
P61626;F8VV32	Lysozyme C	LYZ	3.742329	2.78E-25	+	2.66E-22
Q7Z7G8;Q7Z7G8-4;Q7Z7G8-3	Vacuolar protein sorting-as:	VPS13B	-3.079212	6.48E-19	+	0.0010471
P25311;C9JEV0	Zinc-alpha-2-glycoprotein	AZGP1	3.200577	7.01E-19	+	7.20E-40
P14618;P14618-2;H3BQ34;H3	Pyruvate kinase PKM;Pyruva	PKM;PKM2	3.066269	1.46E-15	+	1.10E-18
P17275	Transcription factor jun-B	JUNB	-3.41736	1.38E-11	+	1.79E-58
Q8IWA0	WD repeat-containing prote	WDR75	-3.254229	1.25E-10	+	4.37E-59
P02790	Hemopexin	HPX	2.225679	7.75E-10	+	1.07E-36
P33778;Q16778;Q99877;Q93	Histone H2B type 1-B;Histor	HIST1H2BB;HIST2H2	2.122673	4.57E-09	+	1.83E-60
Q08554;Q08554-2	Desmocollin-1	DSC1	-1.945415	1.71E-08	+	2.32E-38
Q5T749	Keratinocyte proline-rich pr	KPRP	-1.922817	2.48E-08	+	3.95E-52
Q00341;Q00341-2;H7C0A4;H	Vigilin	HDLBP	-2.654352	1.67E-07	+	6.15E-32
P40424-2;P40424;H0YKH1;H(	Pre-B-cell leukemia transcri	PBX1	-2.627068	2.24E-07	+	2.20E-22
Q9Y2Z9;G3V2L5;G3V2W8;G3	Ubiquinone biosynthesis mo	COQ6	-2.59762	3.06E-07	+	0.010186
Q99583	Max-binding protein MNT	MNT	-2.459705	1.27E-06	+	1.15E-31
Q02413	Desmoglein-1	DSG1	-1.635183	2.02E-06	+	2.31E-87
P61978;P61978-2;Q5T6W5;P	Heterogeneous nuclear ribo	HNRNPK	-1.573532	4.76E-06	+	8.40E-204
Q14151	Scaffold attachment factor I	SAFB2	-2.271959	7.89E-06	+	6.33E-67
O00148;Q13838;Q8N5M0;Q1	ATP-dependent RNA helicas	DDX39A;DDX39B	-1.527676	8.83E-06	+	8.86E-60
P31151;Q86SG5	Protein S100-A7;Protein S1(	S100A7;S100A7A	1.595768	1.14E-05	+	4.11E-19
P12273	Prolactin-inducible protein	PIP	1.627232	1.85E-05	+	3.89E-22
Q05519;Q05519-2;Q5T760;Q	Serine/arginine-rich splicing	SRSF11	-1.435543	2.91E-05	+	3.66E-15
Q14247;Q14247-3;Q14247-2	Src substrate cortactin	CTTN	-1.395098	4.81E-05	+	1.15E-27
Q9UI10-2;Q9UI10-3;Q9UI10;I	Translation initiation factor	EIF2B4	1.532175	5.40E-05	+	1.55E-41
Q13247;Q13247-3	Serine/arginine-rich splicing	SRSF6	-2.023279	7.17E-05	+	8.21E-16
Q8N684;F5H669;Q8N684-2;C	Cleavage and polyadenylati	CPSF7	-2.014002	7.75E-05	+	1.05E-09
Q9UHX1-4;Q9UHX1-6;Q9UH>	Poly(U)-binding-splicing fact	PUF60	-1.355198	7.80E-05	+	1.13E-100
O43809;H3BND3;H3BV41	Cleavage and polyadenylati	NUDT21	-1.269453	0.000211246	+	2.59E-44
Q92979	Ribosomal RNA small subun	EMG1	1.391155	0.000237676	+	9.29E-13
Q9H2K8	Serine/threonine-protein ki	TAOK3	-1.866413	0.000255536	+	8.70E-24
P63167;Q96FJ2	Dynein light chain 1, cytopla	DYNLL1;DYNLL2	-1.228333	0.000333847	+	2.61E-73

P01617;P01614;P06309;P063	Ig kappa chain V-II region TEW;Ig kappa chain V-		-1.200989	0.000449334	+	3.86E-46
P35321	Cornifin-A	SPRR1A	1.307377	0.000540413	+	6.04E-36
P61981	14-3-3 protein gamma;14-3	YWHAG	1.250254	0.000618282	+	1.17E-114
P35908	Keratin, type II cytoskeletal	KRT2	-1.142862	0.000828924	+	0
P04792;F8WE04	Heat shock protein beta-1	HSPB1	1.258228	0.00085738	+	1.18E-07
Q9Y450;B7Z524;H0YDX7;J3Q	HBS1-like protein	HBS1L	-1.693838	0.000928501	+	2.71E-48
Q15517;Q2L6G8;G8JLG2	Corneodesmosin	CDSN	-1.689846	0.000955361	+	0.00025876
P14923	Junction plakoglobin	JUP	-1.128023	0.000965157	+	1.03E-76
P27816;P27816-6;E7EVA0;P2	Microtubule-associated pro	MAP4	-1.11367	0.001116392	+	4.84E-30
Q09161	Nuclear cap-binding protein	NCBP1	-1.590069	0.001911121		1.49E-13
P0C0L5;P0C0L4;P0C0L4-2;F8\	Complement C4-B;Complen	C4B;C4A	1.120478	0.002886583		1.71E-34
P18124;A8MUD9	60S ribosomal protein L7	RPL7	1.070112	0.003500857		3.35E-34
Q9BUL8;C9J363;C9J5C3;C9J6	Programmed cell death pro	PDCD10	-0.9940052	0.003533966		9.19E-46
F5GWP8		JUP	-0.9869232	0.003770468		1.14E-104
Q9UKS6;E9PIY1	Protein kinase C and casein	PACSIN3	-0.9711704	0.00434891		8.48E-114
P45880;B4DKM5;P45880-2;P	Voltage-dependent anion-s	VDAC2	1.033531	0.004836838		1.06E-06
Q14697;Q14697-2;F5H6X6;E	Neutral alpha-glucosidase A	GANAB	-1.406168	0.006224038		7.21E-80
E9PLD3;E9PRG8		C11orf48	1.021023	0.006450178		3.55E-05
P05114;A6NL93;A6NEL0	Non-histone chromosomal	HMGN1	0.9899445	0.008190749		1.02E-07
P01024	Complement C3;Compleme	C3	0.96171	0.008871764		7.83E-138
P24752;G3XAB4;H0YEL7;E9P	Acetyl-CoA acetyltransferas	ACAT1	-1.344604	0.008986795		1.85E-38
Q9Y3F4;B0AZV0;B4DNJ6;H0Y	Serine-threonine kinase rec	STRAP	-1.334658	0.00952387		3.22E-31
P63272;J3QR04;J3KSQ6	Transcription elongation fac	SUPT4H1	-0.8622074	0.011088568		1.30E-16
P53985;Q5T8R3;Q49A45;Q5	Monocarboxylate transport	SLC16A1	0.9318581	0.012601883		0.012637
P40855;B7Z6I5;Q5QNY5;P40	Peroxisomal biogenesis fact	PEX19;DCAF8	-1.284887	0.012664478		1.55E-108
P49959;P49959-2;F8W7U8;B	Double-strand break repair	MRE11A	-1.238564	0.016378155		2.18E-21
Q9NVI1;F8W7R3;Q9NVI1-2;C	Fanconi anemia group I pro	FANCI	-1.234787	0.01671938		4.16E-09
Q8WVV9;Q5JB52;B7WPG3;Q	Heterogeneous nuclear ribo	HNRNPLL;HNRPLL	-1.230684	0.017097134		4.65E-41
Q9NZT1	Calmodulin-like protein 5	CALML5	-1.229403	0.017216674		1.57E-34
P63244;D6RFX4;H0YAF8;D6R	Guanine nucleotide-binding	GNB2L1	0.8806419	0.018117663		1.30E-07
P43243;A8MXP9;D6REM6;D6	Matrin-3	MATR3	-0.7845535	0.020465681		1.48E-64
P31943;E9PCY7;G8JLB6;D6R	Heterogeneous nuclear ribo	HNRNPH1	-0.7810822	0.021012143		1.37E-87
P07237;H7BZ94;I3L2P8;F5H8	Protein disulfide-isomerase	P4HB	-0.7779045	0.021523468		3.53E-149
P62308;Q49AN9;F5H013;A8N	Small nuclear ribonucleopr	SNRPG	-0.7723751	0.022439077		4.75E-58
P62937;F8WE65;C9J5S7;Q56	Peptidyl-prolyl cis-trans isor	PPIA	0.8372536	0.024341255		1.33E-27
Q8N108;Q8N108-17;Q8N108	Mesoderm induction early r	MIER1	-1.163668	0.024416075		9.84E-09
P62805	Histone H4	HIST1H4A	0.8337688	0.024913313		7.27E-14
Q9H875;B4DGM2	PRKR-interacting protein 1	PRKRIP1	0.8318329	0.025236125		1.41E-22
Q9Y5L4	Mitochondrial import inner	TIMM13	-0.7391167	0.028692361		2.53E-63
P63010;P63010-2;Q7Z451;K7	AP-2 complex subunit beta;	AP2B1;AP1B1	0.8072548	0.029659722		6.72E-36
P62263	40S ribosomal protein S14	RPS14	0.7913342	0.032295079		3.88E-32
Q9NRY2;Q9NRY2-2	SOSS complex subunit C	INIP	-1.101973	0.033414111		2.42E-05

P46779;C9JB50;G5E9L2;H0YL	60S ribosomal protein L28	RPL28	0.785675	0.034078068	0.0090483
P46782;M0R0F0;M0QZN2;M(	40S ribosomal protein S5;4(	RPS5	0.7828064	0.034268061	6.54E-109
Q5T750	Skin-specific protein 32	XP32	-1.09687	0.034271482	0.00011919
P04908;P0C0S8;Q99878;Q93	Histone H2A type 1-B/E;Hist	HIST1H2AB;HIST1H2	0.7815151	0.034575613	9.67E-96
Q9H4L7;Q9H4L7-2	SWI/SNF-related matrix-ass	SMARCAD1	0.7828312	0.034700326	0.014817
Q7L0Y3	Mitochondrial ribonuclease	TRMT10C	-1.093466	0.034853997	8.40E-30
Q01844;Q01844-6;B0QYK0;Q	RNA-binding protein EWS	EWSR1	0.7670536	0.038184135	7.81E-118
P23528;Q9Y281;E9PQB7;G3V	Cofilin-1;Cofilin-2	CFL1;CFL2	0.7588654	0.040340251	4.73E-09
Q9P289;Q8NBY1;B4E0Y9;Q9I	Serine/threonine-protein ki	MST4	-0.6872673	0.041417334	5.52E-122
Q15269	Periodic tryptophan protein	PWP2	-1.055935	0.041855426	4.93E-18
P37108;H0YLA2	Signal recognition particle 1	SRP14	0.7517033	0.042359903	5.12E-97
Q92499	ATP-dependent RNA helicas	DDX1	-1.046686	0.043753057	5.75E-08
Q6SPF0;E9PIW9	Atherin	SAMD1	0.7455177	0.04380568	2.58E-15
P55735;A8MXL6;P55735-2;B4	Protein SEC13 homolog	SEC13	0.7304955	0.048003292	0.00023769
P41236;E7EUI7;E7EMN6;Q6N	Protein phosphatase inhibit	PPP1R2;PPP1R2P3	-1.014549	0.050920891	2.62E-15
Q9ULX3;H3BUR4	RNA-binding protein NOB1	NOB1	-1.011868	0.051561122	4.64E-45
P04843;B7Z4L4	Dolichyl-diphosphooligosac	RPN1	-0.6537666	0.051959394	8.66E-155
P68133;P68032;P63267;P627	Actin, alpha skeletal muscle	ACTA1;ACTC1;ACTG	0.7140121	0.054277354	2.47E-60
P06753-2;P06753-3;Q5VU59;	Tropomyosin alpha-3 chain	TPM3;TPM1;TPM4	-0.9992027	0.054676402	2.69E-10
P16435;H0Y4R2;F5H468;E7E\	NADPH--cytochrome P450 r	POR	-0.6405392	0.056699339	3.93E-158
Q9BQ61;K7ELS0;K7ERU7;K7E	Uncharacterized protein C1	C19orf43	-0.6403637	0.05676456	5.70E-08
Q14978-3;Q14978	Nucleolar and coiled-body p	NOLC1	0.702528	0.058422106	2.01E-93
F5H6N3;P09496-2	Clathrin light chain A	CLTA	0.6964779	0.058769099	8.21E-09
P18583-6;P18583;P18583-3;F	Protein SON	SON	-0.6318398	0.060008759	8.54E-40
Q01658	Protein Dr1	DR1	0.6938705	0.061717662	4.45E-15
P62854;Q5JNZ5;F8VZW7	40S ribosomal protein S26;F	RPS26;RPS26P11	0.6867352	0.064548174	5.13E-30
Q04917;A2IDB2	14-3-3 protein eta	YWHAH	0.6782703	0.065310815	7.49E-51
Q8NC51-3;Q8NC51-4	Plasminogen activator inhib	SERBP1	0.6749668	0.066559815	1.51E-152
P78347;P78347-2;P78347-4;F	General transcription factor	GTF2I	-0.6116371	0.068315191	4.32E-165
P26232;P26232-3;P26232-2;F	Catenin alpha-2	CTNNA2	-0.9425907	0.070581067	2.48E-40
P20042	Eukaryotic translation initia	EIF2S2	0.6641407	0.070791513	9.37E-42
Q9H0A0;E7ESU4	N-acetyltransferase 10	NAT10	0.6531391	0.075315322	1.80E-30
P15924;P15924-2	Desmoplakin	DSP	-0.5929489	0.07681908	0
Q8WWY3;E7EVX8;Q8WWY3-	U4/U6 small nuclear ribonu	PRPF31	-0.5857601	0.080312017	2.25E-27
Q99590;Q99590-2;F8VXG7;F8	Protein SCAF11	SCAF11	-0.5835876	0.081392618	4.01E-83
P26368;K7ENG2;P26368-2	Splicing factor U2AF 65 kDa	U2AF2	-0.5782566	0.084094405	5.79E-109
O43290	U4/U6.U5 tri-snRNP-associa	SART1	-0.9014835	0.08436333	2.37E-16
Q15020;Q15020-2;F8VV04;B7	Squamous cell carcinoma ar	SART3	0.6255627	0.087697653	5.43E-14
P63104;E7EX29;E7ESK7;B0AZ	14-3-3 protein zeta/delta	YWHAZ	0.6347122	0.088574591	3.78E-114
Q6P5R6;C9JYQ9;H0Y8C2	60S ribosomal protein L22-l	RPL22L1	0.6236839	0.088597742	2.63E-22
P61254;J3KTJ8;J3QRI7;J3QQ(	60S ribosomal protein L26;6	RPL26;KRBA2;RPL26	0.6343174	0.088781319	4.53E-08
Q12996	Cleavage stimulation factor	CSTF3	0.620615	0.090084041	5.20E-16

Q96QA5;J3KRG2	Gasdermin-A	GSDMA	-0.8755455	0.094128248	0.0036793
Q15014;Q5JXX1;Q5JXX2;Q5J)	Mortality factor 4-like prote	MORF4L2	0.609705	0.095530988	3.99E-07
Q53EL6;Q53EL6-2	Programmed cell death pro	PDCD4	-0.5557899	0.096287856	8.83E-68
Q15427;Q5SZ64	Splicing factor 3B subunit 4	SF3B4	0.6182461	0.097531468	2.73E-97
P13674;P13674-2;P13674-3	Prolyl 4-hydroxylase subuni	P4HA1	-0.5497017	0.099825109	4.43E-68
Q02878;F8W181;F8VR69;F8V	60S ribosomal protein L6	RPL6	0.5948429	0.103371834	1.34E-06
Q86X53;E5RHA3;H0YBT6;H0Y	Glutamate-rich protein 1	ERICH1	0.5946102	0.103498548	1.00E-36
O15533;O15533-2;D3YTI9;A8	Tapasin	TAPBP	0.6070328	0.104035115	2.96E-10
Q8IVL5;D3DNV8	Prolyl 3-hydroxylase 2	LEPREL1	-0.5419388	0.10448479	3.00E-46
P60660;P60660-2;G3V1Y7;F8	Myosin light polypeptide 6	MYL6	0.5900936	0.105982603	1.19E-06
O00267;O00267-2	Transcription elongation fac	SUPT5H	-0.5386772	0.106493366	2.51E-237
Q15428	Splicing factor 3A subunit 2	SF3A2	-0.5380955	0.106854816	7.86E-31
P22695;H3BSJ9;H3BRG4;H3B	Cytochrome b-c1 complex s	UQCRC2	-0.5344582	0.10913686	2.96E-112
P31930	Cytochrome b-c1 complex s	UQCRC1	-0.533308	0.109866416	1.56E-270
O14519;F5GZF0;F5GYA4	Cyclin-dependent kinase 2-a	CDK2AP1	0.5925159	0.112961267	1.63E-26
P80303;E9PKG6;J3KQU0;P80	Nucleobindin-2;Nesfatin-1	NUCB2	-0.8259182	0.115322887	8.49E-43
Q16643;Q16643-2;A8MV58;D	Drebrin	DBN1	-0.8208332	0.117691363	1.20E-22
Q9P013	Spliceosome-associated pro	CWC15	-0.5133743	0.123132852	1.30E-39
Q9UQ80;F8VTY8	Proliferation-associated pro	PA2G4	0.5602798	0.123585297	8.63E-20
P62913;P62913-2;Q5VVC9;Q	60S ribosomal protein L11	RPL11	-0.5126534	0.123635153	9.18E-32
Q9Y6E2;E7ETZ4;B5MCE7;Q7	Basic leucine zipper and W2	BZW2	-0.8081074	0.123786123	6.71E-10
Q32P28;Q32P28-3;H7C0D1;H	Prolyl 3-hydroxylase 1	LEPRE1	-0.8064232	0.124610861	5.97E-51
P55039;A8MZF9;J3QRI9;J3Q	Developmentally-regulated	DRG2	0.5557995	0.126417351	1.02E-26
P36578;H3BM89	60S ribosomal protein L4	RPL4	0.5719357	0.12663712	2.54E-42
Q96AG4	Leucine-rich repeat-contain	LRRC59	0.5635548	0.132562567	6.99E-89
P07919	Cytochrome b-c1 complex s	UQCRH	-0.4973087	0.134709254	1.32E-44
P17987;F5GZ03;F5H282;E7E	T-complex protein 1 subuni	TCP1	0.5385094	0.137822396	2.78E-06
P15880;H0YEN5;E9PQD7	40S ribosomal protein S2	RPS2	0.5377808	0.138319877	7.26E-23
Q6QNY0	Biogenesis of lysosome-rela	BLOC1S3	0.5338001	0.141062251	1.45E-23
P82979;Q567R9;H0YHG0	SAP domain-containing ribo	SARNP;CIP29	-0.7739182	0.141381739	4.18E-20
Q9Y5V0;E5RHV4;H0YAP1;E5F	Zinc finger protein 706	ZNF706	-0.7673836	0.144953614	6.12E-06
P02545;P02545-3;D6RAQ3;P	Prelamin-A/C;Lamin-A/C	LMNA	-0.4789753	0.148928084	5.30E-278
Q8WUA2	Peptidyl-prolyl cis-trans isor	PPIL4	-0.7601166	0.149006581	2.70E-12
Q15054;Q32N00;Q32MZ9	DNA polymerase delta subu	POLD3	-0.7472363	0.156401967	3.53E-20
Q92841;Q92841-3;Q92841-1	Probable ATP-dependent RI	DDX17	-0.7445545	0.157976149	5.26E-23
Q07020;F8VUA6;F8VWC5;G3	60S ribosomal protein L18	RPL18	0.510294	0.158115587	5.53E-32
P16989;P16989-3;P16989-2	Y-box-binding protein 3	YBX3	0.5282612	0.159895239	1.28E-121
Q9UNP9;Q9UNP9-2;Q5TGA3;	Peptidyl-prolyl cis-trans isor	PPIE	0.5017776	0.164665154	9.45E-08
Q8N488	RING1 and YY1-binding prot	RYBP	0.5004368	0.16571471	1.04E-18
Q92905;E5RHF2;E5RHH5	COP9 signalosome complex	COPS5	-0.7260017	0.169196993	2.76E-27
Q6NZI2;Q6NZI2-2	Polymerase I and transcript	PTRF	-0.724184	0.170327719	4.63E-88
Q86WB0;Q86WB0-3;C9J0I9;C	Nuclear-interacting partner	ZC3HC1	-0.7241821	0.170328908	2.50E-13

Q9NX58	Cell growth-regulating nucle	LYAR	0.5143147	0.171800277	5.26E-89
Q9H0G5;K7ERM9	Nuclear speckle splicing reg	NSRP1	-0.72052	0.172624163	9.17E-07
K7ER90	Eukaryotic translation initia	EIF3G	0.4895287	0.174440715	6.15E-50
P39023;H7C422;G5E9G0;H7C	60S ribosomal protein L3	RPL3	0.5058022	0.179385744	1.13E-35
P17096;P17096-3;H7BYM6	High mobility group protein	HMGA1	0.4828777	0.179926941	0.00021461
Q9UH99;J3KQE0;Q9UH99-2;E	SUN domain-containing pro	SUN2	0.4796028	0.182675015	2.55E-06
Q969S3	Zinc finger protein 622	ZNF622	-0.4380245	0.184789059	4.48E-50
P62241;Q5JR95	40S ribosomal protein S8	RPS8	0.4988155	0.185795326	1.93E-77
O60841	Eukaryotic translation initia	EIF5B	-0.4366341	0.186110121	5.90E-238
P16401	Histone H1.5	HIST1H1B	0.4982052	0.186363209	6.14E-10
Q9BQE3;F5H5D3	Tubulin alpha-1C chain	TUBA1C	-0.4358768	0.186832476	2.58E-276
Q96NB3	Zinc finger protein 830	ZNF830	-0.6963692	0.188343063	5.29E-21
P35250;P35250-2	Replication factor C subunit	RFC2	0.4723473	0.188873969	1.41E-05
P67809;H0Y449	Nuclease-sensitive element	YBX1	0.4954891	0.188905847	7.14E-115
P62857	40S ribosomal protein S28	RPS28	-0.4268742	0.195579893	5.10E-68
Q9UKL0;J3KN32	REST corepressor 1	RCOR1	-0.6846733	0.196324507	1.21E-25
Q8WWM7;H3BSK9;Q8WWM	Ataxin-2-like protein	ATXN2L	0.4635525	0.196594276	0.00039853
P14625	Endoplasmin	HSP90B1	-0.4249687	0.197469195	1.66E-97
P84103;B4E241	Serine/arginine-rich splicing	SRSF3;SFRS3	-0.4239712	0.1984636	1.66E-33
P62310	U6 snRNA-associated Sm-lik	LSM3	0.4605885	0.199247556	0.0023264
P08670;B0YJC4	Vimentin	VIM	0.4845448	0.199410737	3.68E-247
Q8IUE6	Histone H2A type 2-B	HIST2H2AB	0.4830246	0.200902967	3.41E-160
P61619;P61619-3;B4DR61	Protein transport protein Se	SEC61A1	0.4576988	0.201859345	1.34E-05
P61247;D6RG13;D6RAT0;F5H	40S ribosomal protein S3a	RPS3A	0.4811516	0.202752758	8.13E-67
Q9P287;Q9P287-3;Q9P287-2	BRCA2 and CDKN1A-interac	BCCIP	-0.4192715	0.203197805	4.09E-85
Q12800;Q12800-4;F8VWL0;F	Alpha-globin transcription f	TFCP2	-0.6719646	0.205274963	1.35E-65
Q9UBS4	DnaJ homolog subfamily B r	DNAJB11	0.4530163	0.206144496	2.06E-24
Q9P258	Protein RCC2	RCC2	-0.4151173	0.20745052	4.09E-82
P46778;G3V1B3;M0R181	60S ribosomal protein L21	RPL21	0.4760036	0.207900705	8.37E-17
Q13151	Heterogeneous nuclear ribo	HNRNPA0	0.4755096	0.208399649	4.93E-75
O43823	A-kinase anchor protein 8	AKAP8	-0.6625824	0.212070476	3.38E-06
Q07065	Cytoskeleton-associated pro	CKAP4	0.4463921	0.21231883	4.35E-63
P49411	Elongation factor Tu, mitoch	TUFM	-0.6588707	0.214803231	1.36E-29
Q9NQG5	Regulation of nuclear pre-m	RPRD1B	-0.4071712	0.215764109	5.32E-146
P60709	Actin, cytoplasmic 1;Actin, c	ACTB	0.467411	0.216703331	1.63E-229
Q92522	Histone H1x	H1FX	0.440897	0.217541199	1.32E-26
J3KN66;Q5JTV8;Q5JTV8-2;H0	Torsin-1A-interacting protei	TOR1AIP1	0.4659004	0.218278194	1.05E-113
C9J3L7;C9JZE3;C9JYJ8;Q9BWI	Replication initiator 1	REPIN1	-0.6502972	0.221212349	3.74E-12
Q15424;B7ZLP6;A0AV56;Q15	Scaffold attachment factor I	SAFB	-0.4018555	0.221458126	4.89E-275
P05783;F8VZY9	Keratin, type I cytoskeletal 1	KRT18	0.4608212	0.223633748	3.70E-154
P62633;P62633-2;B4DP17;P6	Cellular nucleic acid-binding	CNBP	0.4329948	0.225212137	3.77E-63
Q14865;Q14865-3	AT-rich interactive domain-c	ARID5B	-0.637907	0.230714944	1.10E-15

Q99714;Q99714-2;Q5H928	3-hydroxyacyl-CoA dehydro	HSD17B10	-0.3933125	0.230833189	2.97E-194
P26373;J3QSB4;P26373-2	60S ribosomal protein L13	RPL13	0.4532719	0.231766271	1.94E-22
Q9UNF0;Q9UNF0-2;B0QYG7;	Protein kinase C and casein	PACSIN2	-0.3918037	0.232517723	1.52E-112
Q14444;Q14444-2;G3V153;E	Caprin-1	CAPRIN1	-0.3912373	0.233152486	1.05E-77
Q8IWS0;E9PC97;B4E0G4;Q5J	PHD finger protein 6	PHF6	0.4244614	0.233710796	1.80E-42
Q9H0E9-2;Q9H0E9;H7C128;H	Bromodomain-containing p	BRD8	-0.6337299	0.23398304	7.88E-21
Q5VT52;Q5VT52-3;Q5VT52-2	Regulation of nuclear pre-m	RPRD2	-0.3904381	0.234050056	2.94E-155
P40429;M0QYS1;Q6NVV1	60S ribosomal protein L13a,	RPL13A	0.4234924	0.234689956	3.20E-11
P08238;P07900;P07900-2	Heat shock protein HSP 90-l	HSP90AB1;HSP90AA	-0.6322613	0.235139848	7.79E-34
P49756;E9PQU5;P49756-2;P4	RNA-binding protein 25	RBM25	0.4212399	0.236977531	2.09E-05
Q9Y3B3;Q6JUT2;Q9Y3B3-2	Transmembrane emp24 dor	TMED7;TICAM2	0.4472485	0.23840402	4.34E-59
Q8TF01	Arginine/serine-rich protein	PNISR	-0.6281147	0.238427788	0.0088198
P05198;H0YJS4;G3V4T5	Eukaryotic translation initia	EIF2S1	0.4196358	0.238616109	2.29E-33
P62249;M0R3H0;M0R210;Q6	40S ribosomal protein S16	RPS16	0.4451256	0.240775087	9.58E-15
P35637;P35637-2;H3BPE7	RNA-binding protein FUS	FUS	0.4446545	0.241303524	3.30E-47
Q7L014	Probable ATP-dependent RI	DDX46	-0.3833199	0.242152779	9.32E-92
Q99873;E9PKG1;Q99873-3;Q	Protein arginine N-methyltr	PRMT1	-0.3828239	0.242724542	2.11E-24
Q9NQT4;M0R050	Exosome complex compone	EXOSC5	0.4136143	0.244838398	9.68E-05
Q8N9T8;H0YFD2;D3YTE0;Q8I	Protein KRI1 homolog	KRI1	0.412693	0.24580032	5.23E-39
Q9Y2X3	Nucleolar protein 58	NOP58	0.4120502	0.246473042	4.60E-23
P63261;I3L3I4;I3L4N8;I3L3I0;	Actin, cytoplasmic 2;Actin, c	ACTG1	0.439436	0.247211646	2.91E-282
Q07955;J3KTL2;Q07955-3;Q0	Serine/arginine-rich splicing	SRSF1	0.4385777	0.248192995	3.10E-113
P31689;B7Z5C0	DnaJ homolog subfamily A r	DNAJA1	-0.3728123	0.254470983	3.04E-51
Q92733;A6NG79	Proline-rich protein PRCC	PRCC	-0.371582	0.255941223	7.94E-60
O76094;O76094-2	Signal recognition particle s	SRP72	0.4318199	0.256014731	0
P62258;P62258-2	14-3-3 protein epsilon	YWHAE	0.4310131	0.256959889	2.14E-186
Q8N2W9	E3 SUMO-protein ligase PIA	PIAS4	0.4283409	0.26010759	1.88E-80
P52298;E9PAR5;C9JQX9;B3KS	Nuclear cap-binding protein	NCBP2	0.4282856	0.260173027	4.27E-19
P84090;G3V279	Enhancer of rudimentary hc	ERH	0.4273643	0.261264559	5.40E-90
P54727;H0Y579;P54727-2	UV excision repair protein R	RAD23B	0.3981953	0.261287863	4.60E-07
Q8N4Q1;Q8N4Q1-2	Mitochondrial intermembra	CHCHD4	-0.366991	0.261479897	5.16E-81
Q15293;B7Z1M1;E9PJD5;E9P	Reticulocalbin-1	RCN1	-0.3663731	0.262231723	2.94E-115
P35221;G3XAM7;P35221-2;E	Catenin alpha-1	CTNNA1	-0.5972214	0.263946889	1.22E-42
O00139;O00139-2;O00139-1;	Kinesin-like protein KIF2A	KIF2A	-0.59408	0.266643558	2.16E-26
Q5T8D3-3;Q5T8D3-4;Q5T8D3	Acyl-CoA-binding domain-cc	ACBD5	-0.5909882	0.269316106	1.39E-11
O95400	CD2 antigen cytoplasmic tai	CD2BP2	0.3878021	0.272797576	3.94E-23
Q9NY12;Q9NY12-2	H/ACA ribonucleoprotein cc	GAR1	0.4174175	0.273251705	3.87E-21
P14635;H0YA62;Q5TZP9;E9P	G2/mitotic-specific cyclin-B	CCNB1	0.3862495	0.274546286	0.0010346
P49321;P49321-2;P49321-3;E	Nuclear autoantigenic sperr	NASP	0.4151039	0.276092971	7.06E-64
O60884	DnaJ homolog subfamily A r	DNAJA2	0.3835926	0.277556584	1.45E-54
Q96IZ0	PRKC apoptosis WT1 regula	PAWR	0.412981	0.278717668	4.90E-22
O95831;O95831-3;O95831-2	Apoptosis-inducing factor 1	AIFM1	-0.3529091	0.278983098	1.08E-86

Q13445	Transmembrane emp24 dor	TMED1	0.3818378	0.279557003	1.25E-55
P62314;J3QLI9	Small nuclear ribonucleopro	SNRPD1	0.4118462	0.280127749	1.33E-86
P41208	Centrin-2	CETN2	0.3791542	0.282635254	1.46E-12
O43865;O43865-2;Q96HN2;C	Putative adenosylhomocyst	AHCYL1;AHCYL2	0.3787689	0.28307907	6.02E-11
Q9BW71;Q9BW71-2	HIRA-interacting protein 3	HIRIP3	0.4087849	0.283955553	7.05E-149
P10412;P16403;P16402	Histone H1.4;Histone H1.2;I	HIST1H1E;HIST1H1C	0.4067669	0.28649808	2.94E-11
P55081	Microfibrillar-associated pro	MFAP1	-0.3461151	0.287706448	8.04E-85
Q14181;E9PIQ6;B4DNB4	DNA polymerase alpha subu	POLA2	-0.5695744	0.288330209	0.00071899
P55072	Transitional endoplasmic re	VCP	-0.3447132	0.289529124	0
P02545-2;Q6UYC3;Q5TCI8	Prelamin-A/C;Lamin-A/C	LMNA	-0.5677166	0.29002143	1.55E-224
P60842;J3KSZ0;J3KT12;P6084	Eukaryotic initiation factor 4	EIF4A1	-0.5673256	0.290378234	4.62E-70
Q9Y2D5-4;C9JVY5;Q9Y2D5;Q	A-kinase anchor protein 2	AKAP2	-0.5632477	0.294117042	1.79E-37
P06576;H0YH81;F8VPV9	ATP synthase subunit beta,	ATP5B	0.3691788	0.294278391	2.91E-42
P25440;H0Y6K2;H0Y5T9;P254	Bromodomain-containing p	BRD2;DKFZp313H13	0.4006195	0.294337948	2.72E-234
Q92541	RNA polymerase-associated	RTF1	0.3685131	0.295066626	1.09E-15
P62266	40S ribosomal protein S23	RPS23	-0.5622082	0.295075246	1.04E-08
Q15059;Q15059-2	Bromodomain-containing p	BRD3	0.3979549	0.297780384	4.78E-162
P21127;Q9UQ88;P21127-12;(	Cyclin-dependent kinase 11	CDK11B;CDK11A	0.3654671	0.298691568	3.21E-07
P36873;F8VYE8;P36873-2;F8	Serine/threonine-protein ph	PPP1CC	-0.5580387	0.298939609	2.34E-191
Q13206;E9PIF2	Probable ATP-dependent RI	DDX10	0.3647537	0.299544768	0.0044254
Q9Y305;C9J7L8;Q9Y305-3;Q9	Acyl-coenzyme A thioestera	ACOT9	-0.556572	0.300307026	5.82E-06
P12956;B1AHC9;F5H1I8;B1AH	X-ray repair cross-complem	XRCC6	0.3941078	0.302797884	6.92E-64
P60866;P60866-2;E5RIP1;E5F	40S ribosomal protein S20	RPS20	0.3931141	0.30410301	1.01E-28
Q14011;K7ELV6;K7ENX8;K7E	Cold-inducible RNA-binding	CIRBP	0.391613	0.306081551	2.78E-126
Q9NWH9;H0YL55;H7BXE3;H0	SAFB-like transcription mod	SLTM	-0.5493584	0.307092684	6.21E-82
Q03111	Protein ENL	MLLT1	0.3902416	0.307896581	5.79E-29
Q9BRX2	Protein pelota homolog	PELO	-0.330658	0.308232428	4.25E-178
P50914;E7EPB3	60S ribosomal protein L14	RPL14	0.3882675	0.310521796	1.95E-22
P17028	Zinc finger protein 24	ZNF24	-0.545742	0.310532403	3.45E-07
Q06546	GA-binding protein alpha ch	GABPA	-0.5438976	0.312296488	4.13E-66
P52597	Heterogeneous nuclear ribo	HNRNPF	-0.5437832	0.312406162	1.86E-126
Q15363;F5GX39;E7EQ72	Transmembrane emp24 dor	TMED2	0.385397	0.314365422	4.44E-49
Q2KHR3;Q2KHR3-2;H0YCQ7	Glutamine and serine-rich p	QSER1	0.3521557	0.314880435	3.91E-104
Q15287;H3BV80;H3BMM9;H	RNA-binding protein with se	RNPS1	0.3848248	0.315135317	1.90E-41
P51571	Translocon-associated prote	SSR4	0.3846321	0.315394793	6.69E-99
Q9BZZ5;Q9BZZ5-2;Q9BZZ5-1;	Apoptosis inhibitor 5	API5	-0.5386925	0.317310509	9.74E-20
Q92804;K7EPT6;Q92804-2	TATA-binding protein-assoc	TAF15	0.3501968	0.317310509	1.72E-51
O60220	Mitochondrial import inner	TIMM8A	-0.3240395	0.317310509	7.23E-34
O60885;O60885-2;Q4G0X8;M	Bromodomain-containing p	BRD4	0.383213	0.317310509	1.58E-121
O00193;E9PM92;E9PRZ9;E3V	Small acidic protein	SMAP;C11orf58	0.3500328	0.317514557	5.91E-79
P40938;P40938-2	Replication factor C subunit	RFC3	-0.5384102	0.317583931	4.14E-16
Q9Y6A4	UPF0468 protein C16orf80	C16orf80	0.382988	0.317615045	8.06E-86

Q86XP3;Q86XP3-2	ATP-dependent RNA helicas	DDX42	-0.3236046	0.31791307	5.59E-295
P31944	Caspase-14;Caspase-14 sub	CASP14	-0.5374527	0.318512501	7.60E-20
P78344;P78344-2;H0Y3P2;D3	Eukaryotic translation initia	EIF4G2	-0.3211098	0.321384348	3.41E-232
Q96JB5;J3QQY1;F5H3I5;J3QS	CDK5 regulatory subunit-as	CDK5RAP3	-0.3206539	0.322021291	1.17E-39
P23396;J3KN86;E9PPU1;E9PL	40S ribosomal protein S3	RPS3	0.3797054	0.322078392	7.50E-126
P05386	60S acidic ribosomal protei	RPLP1	0.378643	0.323531687	1.39E-19
P62424;Q5T8U2;Q5T8U3	60S ribosomal protein L7a	RPL7A	0.3773212	0.325345768	1.50E-96
P29084	Transcription initiation fact	GTF2E2	0.3770981	0.325652692	1.71E-78
Q16576;E9PC52;Q16576-2	Histone-binding protein RBI	RBBP7	-0.3180161	0.325723206	6.02E-44
O14579;O14579-2;M0QXB4;I	Coatomer subunit epsilon	COPE	-0.3176956	0.326174772	1.26E-96
P49736;H0Y8E6	DNA replication licensing fa	MCM2	-0.3170433	0.327095289	2.51E-103
P27635;B8A6G2;F8W7C6;A6(	60S ribosomal protein L10	RPL10	0.3377075	0.333092636	1.64E-21
P04217;P04217-2;M0R009	Alpha-1B-glycoprotein	A1BG	0.3370037	0.333996838	9.48E-25
Q13148;Q13148-2;B1AKP7;G	TAR DNA-binding protein 43	TARDBP	-0.5188313	0.336924627	1.54E-45
Q4G0J3;D6R9Z6;Q4G0J3-2;H(	La-related protein 7	LARP7	0.333622	0.338363468	1.43E-39
Q8WWI1-3;E9PMS6;Q8WWI1	LIM domain only protein 7	LMO7	0.3330173	0.339148037	2.68E-113
O75494;O75494-3;O75494-4,	Serine/arginine-rich splicing	SRSF10;SRSF12	-0.5158157	0.339969394	2.67E-13
Q9UJV9;H0Y8L8;J3KNN5	Probable ATP-dependent RI	DDX41	0.3285542	0.344975552	1.32E-10
Q9Y3I0;E7EQS9	tRNA-splicing ligase RtcB ho	C22orf28;RTCB	-0.3044147	0.345248084	6.45E-104
Q9Y5J9;G3XAN8	Mitochondrial import inner	TIMM8B	-0.3029842	0.347344116	1.82E-18
Q9NX40;D6RDI5;D6RA54;D6I	OCIA domain-containing pr	OCIAD1	0.3266792	0.347442543	5.88E-21
P62906	60S ribosomal protein L10a	RPL10A	0.3614731	0.347610289	2.03E-101
Q9UBD5;Q9UBD5-2;Q9UBD5	Origin recognition complex	ORC3	-0.5054569	0.35056254	1.74E-17
Q16698;B7Z6B8	2,4-dienoyl-CoA reductase,	DECR1	0.323719	0.351360312	2.10E-19
P62861;E9PR30	40S ribosomal protein S30	FAU	0.3588505	0.351386037	8.08E-07
Q15365	Poly(rC)-binding protein 1	PCBP1	-0.5034504	0.352638407	5.12E-22
Q15393;Q15393-3	Splicing factor 3B subunit 3	SF3B3	-0.5030422	0.353061634	1.73E-29
Q53F19;Q53F19-2	Uncharacterized protein C1	C17orf85	-0.5027294	0.353386194	4.72E-22
P08574	Cytochrome c1, heme prote	CYC1	-0.2982655	0.354315475	1.16E-41
Q9HC35;B5MCW9;B5MBZ0;F	Echinoderm microtubule-as	EML4	-0.4995117	0.356735749	3.41E-36
P60510;H3BV22;H3BTA2	Serine/threonine-protein pl	PPP4C	-0.4976902	0.358640748	0.00071779
Q9Y2K7;D4QA03;E9PIL6;Q9Y	Lysine-specific demethylase	KDM2A	0.3181076	0.358863386	8.23E-15
Q12906;Q12906-4;Q12906-5	Interleukin enhancer-bindin	ILF3	0.3527126	0.360323779	8.73E-73
P61224;P62834;A6NIZ1;F5H8	Ras-related protein Rap-1b;	RAP1B;RAP1A	0.3163395	0.36124826	2.21E-17
P18846;B4DRF9;P16220;Q03	Cyclic AMP-dependent tran	ATF1;CREB1;CREM	-0.2932644	0.361799635	2.09E-17
P29083	General transcription factor	GTF2E1	-0.292635	0.362748548	1.02E-72
Q13263;Q13263-2;M0R0K9	Transcription intermediary	TRIM28	-0.4900723	0.366676969	1.53E-23
P06748;P06748-2;P06748-3	Nucleophosmin	NPM1	0.3477554	0.36764551	0
P55769;B1AHD1	NHP2-like protein 1;NHP2-li	NHP2L1	-0.2885303	0.368974759	3.39E-24
Q9H1E5	Thioredoxin-related transm	TMX4	0.309948	0.369951945	1.46E-12
Q9NS91;F8WE49	E3 ubiquitin-protein ligase F	RAD18	0.3449841	0.371778902	3.07E-62
Q9UHB7;Q9UHB7-2	AF4/FMR2 family member 4	AFF4	0.3446865	0.372224387	2.07E-42

O15156;O15156-2	Zinc finger and BTB domain	ZBTB7B	-0.4843826	0.372751636	1.94E-08
Q6ZW49;Q6ZW49-4;F8WC23	PAX-interacting protein 1	PAXIP1	0.3064976	0.37470423	3.19E-25
Q99733;F5HFY4;C9JZI7;A8M)	Nucleosome assembly prot	NAP1L4;NAP1L4b	0.3429108	0.37488989	1.87E-121
P13861;Q9BUB1;H7C1L0	cAMP-dependent protein ki	PRKAR2A	-0.481617	0.375726844	1.13E-35
Q08211	ATP-dependent RNA helicas	DHX9	0.3056812	0.375834092	1.36E-24
Q9BRK5;Q9BRK5-4;H0Y3T6;C	45 kDa calcium-binding pro	SDF4	-0.4812336	0.376140424	6.44E-32
Q13724;F5H6D0;C9J8D4	Mannosyl-oligosaccharide g	MOGS	-0.2834835	0.376720727	8.65E-41
Q9Y2Q9;E5RGC7;E5RK86;E5R	28S ribosomal protein S28,	MRPS28	-0.4802361	0.37721787	0.0003874
Q9NXV6	CDKN2A-interacting protein	CDKN2AIP	-0.479454	0.378063851	6.05E-16
P43487;C9JJ34;C9JXG8;C9JG\	Ran-specific GTPase-activat	RANBP1	-0.281805	0.37931893	6.65E-16
P49458;E9PE20;P49458-2	Signal recognition particle 9	SRP9	-0.2802486	0.381737996	4.91E-33
Q9Y265;Q9Y265-2;H7C4G5;E	RuvB-like 1	RUVBL1	-0.2787018	0.384151595	4.68E-150
Q00325-2;F8VVM2;Q00325;F	Phosphate carrier protein, r	SLC25A3	0.3364906	0.38462499	3.59E-20
P46781;B5MCT8;C9JM19	40S ribosomal protein S9	RPS9	0.298727	0.385544118	1.77E-16
P38159;Q96E39;H3BNC1;H3E	RNA-binding motif protein,	RBMX;RBMXL1	0.2976665	0.387038212	4.08E-46
Q14974;B7ZAV6;F5H4R7;J3K	Importin subunit beta-1	KPNB1	0.3349133	0.387040283	0
O43681;K7ERW9	ATPase ASNA1	ASNA1	-0.4700603	0.388317058	1.07E-07
O95218;O95218-2	Zinc finger Ran-binding dom	ZRANB2	0.3322239	0.391179528	2.47E-196
P80723	Brain acid soluble protein 1	BASP1	0.2922058	0.394787486	1.05E-06
P22087;M0R299;M0QXL5;M(	rRNA 2-O-methyltransferas	FBL	0.3295898	0.395259586	1.09E-11
O00410;O00410-3;B4E0R6;O	Importin-5	IPO5	0.28969	0.398388971	1.64E-33
P50991;B7Z9L0;P50991-2	T-complex protein 1 subuni	CCT4	0.2890854	0.399257469	9.40E-27
O75400;O75400-2;O75400-3	Pre-mRNA-processing facto	PRPF40A	-0.4593906	0.400166281	4.92E-12
O75643	U5 small nuclear ribonuclec	SNRNP200	-0.2681751	0.400823324	1.15E-92
O14980;C9JF49;C9IYM2;F8W	Exportin-1	XPO1	0.2873535	0.401751452	0.00014908
Q9NRF9	DNA polymerase epsilon sul	POLE3	0.2841568	0.406379365	6.05E-11
Q9HCG8;B7WP27;B7WP74	Pre-mRNA-splicing factor C\	CWC22	0.2834377	0.407424736	1.02E-32
O15355	Protein phosphatase 1G	PPM1G	0.3214588	0.4080156	1.79E-106
P47712;E7EU42	Cytosolic phospholipase A2	PLA2G4A	-0.4521942	0.408279623	2.88E-23
P62851	40S ribosomal protein S25	RPS25	0.3206387	0.409315751	2.73E-28
P62753;A2A3R7;A2A3R5	40S ribosomal protein S6	RPS6	0.3196239	0.410927716	6.23E-49
P62280;M0QZC5;M0R1H5	40S ribosomal protein S11	RPS11	0.3176613	0.414056272	3.11E-32
O96019;O96019-2;H7C5S0	Actin-like protein 6A	ACTL6A	0.3174152	0.414449473	4.43E-50
Q6PD62	RNA polymerase-associated	CTR9	-0.4448795	0.416625877	3.83E-07
P13667	Protein disulfide-isomerase	PDIA4	-0.2575932	0.418013265	1.38E-237
P11940;P11940-2;E7EQV3;E7	Polyadenylate-binding prot	PABPC1	0.3141422	0.419700941	7.83E-186
P62158;H0Y7A7;F8WBR5;M0	Calmodulin	CALM1;CALM2;CALI	0.3138161	0.420226386	2.17E-16
P52272;P52272-2;M0R0Y6;M	Heterogeneous nuclear ribc	HNRNPM	-0.4375553	0.425083023	5.33E-12
P55199	RNA polymerase II elongati	ELL	0.2711163	0.425585336	8.55E-24
P35659;P35659-2;B4DFG0;D	Protein DEK	DEK	-0.2525406	0.42637179	4.63E-186
Q9NV56	MRG/MORF4L-binding prot	MRGBP	-0.4352856	0.427724071	5.69E-09
Q9BRP8;Q9BRP8-2	Partner of Y14 and mago	WIBG	0.2690601	0.428661207	1.80E-10

P17480;P17480-2;E9PKP7	Nucleolar transcription fact	UBTF	0.3072376	0.430906641	1.96E-151
Q9BZK7;O60907;Q9BQ87;C9J	F-box-like/WD repeat-conta	TBL1XR1;TBL1X;TBL	0.2673988	0.43115586	1.67E-14
P39019	40S ribosomal protein S19	RPS19	0.3068314	0.431571344	4.19E-26
Q9H3Q1;B3KUS7	Cdc42 effector protein 4	CDC42EP4	-0.4310379	0.432692193	2.87E-168
Q99613;H3BRV0;B5ME19	Eukaryotic translation initia	EIF3C;EIF3CL	0.2660561	0.433178342	1.45E-81
P08865;C9J9K3;A6NE09	40S ribosomal protein SA	RPSA;RPSAP58	0.3056316	0.433537712	3.48E-92
P62826;B5MDF5;F5H018;J3K	GTP-binding nuclear protein	RAN	0.3051262	0.434367699	2.79E-17
Q9H814	Phosphorylated adapter RN	PHAX	-0.4256477	0.43904444	1.53E-10
P98179	Putative RNA-binding prote	RBM3	0.2617512	0.43969924	6.55E-05
P49770	Translation initiation factor	EIF2B2	-0.42272	0.44251712	4.55E-11
P32119;A6NIW5	Peroxiredoxin-2	PRDX2	-0.4226837	0.442560203	3.86E-15
O75607	Nucleoplasmin-3	NPM3	0.2992916	0.4440148	6.80E-84
P39880;P39880-6;P39880-4;F	Homeobox protein cut-like	CUX1	-0.2399731	0.447580231	8.48E-69
Q14919;E9PQX9;Q14919-2;C	Dr1-associated corepressor	DRAP1	-0.417902	0.448265833	4.13E-17
Q9H4L4;J3KNH7	Sentrin-specific protease 3	SENP3	0.2555714	0.449157982	9.78E-07
Q13428;Q13428-3;Q13428-2	Treacle protein	TCOF1	0.2960491	0.449428501	1.51E-243
Q9H2P0	Activity-dependent neuropr	ADNP	-0.2381382	0.450726082	5.05E-188
Q96EY4;D6RA57;H0Y9X1	Translation machinery-asso	TMA16	-0.4149628	0.451793595	4.61E-20
P48681	Nestin	NES	-0.4143543	0.452525828	1.04E-16
P63208;E5RJR5;E5RGM3;E7E	S-phase kinase-associated p	SKP1	-0.2369156	0.452829157	2.41E-52
Q8N9E0;G3XAI9;Q5BKY9	Protein FAM133A;Protein F.	FAM133A;FAM133E	-0.4140778	0.452858883	5.24E-09
P13639	Elongation factor 2	EEF2	-0.413353	0.453732372	5.75E-34
P62304;A6NHK2	Small nuclear ribonucleopro	SNRPE	-0.2359886	0.454427375	4.45E-93
Q9UDW1	Cytochrome b-c1 complex s	UQCR10	-0.4125919	0.454650558	9.60E-11
Q9NUP9;G3V1D4	Protein lin-7 homolog C	LIN7C	-0.4100437	0.457732594	1.46E-07
P62829;C9JD32;B9ZVP7;J3KT	60S ribosomal protein L23	RPL23	-0.2339287	0.457990327	1.47E-62
Q9Y232;Q9Y232-2;Q9Y232-3;	Chromodomain Y-like prote	CDYL	0.249836	0.458038866	1.14E-22
P62244;I3L3P7;I3L246;H3BN	40S ribosomal protein S15a	RPS15A	0.290369	0.459001667	5.14E-38
Q8NEF9	Serum response factor-bind	SRFBP1	-0.4085522	0.459542027	4.47E-14
Q12874;E7EUT8	Splicing factor 3A subunit 3	SF3A3	-0.2325878	0.460317942	4.23E-39
Q96EP5;K7EQ02;Q96EP5-2	DAZ-associated protein 1	DAZAP1	0.2478008	0.461213671	1.79E-44
Q96AX1;F5H2X5;H3BMM5	Vacuolar protein sorting-as	VPS33A	0.2475052	0.461675886	0.00084182
Q12905	Interleukin enhancer-bindin	ILF2	0.2887573	0.461738695	1.09E-23
Q9BVK6	Transmembrane emp24 dor	TMED9	0.2856293	0.467076715	5.53E-78
P21333;Q5HY54;P21333-2	Filamin-A	FLNA	0.2827721	0.4719823	8.01E-59
O00505	Importin subunit alpha-4	KPNA3	0.2824554	0.472527651	8.18E-65
Q14157;Q14157-1;Q14157-4	Ubiquitin-associated protei	UBAP2L	0.2821465	0.473060196	5.68E-103
Q86VM9;E7ERS3;Q86VM9-2	Zinc finger CCCH domain-co	ZC3H18	0.2401867	0.473200064	2.97E-20
Q8N556;Q8N556-2	Actin filament-associated pı	AFAP1	-0.2234402	0.476372723	4.47E-53
Q15024	Exosome complex compone	EXOSC7	0.2376785	0.477185694	0.00050345
Q14331;E9PRR7	Protein FRG1	FRG1	0.2369328	0.478374308	7.86E-23
Q14318;Q14318-2;J3KQ73;C8	Peptidyl-prolyl cis-trans isor	FKBP8	-0.2222805	0.478429712	6.23E-73

P35251;P35251-2	Replication factor C subunit	RFC1	0.2349033	0.481616976	1.40E-07
P07437;Q5JP53;F8VYX6;F8VW	Tubulin beta chain	TUBB	-0.2202225	0.48209211	0
O15160;O15160-2;E7EQB9	DNA-directed RNA polymer	POLR1C	-0.3899612	0.482428224	2.90E-27
Q04637;Q04637-3;Q04637-6	Eukaryotic translation initia	EIF4G1	-0.3898659	0.482547201	1.13E-18
Q9H1E3	Nuclear ubiquitous casein a	NUCKS1	-0.2191544	0.483998884	4.83E-178
P09132;P09132-2	Signal recognition particle 1	SRP19	0.2332954	0.484194587	9.05E-21
Q9Y3A6;M0R072;B1AKT3;B1	Transmembrane emp24 do	TMED5	-0.3874454	0.485572166	9.50E-29
P62273;P62273-2	40S ribosomal protein S29	RPS29	-0.3854198	0.488111565	8.80E-05
Q92791;K7ERA3	Synaptonemal complex pro	LEPREL4	-0.2162933	0.489126403	1.03E-59
Q9BW61	DET1- and DDB1-associated	DDA1	0.2299957	0.489507691	3.01E-10
P51648;P51648-2;J3QKK9;K7	Fatty aldehyde dehydrogen	ALDH3A2	-0.3821545	0.492220244	5.18E-24
P22626;P22626-2	Heterogeneous nuclear ribo	HNRNPA2B1	0.2704678	0.493428669	9.30E-114
Q14566	DNA replication licensing fa	MCM6	-0.2121143	0.496668276	4.57E-182
Q9Y5M8;H7C4H2	Signal recognition particle r	SRPRB	-0.2120609	0.496765057	8.91E-72
Q9Y224;G3V4C6	UPF0568 protein C14orf166	C14orf166	-0.2105179	0.499565682	7.18E-102
Q7Z7H5;Q7Z7H5-3;Q7Z7H5-2	Transmembrane emp24 do	TMED4	0.2666111	0.500256665	2.23E-99
Q9Y3E5;J3KQ48	Peptidyl-tRNA hydrolase 2,	PTRH2	-0.3756371	0.500475956	5.42E-143
Q9NYF8-3;E9PK91;E9PK09;E9	Bcl-2-associated transcripti	BCLAF1	0.2205734	0.504850422	5.24E-222
Q06830	Peroxiredoxin-1	PRDX1	0.2637482	0.505357549	6.92E-19
Q7KZ85;Q7KZ85-3	Transcription elongation fac	SUPT6H	-0.3702011	0.507417513	6.12E-109
Q13347;Q5TFK1	Eukaryotic translation initia	EIF3I	0.2185497	0.508178447	4.00E-06
Q8IYT4;K7EM02;K7EIJ8;Q8IYT	Katanin p60 ATPase-contair	KATNAL2	-0.3695984	0.508190275	0.002077
Q9BTC0;Q9BTC0-2;Q9BTC0-3	Death-inducer obliterator 1	DIDO1	-0.3689537	0.50901752	1.13E-11
Q9H211	DNA replication factor Cdt1	CDT1	0.2175293	0.509860928	3.33E-20
Q00839;Q00839-2	Heterogeneous nuclear ribo	HNRNPU	0.2609825	0.510311006	3.71E-164
Q5QJE6;J3KP30	Deoxynucleotidyltransferas	DNTTIP2	0.2605724	0.51104764	4.56E-100
P08579	U2 small nuclear ribonuclec	SNRPB2	-0.2030525	0.513232839	2.19E-65
P54105;E9PMI6;E9PJF4;J3KN	Methylosome subunit pICln	CLNS1A	-0.3649216	0.514207433	1.66E-11
Q13283;F5H4D6	Ras GTPase-activating prote	G3BP1	0.2574844	0.516612454	3.44E-148
P62277;J3KMX5	40S ribosomal protein S13	RPS13	-0.1982784	0.522073966	1.04E-15
Q9UNL2;C9JA28;B4E2P2	Translocon-associated prote	SSR3	0.2079678	0.525766433	0.0025334
Q9H6Y2;G3V1J0	WD repeat-containing prote	WDR55	-0.3550091	0.527082179	1.04E-27
Q96B36;Q96B36-2;J3KPM3	Proline-rich AKT1 substrate	AKT1S1	0.2068634	0.527619723	0.00017335
P41091;Q2VIR3;Q2VIR3-2;F8	Eukaryotic translation initia	EIF2S3;EIF2S3L	0.2066727	0.527940145	2.28E-09
Q9H3P2;B3KSP0;H0Y3X6;C9J	Negative elongation factor	NELFA;WHSC2	-0.3536434	0.528868762	1.22E-34
Q5C9Z4	Nucleolar MIF4G domain-co	NOM1	-0.3535042	0.529051087	2.22E-11
O15446;O15446-2	DNA-directed RNA polymer	CD3EAP	0.2500954	0.530054484	8.96E-113
P46100;P46100-2;P46100-5;F	Transcriptional regulator AT	ATRX	0.2053833	0.530108794	6.81E-38
Q07666;Q07666-3;Q07666-2	KH domain-containing, RNA	KHDRBS1	-0.3515911	0.531559412	2.94E-20
Q9BQ15;Q9BQ15-2;C9JT95;C	SOSS complex subunit B1	NABP2	0.2483025	0.533342687	8.83E-66
P14927;P14927-2;B7Z2R2	Cytochrome b-c1 complex s	UQCRB	-0.1918335	0.5341323	7.81E-54
O95373	Importin-7	IPO7	0.2474327	0.534941521	7.08E-195

Q9Y230;B3KQ59;M0R0Y3	RuvB-like 2	RUVBL2	-0.1912613	0.53520964	4.35E-81
P62140;E7ETD8	Serine/threonine-protein pl	PPP1CB	0.2471714	0.535422345	9.39E-171
F8W9Q2;Q8N7H5-2;M0QX35	RNA polymerase II-associat	PAF1	0.2018318	0.536105511	2.30E-23
Q6UWP8	Suprabasin	SBSN	0.2017288	0.53627993	9.13E-06
Q92945;M0R0I5	Far upstream element-bind	KHSRP	-0.3452644	0.539897348	1.49E-18
Q9Y6E0-2;B4DR80;Q9Y6E0;H	Serine/threonine-protein ki	STK24	0.1982746	0.54214593	4.23E-56
Q92688;Q92688-2;Q5T6W8	Acidic leucine-rich nuclear p	ANP32B	0.2421227	0.544754958	1.33E-118
Q66PJ3;Q66PJ3-2;Q66PJ3-3;F	ADP-ribosylation factor-like	ARL6IP4	-0.1860294	0.545110643	3.04E-77
P40227;B4DPJ8	T-complex protein 1 subuni	CCT6A	0.1958771	0.546236202	1.10E-09
P26599;P26599-2;P26599-3;H	Polypyrimidine tract-bindin	PTBP1	0.241003	0.546835457	1.77E-134
Q01130;J3QL05;J3KP15;B4DN	Serine/arginine-rich splicing	SRSF2;SFRS2	-0.1842461	0.548506236	5.81E-90
P15408;C9JCN8;P15408-3;P1	Fos-related antigen 2	FOSL2	-0.3385544	0.548811492	0.00056285
Q13427;C9JN15;Q13427-2;C9	Peptidyl-prolyl cis-trans isor	PPIG	-0.3380909	0.549429894	2.26E-40
P09651;P09651-3;F8VXY0;F8	Heterogeneous nuclear ribo	HNRNPA1;HNRNPA1	0.2373638	0.553625017	2.26E-147
P05388;Q3B7A4;F8VWS0;F8V	60S acidic ribosomal protein	RPLP0;RPLP0P6	-0.181551	0.553657495	2.18E-70
O43684;J3QT28;O43684-2;B4	Mitotic checkpoint protein I	BUB3	0.236496	0.555250197	1.08E-27
O60828;O60828-4;O60828-2	Polyglutamine-binding prot	PQBP1	-0.1803055	0.556046064	2.49E-31
P84098;J3QR09;J3KTE4;J3QL	60S ribosomal protein L19;F	RPL19	0.2351532	0.557769346	3.09E-37
P60468	Protein transport protein Se	SEC61B	0.2348785	0.55828531	0.0025644
Q13098;Q13098-6;J3QLT0;J3	COP9 signalosome complex	GPS1	-0.3310375	0.558882971	1.28E-05
Q8WXI9	Transcriptional repressor p6	GATAD2B	-0.3286285	0.562129537	2.27E-34
Q9NP74;Q9NP74-2;Q9NP74-3	Palmdelphin	PALMD	-0.1770725	0.562269463	4.77E-36
Q9NX24;D6RC52;D6RCB9;J3C	H/ACA ribonucleoprotein co	NHP2	0.2326717	0.562439343	1.42E-121
E9PAV3;F8VZJ2;F8W0W4;H0	Nascent polypeptide-associ	NACA	-0.1762276	0.563901519	9.64E-45
O60573;B9A044;B8ZZJ9;B8ZZ	Eukaryotic translation initia	EIF4E2	0.1843147	0.566174024	2.49E-11
P18754;C9JW69;P18754-2;H7	Regulator of chromosome c	RCC1	0.1843147	0.566174024	1.31E-36
P19338;H7BY16	Nucleolin	NCL	0.2302742	0.566969258	0
O76071	Probable cytosolic iron-sulfu	CIAO1	-0.3244419	0.567793411	0.0013845
E5RGW4		NPM1	0.1824055	0.56949959	1.19E-42
Q99729-3;D6R9P3;D6RD18;D	Heterogeneous nuclear ribo	HNRNPAB	-0.1732769	0.569618652	7.10E-23
Q5SSJ5;B0QZK4;Q5SWC8;Q5	Heterochromatin protein 1-	HP1BP3	-0.3224678	0.570473522	0.0001119
Q9NPA8;G3V117;E5RHX8	Enhancer of yellow 2 transc	ENY2	-0.1727943	0.570556274	7.60E-33
P08708;P0CW22;H0YN88;H0	40S ribosomal protein S17;4	RPS17;RPS17L	0.2267132	0.573729562	8.41E-48
Q86VP6;Q86VP6-2	Cullin-associated NEDD8-dis	CAND1	-0.1699238	0.576148947	5.00E-115
Q13586;E9PQJ4	Stromal interaction molecul	STIM1	-0.3182354	0.576239799	5.80E-18
Q15165;Q15165-3;J3QT77;Q	Serum paraoxonase/arylest	PON2	0.1782379	0.576790907	3.45E-60
Q71UM5;C9JLI6;H0YMV8	40S ribosomal protein S27-l	RPS27L	0.2249889	0.577016563	4.58E-11
P61313;E7EQV9;E7ENU7	60S ribosomal protein L15;F	RPL15	0.1779289	0.577333246	1.62E-13
Q92973;Q92973-2;Q92973-3	Transportin-1	TNPO1	-0.1691132	0.577732945	7.50E-239
Q86V81;E9PB61	THO complex subunit 4	ALYREF	0.2245102	0.577930787	2.82E-113
P35249;H7C1P0	Replication factor C subunit	RFC4	0.1768703	0.579193079	9.70E-28
P11021	78 kDa glucose-regulated pr	HSPA5	-0.1681938	0.579531862	5.80E-242

O43447;C9JQD4;A6NM32;H0	Peptidyl-prolyl cis-trans isor	PPIH	0.1763935	0.580031759	3.08E-08
Q14966;E7EUZ3;Q14966-5;Q	Zinc finger protein 638	ZNF638	-0.1675205	0.580850991	4.05E-39
O43670;J3KS31;O43670-3;O4	Zinc finger protein 207	ZNF207	0.1734238	0.585267831	2.22E-08
Q9UMS4;F5GY56	Pre-mRNA-processing facto	PRPF19	-0.1651421	0.585522133	2.12E-28
P61201;B4DIH5;P61201-2;H0	COP9 signalosome complex	COPS2	-0.1647568	0.586280444	1.26E-64
P36954;K7EKS1	DNA-directed RNA polymera	POLR2I	0.1720371	0.587720173	6.81E-111
Q9NXS2	Glutaminyl-peptide cyclotra	QPCTL	0.1706486	0.590180641	4.14E-62
Q9H936;E9PJH7	Mitochondrial glutamate ca	SLC25A22	0.1705608	0.59033627	6.66E-29
P33993;P33993-3	DNA replication licensing fa	MCM7	-0.162672	0.59039146	9.70E-251
P12236;I7HJJ0;P12235	ADP/ATP translocase 3;ADP	SLC25A6;SLC25A4	0.2176247	0.591154338	3.20E-54
Q14839;F5GWX5;Q14839-2	Chromodomain-helicase-DN	CHD4	-0.3064919	0.592381695	4.72E-66
P62888;E5RI99	60S ribosomal protein L30	RPL30	0.2165756	0.593181156	4.01E-97
Q96RE7	Nucleus accumbens-associa	NACC1	-0.1601524	0.595377671	9.47E-98
Q9Y295	Developmentally-regulated	DRG1	-0.1594162	0.596838276	9.26E-109
P14316;D6RB34;D6R9N5;D6R	Interferon regulatory factor	IRF2;IRF1	-0.3025799	0.597804522	3.64E-15
Q6ZRS2;Q6ZRS2-3;Q6ZRS2-2	Helicase SRCAP	SRCAP	-0.3009453	0.600077084	0.00034704
Q15233;Q15233-2;C9JYS8	Non-POU domain-containin	NONO	-0.157444	0.600758891	3.23E-68
P19388	DNA-directed RNA polymera	POLR2E	-0.1569958	0.60165156	3.35E-82
O00571;O00571-2;O15523;B4	ATP-dependent RNA helicas	DDX3X;DDX3Y	0.1637554	0.602464739	7.87E-23
Q9H446;E5RGQ3;Q9H446-2;E	RWD domain-containing pro	RWDD1	0.1637192	0.602529624	1.28E-05
P07355;P07355-2;H0YMD0;H	Annexin A2;Annexin;Putativ	ANXA2;ANXA2P2	-0.1560078	0.603621341	3.98E-97
Q16656;Q16656-2;Q96AN2;C	Nuclear respiratory factor 1	NRF1	0.2111549	0.603704628	4.17E-21
P0DI83	Ras-related protein Rab-34,	RAB34	0.1624928	0.604727321	3.59E-23
Q9NWS0;M0QYF4;M0QXD5;M	PIH1 domain-containing pro	PIH1D1	0.1607113	0.607926007	7.84E-19
P22392;J3KPD9;P22392-2;Q3	Nucleoside diphosphate kin	NME2;NME1-NME2	-0.2947712	0.608695884	1.76E-39
P62081;B5MCP9	40S ribosomal protein S7	RPS7	0.2077045	0.610446455	6.50E-29
P46013;P46013-2	Antigen KI-67	MKI67	0.1586571	0.611623762	1.51E-06
Q969G3;H7C048;K7EMQ8;C0	SWI/SNF-related matrix-ass	SMARCE1	0.1586533	0.611630638	2.76E-18
P25705;P25705-2;K7EQT2;K7	ATP synthase subunit alpha	ATP5A1	0.2069435	0.611937945	6.26E-46
P19387	DNA-directed RNA polymera	POLR2C	-0.2922707	0.61220215	0.0026766
P62917;G3V1A1;E9PKZ0;E9PI	60S ribosomal protein L8	RPL8	0.204813	0.616121915	2.01E-19
P55084;B5MD38;B4E2W0;F5	Trifunctional enzyme subun	HADHB	-0.1494713	0.61672542	1.76E-134
P61923;F8VWL5;F8VVA7;F8V	Coatomer subunit zeta-1	COPZ1	-0.2881584	0.6179877	5.00E-07
P78316;E9PFK5;P78316-2	Nucleolar protein 14	NOP14	0.1548538	0.618496073	1.39E-27
P30101;G5EA52	Protein disulfide-isomerase	PDIA3	-0.1481476	0.619394237	9.64E-172
Q9BUJ2;Q9BUJ2-4;B7Z4B8;Q	Heterogeneous nuclear ribo	HNRNPUL1	0.2029228	0.619844401	8.27E-94
Q12899;A2AE48;A2AE51	Tripartite motif-containing p	TRIM26	0.1536369	0.620702041	5.94E-21
Q14103;Q14103-3;H0YA96;H	Heterogeneous nuclear ribo	HNRNPD	0.2020302	0.621605747	4.42E-45
Q9UN86;Q9UN86-2;D6RAC7;	Ras GTPase-activating prote	G3BP2	0.201149	0.62334664	6.45E-111
P56537;B7ZBG9;B7ZBH1	Eukaryotic translation initia	EIF6	0.2009964	0.623648308	3.58E-46
P23497;P23497-4;P23497-2;F	Nuclear autoantigen Sp-100	SP100	0.15168	0.624256728	4.28E-35
P26641;B4DTG2;E7EMT2	Elongation factor 1-gamma	EEF1G	-0.1453114	0.625129466	2.82E-55

Q6UN15;Q6UN15-4;Q6UN15	Pre-mRNA 3-end-processing	FIP1L1	0.149437	0.628341885	1.23E-32
Q9H4A6	Golgi phosphoprotein 3	GOLPH3	0.1493359	0.628526265	7.43E-07
Q8NAV1	Pre-mRNA-splicing factor 38	PRPF38A	0.1985092	0.628574363	2.36E-75
P09661;H0YMA0;H0YLR3	U2 small nuclear ribonucleo	SNRPA1	0.1971779	0.631217992	1.33E-121
P35268;K7EJT5;K7EP65;K7EK	60S ribosomal protein L22	RPL22	0.1953831	0.634789444	1.61E-38
P06493;P06493-2;E5RIU6	Cyclin-dependent kinase 1	CDK1	-0.2750092	0.63664569	6.15E-31
Q9NPE3	H/ACA ribonucleoprotein co	NOP10	-0.2749577	0.636719228	5.83E-11
P39656;E7EWT1	Dolichyl-diphosphooligosacc	DDOST	-0.1394062	0.63714324	3.08E-94
P14406;D6RIE3;D6RGV5;H0U	Cytochrome c oxidase subu	COX7A2	-0.2739086	0.63821801	4.81E-16
O75179;O75179-5;H0YMS3;C	Ankyrin repeat domain-con	ANKRD17	-0.2734756	0.63883703	2.56E-10
P62910;D3YTB1;F8W727	60S ribosomal protein L32	RPL32	0.193121	0.639302891	6.73E-93
P30153;B3KQV6;F5H3X9;P30	Serine/threonine-protein ph	PPP2R1A;PPP2R1B	-0.2731342	0.639325333	4.88E-30
Q96QC0;F5H5K4	Serine/threonine-protein ph	PPP1R10	-0.2730598	0.639431744	1.77E-08
Q13547;F5GXM1	Histone deacetylase 1	HDAC1	-0.1382732	0.639459282	6.10E-69
Q9UBW8;F5GYF7;F5H248	COP9 signalosome complex	COPS7A	-0.2717037	0.641372995	9.47E-10
Q9UBV2;Q9UBV2-2	Protein sel-1 homolog 1	SEL1L	0.1419716	0.642019865	3.41E-17
O43491;E9PHY5;E9PPD9;O43	Band 4.1-like protein 2	EPB41L2	-0.1365662	0.642955583	2.16E-85
P32969;E7ESE0;D6RAN4;H0Y	60S ribosomal protein L9	RPL9	0.1907196	0.644108802	1.70E-25
P13010	X-ray repair cross-complem	XRCC5	0.1401768	0.645326732	1.33E-59
Q8IXQ4;Q8IXQ4-2;Q8IXQ4-3	Uncharacterized protein KIA	KIAA1704	0.1392765	0.646988088	6.58E-30
Q9NS69	Mitochondrial import recep	TOMM22	-0.1345081	0.647181208	1.95E-56
Q14676;Q14676-2;E9PGY5;Q	Mediator of DNA damage ch	MDC1	0.1890678	0.647423209	1.22E-172
P46777	60S ribosomal protein L5	RPL5	-0.133831	0.648573972	1.19E-125
Q9UFC0	Leucine-rich repeat and WD	LRWD1	-0.1332798	0.649708709	1.80E-40
P35269;M0R0R9;E7EUG6;M0	General transcription factor	GTF2F1	0.1879292	0.649712166	1.53E-220
Q9UJX3;Q9UJX3-2;F8VZ62	Anaphase-promoting compl	ANAPC7	-0.2657032	0.649991863	2.49E-47
P62318;B4DJP7;H3BT13	Small nuclear ribonucleopro	SNRPD3	0.1866055	0.652377192	2.96E-175
Q96PK6;Q96PK6-3;Q2PYN1;F	RNA-binding protein 14	RBM14;RBM14-RBM	0.1363029	0.652487964	2.93E-39
Q16181;H0Y3Y4;H0YFF6;B4D	Septin-7	Sep-07	0.1863995	0.652792322	3.51E-23
Q9BYP7;Q9BYP7-2;Q9BYP7-3	Serine/threonine-protein ki	WNK3	0.1355515	0.653880938	0.0018478
O14773;O14773-2	Tripeptidyl-peptidase 1	TPP1	0.1352196	0.654496493	6.98E-21
Q92896;Q92896-2;H3BQT1;H	Golgi apparatus protein 1	GLG1	0.1346493	0.655554807	9.66E-07
O75925;B3KSY9;O75928;H3B	E3 SUMO-protein ligase PIA	PIAS1;PIAS2	-0.2603531	0.657716254	2.27E-07
P55060;B4DUC5;P55060-3;F8	Exportin-2	CSE1L	0.1836929	0.658256625	4.45E-18
Q9UHD8;Q9UHD8-2;Q9UHD8	Septin-9	Sep-09	0.1331539	0.658333065	1.82E-89
Q7Z4V5;Q7Z4V5-2;K7EQZ6;I3	Hepatoma-derived growth f	HDGFRP2	0.1830139	0.659630398	1.61E-77
P62701;A6NH36	40S ribosomal protein S4, X	RPS4X	0.182724	0.660217303	7.66E-125
Q04837;C9K0U8;E7EUY5	Single-stranded DNA-bindin	SSBP1	-0.257864	0.661322516	4.80E-32
P06730;D6RBW1;P06730-3;P	Eukaryotic translation initia	EIF4E	-0.2577324	0.661513412	1.03E-10
Q13144;E9PC74	Translation initiation factor	EIF2B5	0.1312313	0.66191205	8.54E-94
Q96A33;Q96A33-2	Coiled-coil domain-containi	CCDC47	0.1818027	0.662083658	1.89E-147
Q15185;B4DP11;B4DP21	Prostaglandin E synthase 3	PTGES3	-0.1272526	0.66216878	1.80E-49

O43390;Q2L7G6;O43390-2;E	Heterogeneous nuclear ribo	HNRNPR		0.1305332	0.663213482	7.35E-48
Q86UE4;E5RJU9	Protein LYRIC	MTDH		0.181015	0.663681193	9.39E-66
P23284	Peptidyl-prolyl cis-trans isor	PPIB		-0.1262035	0.664347176	1.02E-46
P27348	14-3-3 protein theta	YWHAQ		-0.2556744	0.664501435	1.02E-118
Q9Y3B4	Pre-mRNA branch site prote	SF3B14		0.1295147	0.665114115	1.06E-33
Q01105;Q01105-2;Q01105-3	Protein SET	SET		0.1799946	0.665752914	4.70E-142
P25398	40S ribosomal protein S12	RPS12		0.1799107	0.665923412	2.46E-61
Q9BQG0;Q9BQG0-2;I3L1L3	Myb-binding protein 1A	MYBBP1A		0.1277847	0.668347278	3.16E-12
Q13310;Q13310-2;B1ANR0;C	Polyadenylate-binding prote	PABPC4		0.1270809	0.669664411	1.62E-148
Q96C57;E7ENF1;F5H7W8;F5H	Uncharacterized protein C1	C12orf43		0.1777763	0.670265278	8.97E-64
Q05682;Q05682-3;Q05682-2	Caldesmon	CALD1		0.1265316	0.670693124	9.38E-26
Q14202;Q14202-3;A6NL54;A	Zinc finger MYM-type prote	ZMYM3		0.1264877	0.670775305	5.17E-22
Q9UNF1;Q5H909;Q9UNF1-2;	Melanoma-associated antig	MAGED2		-0.2478352	0.675931631	7.12E-24
P33240;E9PID8;P33240-2;E7E	Cleavage stimulation factor	CSTF2;CSTF2T		-0.2477913	0.675995809	1.81E-11
Q92785;B4DT58;J3KMZ8	Zinc finger protein ubi-d4	DPF2		-0.2470989	0.677009022	3.54E-18
Q8NI22;Q8NI22-2;Q8NI22-3;I	Multiple coagulation factor	MCFD2		-0.2470512	0.677078825	3.12E-30
O94776	Metastasis-associated prote	MTA2		0.1741333	0.677701768	3.12E-139
P14314;P14314-2;K7ELL7;K7E	Glucosidase 2 subunit beta	PRKCSH		-0.1196213	0.678079778	4.53E-136
Q9H1C3	Glycosyltransferase 8 doma	GLT8D2		-0.245697	0.679062359	0.00012396
P35606;B4DZI8	Coatomer subunit beta	COPB2		-0.2449875	0.680102508	2.76E-25
P60228;H0YBR5	Eukaryotic translation initia	EIF3E		-0.2444572	0.680880222	6.87E-06
Q9Y333	U6 snRNA-associated Sm-lik	LSM2		0.1197796	0.683387656	1.59E-10
Q8IZL8;F8WDZ1;I3L3A8;C9JF	Proline-, glutamic acid- and	PELP1		-0.2427235	0.683425543	9.95E-05
Q9NQZ2	Something about silencing p	UTP3		-0.2423363	0.683994464	1.29E-111
P14854	Cytochrome c oxidase subu	COX6B1		-0.2416744	0.684967368	2.66E-18
P68363;F8VVB9	Tubulin alpha-1B chain	TUBA1B		-0.1156197	0.686481309	0
Q99848;H7C2Q8	Probable rRNA-processing p	EBNA1BP2		0.1167316	0.689147852	1.88E-58
O00264;B7Z1L3	Membrane-associated prog	PGRMC1		-0.1136589	0.690612243	1.79E-50
Q9Y6H1;Q5T1J5	Coiled-coil-helix-coiled-coil-	CHCHD2;CHCHD2P9		0.1657963	0.694837667	3.09E-18
Q9H089	Large subunit GTPase 1 hom	LSG1		-0.1112919	0.695611394	8.88E-29
Q96IZ7;Q96IZ7-2;H7C5Q0	Serine/Arginine-related pro	RSRC1		-0.2324352	0.698602726	5.15E-43
P38646	Stress-70 protein, mitochon	HSPA9		-0.1096897	0.699002727	6.50E-221
Q15019;C9J2Q4;B5MCX3;Q1	Septin-2		Sep-02	0.1111736	0.699698064	2.11E-27
Q96ST2;Q96ST2-2;Q96ST2-3	Protein IWS1 homolog	IWS1		0.1629524	0.700719318	6.18E-81
Q8WXX5	DnaJ homolog subfamily C r	DNAJC9		-0.2305813	0.701350732	4.29E-13
P10155;Q5LJA0;P10155-3;P1	60 kDa SS-A/Ro ribonucleop	TROVE2		-0.2305012	0.701469562	2.41E-32
P08195;P08195-3;P08195-2;F	4F2 cell-surface antigen hea	SLC3A2		-0.1082458	0.70206412	9.01E-85
O00203;E5RJ68	AP-3 complex subunit beta-	AP3B1		0.1622543	0.702165867	1.18E-86
Q96JP5;Q96JP5-2	E3 ubiquitin-protein ligase Z	ZFP91		0.1092587	0.703346666	0.00086905
P20290;P20290-2;H0Y9Y1;D6	Transcription factor BTF3	BTF3		-0.2289009	0.703844847	3.87E-19
P49792;E9PGT0	E3 SUMO-protein ligase Ran	RANBP2		-0.2285347	0.704388825	8.81E-58
Q13416	Origin recognition complex	ORC2		0.1608658	0.705046361	1.30E-147

P61011;P61011-2;G3V4F7	Signal recognition particle 5	SRP54	0.1605892	0.705620592	0
Q9BXW9;Q9BXW9-2;Q9BXW	Fanconi anemia group D2 p	FANCD2	0.1079617	0.705821722	6.39E-81
P61964	WD repeat-containing prote	WDR5	0.10746	0.706779824	2.83E-50
Q96MX6;Q8ND98;Q96MX6-2	WD repeat-containing prote	WDR92;DKFZp434B	-0.2265987	0.707267044	1.47E-21
P09429;Q5T7C4;Q5T7C6	High mobility group protein	HMGB1	-0.2255936	0.708763104	1.19E-11
P82673;P82673-2	28S ribosomal protein S35,	MRPS35	-0.2254143	0.709030072	2.33E-17
H7BZJ3	Thioredoxin	PDIA3	-0.1049271	0.709119158	1.16E-58
P05141	ADP/ATP translocase 2;ADP	SLC25A5	-0.1043415	0.71036654	5.21E-49
Q99623;Q99623-2;J3KPX7	Prohibitin-2	PHB2	-0.2244186	0.710513245	2.49E-16
P61513;C9J4Z3;M0R0A1;Q6P	60S ribosomal protein L37a	RPL37A	0.1580276	0.710947096	1.40E-23
P14866;P14866-2;M0QXS5;M	Heterogeneous nuclear ribo	HNRNPL	-0.104044	0.711000686	7.28E-125
O94842;B4DPY8	TOX high mobility group bo:	TOX4	-0.2239323	0.711238182	2.93E-22
Q6W2J9;H7C2V9;A6NE70;Q6	BCL-6 corepressor	BCOR	0.1047726	0.711920652	9.93E-34
P62841;K7EM56;K7ELC2;K7E	40S ribosomal protein S15	RPS15	0.157238	0.712591936	2.65E-05
P68371;P04350;A8K854	Tubulin beta-4B chain;Tubu	TUBB4B;TUBB4A;TU	-0.1027985	0.713657326	3.16E-259
Q27J81;Q27J81-2	Inverted formin-2	INF2	0.1027107	0.715873661	6.95E-65
P05387	60S acidic ribosomal protei	RPLP2	0.1554184	0.716387301	8.25E-138
O14974;O14974-4;O14974-3	Protein phosphatase 1 regu	PPP1R12A	0.1553192	0.716594379	2.48E-239
Q13868;A3KFL1;B4DKK6;A3K	Exosome complex compone	EXOSC2	0.1010418	0.719078962	9.47E-15
P46060;F8W7I9	Ran GTPase-activating prote	RANGAP1	0.15341	0.720584671	1.73E-200
Q15554;H3BR06	Telomeric repeat-binding fa	TERF2	-0.09934616	0.721039142	1.88E-34
P55010;H0YLZ1;H0YN40	Eukaryotic translation initia	EIF5	-0.09774017	0.724482041	1.62E-150
Q16531;F5GY55	DNA damage-binding protei	DDB1	0.1507511	0.726154261	5.05E-68
Q9UEE9;Q9UEE9-2	Craniofacial development p	CFDP1	-0.2137089	0.726535618	0.0011316
O60341;O60341-2;F6S0T5	Lysine-specific histone dem	KDM1A	-0.09584045	0.72856184	1.61E-106
Q9UBQ5;K7ES31;K7EMQ9;K7	Eukaryotic translation initia	EIF3K	0.09395981	0.732734856	3.01E-33
P52655;J3KNC0	Transcription initiation fact	GTF2A1	-0.2085304	0.734326486	1.76E-17
Q96DI7;B4DQJ1	U5 small nuclear ribonucleo	SNRNP40	0.145668	0.736842179	4.39E-46
Q9BXP5;Q9BXP5-5;Q9BXP5-4	Serrate RNA effector molec	SRRT	0.1455536	0.737083403	6.68E-70
P10599;P10599-2	Thioredoxin	TXN	0.09165764	0.737192563	4.37E-20
P09012;M0R268;M0QZG7;M	U1 small nuclear ribonucleo	SNRPA	-0.09111404	0.738745377	4.58E-101
P62306;F8W0W6	Small nuclear ribonucleopro	SNRPF	0.14431	0.739706375	8.07E-77
Q9NYB0	Telomeric repeat-binding fa	TERF2IP	0.1433029	0.741832722	2.20E-72
Q15459;E9PAW1	Splicing factor 3A subunit 1	SF3A1	-0.08943176	0.742381221	1.76E-221
Q02078;Q14814;Q06413;Q02	Myocyte-specific enhancer	MEF2A;MEF2D;MEF	-0.2025204	0.743402844	4.23E-06
O95782;O95782-2;O94973;O	AP-2 complex subunit alpha	AP2A1;AP2A2	0.08697701	0.746282875	2.19E-13
P68400;E7EU96;P68400-2	Casein kinase II subunit alph	CSNK2A1	-0.08728981	0.747018855	3.00E-302
Q9UQ35	Serine/arginine repetitive m	SRRM2	0.1406288	0.74748835	1.17E-150
P11279;B4DWL3	Lysosome-associated memb	LAMP1	-0.1987228	0.749156389	6.55E-07
Q9BT78;D6RFN0;D6RD63;Q9	COP9 signalosome complex	COPS4	0.1394329	0.750022076	7.90E-70
P55884;P55884-2	Eukaryotic translation initia	EIF3B	-0.1973572	0.751228906	1.67E-09
Q14160;Q14160-3;Q14160-2	Protein scribble homolog	SCRIB	0.1375523	0.754011984	3.49E-97

Q6P1J9	Parafibromin	CDC73	-0.08321762	0.755860907	3.02E-42
P38432	Coilin	COIL	0.08181	0.756358895	1.71E-26
P40939	Trifunctional enzyme subun	HADHA	-0.08258629	0.757234637	9.51E-175
O60237;F8W8M3;O60237-4;I	Protein phosphatase 1 regu	PPP1R12B	-0.1929302	0.757959496	8.51E-09
Q13243;Q13243-3;B4DJK0;B4	Serine/arginine-rich splicing	SRSF5	-0.08213425	0.758218715	4.90E-66
O43852;O43852-3;O43852-4,	Calumenin	CALU	-0.08208656	0.758322544	1.38E-72
P04406;E7EUT4;E7EUT5	Glyceraldehyde-3-phosphat	GAPDH	0.1350765	0.759274432	8.58E-122
P19784;H3BSA1;H3BV19	Casein kinase II subunit alph	CSNK2A2	-0.08158112	0.759423395	1.06E-203
P00403	Cytochrome c oxidase subu	MT-CO2	-0.1911392	0.760687725	2.13E-07
P42704	Leucine-rich PPR motif-cont	LRPPRC	-0.08002472	0.762816239	8.47E-73
Q5T8P6;Q5T8P6-3;Q5T8P6-2,	RNA-binding protein 26	RBM26	-0.1890259	0.763910806	7.51E-12
Q9NR30;Q9NR30-2	Nucleolar RNA helicase 2	DDX21	0.1322861	0.765219214	2.46E-41
Q9BTT0;Q5TB20;E9PPH5;Q5T	Acidic leucine-rich nuclear p	ANP32E	-0.07776451	0.767751456	2.92E-206
O94992	Protein HEXIM1	HEXIM1	-0.1854286	0.769406417	4.43E-07
Q9NZJ4;Q9NZJ4-2	Sacsin	SACS	-0.07531166	0.77311798	0.0067645
Q9NW82;D6RIW8	WD repeat-containing prote	WDR70	0.1280079	0.774360269	6.27E-61
Q9Y5B9	FACT complex subunit SPT1	SUPT16H	0.127285	0.775907967	2.04E-165
O43143	Putative pre-mRNA-splicing	DHX15	0.07170868	0.776175824	3.99E-09
P14678;P63162;P14678-2;B3	Small nuclear ribonucleopro	SNRPB;SNRPN	-0.07205582	0.780258102	1.04E-127
Q9P0L0;Q9P0L0-2	Vesicle-associated membra	VAPA	0.0693264	0.780871266	9.25E-12
O75822;B4DUI3;F5H425	Eukaryotic translation initia	EIF3J	-0.0716095	0.781238349	1.48E-35
Q92769;B3KRS5;J3KPW7	Histone deacetylase 2;Histo	HDAC2	0.06877518	0.781958867	5.87E-23
Q15061;C9IZK7	WD repeat-containing prote	WDR43	-0.07111549	0.782323729	3.79E-35
P04844;P04844-2	Dolichyl-diphosphooligosac	RPN2	0.1239796	0.782996165	1.28E-298
Q9BY77;Q9BY77-2;F8WCX5	Polymerase delta-interactin	POLDIP3	0.06766891	0.784142882	2.59E-09
P68366;A8MUB1	Tubulin alpha-4A chain	TUBA4A	0.06751442	0.784448026	2.45E-237
P32519;P32519-2	ETS-related transcription fa	ELF1	0.06737328	0.78472683	1.82E-53
P52701;F5H2F9;B4DF41;P527	DNA mismatch repair prote	MSH6	-0.06948471	0.78590974	2.09E-250
Q6UX04;D6REK3;Q6UX04-2	Peptidyl-prolyl cis-trans isor	CWC27	0.06654167	0.786370067	4.26E-16
P62995;P62995-3;H7BXF3	Transformer-2 protein hom	TRA2B	-0.06889534	0.787206859	4.08E-24
Q12797;F5H667	Aspartyl/asparaginyl beta-h	ASPH	0.1212387	0.788887404	4.15E-66
P62847;P62847-2;E7ETK0;P6	40S ribosomal protein S24	RPS24	0.1210899	0.789207528	3.48E-16
P43307;C9J3L8;C9J5W0;E9PA	Translocon-associated prote	SSR1	0.1208191	0.789790409	2.51E-72
Q7Z5K2;Q7Z5K2-2;Q7Z5K2-3;	Wings apart-like protein hor	WAPAL	0.1206646	0.79012295	7.54E-88
O43172;O43172-2	U4/U6 small nuclear ribonu	PRPF4	0.1197777	0.792032721	9.22E-45
O60832;H7C2Q9;H7C0M1;C9	H/ACA ribonucleoprotein co	DKC1	-0.1706657	0.792078718	9.42E-14
Q9Y2W1	Thyroid hormone receptor-a	THRAP3	0.118454	0.794885322	1.64E-243
O43395;E7EVD1	U4/U6 small nuclear ribonu	PRPF3	-0.06531525	0.795098691	6.30E-44
O43719	HIV Tat-specific factor 1	HTATSF1	0.1179848	0.795897136	1.37E-134
P08240;P08240-2	Signal recognition particle r	SRPR	0.06170082	0.795954199	7.53E-29
P98175-2;P98175;P98175-3;F	RNA-binding protein 10	RBM10	-0.06477547	0.796290405	0
P40426;B7Z5Q0;P40426-2;P4	Pre-B-cell leukemia transcri	PBX3	-0.1662941	0.798827375	2.44E-11

Q8WX92	Negative elongation factor I	NELFB	-0.1660385	0.7992224	5.28E-35
P46783;F6U211	40S ribosomal protein S10	RPS10	-0.06334496	0.79945095	2.84E-62
P62136;P62136-2;A6NNR3;E9	Serine/threonine-protein pl	PPP1CA	-0.163929	0.80248479	1.28E-151
P08621;P08621-2;P08621-3;I	U1 small nuclear ribonucleo	SNRNP70	0.05830193	0.802702019	1.34E-10
O95365	Zinc finger and BTB domain-	ZBTB7A	-0.1632156	0.803588767	5.80E-10
P27824;B4DGP8;B4E2T8	Calnexin	CANX	-0.06116486	0.804273927	1.02E-211
Q15029;Q15029-2;K7EJ81	116 kDa U5 small nuclear ril	EFTUD2	-0.06023026	0.806343816	2.56E-84
P78527;E7EUY0;P78527-2	DNA-dependent protein kin	PRKDC	0.05605698	0.807167027	1.61E-67
P25490;H0YJV7	Transcriptional repressor pr	YY1	0.112505	0.807738952	3.24E-37
O14617;O14617-5;O14617-4,	AP-3 complex subunit delta-	AP3D1	-0.1598892	0.808741868	4.48E-35
P01011;G3V3A0;G3V5I3	Alpha-1-antichymotrypsin;A	SERPINA3	-0.15979	0.808895644	0.012304
P27797;K7EJB9	Calreticulin	CALR	-0.05867958	0.809781129	2.23E-136
Q16629;Q16629-3;Q16629-2	Serine/arginine-rich splicing	SRSF7	0.1114922	0.809932526	6.92E-35
Q9H6T3;Q9H6T3-3;Q9H6T3-2	RNA polymerase II-associate	RPAP3	-0.1585522	0.810815507	3.92E-30
Q9Y2W2;F5H721	WW domain-binding protei	WBP11	0.1104546	0.812181353	3.53E-299
O15391	Transcription factor YY2	YY2	-0.1566963	0.813695953	2.35E-13
Q96B01;Q96B01-3;Q96B01-2	RAD51-associated protein 1	RAD51AP1	-0.05657005	0.814463112	4.39E-16
P06454;P06454-2;B8ZZQ6	Prothymosin alpha;Prothym	PTMA	-0.05621529	0.815251155	0.00067329
P51610;P51610-3;P51610-2;A	Host cell factor 1;HCF N-teri	HCFC1	-0.1534405	0.818755384	8.09E-06
Q9NR50;H0Y580;Q9NR50-3;C	Translation initiation factor	EIF2B3	-0.05404854	0.820068173	5.74E-36
P52292	Importin subunit alpha-1	KPNA2	0.1067047	0.820321341	2.64E-185
P25205;J3KQ69;B4DWW4	DNA replication licensing fa	MCM3	0.04728127	0.824680214	1.32E-70
P42568;B7Z755	Protein AF-9	MLLT3	0.04674911	0.825745103	0.00010234
P61353;K7EQQ9;K7ELC7	60S ribosomal protein L27	RPL27	-0.05112076	0.826587851	1.30E-14
P35613;P35613-2;P35613-4;I	Basigin	BSG	-0.0495491	0.830092633	7.13E-132
Q92766;C9JU34;Q92766-3;Q!	Ras-responsive element-bin	RREB1	-0.1458015	0.830655241	6.55E-08
P20645;H0YGT2;H0YF90	Cation-dependent mannose	M6PR	0.04370499	0.831842828	2.60E-17
Q8WYA6;B4DE16;Q8WYA6-3	Beta-catenin-like protein 1	CTNNBL1	-0.04647827	0.83695027	3.78E-263
Q01650	Large neutral amino acids tı	SLC7A5	0.04049683	0.838280092	8.50E-43
P63165;B8ZZN6;B8ZZJ0;B8ZZ	Small ubiquitin-related moc	SUMO1	0.03909683	0.841092642	1.60E-05
Q9UKV3;E7EQT4;Q9UKV3-5	Apoptotic chromatin conde	ACIN1	0.096838	0.841829999	5.35E-223
Q8IX15;F8WCA3	Homeobox and leucine zipp	HOMEZ	0.03853989	0.842212097	4.63E-22
P49790;F6QR24	Nuclear pore complex prote	NUP153	0.03821373	0.842867817	1.78E-34
P0C0S5;Q71UI9;Q71UI9-3;Q7	Histone H2A.Z;Histone H2A.	H2AFZ;H2AFV	0.03821373	0.842867817	1.04E-14
Q9NYL9;H0YKU1;H0YNJ8	Tropomodulin-3	TMOD3	-0.1375275	0.843588397	4.24E-67
Q9UGP8	Translocation protein SEC63	SEC63	-0.04309464	0.844520803	4.01E-267
Q07021;I3L3Q7;I3L3B0	Complement component 1 (	C1QBP	-0.04268074	0.84544785	8.98E-112
P13073;Q86WV2;H3BN72;H3	Cytochrome c oxidase subu	COX4I1	-0.1351719	0.847278268	1.21E-12
P07910;P07910-4;P07910-2;(	Heterogeneous nuclear ribc	HNRNPC;HNRNPCL1	0.09394836	0.848152345	1.49E-53
O60763;O60763-2;F5H4X1;F5	General vesicular transport	USO1	-0.04133034	0.848473984	5.03E-111
P10809	60 kDa heat shock protein, ı	HSPD1	-0.04074097	0.849795415	1.58E-112
Q9BRJ6;C9JQV0;H7C0T1;H7C	Uncharacterized protein C7	C7orf50	0.03206444	0.855250523	7.45E-19

H0YNU2;H0YNW9;H0YMH7;C	CTD small phosphatase-like	CTDSPL2	0.03134155	0.856708588	0.00018703
Q9H307;Q9H307-2	Pinin	PNN	0.08990479	0.857015793	6.53E-66
O00629	Importin subunit alpha-3	KPNA4	0.08955956	0.857773384	1.74E-53
Q9BWJ5	Splicing factor 3B subunit 5	SF3B5	-0.03499413	0.862702006	3.25E-21
Q13185	Chromobox protein homolo	CBX3	0.08728409	0.86277003	4.85E-77
P42167;G5E972;P42167-2;A2	Lamina-associated polypept	TMPO	-0.03475189	0.863246858	2.61E-56
P46776;E9PJD9;E9PLL6	60S ribosomal protein L27a	RPL27A	-0.03367996	0.865658716	1.50E-23
P09234	U1 small nuclear ribonuclec	SNRPC	-0.03295517	0.86729022	8.19E-84
P55209;F8VUX1;F8VY35;B3KI	Nucleosome assembly prote	NAP1L1	-0.0328064	0.867625177	1.16E-134
P37275;H0YMD3;H0YK86;H0	Zinc finger E-box-binding hc	ZEB1	-0.1219692	0.868019246	2.57E-07
P62316;P62316-2	Small nuclear ribonucleopro	SNRPD2	-0.03245544	0.868415428	6.59E-109
Q68E01;Q68E01-2;Q68E01-3	Integrator complex subunit	INTS3	-0.03174591	0.870013511	1.85E-160
Q7L1Q6;Q7L1Q6-4;Q7L1Q6-3	Basic leucine zipper and W2	BZW1	-0.03147507	0.870623672	1.41E-15
Q9NWV8;M0QY17;Q9NWV8-	BRISC and BRCA1-A comple	BABAM1	-0.1186523	0.873244855	4.87E-35
P52434;C9JBJ6;C9JLU1;C9JU	DNA-directed RNA polymer	POLR2H	0.02291298	0.873743915	5.77E-39
P13984	General transcription factor	GTF2F2	-0.02958107	0.874892634	7.60E-105
O60684;F5H4G7;F5GYL8;O15	Importin subunit alpha-7;Im	KPNA6;KPNA5	0.08142471	0.87566132	6.49E-97
Q14257;A8MXP8;F8WCY5	Reticulocalbin-2	RCN2	0.02070808	0.878210326	0.00026448
P20674;H3BNX8;H3BRI0	Cytochrome c oxidase subu	COX5A	-0.1153507	0.878451922	2.60E-19
P16615;P16615-5;P16615-2;F	Sarcoplasmic/endoplasmic r	ATP2A2	0.07977295	0.879301511	3.06E-136
P52739;D6RJH2;D6R9I2;P527	Zinc finger protein 131	ZNF131	0.01998901	0.879667778	4.26E-13
P39687;H0YN26	Acidic leucine-rich nuclear p	ANP32A	0.07938194	0.880163595	1.83E-143
Q9NYF8;Q9NYF8-2;Q9NYF8-4	Bcl-2-associated transcriptio	BCLAF1	0.07927704	0.88039491	4.04E-235
P61244;P61244-2;Q6V3B1;P6	Protein max	MAX	-0.1132469	0.881772646	0.0002923
P62269;Q5GGW2;J3JS69	40S ribosomal protein S18	RPS18	0.07734299	0.884661329	1.07E-15
P62891;Q59GN2	60S ribosomal protein L39;F	RPL39;RPL39P5	0.07692719	0.885579009	0.0016918
Q9BQE9;Q9BQE9-4;Q4VC05;(	B-cell CLL/lymphoma 7 prot	BCL7B;BCL7A	-0.1104794	0.886144226	3.66E-05
Q99933;Q99933-4;Q99933-3	BAG family molecular chape	BAG1	0.01579666	0.888173035	2.29E-06
Q9Y2S0	DNA-directed RNA polymer	POLR1D	-0.1091595	0.888230316	8.98E-106
P62979;P0CG47;J3QS39;J3QT	Ubiquitin-40S ribosomal pro	RPS27A;UBB;UBC;U	0.07549095	0.888749964	3.78E-66
P29692;E9PK01;P29692-2;P2	Elongation factor 1-delta	EEF1D	0.07520866	0.889373417	1.27E-98
Q06587;Q06587-2	E3 ubiquitin-protein ligase F	RING1	-0.02306366	0.889609591	8.88E-110
E9PGZ2;Q32MZ4;Q32MZ4-3;	Leucine-rich repeat flightles	LRRFIP1	0.01491928	0.889954689	0.0001358
Q9UNS2;J3QL22;J3QS85;J3KT	COP9 signalosome complex	COPS3	-0.1065044	0.892428944	1.81E-27
P30508;E7ERM2;P30510;Q29	HLA class I histocompatibilit	HLA-C	-0.02166367	0.892776064	1.02E-172
O00422;H7BZW6	Histone deacetylase comple	SAP18	-0.1059265	0.893343274	0.00099171
P63173;J3KT73;J3QL01	60S ribosomal protein L38	RPL38	-0.02047348	0.895469345	1.46E-28
O95251;O95251-4;E7EUP3;G	Histone acetyltransferase K	KAT7	0.01203728	0.8958109	9.43E-30
P05455;E9PFH8;E7ERC4;E9P(	Lupus La protein	SSB	0.07208252	0.89628223	3.68E-181
Q9BUF5	Tubulin beta-6 chain	TUBB6	-0.1019478	0.899641749	1.32E-43
Q9GZS3;H0YL19;H0YN81	WD repeat-containing prote	WDR61	0.07016373	0.900526716	7.94E-46
Q07157;Q07157-2;G5E9E7;G	Tight junction protein ZO-1	TJP1	-0.1012669	0.90072032	1.10E-12

Q8NCA5;E9PH82;Q8NCA5-2;I	Protein FAM98A	FAM98A	-0.1011734	0.900868374	2.49E-08
Q8NC51;Q8NC51-2	Plasminogen activator inhib	SERBP1	0.06994629	0.901007883	7.19E-155
P18887;F5H8D7	DNA repair protein XRCC1	XRCC1	-0.01652145	0.904420893	6.19E-108
Q13435;E9PPJ0;E9PJ04;H0YC	Splicing factor 3B subunit 2	SF3B2	-0.01500893	0.907850127	1.21E-84
Q9Y3U8	60S ribosomal protein L36	RPL36	0.06638336	0.908897326	9.69E-22
Q9ULU4;B7ZM62;H7C4X9;Q9	Protein kinase C-binding pro	ZMYND8	0.005449295	0.909218466	3.72E-25
Q96HY6;Q96HY6-2	DDRGK domain-containing p	DDRGK1	-0.09568977	0.909561059	5.34E-106
P30050;P30050-2	60S ribosomal protein L12	RPL12	-0.01410294	0.909905035	7.43E-91
O75496;H7C608;E2QRF9	Geminin	GMNN	-0.09457588	0.911328167	2.90E-07
Q8NFC6	Biorientation of chromosom	BOD1L1	0.004323959	0.911511394	4.88E-81
O75821;K7EL20;K7ENA8	Eukaryotic translation initia	EIF3G	0.06482506	0.912350774	9.57E-105
Q9Y678	Coatomer subunit gamma-1	COPG1	-0.09332275	0.913316686	4.83E-61
P62899;H7C2W9;C9JU56;B7Z	60S ribosomal protein L31	RPL31	-0.01239014	0.913791529	1.35E-72
Q8TAQ2;Q8TAQ2-2;Q8TAQ2-	SWI/SNF complex subunit S	SMARCC2;SMARCC1	0.003141403	0.913921706	1.19E-64
P11142;E9PKE3;P11142-2;E9	Heat shock cognate 71 kDa	HSPA8	-0.01221085	0.914198476	3.41E-247
Q9NQC3;Q9NQC3-2;Q9NQC3	Reticulon-4	RTN4	0.06396294	0.914262088	7.63E-48
E7EWI9;P51991-2;P51991	Heterogeneous nuclear ribo	HNRNPA3	0.06396103	0.914266317	4.75E-28
P62750;H7BY10;K7EJV9;K7EF	60S ribosomal protein L23a	RPL23A	0.06363297	0.914993763	1.41E-59
O43707;D6PXK4;F5GXS2;H7C	Alpha-actinin-4	ACTN4	-0.01152611	0.915752876	7.96E-65
Q15007;Q15007-2	Pre-mRNA-splicing regulato	WTAP	-0.09107399	0.916886478	6.28E-110
Q9Y262;B0QY90;Q9Y262-2;J3	Eukaryotic translation initia	EIF3L	0.001293182	0.917690368	1.93E-16
P49755	Transmembrane emp24 dor	TMED10	-0.009723663	0.919846065	4.48E-123
Q9H8H0;J3QLQ6;J3QR28;J3Q	Nucleolar protein 11	NOL11	-0.000377655	0.921098927	1.27E-18
Q9BVI4;F5H303	Nucleolar complex protein 4	NOC4L	-0.08795929	0.921833652	9.84E-08
Q9HCY8	Protein S100-A14	S100A14	-0.08720207	0.923036828	1.20E-32
Q9Y2T2	AP-3 complex subunit mu-1	AP3M1	0.05960274	0.92393597	2.63E-46
P24534	Elongation factor 1-beta	EEF1B2	-0.007616043	0.924634969	6.56E-49
P26196	Probable ATP-dependent RI	DDX6	-0.08458328	0.927199279	1.23E-15
P42677;Q5T4L4	40S ribosomal protein S27	RPS27	-0.006458282	0.927266789	2.15E-12
O00483	NADH dehydrogenase [ubiq	NDUFA4	-0.08410454	0.927960441	2.44E-06
P23246;P23246-2	Splicing factor, proline- and	SFPQ	0.0571804	0.929315307	6.48E-62
Q3B726	DNA-directed RNA polymera	TWISTNB	-0.004526138	0.929568094	5.63E-08
O94874;O94874-2;O94874-3	E3 UFM1-protein ligase 1	UFL1	-0.004617691	0.929755093	7.42E-23
Q9Y3Y2;Q5T7Y7;Q9Y3Y2-4;Q	Chromatin target of PRMT1	CHTOP	-0.08276558	0.930089616	0.005041
P33991;J3KPV4	DNA replication licensing fa	MCM4	-0.004106522	0.932615231	0
P53618;E9PP73	Coatomer subunit beta	COPB1	-0.08109093	0.932753305	7.04E-37
Q13330;Q13330-3;Q13330-2	Metastasis-associated prote	MTA1	-0.006292343	0.933176296	1.99E-07
P83731;C9JXB8;C9JNW5	60S ribosomal protein L24	RPL24	-0.00352478	0.933938722	5.41E-45
Q8WYP5;Q8WYP5-2;Q8WYP5	Protein ELYS	AHCTF1	-0.006904602	0.934427412	9.24E-98
Q7Z417	Nuclear fragile X mental ret	NUFIP2	-0.007385254	0.93540971	3.39E-19
O15042;O15042-2;E7ET15;O1	U2 snRNP-associated SURP	U2SURP	-0.002750397	0.935700767	2.07E-68
P43246;P43246-2;E9PHA6	DNA mismatch repair prote	MSH2	-0.002447128	0.936390916	5.75E-144

Q9ULC4;Q9ULC4-2;Q9ULC4-3	Malignant T-cell-amplified s	MCTS1	-0.00888443	0.93847417	1.12E-19
P55795	Heterogeneous nuclear ribo	HNRNPH2	-0.009338379	0.939402267	3.74E-39
Q8N6N3;Q8N6N3-2;Q8N6N3	UPF0690 protein C1orf52	C1orf52	-0.07532501	0.941930102	1.73E-55
P47914	60S ribosomal protein L29	RPL29	-0.01091003	0.942616138	1.52E-06
Q86T24	Transcriptional regulator Ka	ZBTB33	0.001764297	0.945979574	3.69E-43
Q08945	FACT complex subunit SSRP	SSRP1	0.002552032	0.947774016	1.44E-124
Q96D15	Reticulocalbin-3	RCN3	0.002790451	0.948317181	2.88E-58
Q52LJ0;Q52LJ0-2	Protein FAM98B	FAM98B	-0.06971169	0.950871469	7.00E-21
Q02543;M0R0P7;M0R3D6;M	60S ribosomal protein L18a	RPL18A	-0.06857109	0.952689094	8.90E-10
P62875	DNA-directed RNA polymer	POLR2L	-0.06810951	0.953424725	1.23E-19
P20020;P20020-5;P20020-2;P	Plasma membrane calcium-	ATP2B1;ATP2B4	-0.01680374	0.954676053	0.00018443
K7ELV2;Q96EE3;Q96EE3-1	Nucleoporin SEH1	SEH1L	-0.01756477	0.95623412	0.0032213
Q15366;Q15366-4;Q15366-5	Poly(rC)-binding protein 2	PCBP2	-0.0199852	0.961190558	2.60E-14
P17693;Q5RJ85	HLA class I histocompatibilit	HLA-G	-0.02008629	0.961397598	9.89E-05
P68104;Q5VTE0;Q05639	Elongation factor 1-alpha 1;	EEF1A1;EEF1A1P5;E	0.008897781	0.962238369	4.21E-44
Q86YP4;H7C3H1;F5H7D9;B4D	Transcriptional repressor p6	GATAD2A	-0.02067375	0.962600828	8.96E-34
Q9UBU8;H0YMJ0;H0YLJ3;Q9U	Mortality factor 4-like prote	MORF4L1	-0.02085495	0.962971971	4.70E-08
O60216	Double-strand-break repair	RAD21	-0.06113052	0.964551825	7.06E-06
Q68CQ4	Digestive organ expansion f	DIEXF	0.01025391	0.965331264	1.40E-149
Q09028;Q09028-3;Q09028-4	Histone-binding protein RBI	RBBP4	0.04037666	0.966707406	3.61E-63
P08107;P08107-2;F8VZJ4;E7E	Heat shock 70 kDa protein 1	HSPA1A	0.03998947	0.967570176	3.93E-81
P01040;C9J0E4	Cystatin-A;Cystatin-A, N-ter	CSTA	-0.02334595	0.968074994	1.07E-34
Q5SRQ6;P67870;Q5SRQ3	Casein kinase II subunit beta	CSNK2B;CSNK2B-LY6	0.03932953	0.969040796	2.23E-141
Q9NY27;Q9NY27-2;Q9NY27-3	Serine/threonine-protein ph	PPP4R2	-0.0563755	0.972137181	2.18E-59
Q92572;F5H459	AP-3 complex subunit sigma	AP3S1	0.03792191	0.972177899	3.02E-77
O60506;O60506-4;O60506-3	Heterogeneous nuclear ribo	SYNCRIP	0.03667068	0.974966798	3.85E-67
Q99627;H7C3S9;Q99627-2;E9	COP9 signalosome complex	COPS8	-0.0268898	0.975337086	1.37E-14
O75489;E9PKL8;G3V194;B4D	NADH dehydrogenase [ubiq	NDUFS3	-0.05381393	0.976224565	0.00034405
Q03701	CCAAT/enhancer-binding pr	CEBPZ	-0.02822495	0.978073636	1.49E-17
Q9UHB9;F5H5Y3;Q9UHB9-2;C	Signal recognition particle s	SRP68	0.03491402	0.978882818	1.64E-171
P09874	Poly [ADP-ribose] polymera	PARP1	0.01641655	0.979392197	1.48E-116
Q8IYB3;A9Z1X7;Q8IYB3-2;E9I	Serine/arginine repetitive m	SRRM1	0.03458786	0.979609956	8.28E-90
Q96DH6;Q96DH6-2;B4DHE8;	RNA-binding protein Musas	MSI2	-0.02915955	0.979989377	9.19E-32
P12270	Nucleoprotein TPR	TPR	-0.05107307	0.980598724	6.19E-65
P11387	DNA topoisomerase 1	TOP1	-0.02968788	0.981072408	1.76E-50
Q15910;Q15910-2;Q15910-3	Histone-lysine N-methyltrar	EZH2	-0.0301075	0.981932603	4.15E-06
Q13112	Chromatin assembly factor	CHAF1B	-0.04926872	0.983478629	3.99E-83
P18621;J3QQT2;J3KRX5;J3QL	60S ribosomal protein L17	RPL17	0.01831245	0.98371946	1.24E-51
Q12888;C9JXV0;A6NNK5;F8V	Tumor suppressor p53-bind	TP53BP1	0.01889229	0.98504299	4.50E-154
O75475;O75475-2;O75475-3	PC4 and SFRS1-interacting p	PSIP1	0.01928711	0.985944233	5.91E-116
P17844;B4DLW8;J3KTA4	Probable ATP-dependent RI	DDX5	-0.03232574	0.986480229	6.77E-22
Q01664	Transcription factor AP-4	TFAP4	0.01989174	0.987324432	2.43E-170

Q9GZT3;H0YJW7;H0YJ40;G3\	SRA stem-loop-interacting F	SLIRP	0.03067398	0.988336755	1.92E-32
O43818	U3 small nucleolar RNA-inte	RRP9	-0.04467392	0.990813226	1.99E-69
P49916;P49916-2;K7EQB6;K7	DNA ligase 3	LIG3	-0.04457474	0.990971559	2.40E-150
Q14694;Q14694-3;Q14694-2	Ubiquitin carboxyl-terminal	USP10	0.02303505	0.994500244	8.33E-63
Q6UXN9	WD repeat-containing prote	WDR82	-0.03627014	0.994567626	3.13E-36
Q9UHR5;Q9UHR5-2;J3QQJ0;J	SAP30-binding protein	SAP30BP	0.0231781	0.994826828	7.65E-65
Q9Y3D9;J3QLR8	28S ribosomal protein S23,	MRPS23	-0.04171562	0.99553595	2.38E-07
Q13011;M0QZW4;M0R248	Delta(3,5)-Delta(2,4)-dienoy	ECH1	-0.04046059	0.997539587	0.013709
P53999	Activated RNA polymerase I	SUB1	0.02602196	0.998711128	9.40E-82
Q9C005;B4DIS3	Protein dpy-30 homolog	DPY30;LOC84661	-0.03948593	0.999095614	2.48E-05
Q12824;Q12824-2;B5MCL5;G	SWI/SNF-related matrix-ass	SMARCB1	-0.03891945	1	1.43E-33
P63220;Q8WVC2;Q9BYK1	40S ribosomal protein S21	RPS21	0.02544403	1	3.37E-46
